# Hidden Chromosome Symmetry: *In Silico* Transformation Reveals Symmetry in 2D DNA Walk Trajectories of 671 Chromosomes

**DOI:** 10.1371/journal.pone.0006396

**Published:** 2009-07-28

**Authors:** Maria S. Poptsova, Sergei A. Larionov, Eugeny V. Ryadchenko, Sergei D. Rybalko, Ilya A. Zakharov, Alexander Loskutov

**Affiliations:** 1 University of Connecticut, Storrs, Connecticut, United States of America; 2 Physics Faculty, Moscow State University, Moscow, Russia; 3 Vavilov Institute of General Genetics, Moscow, Russia; Cairo University, Egypt

## Abstract

Maps of 2D DNA walk of 671 examined chromosomes show composition complexity change from symmetrical half-turn in bacteria to pseudo-random trajectories in archaea, fungi and humans. In silico transformation of gene order and strand position returns most of the analyzed chromosomes to a symmetrical bacterial-like state with one transition point. The transformed chromosomal sequences also reveal remarkable segmental compositional symmetry between regions from different strands located equidistantly from the transition point. Despite extensive chromosome rearrangement the relation of gene numbers on opposite strands for chromosomes of different taxa varies in narrow limits around unity with Pearson coefficient r = 0.98. Similar relation is observed for total genes' length (r = 0.86) and cumulative GC (r = 0.95) and AT (r = 0.97) skews. This is also true for human coding sequences (CDS), which comprise only several percent of the entire chromosome length. We found that frequency distributions of the length of gene clusters, continuously located on the same strand, have close values for both strands. Eukaryotic gene distribution is believed to be non-random. Contribution of different subsystems to the noted symmetries and distributions, and evolutionary aspects of symmetry are discussed.

## Introduction

2D DNA walk is a method of DNA sequence representation on a plane whereby a trajectory is drawn, nucleotide after nucleotide, in four directions: G-up, C-down, T-left, A-right [1]. This method has proven to be useful in detection of origins of replication in bacteria [2], [3]. More recently it was shown that 2D DNA walk representation can be applied as a database interface for analysis and identification of many functional structures in chromosome sequences, such as subtelomeres, transposons, imperfect gigantic palindromes, gene clusters and areas of large scale polymorphisms (CNP and segmental duplication) [4]. The existence of chromosome trajectories, i.e., deviation from a point, confirms a deviation from the 2^nd^ Chargaff's parity rule, stating that in a single stranded DNA A = T and G = C [5]–[7]. This phenomenon is known as skew, which is calculated as cumulative values of (G−C)/(G+C) and (T−A)/(T+A) over the chromosome length [8]. Unlike skew graphics, the 2D DNA walk method can detect, along with the effect of nucleotide accumulation, a large-scale correlation in nucleotide composition [4].

**Figure 1:**
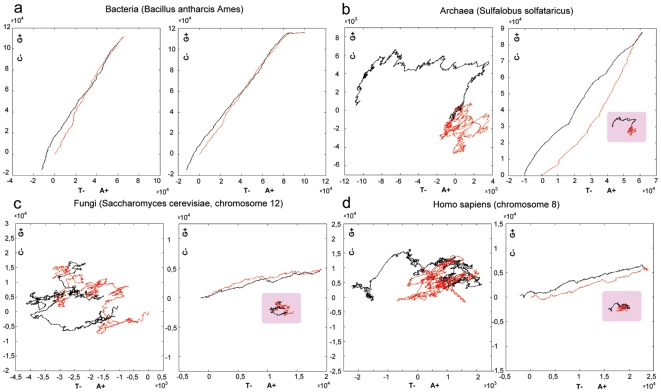
2D DNA graphs of all genes from a chromosome for different organisms. (a) - bacteria (*Bacillus anthracis Ames*), (b) - archaea (*Sulfalobus solfataricus*), (c)–fungi (*Saccharomyces cerevisiae*, chromosome 12), (d) - *Homo sapiens* (chromosome 8). Trajectories of the genes in the original order are shown on the left, GSS transformed trajectories on the right.

2D DNA walks of most bacterial chromosomes have a symmetrical linear form with one transition point [9] (Figure 1a). Accumulation of one nucleotide compared to its complementary is usually observed on one half of the bacterial chromosome, which corresponds to the 2D DNA trajectory before the transition point. After the transition point the same nucleotide is reduced by the value of its initial accumulation, and the trajectory nearly returns to its starting point. This phenomenon was given the name of compositional asymmetry of bacterial chromosomes, and the property was used in the Oriloc tool [10] for prediction of origins of replication in bacteria. Bacterial compositional asymmetry was explained by compositional asymmetry of leading and lagging strands [11], [12]. In those bacteria, which have a single replication origin, leading and lagging strands are defined at Ori-site in the inverse way, so that one half of a single-stranded DNA is a leading and another is a lagging strand. The leading strand tends to accumulate G over C, while the preference for accumulation of T or A may vary between species.

Different explanations were given to explain why the leading strand should have a skewed composition, and these may roughly be subdivided into three groups based on the underlying mechanisms, which are replication-coupled, transcription-coupled and codon-/amino-acid-usage coupled [13]. One explanation consists in the different way of replication for the two strands–the leading strand replicating continuously, and the lagging strand replicating through Okazaki fragments. Cytosine deamination hypothesis [13] states that the leading strand, which is more often single-stranded during the replication stage than the lagging strand, undergoes C->T mutations, that would lead to diminishing of C, and hence to excess of G on the leading strand. On the other hand, different selective mutation pressure was reported for genes located on different strands, and the genes on the lagging strand accumulate more mutations [11], [14]. For a comprehensive review of the mechanisms that could contribute to compositional strand biases in bacteria and discussion of their role, see [13], [15].

It was also noticed that the GC-skews often correlate with gene orientation, or the so-called CDS skews [16]. The first experimental evidence that transcriptional units have a skewed composition was reported by Szybalski [17], who noticed that mRNA transcripts tend to be purine-rich. Forsdyke proposed the idea that purine loading happens mostly in loops of the stem-loop structures, which are formed in single stranded RNA or DNA, and that genes preserve a high potential for folding in stem-loop structures from the times of an RNA-world [18]. The reason for loading loops with one type of nucleotide is to avoid unnecessary interaction on RNA-level and avoid formation of double-stranded DNA[19]. On the other hand genes are composed of amino acids, which are encoded by codons. Trifonov [20] found a universal three-base periodical pattern (G-non-G-N) in protein coding sequences and suggested that this pattern in mRNA may be responsible for monitoring the correct reading frame during translation. In turn C-periodic regions were found at three positions of the small subunit rRNA in *E. coli* [21], [22] suggesting that a proofreading mechanism of mRNA-rRNA interactions is based on a preference for G in the first codon position. Amino acid composition of genes combined with a specific codon usage could explain the tendency of genes to have an excess of one nucleotide over the other. In the early work of [23] it was proposed that the choice of a codon is specific not at the gene level, but at the level of individual genomes. It is thought that a genome possesses a specific codon usage bias. Inside a genome codon usage bias correlates with gene expression, and this correlation is measured as codon adaptation index [24]. Later work of [25] reports domains of specific codon usage that are clustered together in a close regions on a chromosome. To the best of our knowledge, no comprehensive study has been performed of the extent to which codon usage bias is responsible for creating a skew on the leading strand.

The idea that GC skews are always related to replication and can help to detect the origins of replication was extended to the eukaryotes [26]. However these results are controversial, and not all experimentally confirmed replicons have distinctive GC skews. The 2D DNA walk trajectories of the entire chromosomes of eukaryotes look like a blend of linear and tangled trajectories [4] (Figure 1), which cannot be divided into symmetrical trajectories (similar to those in bacteria) to correspond to replication units (see Discussion below). Other studies showed transcription-associated mutation asymmetry in mammals [27] where coding regions tend to accumulate G+T over A+C. This asymmetry results from transcription-coupled repair mechanisms. TA and GC strand asymmetries were reported for human genes [28], and the mechanism responsible for this asymmetry was attributed to transcription. It is however inexplicable why, despite the local asymmetries, chromosomes on the whole comply with the Chargaff's second parity rule. In the present work we address the question of Chargaff's second parity rule and the deviation from it on the level of coding sequences for different groups of species.

**Figure 2:**
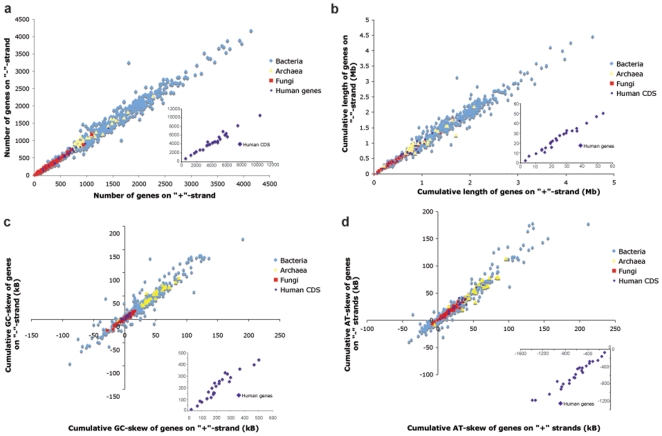
Relations between number, cumulative gene length, GC and AT skews of genes from different strands. Relation between (a) number (Pearson coefficient r = 0.98), (b) cumulative gene length (r = 0.86), (c) cumulative GC (r = 0.95) and (d) AT (r = 0.97) skews of genes from different strands for 671 chromosomes: 524 bacteria, 36 archaea, 87 fungi and 24 humans.

We found that an *in silico* transformation reverts a tangled trajectory to a symmetrical linear form with one transition point, similar to the one we observe in bacteria. The transformation consists in merging genes from one strand, without changing their order, with concatenated genes from the other strand excluding intergenic regions [29]. For the majority of chromosomes, such trajectories of genes sorted by strands (GSS) become either linear or polygonal lines with a distinctive slope and a point of half-turn (Figure 1 and Figure S1). The transformed chromosomal sequences also reveal significant segmental compositional symmetry between regions from different strands, which are located equidistantly from the transition point. A surprising outcome of that transformation was the finding whereby most of the examined 671 chromosomes of bacteria (524), archaea (36), fungi (87) and humans (24) have close numbers of genes on different strands showing a strong correlation with Pearson coefficient r = 0.98. Moreover, an approximate equality with a strong correlation was also observed for the total gene length (r = 0.86) and cumulative GC (r = 0.95) and AT (r = 0.97) skews (Figure 2 and Table S1). This is also true for human CDS sequences, which comprise only several percent of the full chromosome length. We found that on different strands the frequency distributions of the length of gene clusters, continuously located on the same strand, have close values. We suppose, that eukaryotic gene distribution is non-random. Below we discuss contributions of different subsystems that are responsible for noted symmetries and distributions.

## Results

### Concept of gene-vector on 2D DNA plane and G-rich genes

**Figure 3:**
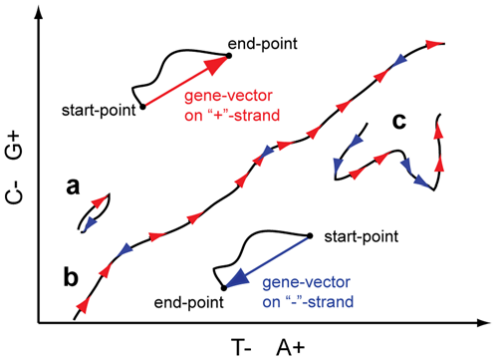
Illustration of gene-vector model on a 2D DNA plane. See text for explanation. Symmetrically correlated regions are drawn in the same color.

To illustrate the emerging symmetry of rearranged chromosomes we introduce a concept of graphical gene-vector on a 2D DNA plane (Figure 3). Each gene can be schematically represented as a vector (G−C, A−T), which has a length and a direction, corresponding to cumulative compositional imbalances. The DNA trajectory on 2D DNA walk can then be approximated by gene-vectors similar to a piecewise linear function. Whole chromosome trajectories are drawn for single-stranded DNA, while genes can be encoded on both strands. This means that for genes from the complementary strand a reverse complement sequence has to be taken. Gene-vectors' direction on «+»-strand is defined by the sign of (G−C) and (T−A) and by the opposite sign on «−»-strand. If two genes with similar cumulative composition are consecutively encoded on different strands, then the trajectory will go forth and return back to its starting point (Figure 3A). The number of unidirectional genes sums up into a line (Figure 3B). Genes with different compositions and from different strands create a wandering curve (Figure 3C). Most of the observed GSS trajectories represent lines. It means that on average they consist of unidirectional genes. Moreover, the majority of the genes are G-rich while property of A- or T-richness fluctuates from species to species (see Tables 1–2 for percent distribution of G-rich and A-rich genes in different groups of species). We also see a high percentage (>95%) of G in the first position (G1 in Table 2) of codons in the genes of bacteria, archaea and fungi (∼62% for humans ), that corresponds to a high percentage of amino acids whose codons start with G: Val, Ala, Asp, Glu, Gly. Indeed, these amino acids are among the most ubiquitous. Val and Ala are small hydrophobic amino acids, which participate in the folding nucleation. Asp and Glu are negatively charged and often substitute each other, and most globular proteins have negative charges on their surface. Gly is the smallest amino acid in globular proteins and is often found in the loops and folds of secondary structures. If G-richness of genes mostly corresponds to a biased amino acid composition remains an open question. Why are G1-codons so widespread? As it was mentioned above, preference for G in the first codon position was attributed to the translation mechanics [20]. It was also shown that preference for G in the first codon position correlates with the gene expression in *E. coli* [30] suggesting that the stickiness to the ribosome might influence the rate of translation. Lately, it was re-observed that the first amino acids are predominantly those codified by codons of the type GNN and that an early version of a primitive genetic code might only have the codons of the type GNC [31]. Also, it is interesting to note that exons in human genome are A-rich, but genes, which consist of exons plus introns, are T-rich. Hence, introns in human genome are T-rich. This fact is clearly seen on a 2D DNA walk of GSS sequences of human chromosomes (Table S1 and Figure S1).

**Table 1:** Percent distribution of G-rich and A-rich genes in 671 chromosomes^1^.

group of organisms	total genes	g-pos	g-neg	a-pos	a-neg
bacteria	1590581	67.5	32.5	62.46	37.54
archaea	77937	84.2	15.8	79.62	20.38
fungi	40375	65.79	34.21	74.92	25.08
human_cds	199087	59.7	40.3	65.92	34.08
human_genes	28658	67.84	32.16	31.99	68.01

1 The values of g-pos (G−C>0), g-neg (G−C<0), a-pos (A−T>0), a–neg (A−T<0) were calculated for the entire gene sequences.

**Table 2:** Percent distribution of G-rich and A-rich genes for the 1^st^, 2^nd^, and 3^rd^ codon positions in 671 chromosomes^2^.

group of organisms	total genes	g1-pos	a1-pos	g2-pos	a2-pos	g3-pos	a3-pos
bacteria	1590581	94.87	90.71	18.66	50.87	46.25	27.15
archaea	77937	98.28	94.96	28.21	56.51	51.3	55.76
fungi	40375	94.52	93.2	11.24	77.97	50.75	23.86
human_cds	199087	61.77	65.8	49.92	54.16	59.53	52.06
human_genes	28658	68.46	34.31	64.55	31.25	65.91	30.16

2 The values of g-pos (G−C>0), a-pos (A−T>0) were calculated for the 1^st^, 2^nd^, and 3^rd^ codon positions. The values of g-neg and a–neg are calculated as 100% - g-pos and 100% - a-pos.

It is known that bacteria and some eukaryotic genomes have a distinct GC-skew. It is also known that archaea and eukaryotes do not possess a distinctive GC skew (Figure 1). Interestingly, the GSS transformation not only reinstates GC-skew to the chromosomes of archaea, fungi and humans, but also allows distinguishing a uniform gene direction characteristic for the entire chromosome. Local regions that deviate from an average chromosomal gene-direction can be indicative of insertions, or areas of codon usage bias, or regions of different mutation pressure. Surprisingly, GSS transformation reveals large-scale correlation of such deviated regions.

### Emergence of correlated symmetry of 2D DNA walk trajectories after GSS transformation

**Figure 4:**
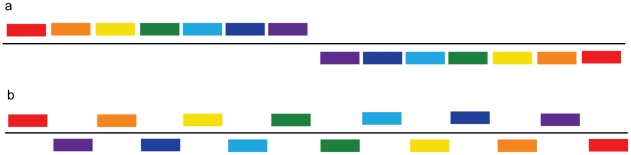
Cluster model of chromosome organization. (a)–chromosome clusters after GSS transformation, (b)–chromosome clusters in the original order.

**Figure 5:**
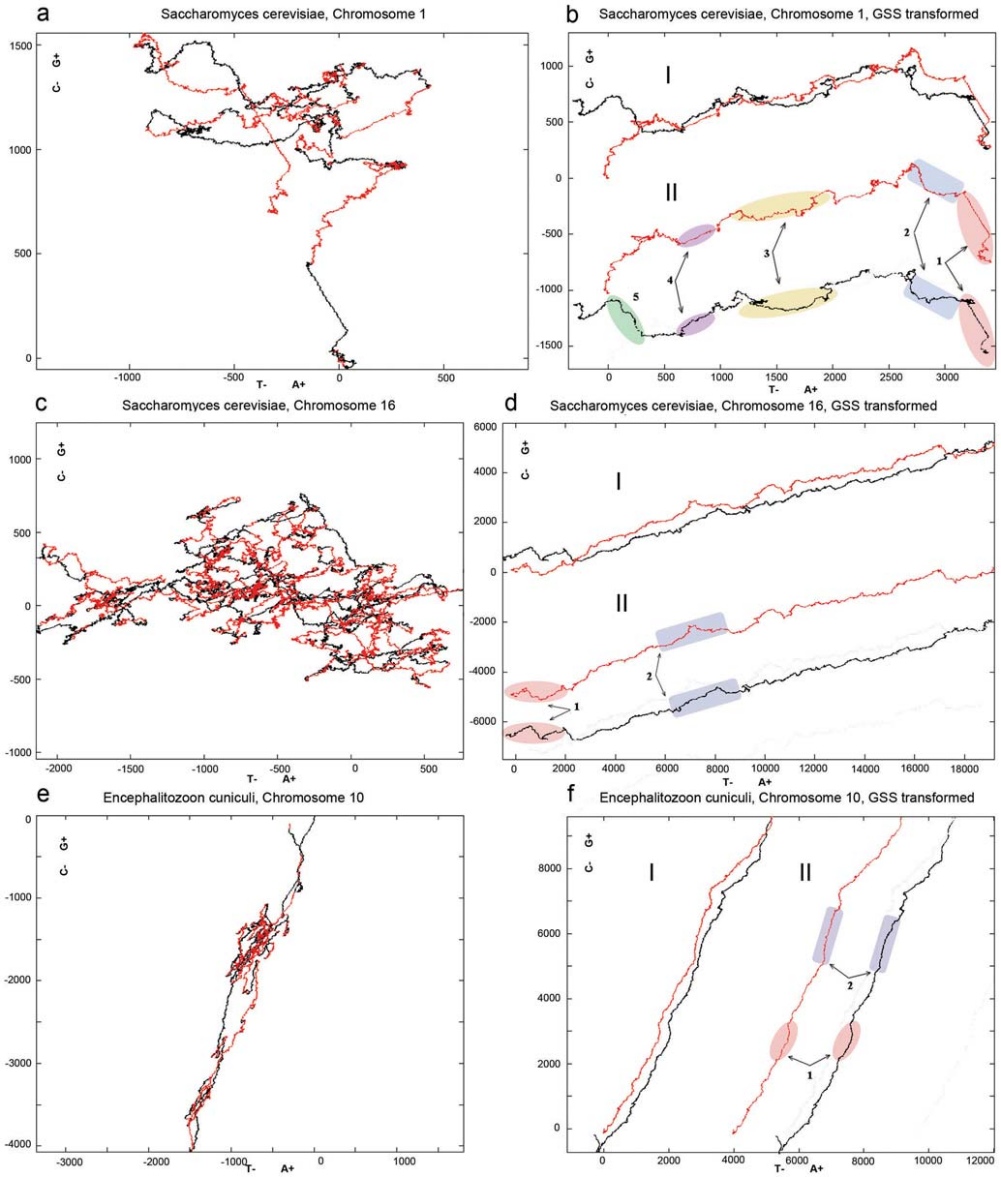
Emergence of correlated symmetry of 2D DNA walk trajectories after GSS transformation. (a–b)–*Saccharomyces cerevisiae*, chromosome 1, - (c–d)–*Saccharomyces cerevisiae*, chromosome 16, (e–f)–*Encephalitozoon cuniculi*, chromosome 10. (a,c,e)–2D DNA walk maps of chromosomes made of genes in the original order; genes from «+»-strand are colored in red, genes from «−»-strand are colored in black. (b,d,f)–GSS-transformed trajectories, I–in the original form, II–two arms of GSS-transformed trajectories are detached to show symmetrical correlated regions, which are highlighted and numbered (see blast hits for these regions in Table S2).

An illustration of GSS transformation algorithm that leads to a symmetrical effect on 2D DNA walk map is given in Figure 4. Symmetrically correlated regions are drawn in the same color. If we observe correlated symmetry of regions (composed of genes or gene clusters) located equidistantly from the transition point (Figure 4A), then the position of genes (gene clusters) in the original order in chromosome should comply with Figure 4B. Figure 5 shows examples of emerging correlated symmetry for two chromosomes of *Saccharomyces cerevisiae* and one of *Encephalitozoon cuniculi*. Ellipses highlight symmetrically correlated areas located on the opposite strands. After GSS transformation some of these regions form the so-called strand “metasequences” that show correlated symmetrical properties of a 2D DNA walk trajectory (Figure 5). Some of these regions contain BLAST hits (Table S2) of the whole genes or domains, but some (regions 3 and 4 for *Saccharomyces cerevisiae*, chrom. 1 (Figure 5b) and region 2 of *Encephalitozoon cuniculi*, chrom. X (Figure 5f)) do not have any hits. Cases of intra-chromosomal duplications in subtelomeric regions in fungi were reported in [32], and region 1 in *Saccharomyces cerevisiae*, chrom. 1 (Figure 5b) contain such an example of duplication of flo-family genes. Regions that do not have significant BLAST hits may still represent the remnants of duplicated areas. As it was pointed out in [32], a significant part of the duplications in fungi could be concealed as a result of an intensive mutation process. Correlated areas of 2D DNA walk maps may potentially reveal cases of unrecoginzed paralogy, and detected hits, however scarce, could serve as seeds for distant similarity searches. We can see that correlated sequences in a GSS-transformed trajectory are more often located equidistantly from the transition point. However some of the symmetrical pairs have one site close to the transition point and another at the beginning or in the end of the chromosome sequence. One of the possible explanations for such symmetry is that it could emerge as a result of duplication, which takes place during the recombination process in subtelomeric sites of repeats. This type of duplication can organize paralogs distantly and also locally, in a palindromic way on different strands. However, correlated areas located in the central regions of the GSS transformed trajectories could be a result of a similar compositional structure of a locus, from where the genes from the different strands were separated. A region in *Saccharomyces cerevisiae*, highlighted with a green ellipse, indicates an area where the trajectory sharply changes direction (Fig. 5B). This particular region corresponds to a retrotransposon and is indicative of insertion. In general, the regions where DNA trajectory deviates from the common direction may potentially contain recently horizontally transferred genes [33], [34].

### Gene distribution over strands

The symmetrical trajectory is distinctly divided into two branches where one arm corresponds to “+”-strand and the other to “−”-strand. For the most of the analyzed chromosomes these arms are equal in length implying the equality of cumulative GC and AT skews for genes located on different strands. We found that this equality originates from the equality of the number of genes on different strands that, in some cases, takes place even if the values of cumulative GC and AT skews are not well correlated (Figure 2 and Table S1). It is known that gene distribution over strands can be locally asymmetrical as, for example, is the case with bacteria where genes are preferentially located on the leading strand, or in fungi, where we see distinct clusters of genes with their relative preference for one strand. Moreover chromosomes undergo extensive rearrangements such as duplications, deletions, insertions, and inversions. In this respect the overall balance of gene number on different strands over diverse groups of genomes such as bacteria, archaea, fungi and humans seems to be a constraint on chromosome organization and can be regarded as an evolutionary invariant.

**Figure 6:**
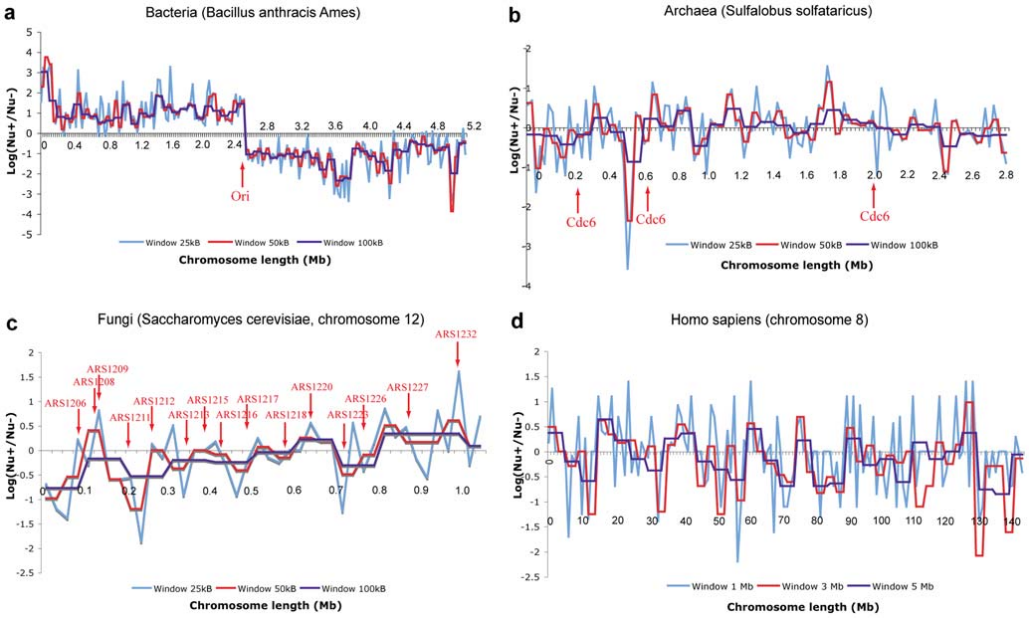
Gene distribution over strands in the entire chromosome. (a) - bacteria (*Bacillus anthracis Ames*), (b) - archaea (*Sulfalobus solfataricus*), (c) - fungi (*Saccharomyces cerevisiae*, chromosome 12), (d) - *Homo sapiens* (chromosome 8). Red arrows point to the positions of the known origins of replications.

**Figure 7:**
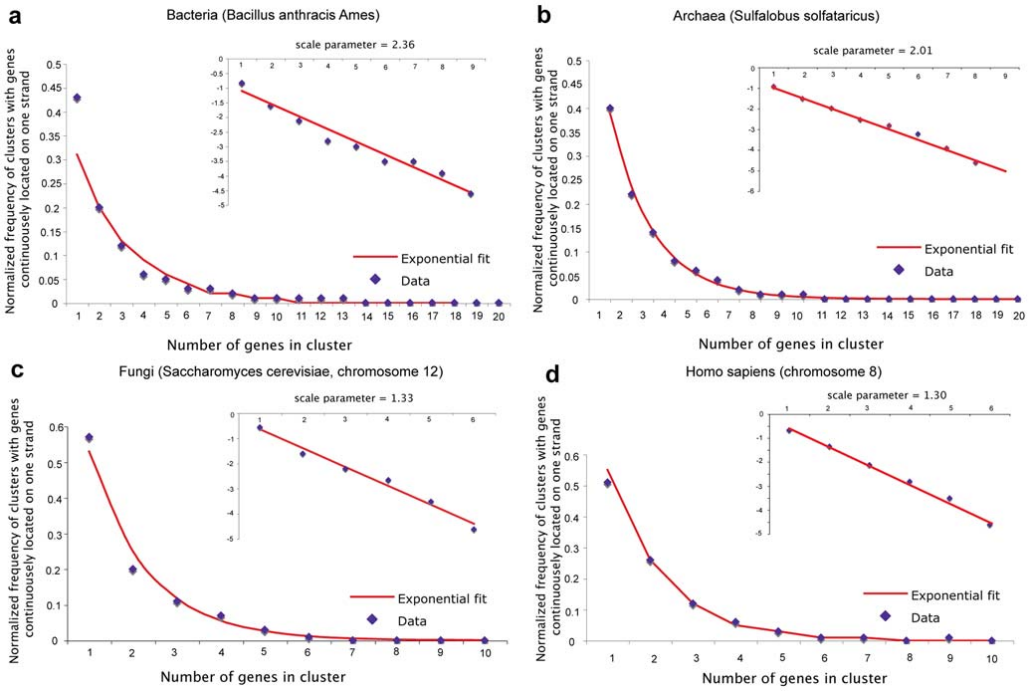
Normalized frequency distributions of clusters with genes continuously located on one strand. Data are approximated by exponential functions with scale parameter beta = 2.36 for (a) bacteria (*Bacillus anthracis Ames*), beta = 2.01 (b) for archaea (*Sulfalobus solfataricus*), beta = 1.33 (c) for fungi (*Saccharomyces cerevisiae*, chromosome 12), beta = 1.3 (d) for *Homo sapiens* (chromosome 8).

To obtain a detailed picture of how genes are distributed over strands on the scale of the whole chromosome, we performed the following analyses. Gene distribution over strands in the whole chromosome is depicted as logarithms of gene number ratio calculated for some sets of windows (Figure 6). Positive values reveal regions where the number of genes on the “+”-strand exceeds that on the “−”-strand, and negative values reveal those with the opposite ratio. Most of bacterial chromosomes show a distinct two-sided symmetry even before transformation. Starting from archaea, gene regions with preferential location on either strand are diminishing in number. Analysis of frequency distribution of the length of such regions showed that they fit exponential distribution (Figure 7) with high level of single- and double-gene clusters. The abundance of small size gene clusters observed on the same strand might be due to bidirectional transcription regulation [35]. The latest estimate of the number of bidirectionally transcribed pairs in yeast increase their share from 30 to 66% [36]. Superposition of bidirectional promoter density with gene distribution over strands for *S. cerevisiae*, chromosome 12, is depicted in Figure S2. Though the correlation is weak (correlation coefficient of 0.3), one can see that the maximum of bidirectional promoter density often falls on the regions where the logarithm of gene number ratio is close to zero, implying local equilibrium of genes from both strands.

It is also important that bacteria and archaea have longer distribution tails than fungi and humans. Thus, the maximum length of a cluster where genes are continuously positioned on one strand exceeds 50 genes in bacterium *Mesoplasma florum*, approaches 20 in archaeon *Sulfolobus solfataricus*, and does not exceed 10 in the known assembled chromosomes of fungi and humans. From bacteria to humans we distinctly see a tendency of genes for a greater intermixture between strands. Frequency distribution of the length of gene clusters calculated for two strands separately showed that they have close values (Figure S3), demonstrating that, on the scale of the whole chromosome, two strands are equally probable for gene location. It is however unexpected that in bacteria, archaea and fungi, despite the difference and local imbalances in strand gene distribution, there exists an overall strand gene balance. In bacteria, gene strand equality is maintained between leading and lagging strands of different replichores (halves) (Figure S4). In fungi we observe relatively large areas of genes with preferential location on either strand, but on the scale of the entire chromosome we observe equilibrium (Figure 6). In humans, according to gene cluster length analysis, one would think that gene strand location is random. Indeed, one would obtain the same results if gene distribution over strands were to be ascribed to chance. Until recently it was common to assume that eukaryotic gene order is random. However, there is increasing evidence for non-randomness of gene order in different groups of organisms including eukaryotes [37], [38]. The position effect and its relation to the diseases was documented [39], and transcription analysis revealed co-expression clusters [40]. Several mechanisms, such as replication, transcription and expression can possibly be responsible for the observed gene distribution, however the impact of each process can be different in different groups of species.

## Discussion

### Bacteria

Compositional symmetry of bacterial chromosomes has been observed since the time of sequencing of the first bacterial genome [2], [15]. The major contribution to the skew is attributed to the symmetry of replication initiated from a single Ori-site. The leading and lagging strands are defined at Ori in the inverse way, so that one half of a single-stranded DNA is the leading and another is the lagging strand. In bacteria genes are preferentially located on the leading strand [15]. In prokaryotes genes make up 95% of the genome so that for bacteria gene-vector approximation almost coincides with the chromosome trajectory. Thus the original chromosome symmetry could be explained by replicon symmetry with genes preferably located on the leading strand. GSS transformation may increase the initial symmetry even more, revealing unidirectional regions of equal length (Figure 1A and Figure S1). These regions correspond to genes on different strands in different replichores. Various gene-vector directions on two strands are generated by different strand-dependent mutation pressure [16]. To what extent a codon usage bias is responsible for the common gene-vector direction remains an open question. Theoretically, one gene-direction can be obtained by various combinations and numbers of codons. The fact that many bacteria have the same angle of 2D DNA trajectories does not mean that they have the same codon usage table, just as well as we cannot claim, without an additional analysis, that the chromosomal regions of different directions are the result of a specific codon usage. These regions could originate from different mutational pressure.

Selection pressure for the balanced genome structure was reported in [41] where authors show that ori-ter imbalance is associated with a reduced growth rate in *Salmonella* genomes. The authors also suggested that insertion of big (>100 kB) pathogenicity islands might trigger genome rearrangement processes in order to reach the ori-ter balance. Surprisingly, approximate gene number balance, equal total length and cumulative skews are also found between two replichores for corresponding strands (Table S3 and Figure S4), which, in our opinion, cannot be explained by equal replication arms alone. Replication arms may be equal, the proportion of genes on corresponding strands could be different, but we observe equality. Not all bacteria show initial symmetry in 2D DNA walk or in nucleotide skew. There are species whose 2D DNA walks have a point of turn, but trajectories do not return to the starting point (*Bordetella parapertussis* in Figure 1; in terms of skew, the graph usually do not return to zero in such cases). It is interesting that the global strand gene number balance also holds true for this type of chromosomes. As we found out, the trajectory is cut because some genes are C-rich and, consequently, gene-vectors have an opposite direction (opposite compositional skews), and this effect partially neutralizes the trajectory. The GSS transformation also revealed an additional symmetry of correlated regions from different strands pointing to the areas with specific codon-usage and/or areas of different mutation pressure [25]. It is also known that recombination processes may cause tandem duplications [42] locally and, in our view, distantly on different strands [43], [44].

### Archaea

A break of symmetry in nucleotide composition is observed in the majority of archaea that are lacking a distinctive nucleotide skew [15]. Indeed, archaeal chromosomes have asymmetrical and tangled DNA trajectories (Figure 1B and Figure S1). After GSS transformation, trajectory becomes a symmetrical curve with a distinctive slope revealing gene-vector's unidirectionality. One of the distinctions of archaea from the majority of bacteria is that archaea can have multiple replication origins. However these origins are distributed non-uniformly with resulting replicons having different length and non-equal arms (see position of Ori on 2D DNA walk of *Sulfolobus solfataricus* in Figure S5). Contrary to the widely held opinion that replicons should show a distinct symmetry in nucleotide skews, replicons of *Sulfolobus solfataricus* do not show asymmetrical gene distribution on leading strands as is typical for bacteria (Figure 1B, Figure 6B). Even if genes in archaea are more shuffled between strands in comparison to those in most of bacteria (Figure 6B), archaeal GSS-transformed trajectories appear more straightened than those in bacteria (Figure S1). This phenomenon is explained by the fact that 84% of the analyzed archaeal genes are G-rich, and 80% are A-rich, and on the large scale gene-vectors have uniform directions. The absence of regions with different slopes could be explained by either absence of areas of different composition or by quick assimilation of foreign DNA with the composition of its host. Symmetrically correlated areas are also observed for archaeal GSS-transformed trajectories, though they are not as obvious as those in other groups.

### Fungi

Fungi do not show a noticeable GC skew and have tangled trajectories. The curves resulting from GSS transformation are polygonal lines, which can still be characterized by a distinctive slope (Figure 1C and Figure S1). Fungi genomes have multiple origins of replications that are distributed non-uniformely [45] (see position of ARS on 2D DNA walks of all chromosomes of *Saccharomyces cerevisiae* in Figure S5). Analysis of gene distribution over strands showed that positive or negative regions corresponding to preferential gene location on one strand considerably exceed replicon boundaries (Figure 6C). Characteristic example is presented for chromosome 12 of *Saccharomyces cerevisiae* (Figure 1C). Large inverted duplications and gigantic “quasi-palindromes” observed in the original chromosomes (Figure S1) also do not coincide with the known replicons [4]. These regions are formed by transposons or inverted duplications of functional clusters [4], [46], [47]. Duplications in fungi can be a part of the whole genome duplication [48], but were also found as large inverted repeats, which are located at the ends of the chromosomes, on opposite strands in subtelomeric regions [32], [49]. Some contain genes as in *Saccharomyces cerevisiae*, some contain short tandem repeats of non-coding regions [4]. As for the gene distribution, the evidence was reported for gene clustering according to different functional criteria in fungi [50], especially for co-expression. Cluster organization related to cell cycle was reported for 25% of co-expressed genes in *Saccharomyces cerevisiae* [51]. We suppose that the observed regions of preferential gene location may include co-expression clusters.

GSS-transformed trajectories of fungi reveal many correlated symmetrical regions (Figure 5). Some of these regions contain gene duplications, which, when re-arranged back to the normal order, are still located symmetrically relative to the middle point of the chromosome (Figure 4). Chromosome compositional symmetry is observed only for bacteria. It was found that in bacteria genes switch their location and strands symmetrically in respect to Ori [52]. In fungi, however, we observe gene duplications, which are symmetrical with respect to the middle point of a chromosome that has multiple Ori. In bacteria this effect was attributed to replication machinery [52] that is symmetrical with respect to Ori. In fungi, however, symmetrical duplication in subtelomeric regions is attributed to recombination processes [32], [49]. It is interesting to note that for some fungi chromosomes (Figure 5), correlation sites are located far from subtelomeric areas and such correlated sequences may be extended along the entire chromosome length, though it is difficult to define the exact position of subtelomeric borders. We need to allow for a possibility that other mechanisms also contribute to symmetrical chromosome re-arrangements. Such mechanisms include chromosome packaging in super-coiled loops that have to be symmetrically located at specific 3D cell locations and can interact because of their physical proximity [53]. Despite different mechanisms of replication between bacteria and fungi (i.e., one Ori versus multiple Oris), the symmetry of re-arrangements and gene duplications with respect to the middle point of a chromosome likely indicates a common evolutionary process that maintains the symmetry in both prokaryotes and eukaryotes.

### Humans

The distinctive feature of human chromosome organization is that genes comprise several percent of the whole chromosome. The GSS-transformed trajectories made solely of CDS become symmetrical with one point of half-turn (Figure 1D). Human chromosomes have multiple replication origins, but their locations are still to be confirmed experimentally. Similar to fungi and archaea, changes in nucleotide skews in humans do not necessarily coincide with Ori-sites [4]. Human genes are characterized by the presence of large introns with an average number of 7–8 per gene [54]. It is interesting that GSS transformation made on whole genes (introns plus exons) reveals the same symmetry (Figure S1). Moreover, both CDS and genes exhibited the same strand-specific global balance in number, total length and cumulative skews (Table S1). Equality of the number of exons and the equality of the number of whole genes (introns plus exons) on two strands implies the equality of the number of introns. Persistence of such a balance over broad range of taxa from bacteria to humans points to an ancient correlation between introns and exons, which is probably explained by the exon theory of genes [55]. Gene density distribution in humans differs essentially from that of fungi, archaea and bacteria, showing areas of high and low gene density [56]. In the regions of high gene density genes are well mixed between strands (Figure 6D). Such gene distribution is most likely due to the peculiarity of transcription machinery in humans, namely to preference for bidirectional transcription, which is connected with expression regulation. Bidirectional transcription was already reported in prokaryotes [57], and often one of the transcribed genes is a regulator of the other. Lately more data were accumulated confirming the abundance of bidirectional transcription in eukaryotes [36], [58].

Cluster organization of co-expressed genes was also found to be true for human chromosomes [40], [59], [60], though these do not have noticeable regions where genes prefer to be located on one strand. Transcription-associated mutational asymmetry is observed in human genome [61], however in general, human replicon composition is not symmetrical [4]. It was shown that some large replicons (L>2 Mb) in human genome in the original form have a symmetrical compositional structure of imperfect giant pseudo-palindrome [4]. It is noteworthy that in human genome there exist regions of gigantic imperfect palindromes partially made of inverted repeats with genes [4], [62] that have a symmetrical compositional structure and can contribute to GSS transformation effect.

Correlated symmetry of GSS-transformed trajectories is also observed for humans, though it is not as evident as with fungi. Human GSS-transformed trajectories are polygonal lines with distinct areas of genes having different directions of their corresponding gene-vectors. 68% of human genes are G-rich (60% for CDS) so that cumulative skew is observed for GSS-transformed human trajectories. It is interesting that A-rich genes and A-rich CDS are in reverse proportion, i.e. only 32% of genes are A-rich, while for CDS this number equals 66%. That is why DNA trajectories have different directions in respect to A−T axes for genes and CDS (Figure S1).

### Conclusions

We suppose that seemingly random processes of chromosome rearrangement (insertions, deletions, duplications, and inversions) may have a restriction in the form of gene number equality on different strands. The latter could point to an existence of an evolutionary invariant in chromosome organization that is related to principles of transcription regulation in taxa as diverse as bacteria and humans [63]. Probably, at an early phase of progenome evolution the number of genes on two strands was statistically adjusted parallel to the unfolding of recombination processes associated with gene reshuffling and intron formation [55], [64], and possible selective advantages of such strategy may be fixed in the evolution of chromosomes.

We suggest that some of the symmetrically correlated patterns revealed via GSS transformation for almost all chromosomes of the investigated groups of organisms–bacteria, archaea, fungi and humans–could point to the existence of a common mechanism that is responsible for symmetrical gene-translocation and duplications. Even if this mechanism is assumed to be either replication or recombination, or both, it could also be some other process that generates the observed symmetry. Perhaps the global gene number balance on the strands also has common origin for prokaryotes and eukaryotes.

Our results shed light on the 2^nd^ Chargaff's parity rule that was previously applied to DNA sequence containing both genes and intergenic regions. We demonstrate that this rule holds true for DNA sequences made up solely of genes and is strongly correlated with the equal number of genes on strands. Besides, our research shows that the absence or presence of nucleotide skews in chromosomes can be explained by the location of genes on strands, and that the majority of the investigated genes (coding sequences) are G- and A-rich.

## Materials and Methods

### Method of 2D DNA walk

Complete genome annotation and sequences of 671 chromosomes of bacteria (524), archaea (36), fungi (87) and humans (build 36.2) were downloaded from NCBI ftp-site: ftp://ftp.ncbi.nlm.nih.gov/genomes/. Gene and CDS boundaries and their position on strands were retrieved from gene location tables (.ptt files) for bacteria, archaea and fungi, and from seq_contig.md file, reference assembly, for humans.

**Figure 8:**
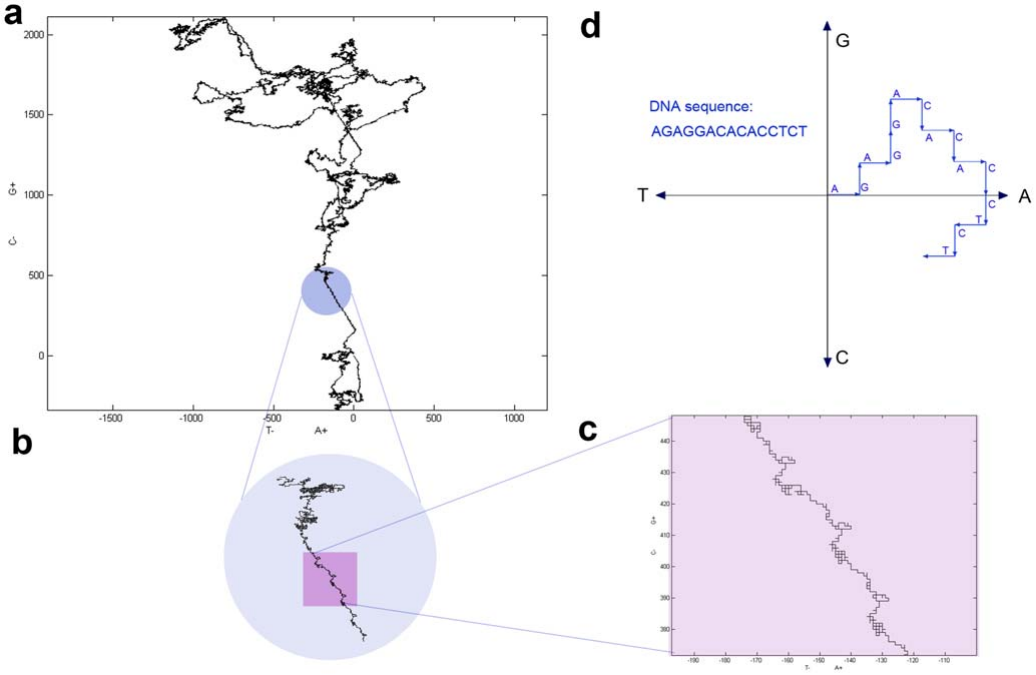
Illustration of the 2D DNA walk method. (a)–an example of the 2D DNA walk of chromosome 1 of *Saccharomyces cerevisiae*; (b) - zoomed in fragment of 2D DNA walk with FLO genes family in the blue circle; (c) - zoomed in fragment of the part of FLO genes in the violet rectangle; (d)–low level representation of the 2D DNA walk method for a short sequence.

**Figure 9:**
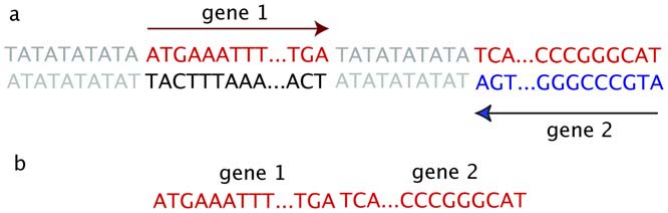
Sequences made up of genes only. Illustration of how genes from different strands are included in to the sequence: (a) piece of DNA with genes and intergenic areas; (b) sequence, made up of genes only.

The 2D DNA walk method works as follows. We map DNA sequence into the square lattice on the plane with G−C and A−T axes, where the origin (0,0) coincides with the first nucleotide in DNA sequence. For every consecutive nucleotide we make a step: for G–one step up, C–one step down, A–one step to the right, T–one step to the left [1]. Thus the original DNA sequence will be mapped as a certain trajectory on a plane with G−C and A−T axes (Figure 8). For mapping and drawing 2D DNA sequences we used homemade scripts, which run under MATLAB software (MathWorks, http://www.mathworks.com). 2D DNA walks of sequences, made up of genes only, are composed of concatenated gene sequences in the same order as they are located on the chromosome, without intergenic areas. Note that when applied to chromosomes, the 2D DNA walk method draws trajectories for only one, usually the “+”, strand. Thus, if a gene is located on the “+”-strand, the corresponding gene-sequence is cut from the “+”-strand, from the start position to the end position. For genes located on the “−”strand, the corresponding sequence is also cut from the “+”-strand, but now from the end position to the start position, because genes on the “−”-strand have the opposite direction (Figure 9). For example, if gene 1 is located on the “+”-strand ATGAAATTT…TGA, and gene 2 is on the “−”-strand ATGCCCGGG…TGA (Figure 9a), then the resulting concatenated sequence, made up of two genes, would be AGTAAATTT…TGATCA…CCCGGGCAT (Figure 9b). GSS transformation consists in merging genes from “+”-strand, without changing their order, with concatenated genes from the “−”-strand, excluding intergenic regions [29]. Sequences for genes from different strands are taken as described above (see also Figure 9).

### Detection of symmetrically correlated areas

Alignment of large and highly diverged DNA sequences is a difficult and still unresolved task, and the standard alignment methods cannot be applied to this type of sequences [4]. Our method of detection of correlated areas is based on two measures: Hausdorf distance [65] and Minimum Volume Enclosing Ellipsoid by Nima Moshtagh (http://www.mathworks.com/matlabcentral/fileexchange/9542).

To define large correlated areas in DNA sequence we use the following assumptions and procedures. For the purpose of identification of large-scale correlations we can ignore local differences in nucleotide sequence. Thus the 2D DNA walk can be approximated (smoothened) by a curvilinear trajectory. The smoothing algorithm is based on the calculation of the “mass center” for a sliding window where the “mass center” is defined as a resulting nucleotide sequence after eliminating complimentary annihilating pairs G−C and A−T. Smoothing can be done on various scales. After trajectory is smoothened, the search of large-scale correlations between two sequences is done by calculation of two measures: the measure of closeness and the measure of complexity of two curvilinear trajectories. The measure of closeness is estimated with the Hausdorf distance d_H_(X,Y). In view of the fact that long parallel regions of the 2D DNA walk trajectories might have high correlations, but they are not the regions of potential duplications and could have emerged as a result of uniform nucleotide composition, we need to introduce an additional criterion for trajectory complexity. Under complexity we understand the curvilinear property of a trajectory, and it is calculated as a minimal square necessary to cover a trajectory by an ellipse. The ellipse size is calculated using the Minimum Volume Enclosing Ellipsoid algorithm. As a characteristic parameter, we take a ratio of minimal to maximum axes of a covering ellipse K(N) where N is the number of a sliding window (Figure S6). Thus we proceed over two trajectories with a sliding window, calculate d_H_(X,Y) and K and plot them together as functions of the sliding window N. The more correlation exists between the areas, the less is the Hausdorf distance and the value of K is close to 1, so that the ellipse will approach the circle. The circle signifies that the trajectory is not a straight line, but has a certain degree of complexity.

Symmetrical correlated regions were tested with protein BLAST [66], significance cutoff of E-4. Cutoff of E-4 was chosen to be able to detect diverged domains and parts of genes as it is based on our experience of assembling orthologous gene families [67]. The absence of hits in BLAST doesn't mean the absence of homology as it was demonstrated in [68], inferring homologous relations of topoisomerase and primase domain with PSI-BLAST iterations. What we see with BLAST is that there are some cases of gene or domain duplication inside the correlated regions, and they could be used as seeds for investigating the surrounding areas by other methods for distant homology identification. In the case of highly divergent DNA sequences even PSI-BLAST might be unable to find homology due to the frameshifts.

### Gene Distribution Over Strands

All statistical analyses were performed with the R software package (www.r-project.org).

Information about the number of genes on the “+” and on the “−”-strand and gene boundaries was taken from gene location tables (.ptt files) for bacteria, archaea and fungi, and from seq_contig.md file for humans.

Graphics of gene distribution over strands in the whole chromosome (Figure 6) were constructed as follows. The entire chromosome sequence was divided in a non-intersecting way into the pieces of a defined window size (here with the sizes of 25 kB, 50 kB and 100 kB), and for each window we calculated the number of genes on the “+”-strand (Nu+) and the number of genes on the “−”-strand (Nu−). The results were depicted as logarithms of gene number ratio: Log(Nu+/Nu−) so that positive values would reveal regions where the number of genes on “+”-strand exceeds that on “−”-strand, and negative values would reveal those with the opposite ratio.

By a cluster of genes continuously located on one strand we understand the region in which genes are located only on one strand (either “+” or “−)” and the closest gene from the either side will be a gene from the opposite strand. The length of the cluster is measured as the number of genes in this cluster. Frequency distributions of the lengths of such clusters were constructed for clusters from both strands in Figure 7 and for each strand separately in Figure S3. Exponential fitting was done by the method of least squares.

Positions of Ori sites for *Sulfolobus solfataricus* were taken from experimental data [69]. Locations of ARS sequences for *Saccharomyces cerevisiae* were taken from OriDB [45]. Data on location of bidirectional promoters in *Saccharomyces cerevisiae* were taken from [36].

## Supporting Information

Figure S1 Examples of chromosome 2D DNA walks before and after GSS transformation. Trajectories of the genes in the original order are shown on the left, GSS transformed trajectories are on the right. The figure occupies the next 16 pages. Presented are 12 chromosomes of bacteria, 4 chromosomes of archaea, 20 chromosomes of fungi and 24 chromosomes of humans.
(5.28 MB PDF)

Figure S2 Superposition of bidirectional promoter density with gene distribution over strands for Saccharomyces cerevisiae, chromosome 12.
(0.15 MB PDF)

Figure S3 Normalized frequency of clusters with genes continuously located on one strand calculated for (a) bacteria Bacillus anthracis Ames, (b) archaea Sulfalobus solfataricus, (c) fungi Saccharomyces cerevisiae, chromosome 12, (d) Homo sapiens, chromosome 8.
(0.24 MB PDF)

Figure S4 Statistics for different replichores in bacteria. Relation between (a) number (Pearson coefficient r = 0.98), (b) cumulative gene length (r = 0.92), (c) cumulative GC (r = 0.97) and (d) AT (r = 0.97) skews of genes from different strands in different replichores in 524 bacteria.
(1.13 MB PDF)

Figure S5 2D DNA walks with the marked sites of replication origins for archaeon Sulfolobus solfataricus and Saccharomyces cerevisiae.
(1.56 MB PDF)

Figure S6 Detection of symmetrically correlated areas. (a) Plot of the Hausdorf distance dH(X,Y) and K-value (from Minimum Value Enclosed Ellipsoid method) against the sliding window N. (b) Detected correlated areas on the 2D DNA walk trajectory.
(0.85 MB PDF)

Table S1 Total number, total length and cumulative GC and AT skews of genes located on different strands for 671 chromosomes: 524 of bacteria, 36 of archaea, 87 of fungi and 24 of humans.
(0.16 MB PDF)

Table S2 BLAST hits for symmetrically correlated regions. BLAST hits (cutoff E-4) for symmetrically correlated regions of Saccharomyces cerevisiae, chromosomes 1 and 16 and Encephalitozoon cuniculi, chromosome X.
(0.11 MB XLS)

Table S3 Total number, total length and cumulative nucleotide skews of genes located on leading/lagging strand in two chromosome halves (approximate replichores) in 524 bacteria.
(0.20 MB PDF)
