# Supplementary material for: Hidden Chromosome Symmetry: In Silico Transformation Reveals Symmetry in 2D DNA Walk Trajectories of 671 Chromosomes
Source: PLoS One. 2009 Jul 28;4(7):e6396. doi: 10.1371/journal.pone.0006396 (PMC2712679; doi:10.1371/journal.pone.0006396)

Supplementary Figure 1. Examples of chromosome 2D DNA walks before and after GSS transformation. Trajectories of the genes in the original order are shown on the left, GSS transformed trajectories are on the right. The figure occupies the next 16 pages. Presented are 12 chromosomes of bacteria, 4 chromosomes of archaea, 20 chromosomes of fungi and 24 chromosomes of humans.

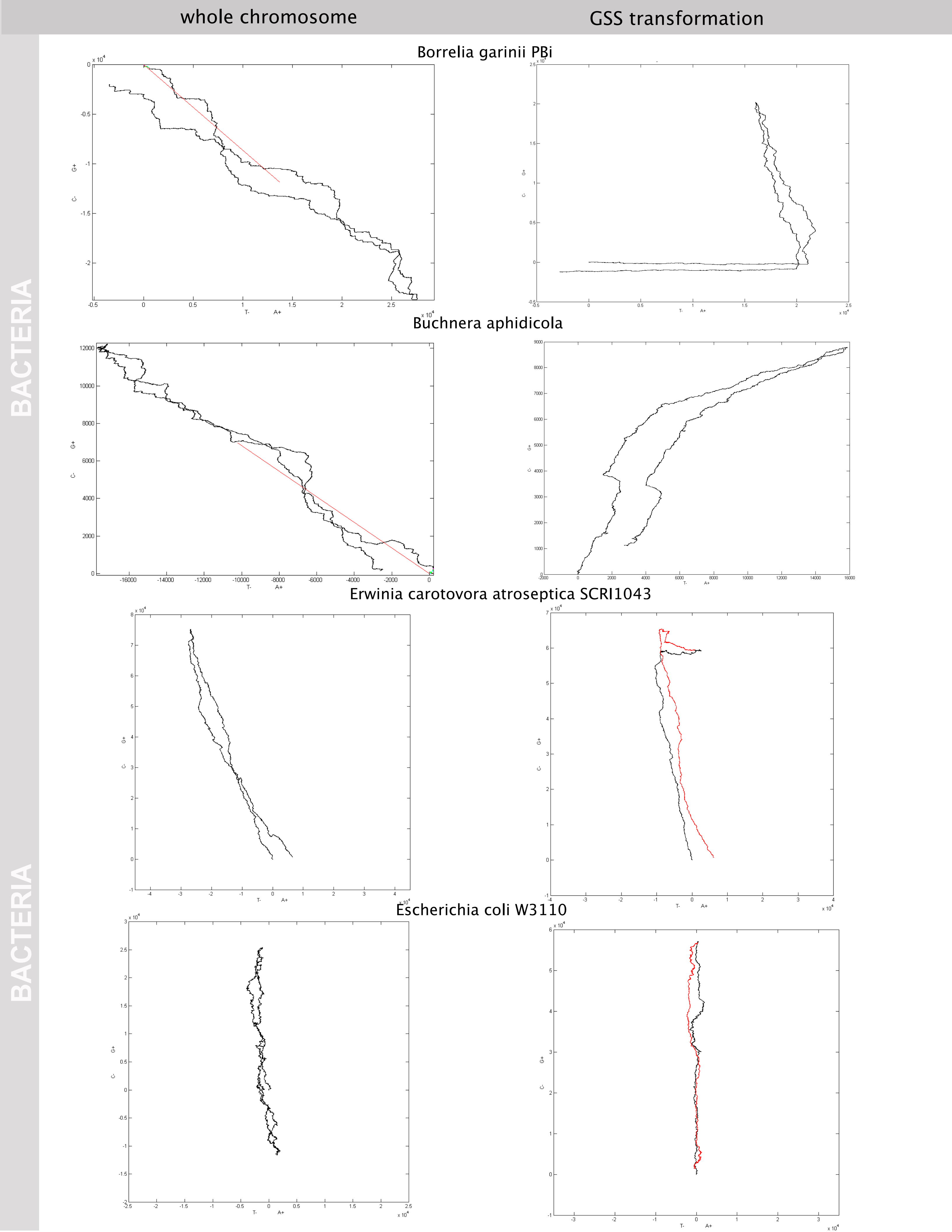

whole chromosome
GSS transformation
BACTERIA
Borrelia garinii PBi
Buchnera aphidicola
BACTERIA
Erwinia carotovora atroseptica SCRI1043
Escherichia coli W3110
T- A+
C- G+

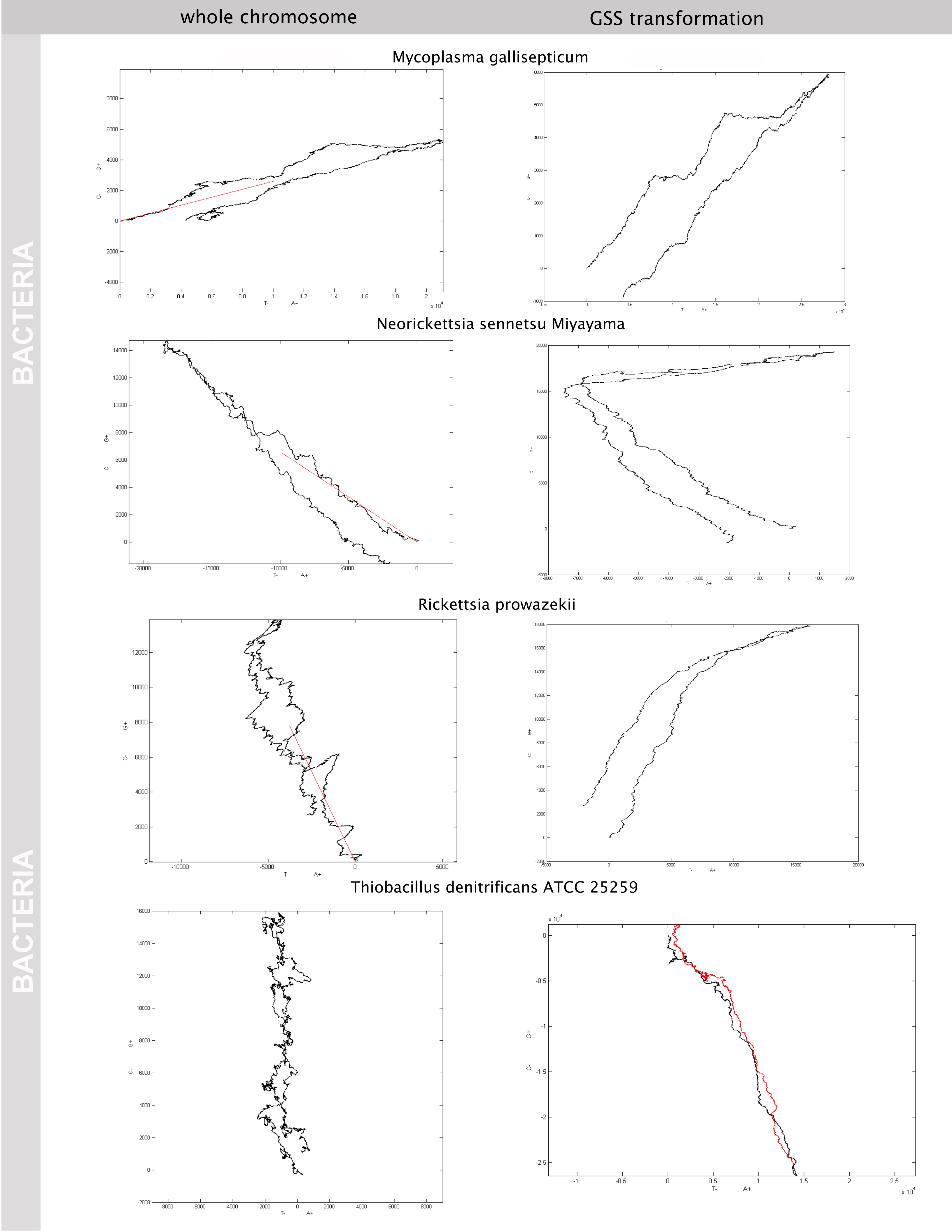

whole chromosome
GSS transformation
BACTERIA
BACTERIA
Mycoplasma gallisepticum
Neorickettsia sennetsu Miyayama
Rickettsia prowazekii
Thiobacillus denitrificans ATCC 25259
T- A+
C- G+

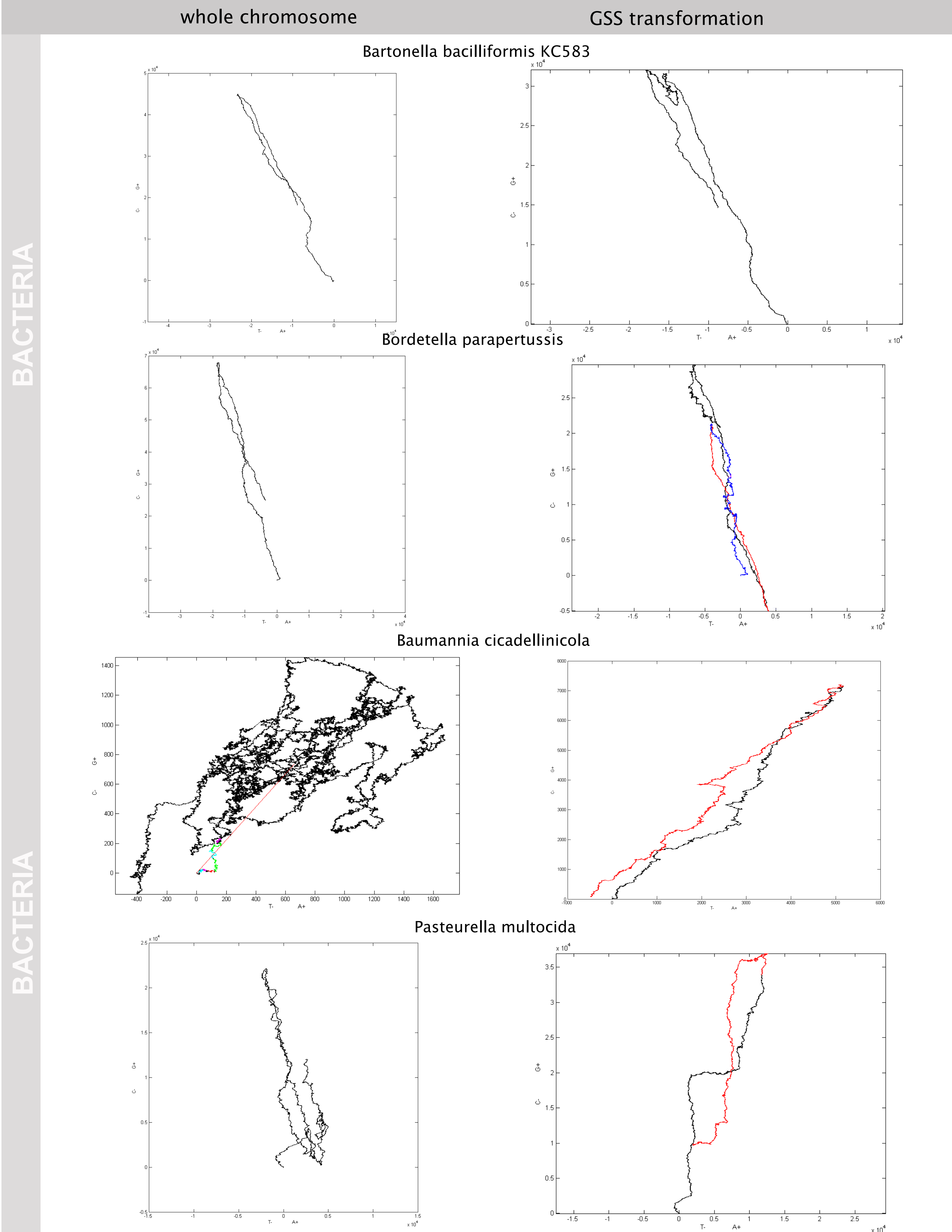
whole chromosome
GSS transformation
BACTERIA
Bartonella bacilliformis KC583
Bordetella parapertussis
Baumannia cicadellinicola
Pasteurella multocida
T- A+
C- G+

whole chromosome	GSS transformation

ARCHAEA

Archaeoglobus fulgidus

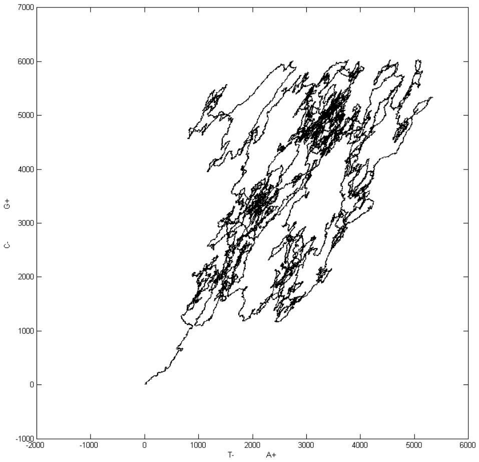

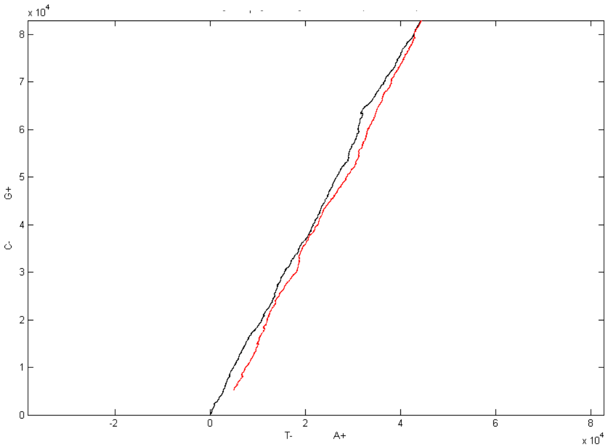

Methanopyrus kandleri

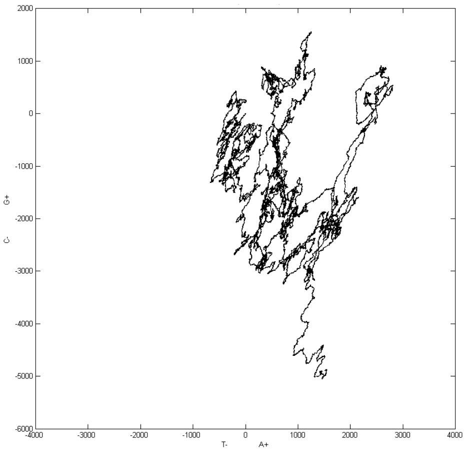

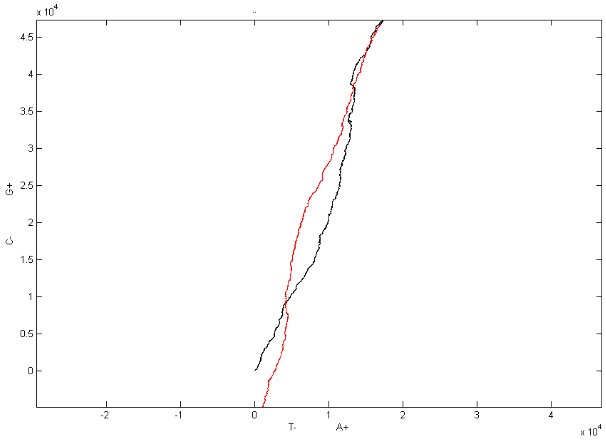

ARCHAEA

Methanosarcina acetivorans

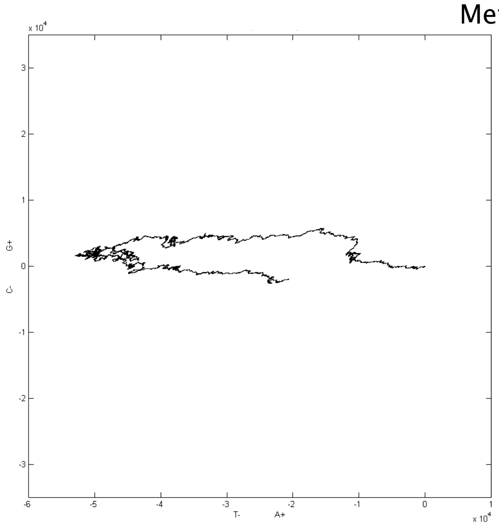

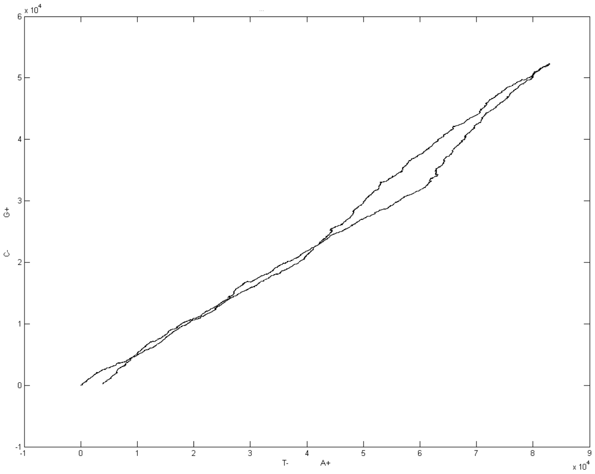

Nanoarchaeum equitans

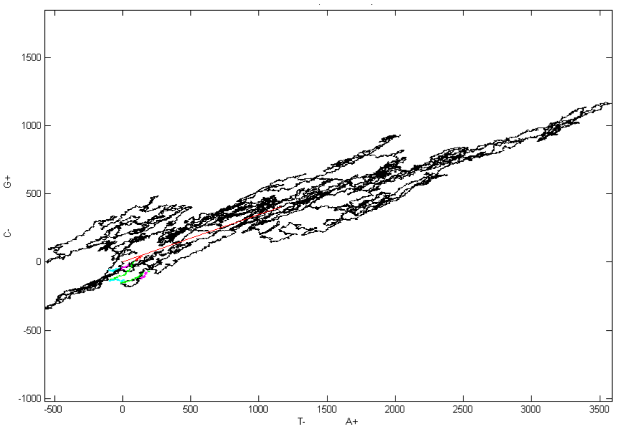

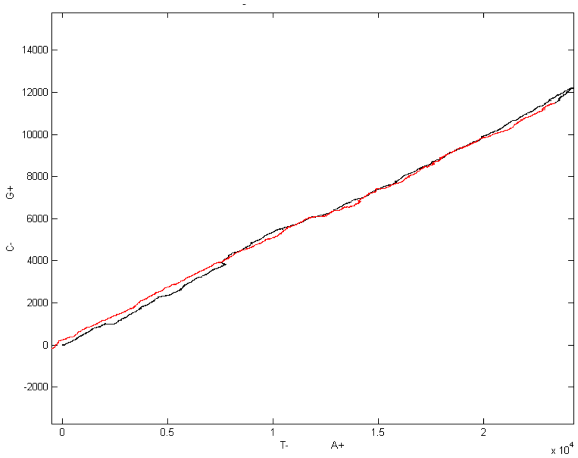

Genes only	GSS transformation

FUNGI

Saccharomyces cerevisiae, chromosome 1

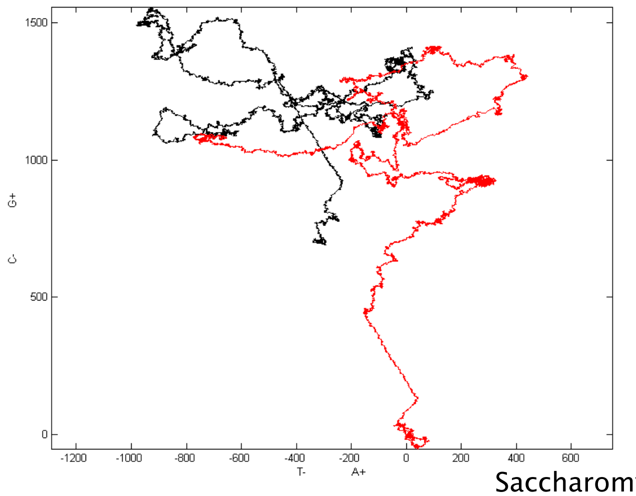

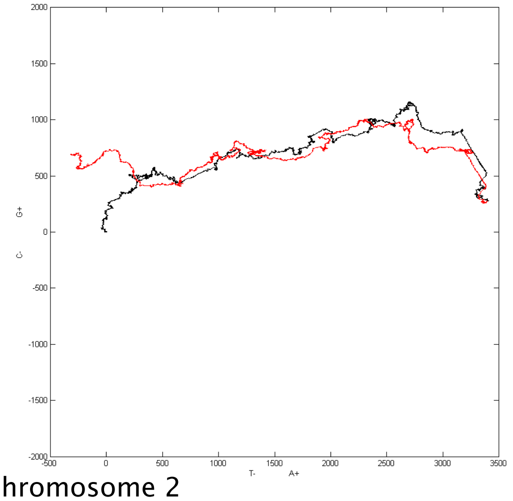

Saccharomyces cerevisiae, chromosome 2

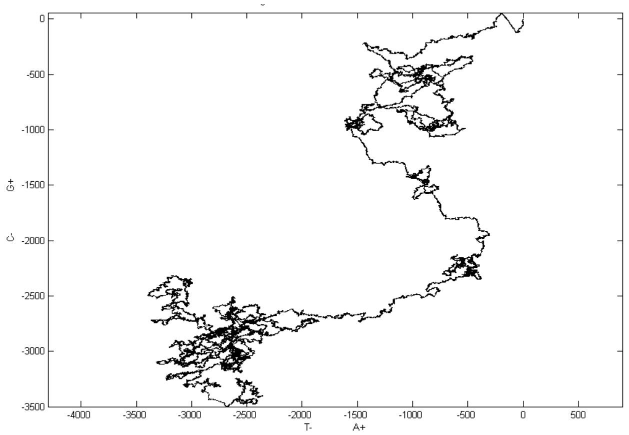

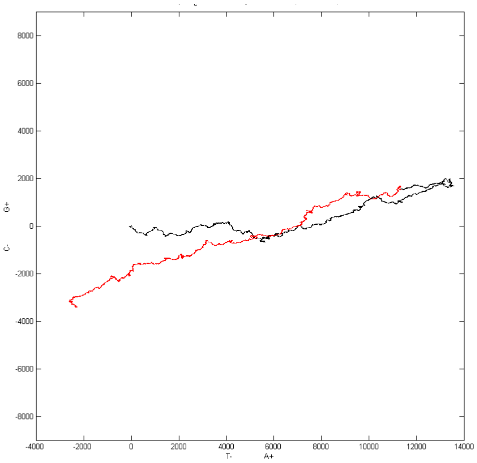

Saccharomyces cerevisiae, chromosome 3

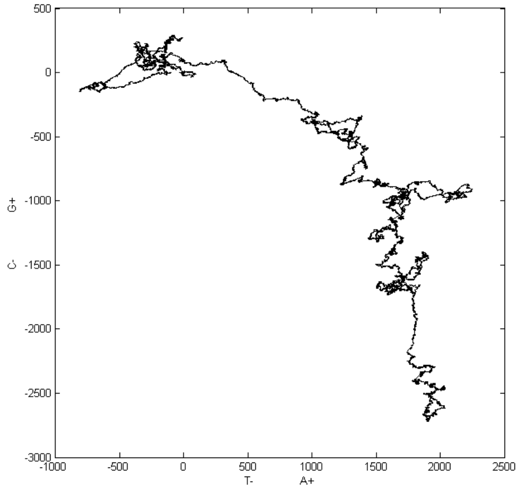

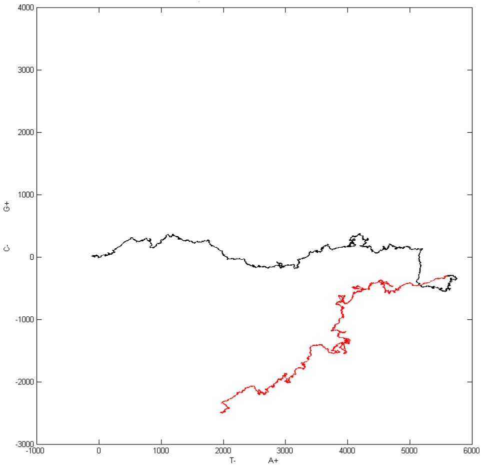

FUNGI

Saccharomyces cerevisiae, chromosome 4

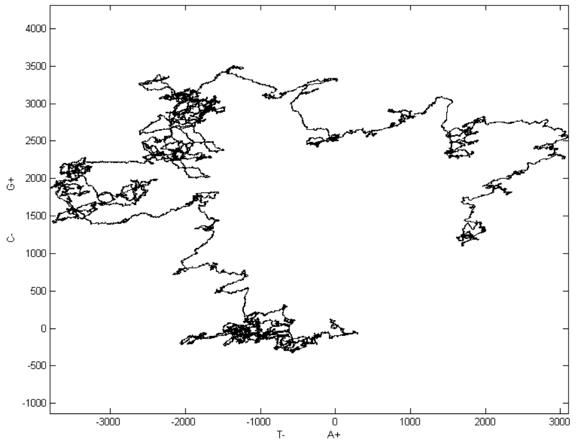

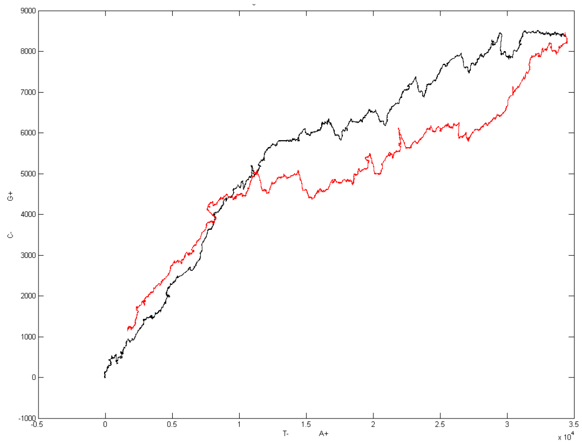

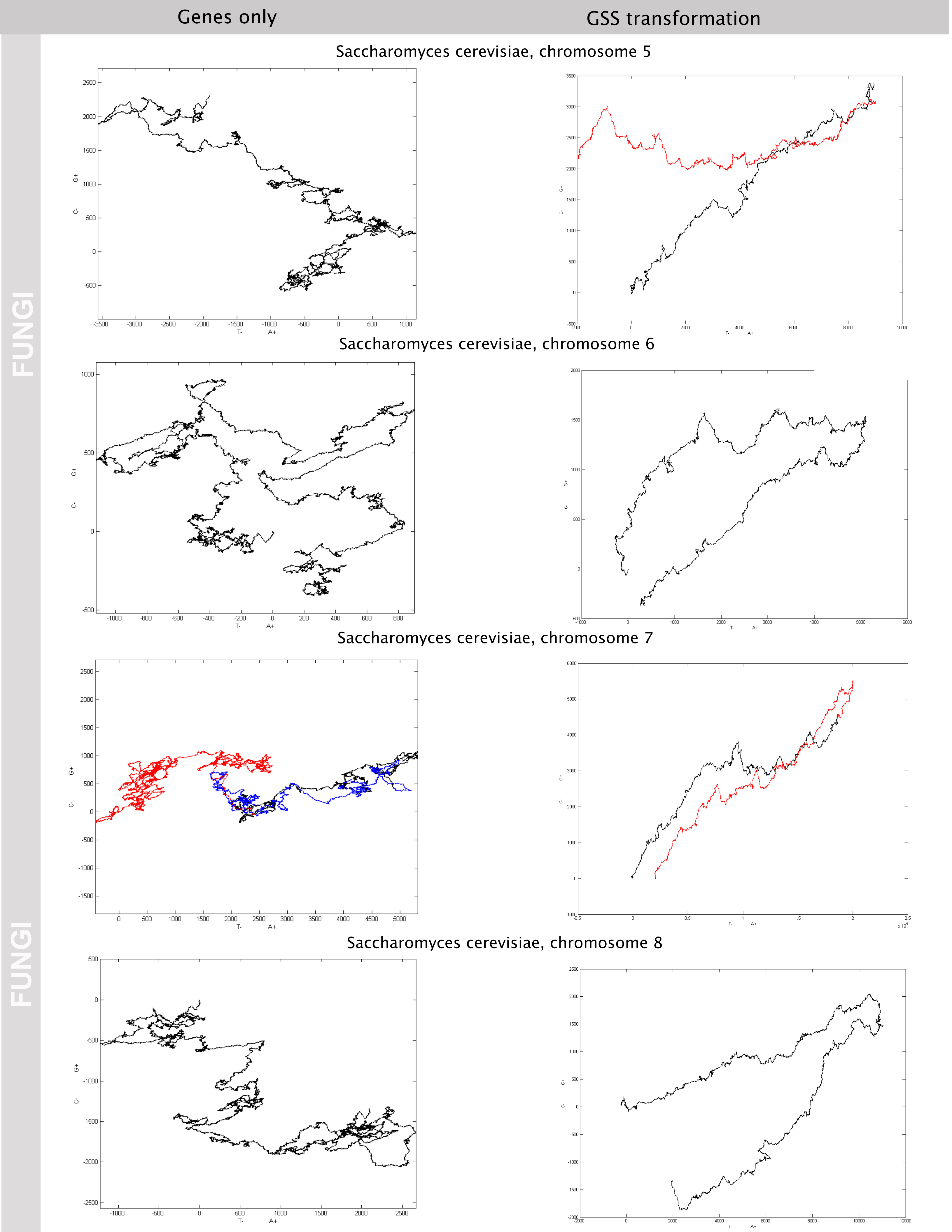
Genes only
GSS transformation
Saccharomyces cerevisiae, chromosome 5
Saccharomyces cerevisiae, chromosome 6
Saccharomyces cerevisiae, chromosome 7
Saccharomyces cerevisiae, chromosome 8
FUNGI
FUNGI

Genes only	GSS transformation

FUNGI

Saccharomyces cerevisiae, chromosome 9

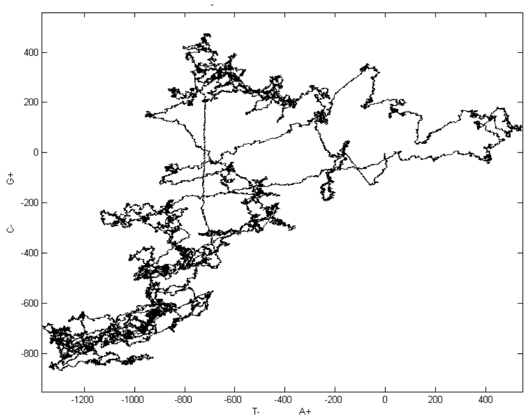

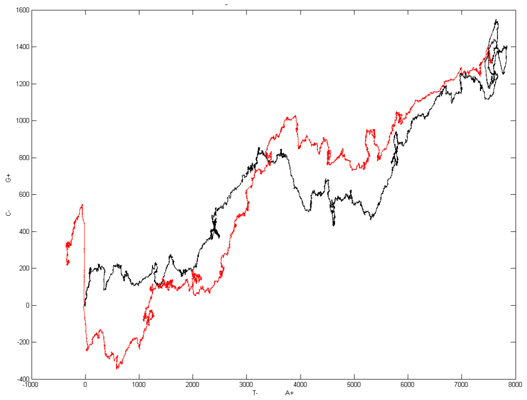

Saccharomyces cerevisiae, chromosome 10

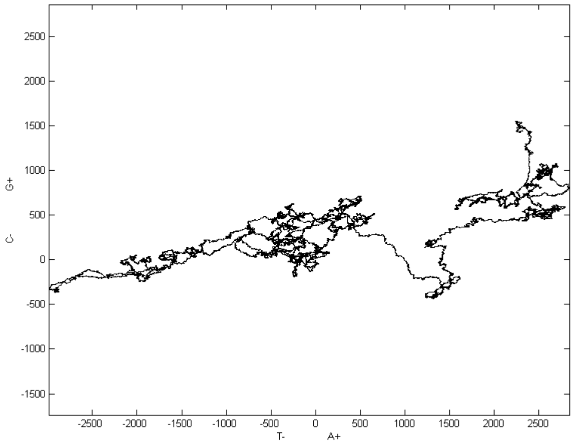

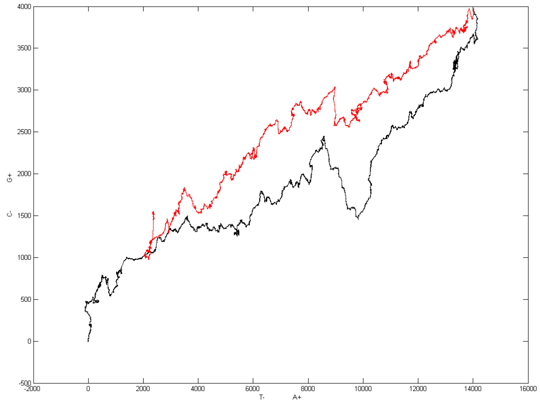

Saccharomyces cerevisiae, chromosome 11

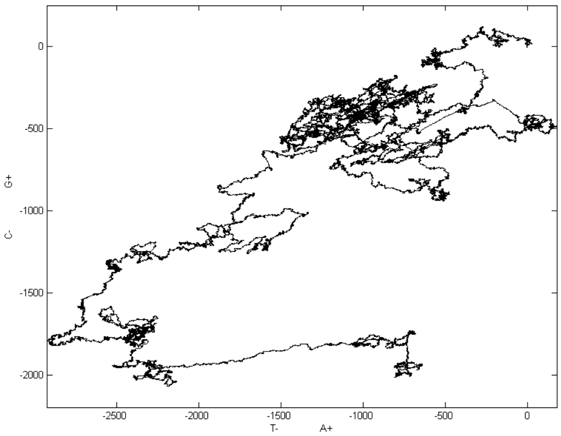

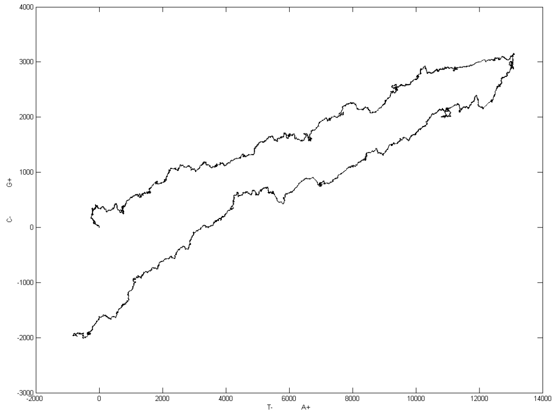

FUNGI

Saccharomyces cerevisiae, chromosome 12

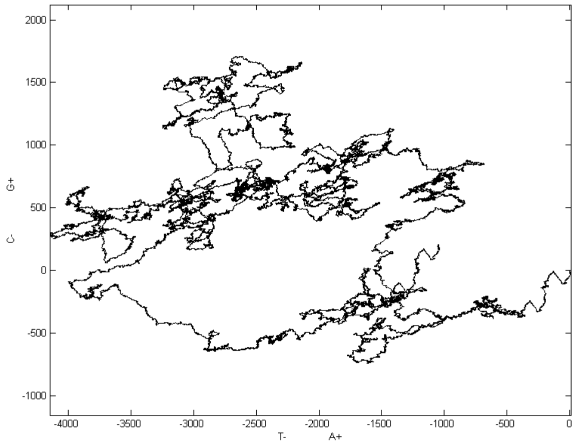

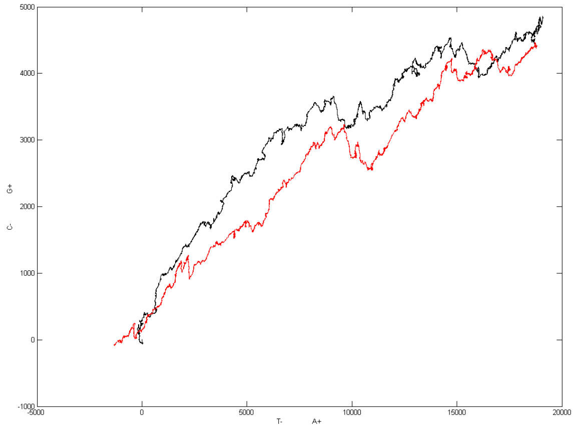

Genes only	GSS transformation

FUNGI

Saccharomyces cerevisiae, chromosome 13

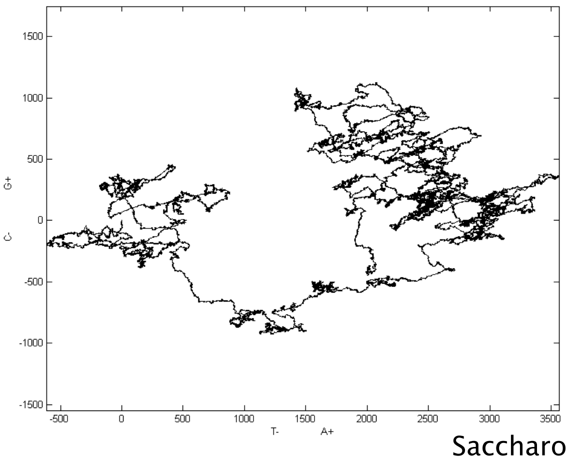

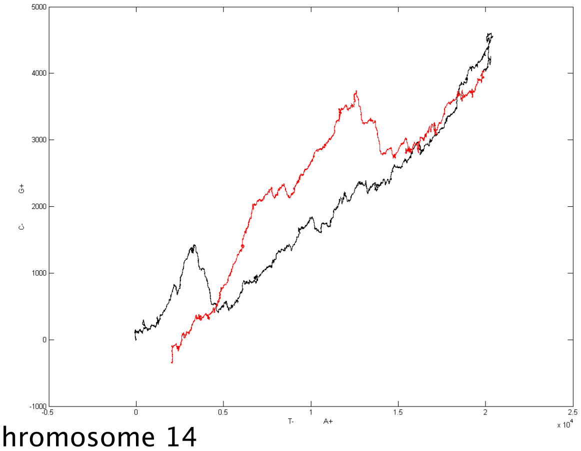

Saccharomyces cerevisiae, chromosome 14

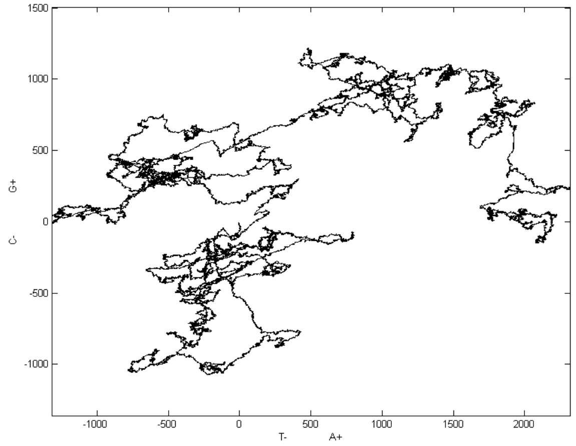

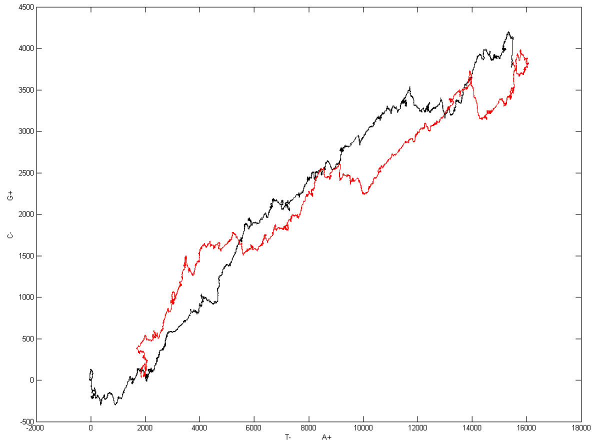

FUNGI

Saccharomyces cerevisiae, chromosome 15

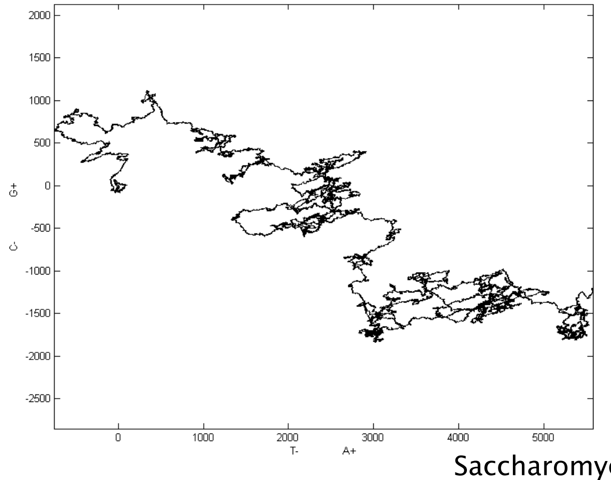

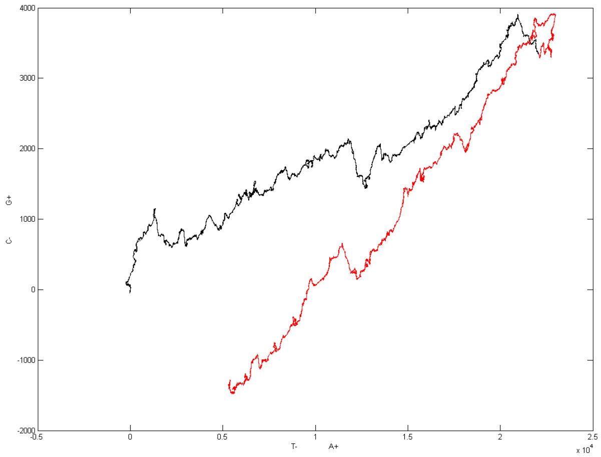

Saccharomyces cerevisiae, chromosome 16

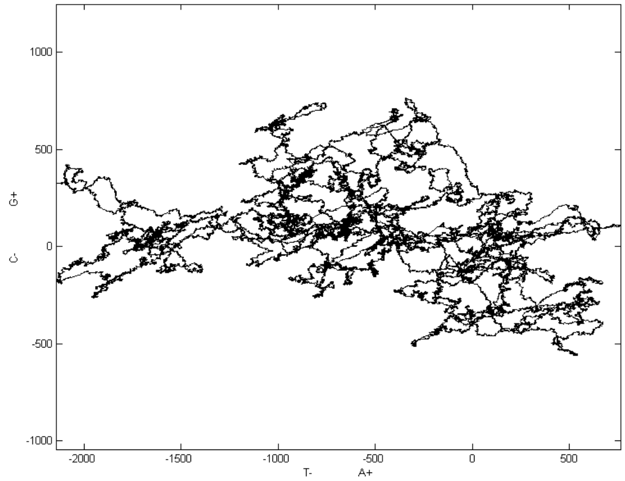

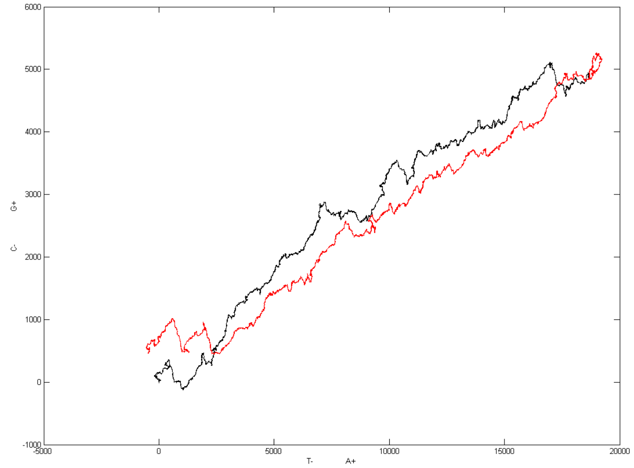

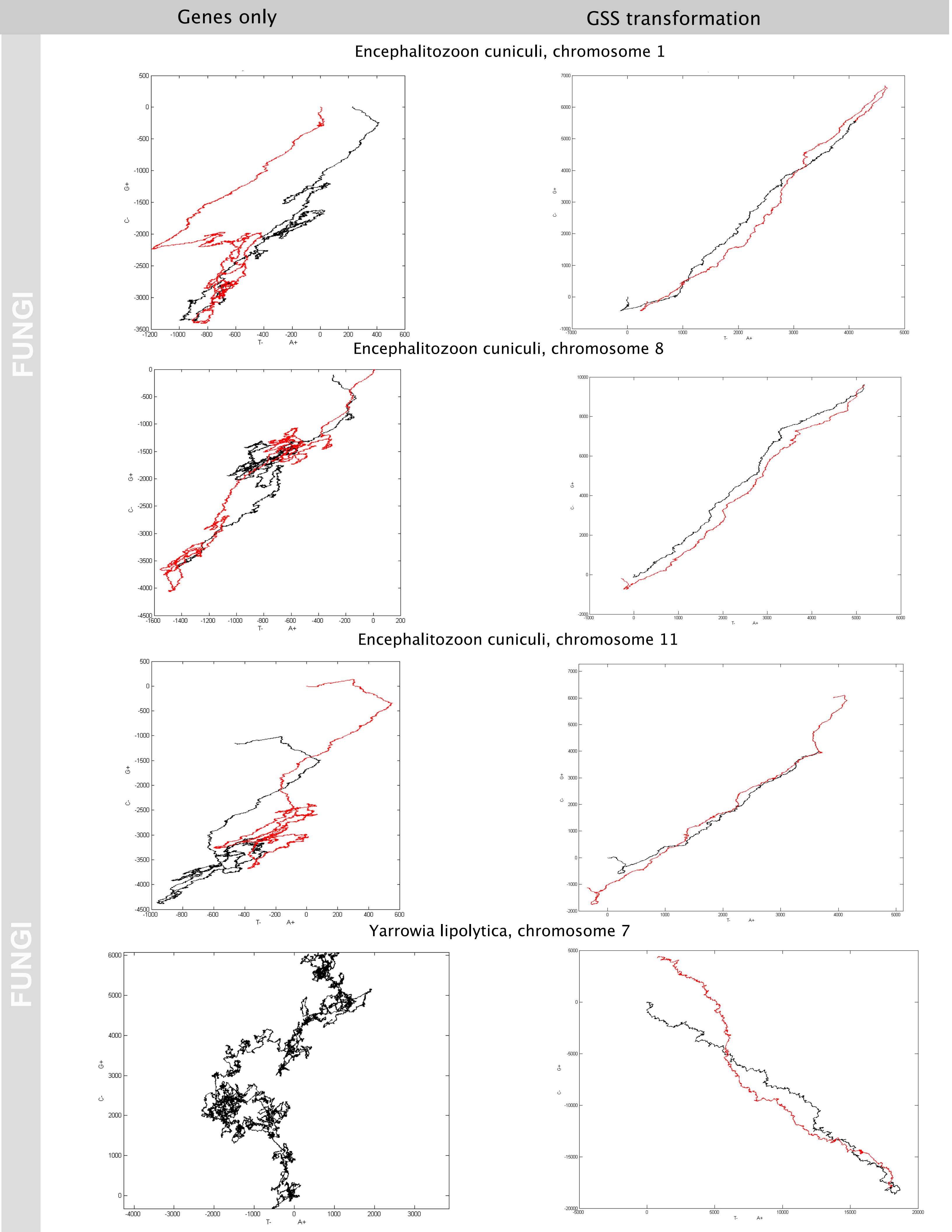
Genes only
GSS transformation
FUNGI
Encephalitozoon cuniculi, chromosome 1
Encephalitozoon cuniculi, chromosome 8
Encephalitozoon cuniculi, chromosome 11
FUNGI
Yarrowia lipolytica, chromosome 7
T-
A+
C-
G+

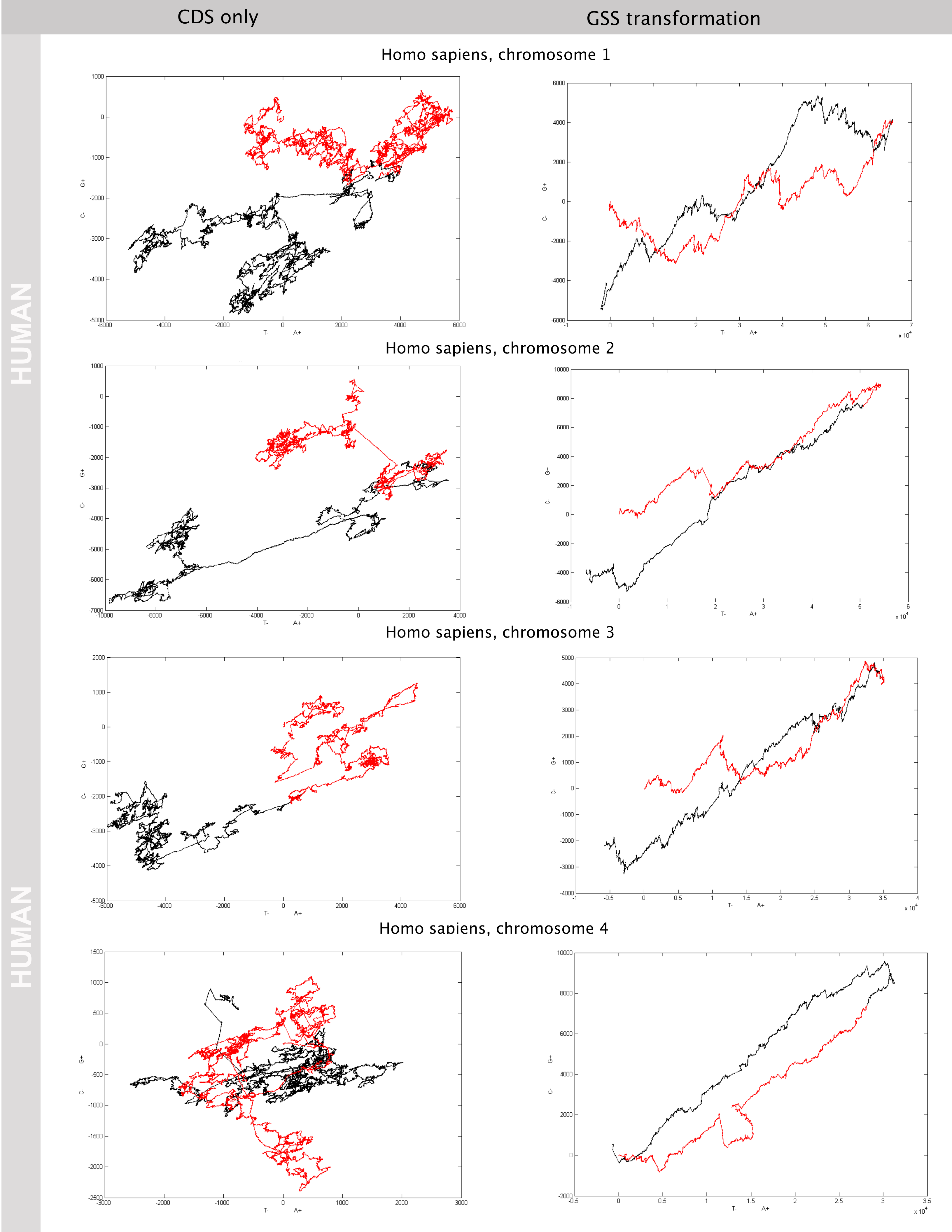

CDS only
GSS transformation
Homo sapiens, chromosome 1
Homo sapiens, chromosome 2
Homo sapiens, chromosome 3
Homo sapiens, chromosome 4
HUMAN
HUMAN
T- A+
C- G+
x 10^4

CDS only	GSS transformation

HUMAN

Homo sapiens, chromosome 5

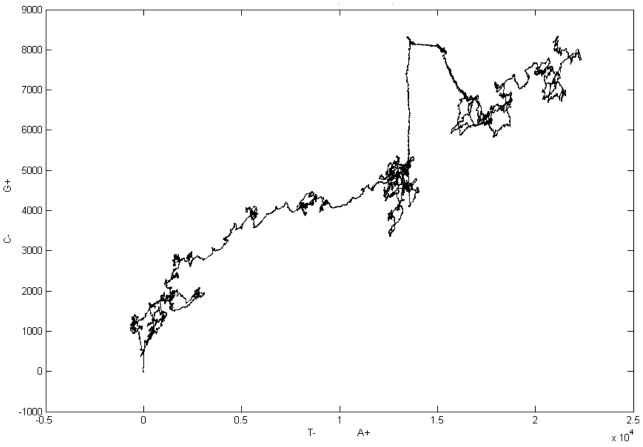

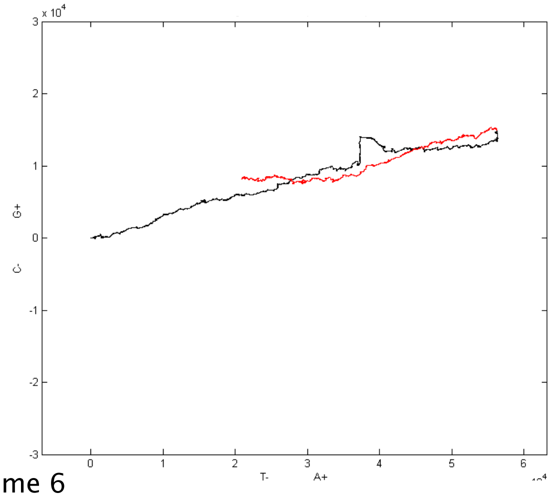

Homo sapiens, chromosome 6

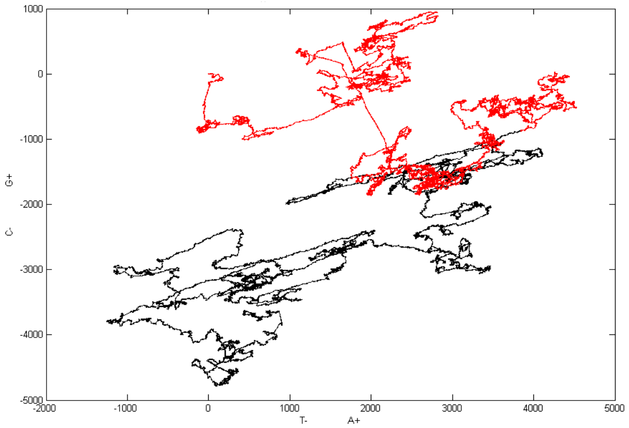

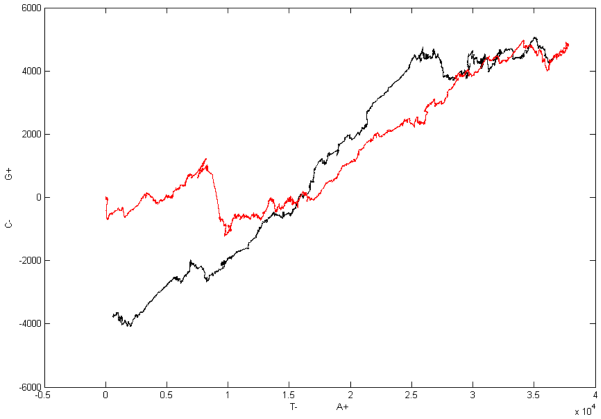

Homo sapiens, chromosome 7

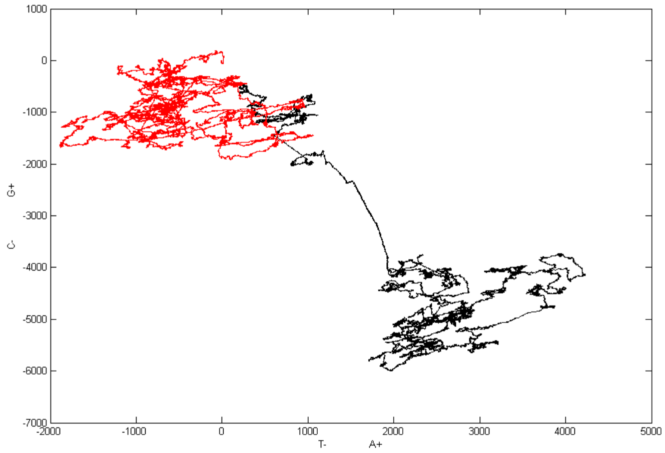

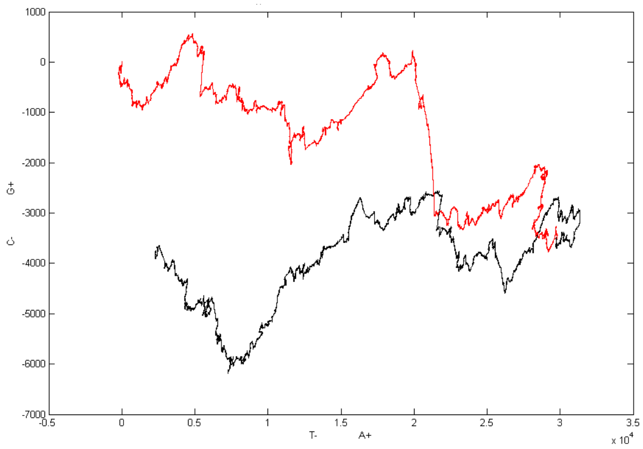

HUMAN

Homo sapiens, chromosome 8

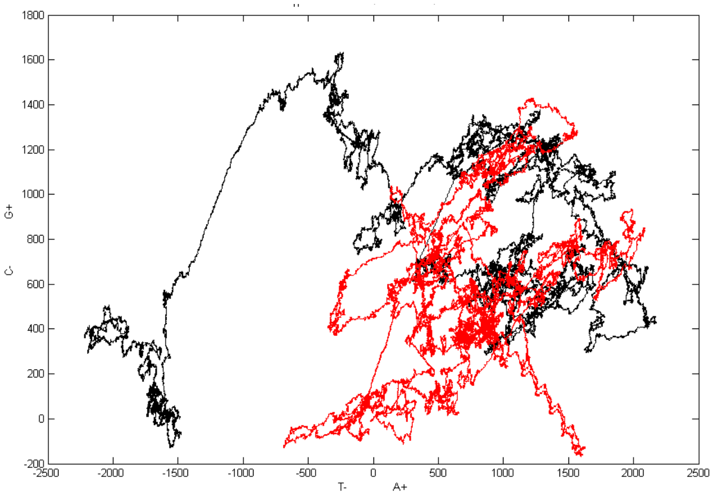

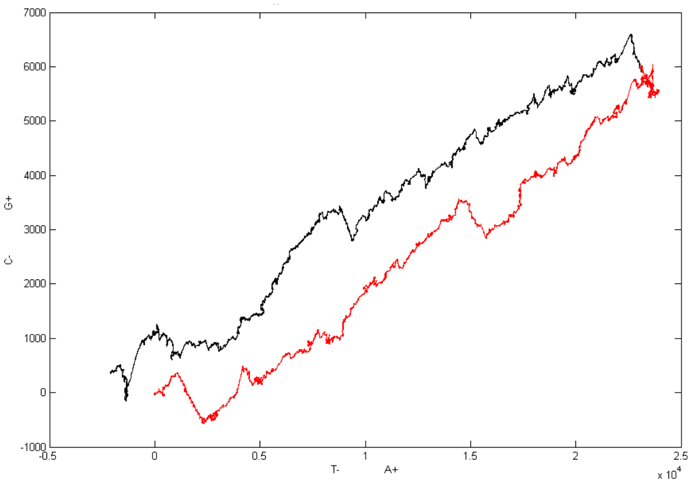

CDS only	GSS transformation

HUMAN

Homo sapiens, chromosome 9

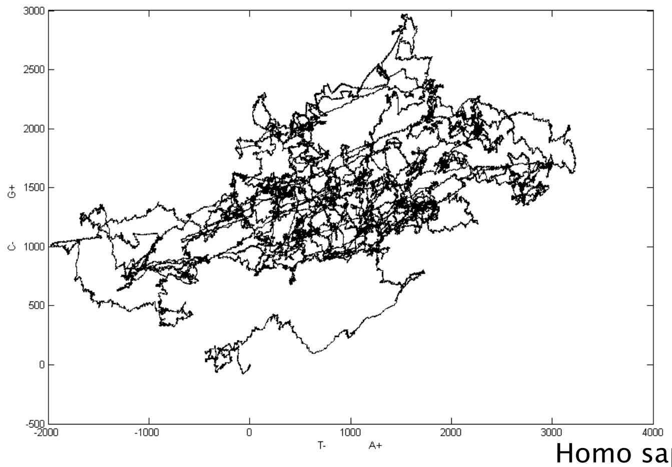

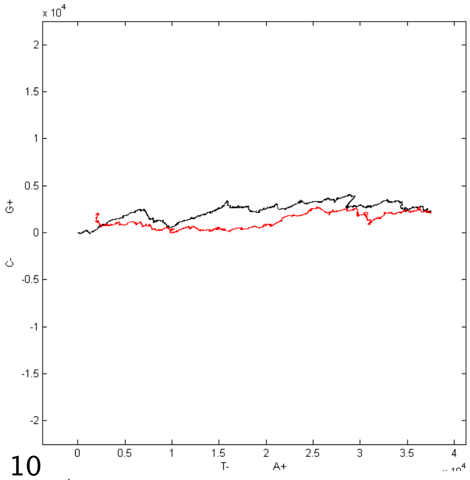

Homo sapiens, chromosome 10

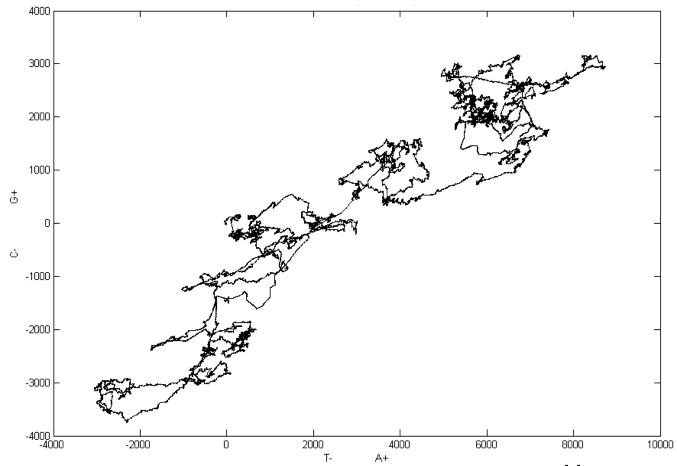

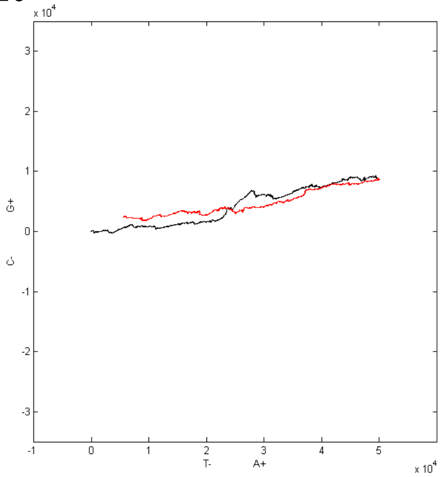

Homo sapiens, chromosome 11

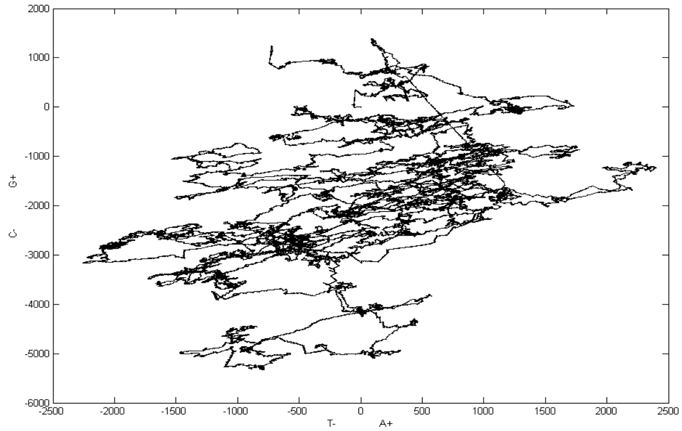

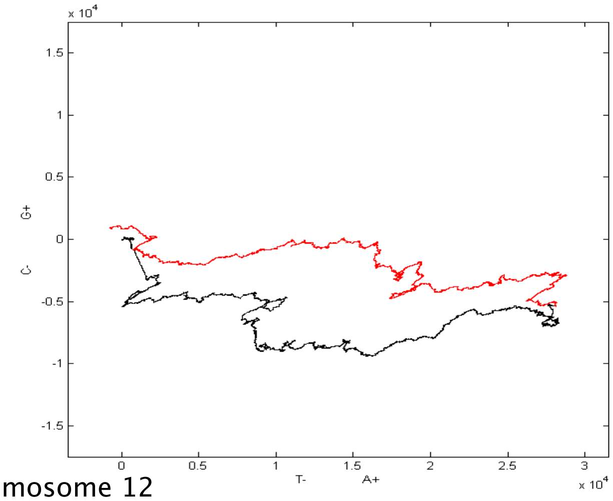

HUMAN

Homo sapiens, chromosome 12

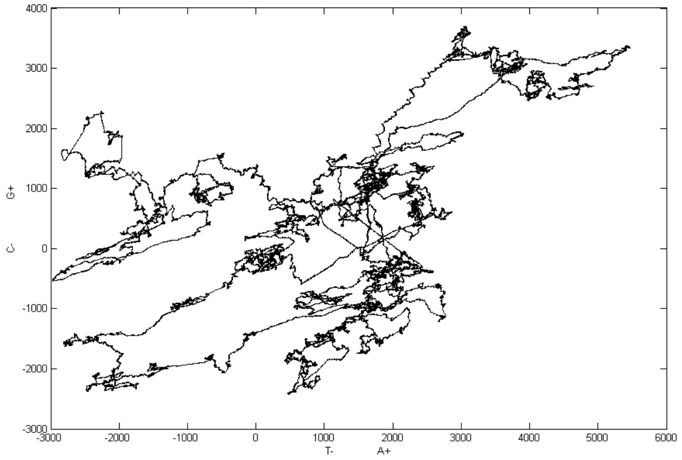

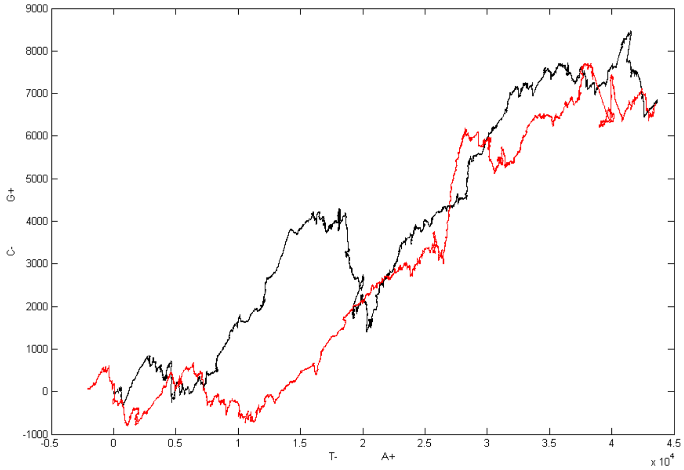

CDS only	GSS transformation

HUMAN

Homo sapiens, chromosome 13

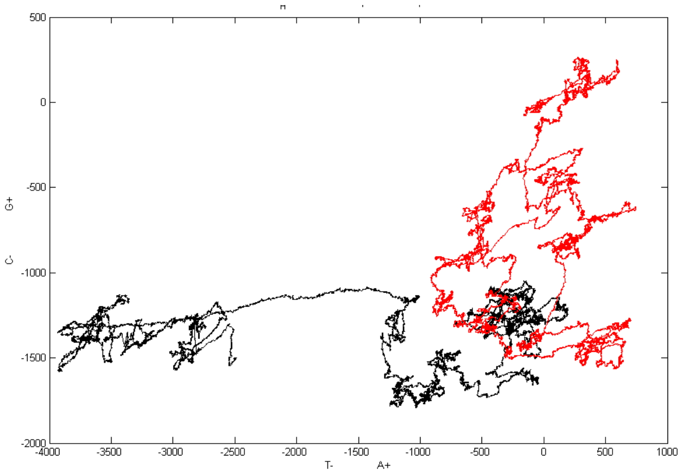

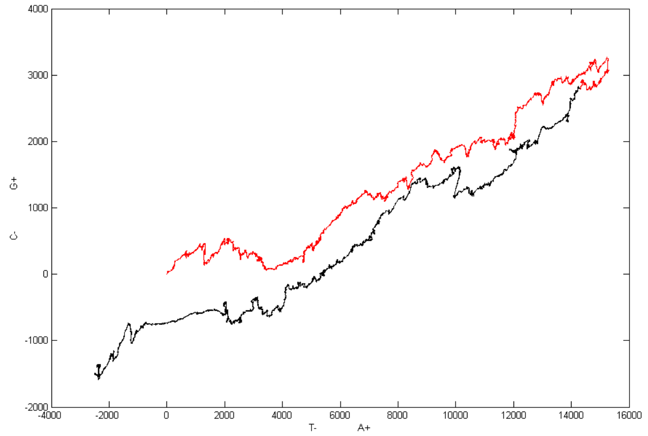

Homo sapiens, chromosome 14

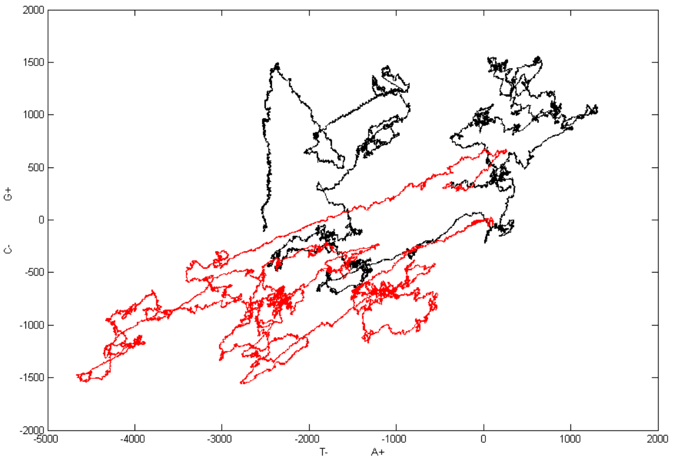

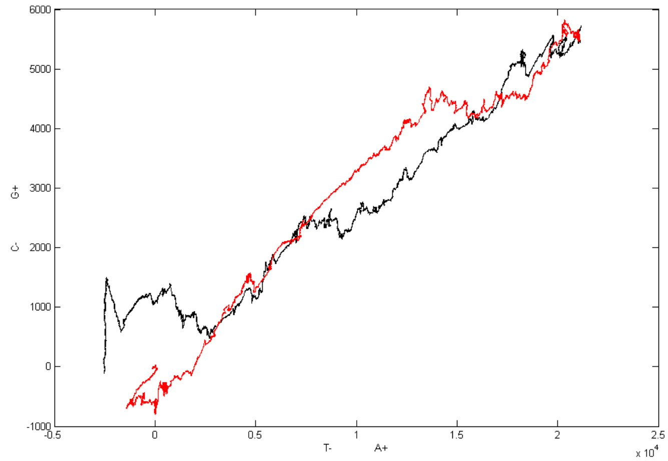

HUMAN

Homo sapiens, chromosome 15

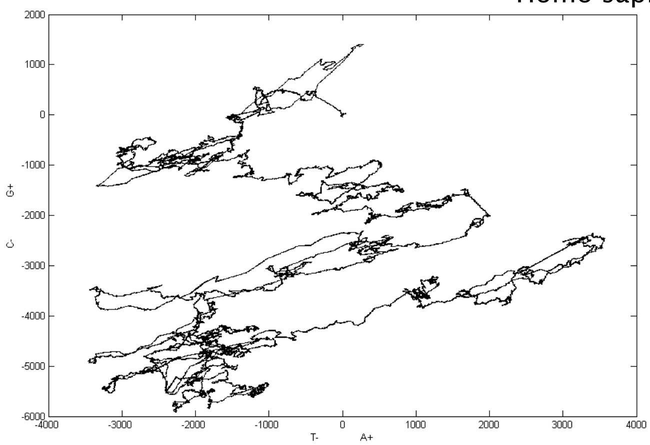

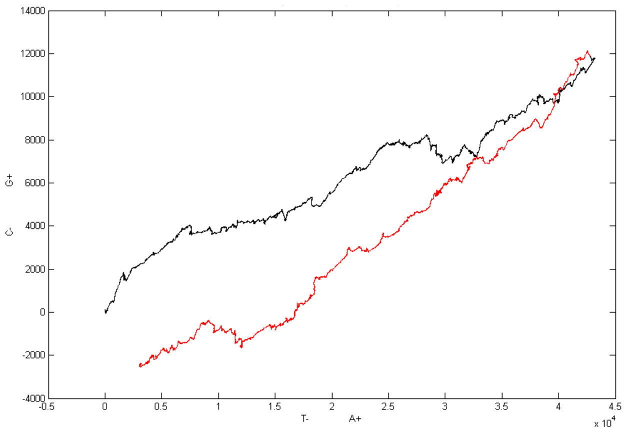

Homo sapiens, chromosome 16

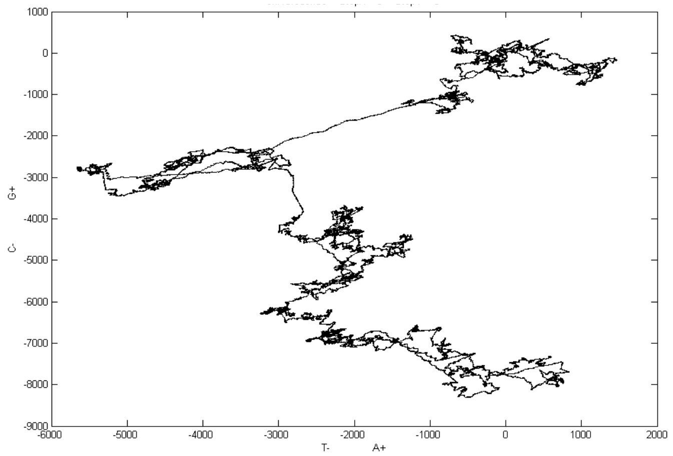

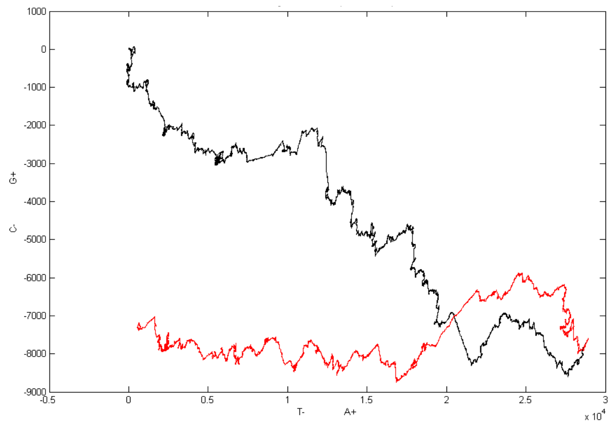

CDS only	GSS transformation

HUMAN

Homo sapiens, chromosome 17

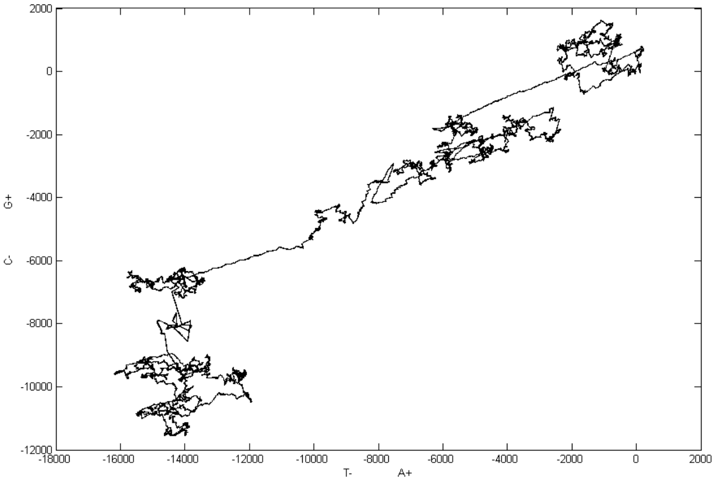

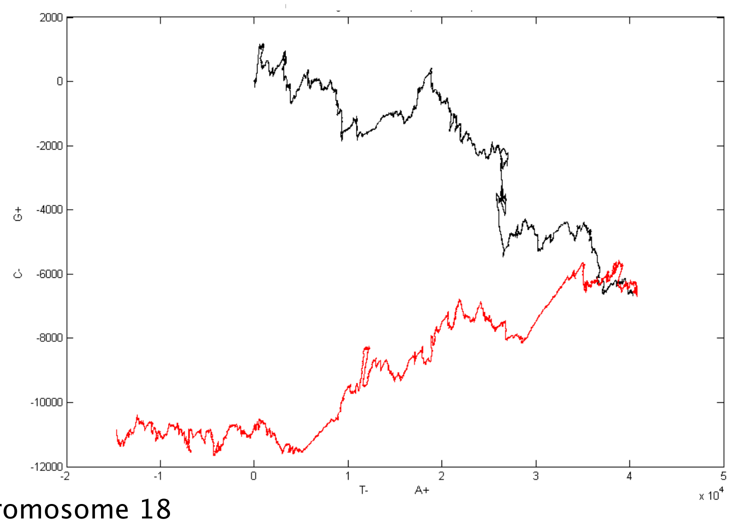

Homo sapiens, chromosome 18

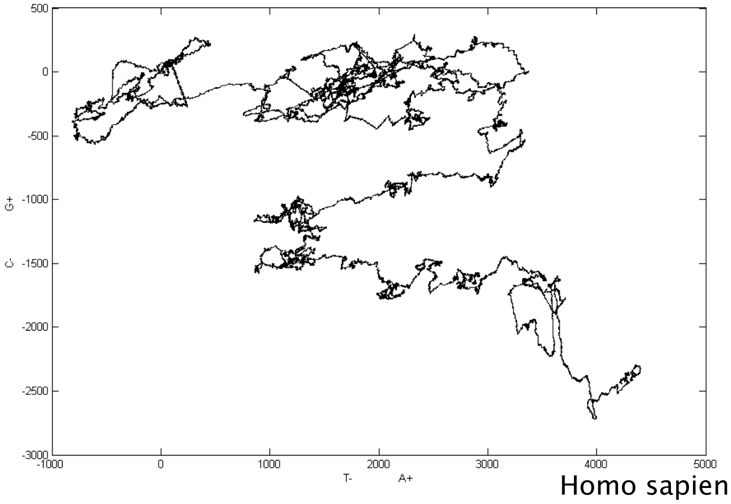

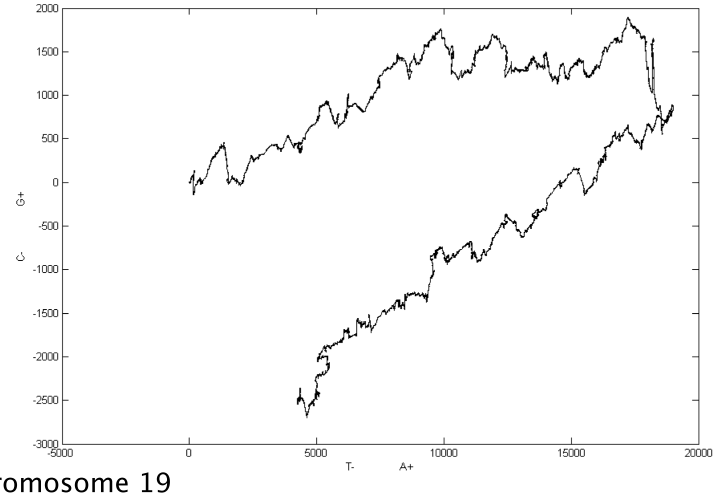

HUMAN

Homo sapiens, chromosome 19

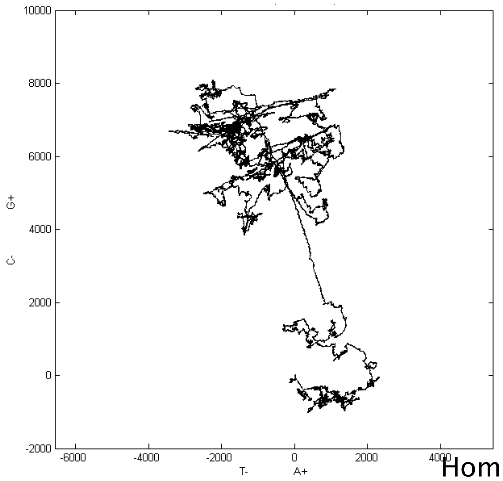

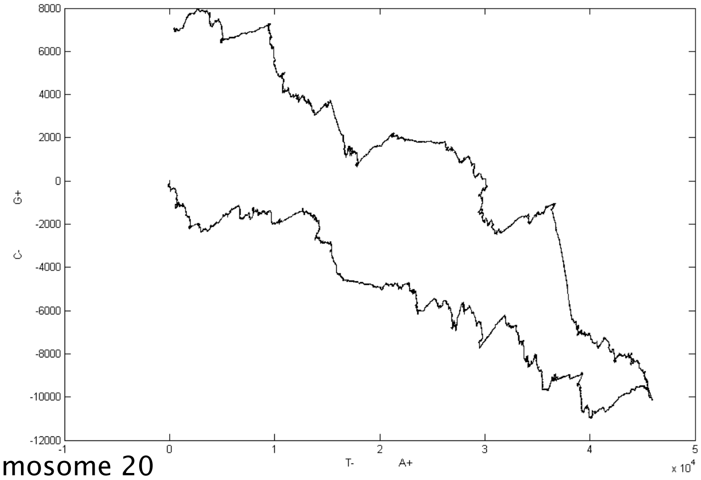

Homo sapiens, chromosome 20

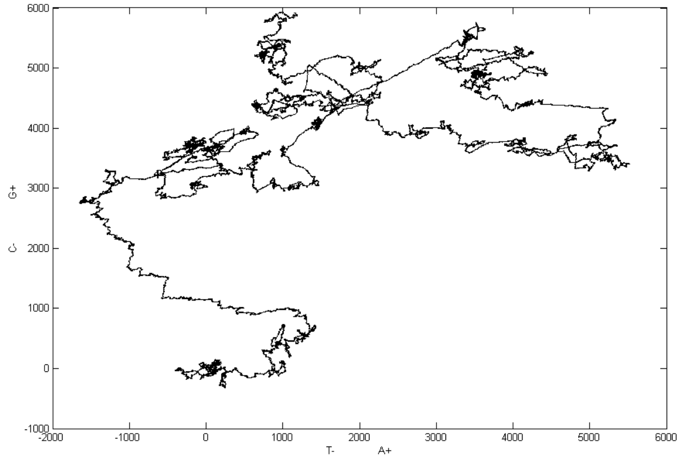

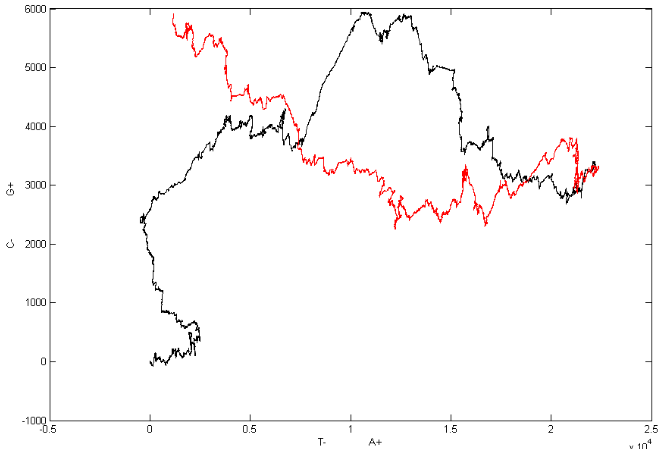

CDS only	GSS transformation

HUMAN

Homo sapiens, chromosome 21

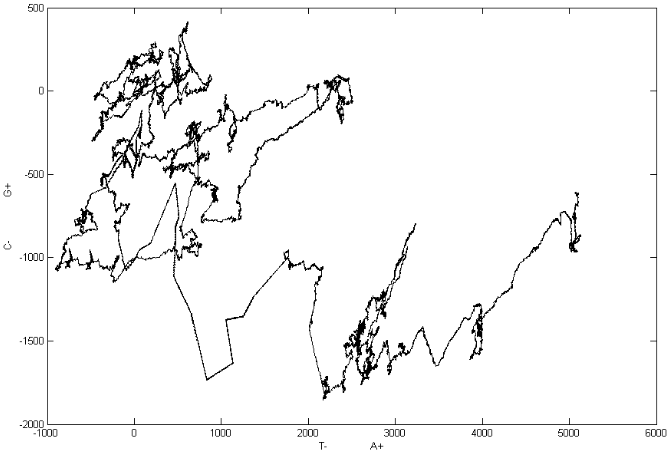

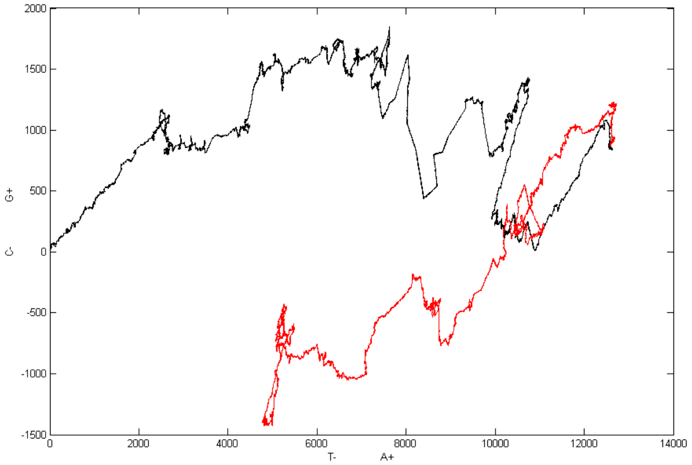

Homo sapiens, chromosome 22

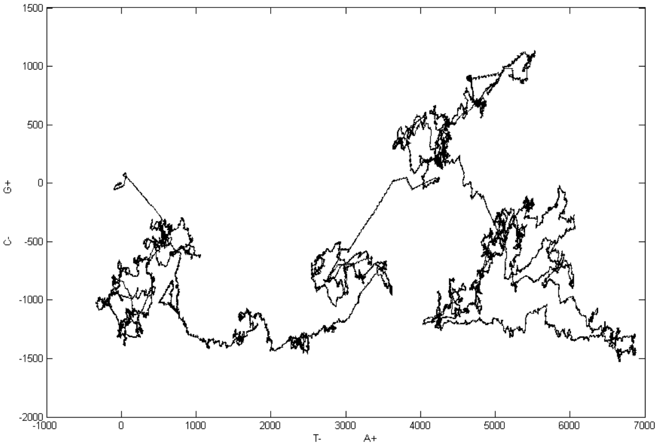

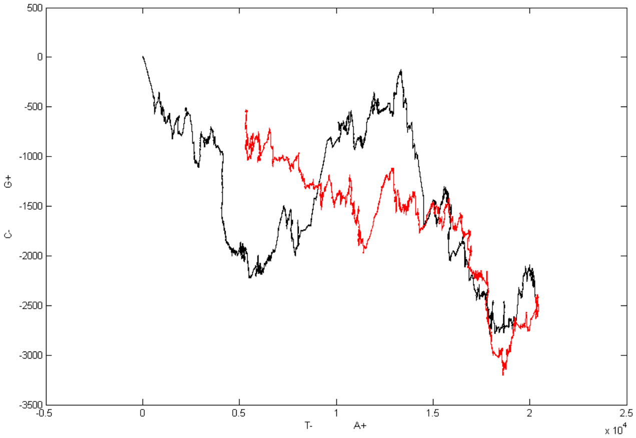

HUMAN

Homo sapiens, chromosome X

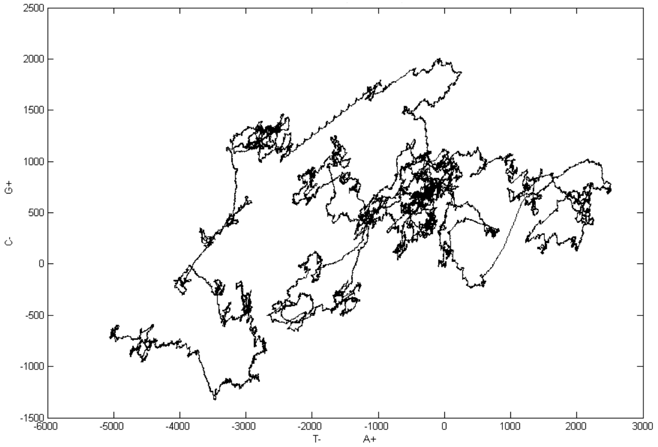

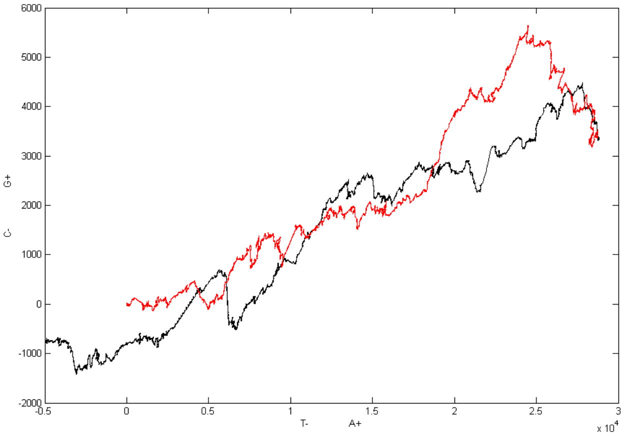

Homo sapiens, chromosome Y

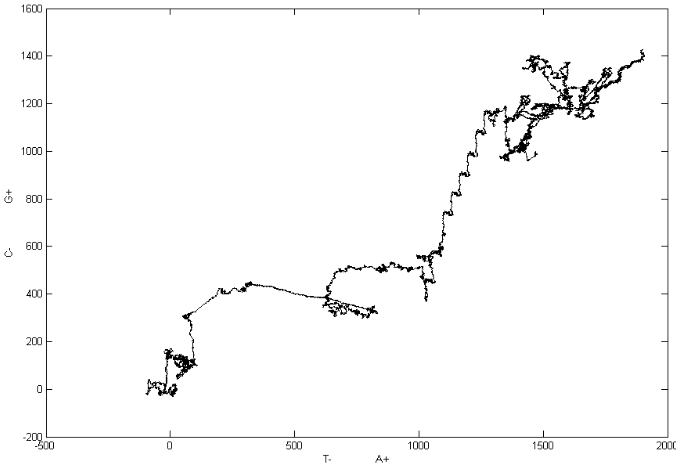

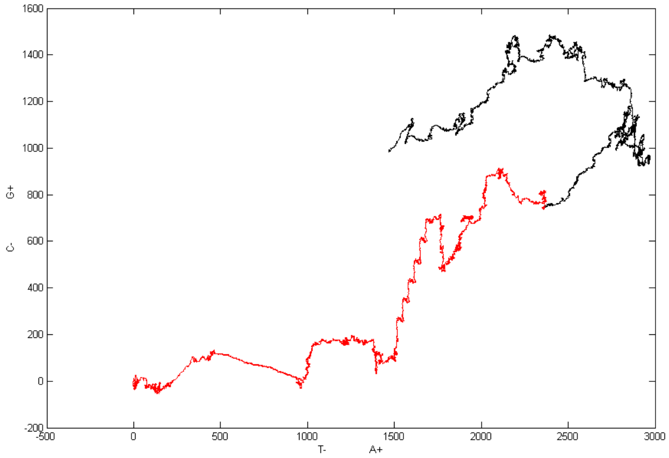

genes (CDS+introns)	GSS transformation

HUMAN

Homo sapiens, chromosome 18

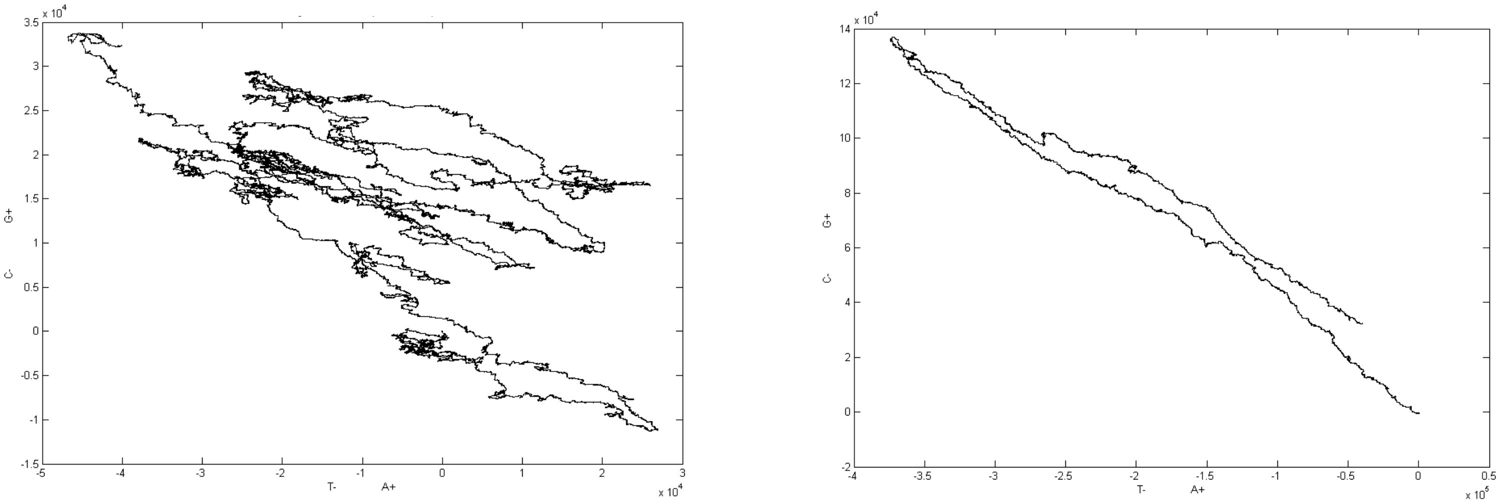

Homo sapiens, chromosome 19

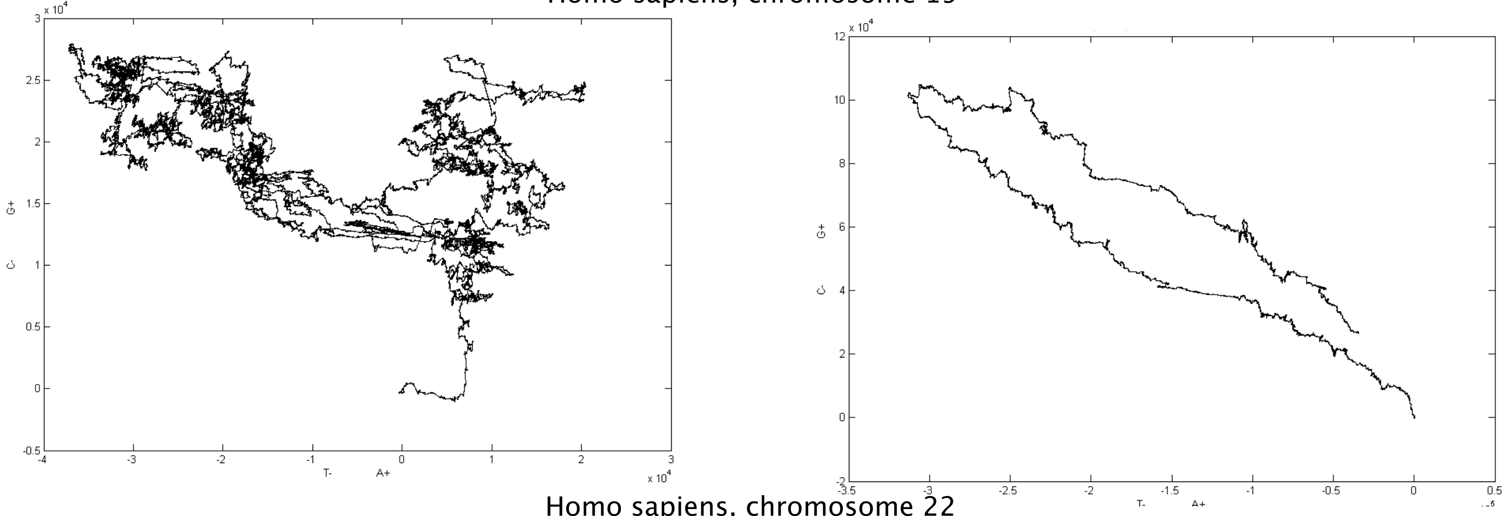

Homo sapiens, chromosome 22

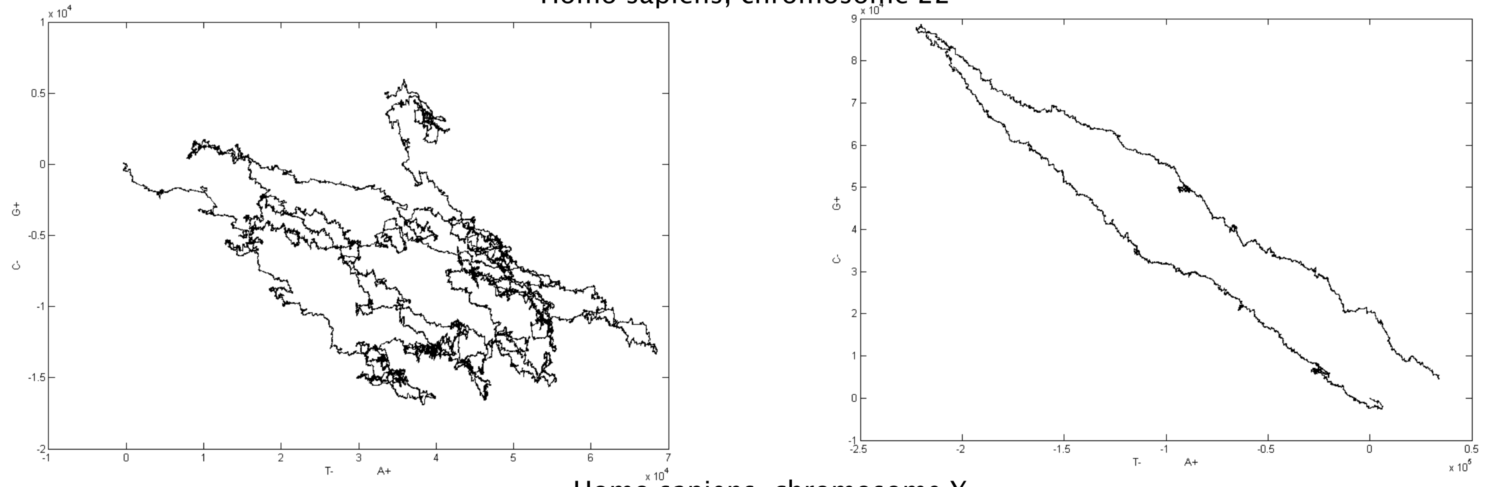

HUMAN

Homo sapiens, chromosome Y

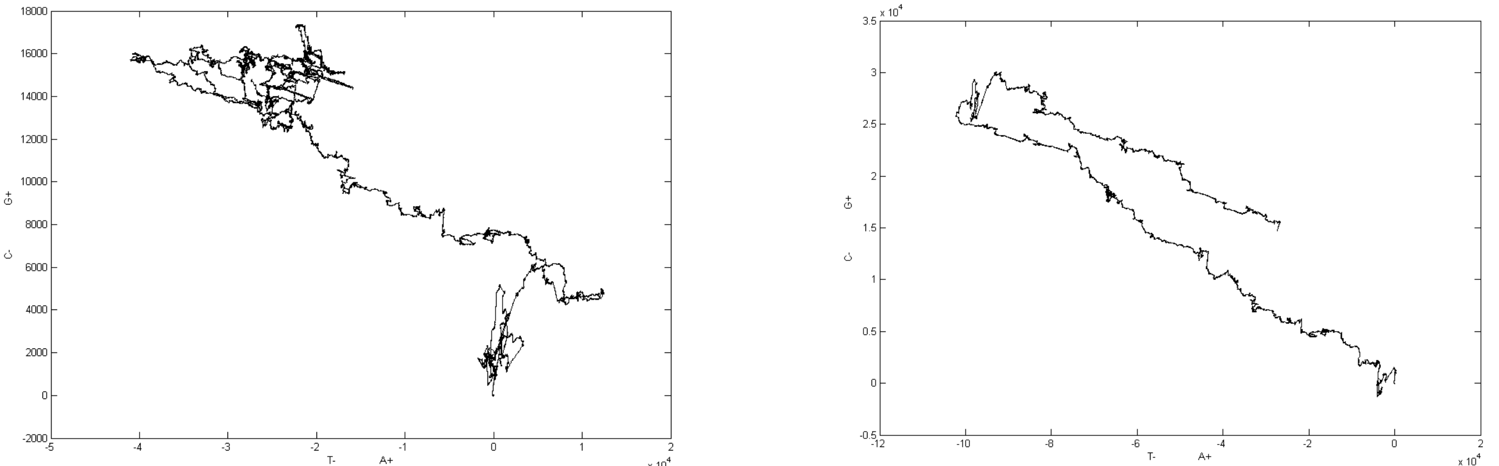

Supplement: Figure S1 — Examples of chromosome 2D DNA walks before and after GSS transformation. Trajectories of the genes in the original order are shown on the left, GSS transformed trajectories are on the right. The figure occupies the next 16 pages. Presented are 12 chromosomes of bacteria, 4 chromosomes of archaea, 20 chromosomes of fungi and 24 chromosomes of humans. (5.28 MB PDF) [file pone.0006396.s001.pdf]
