# Supplementary material for: Hidden Chromosome Symmetry: In Silico Transformation Reveals Symmetry in 2D DNA Walk Trajectories of 671 Chromosomes
Source: PLoS One. 2009 Jul 28;4(7):e6396. doi: 10.1371/journal.pone.0006396 (PMC2712679; doi:10.1371/journal.pone.0006396)
Supplement: Figure S2 — Superposition of bidirectional promoter density with gene distribution over strands for Saccharomyces cerevisiae, chromosome 12. (0.15 MB PDF) [file pone.0006396.s002.pdf]

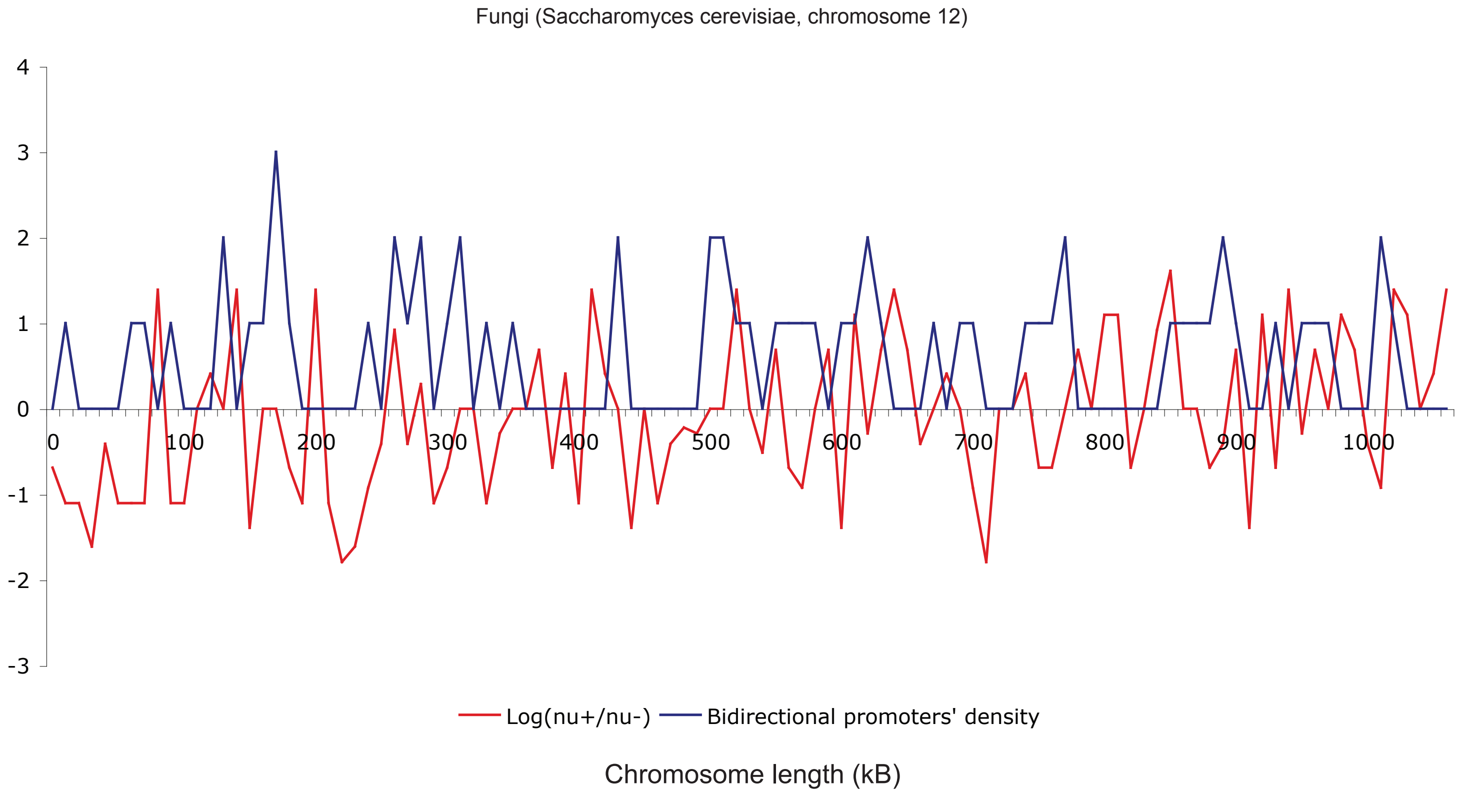


**Supplementary Figure 2. Superposition of bidirectional promoter density with gene distribution over strands for Saccharomyces cerevisiae, chromosome 12.**
