# Supplementary material for: Hidden Chromosome Symmetry: In Silico Transformation Reveals Symmetry in 2D DNA Walk Trajectories of 671 Chromosomes
Source: PLoS One. 2009 Jul 28;4(7):e6396. doi: 10.1371/journal.pone.0006396 (PMC2712679; doi:10.1371/journal.pone.0006396)
Supplement: Figure S3 — Normalized frequency of clusters with genes continuously located on one strand calculated for (a) bacteria Bacillus anthracis Ames, (b) archaea Sulfalobus solfataricus, (c) fungi Saccharomyces cerevisiae, chromosome 12, (d) Homo sapiens, chromosome 8. (0.24 MB PDF) [file pone.0006396.s003.pdf]

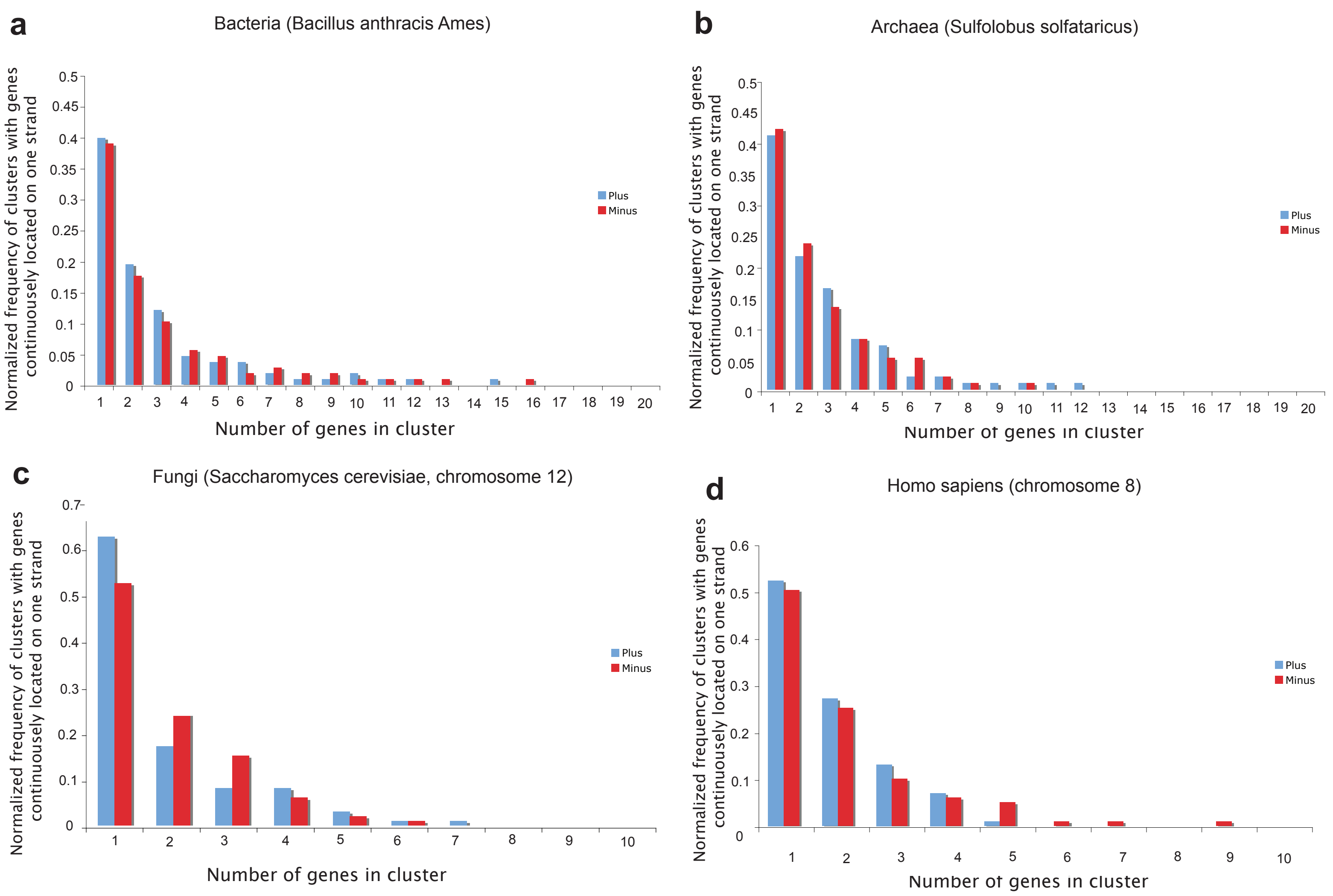

Supplementary Figure 3. Normalized frequency of clusters with genes continuously located on one strand calculated for “+” and “-“strand separately: (a) – bacteria Bacillus anthracis Ames, (b) – archaea Sulfalobus solfataricus, (c) – fungi Saccharomyces cerevisiae, chromosome 12, (d) – Homo sapiens, chromosome 8.
