# Supplementary material for: Hidden Chromosome Symmetry: In Silico Transformation Reveals Symmetry in 2D DNA Walk Trajectories of 671 Chromosomes
Source: PLoS One. 2009 Jul 28;4(7):e6396. doi: 10.1371/journal.pone.0006396 (PMC2712679; doi:10.1371/journal.pone.0006396)
Supplement: Figure S4 — Statistics for different replichores in bacteria. Relation between (a) number (Pearson coefficient r = 0.98), (b) cumulative gene length (r = 0.92), (c) cumulative GC (r = 0.97) and (d) AT (r = 0.97) skews of genes from different strands in different replichores in 524 bacteria. (1.13 MB PDF) [file pone.0006396.s004.pdf]

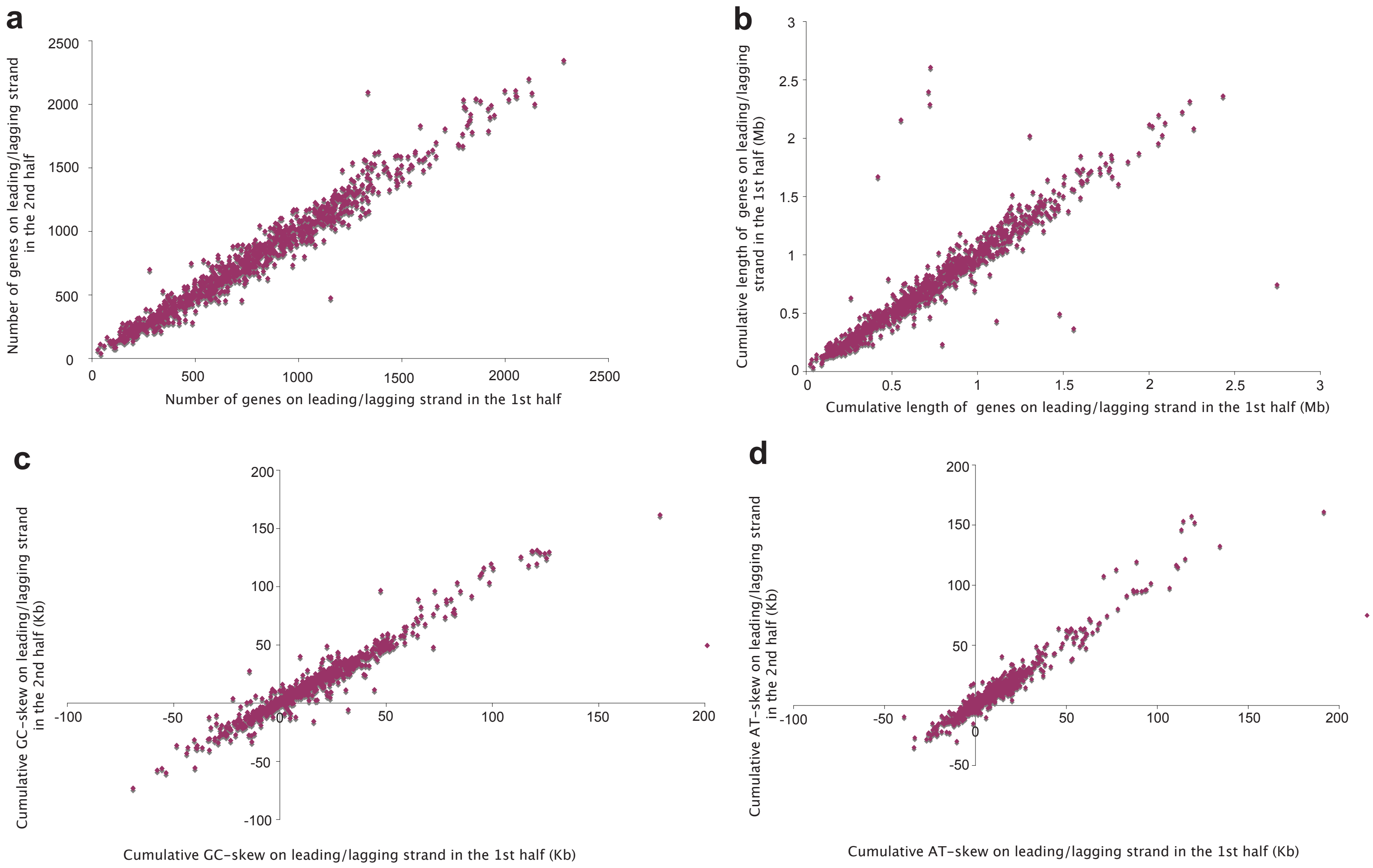

Supplementary Figure 4. Relation between (a) number (Pearson coefficient r=0.98), (b) cumulative gene length (r=0.92), (c) cumulative GC (r=0.97) and (d) AT (r=0.97) skews of genes from different strands in different replichores in 524 bacteria.
