# Supplementary material for: Hidden Chromosome Symmetry: In Silico Transformation Reveals Symmetry in 2D DNA Walk Trajectories of 671 Chromosomes
Source: PLoS One. 2009 Jul 28;4(7):e6396. doi: 10.1371/journal.pone.0006396 (PMC2712679; doi:10.1371/journal.pone.0006396)

Supplementary Figure 5. 2D DNA walks with marked sites of replication origin for archaeon Sulfolobus solfataricus and 16 chromosomes of fungus Saccharomyces cerevisiae. The figure occupies the next 4 pages.

Sulfalobus solfataricus

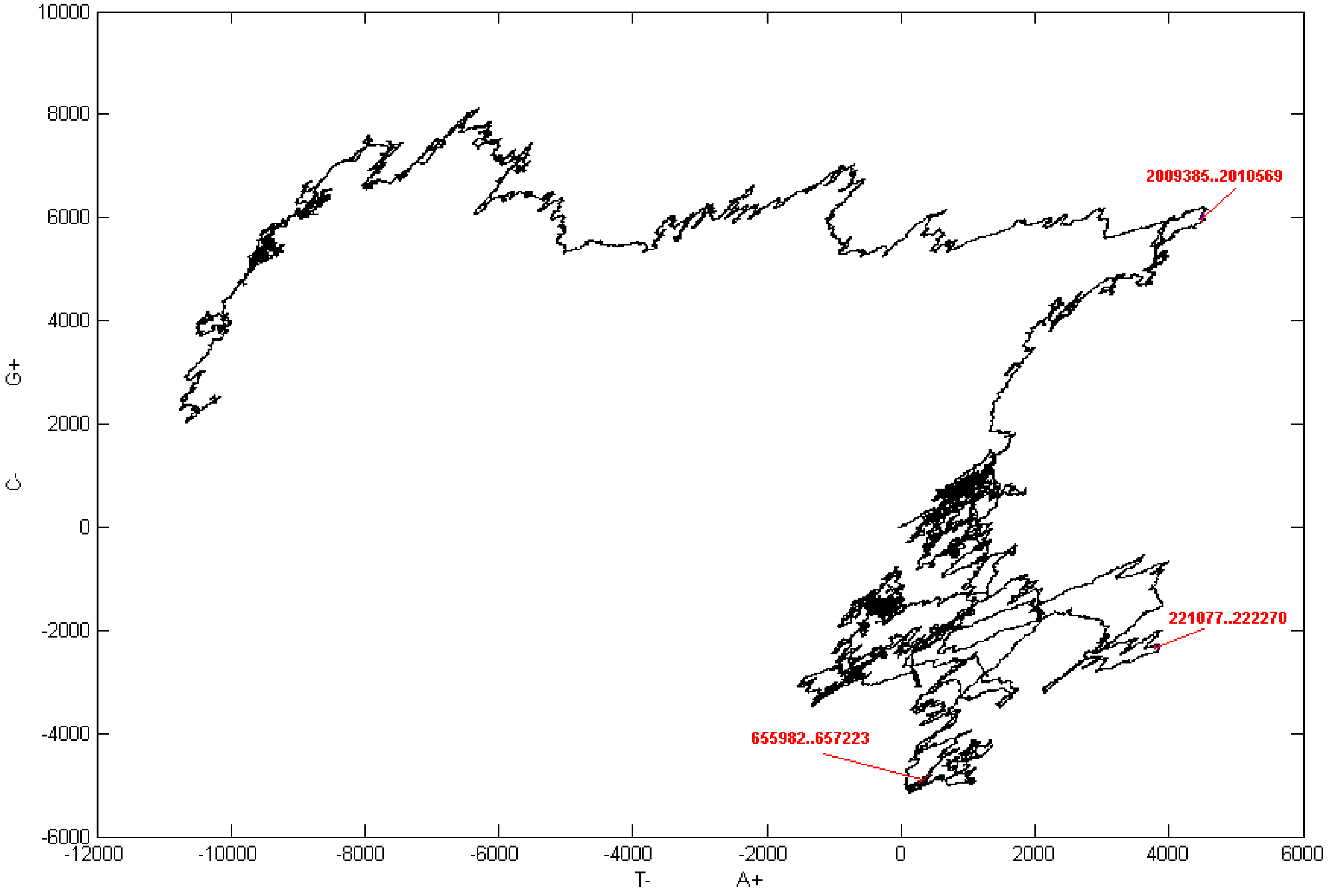

Saccharomyces cereviciae

chromosome 1

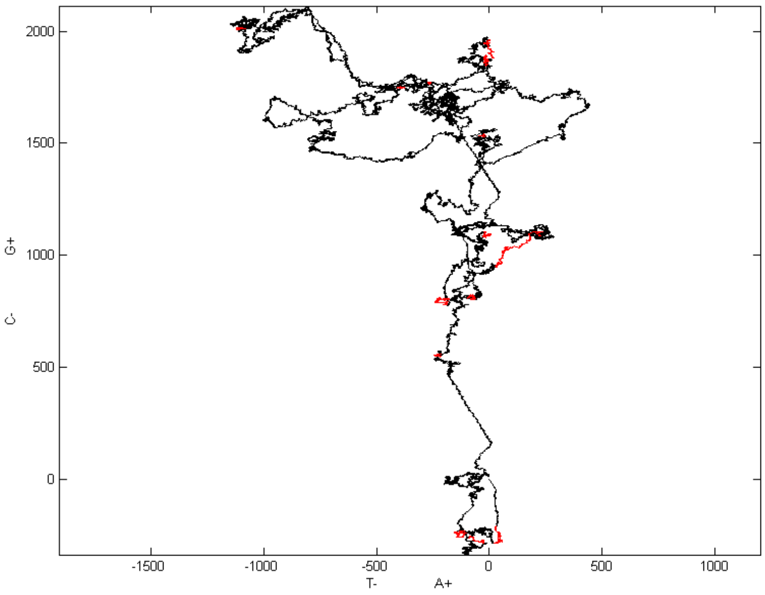

chromosome 2

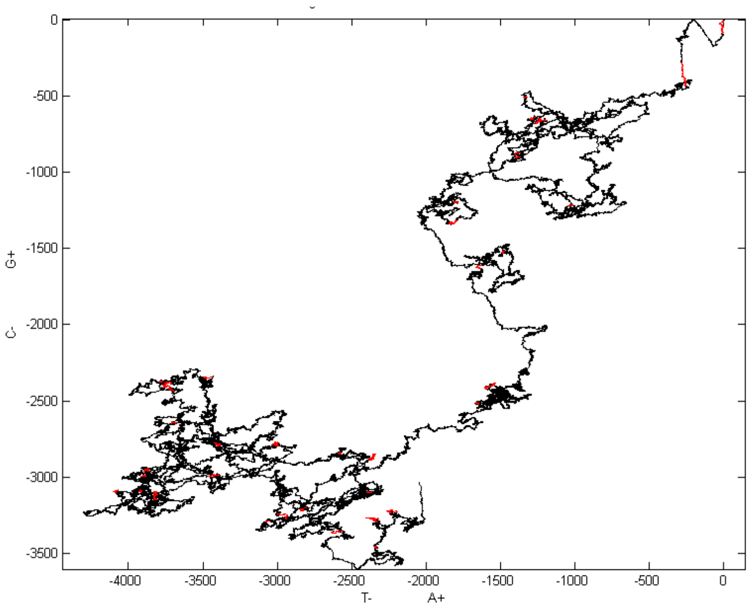

chromosome 3

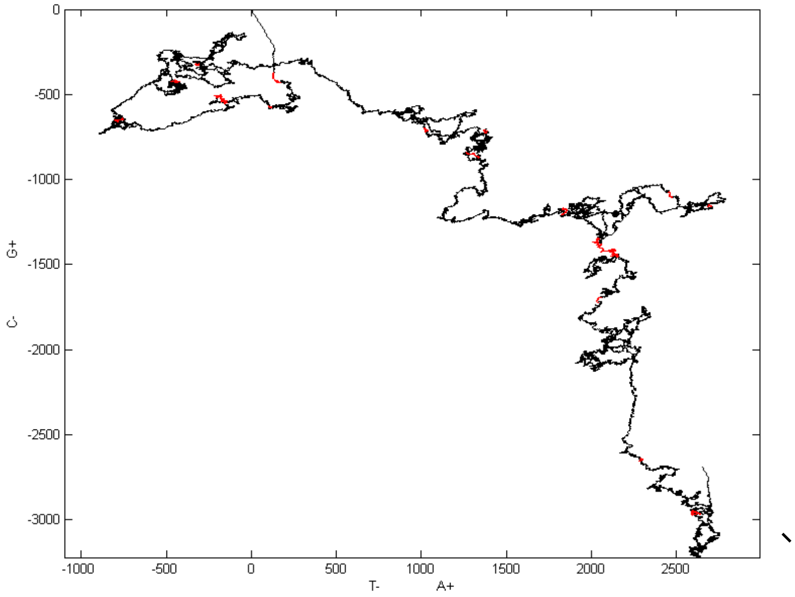

chromosome 4

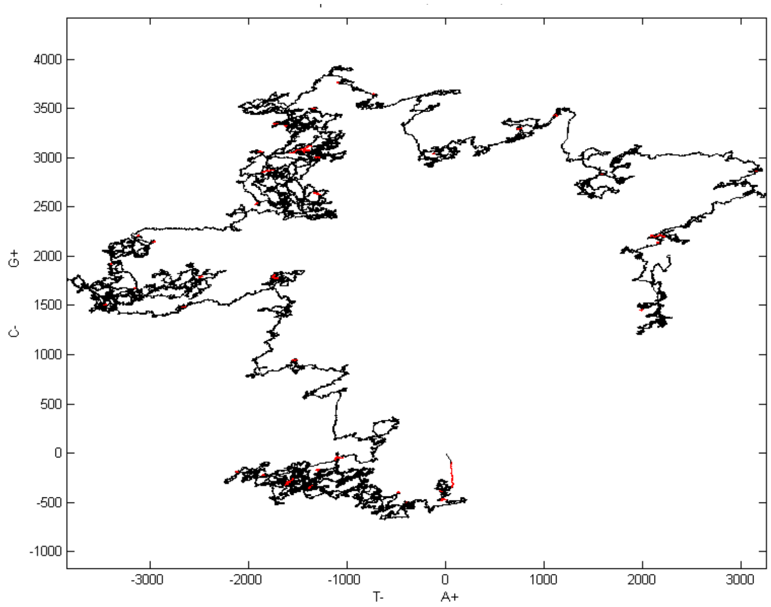

chromosome 5

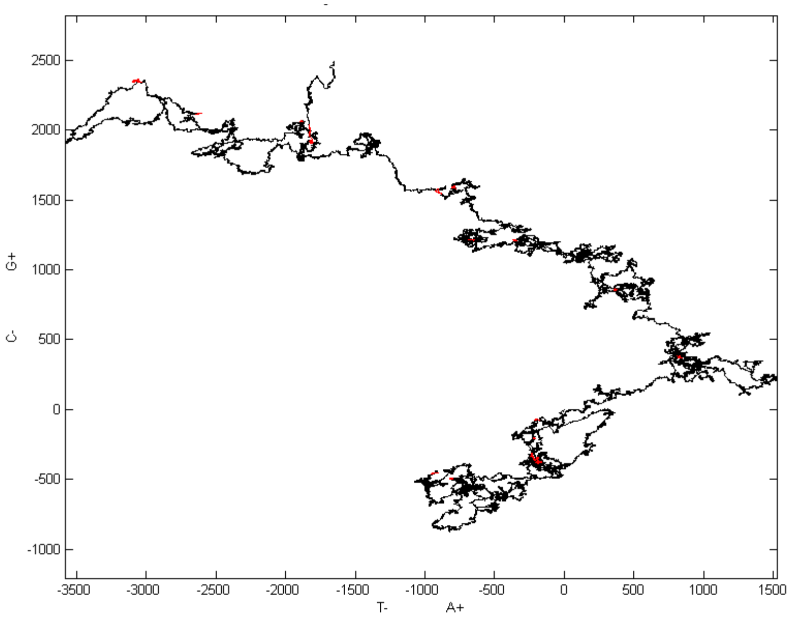

chromosome 6

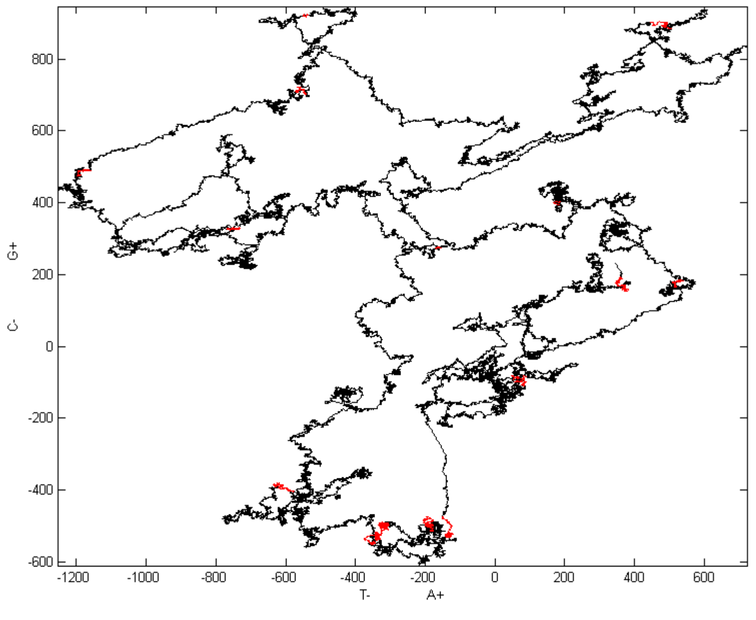

Saccharomyces cereviciae

chromosome 7

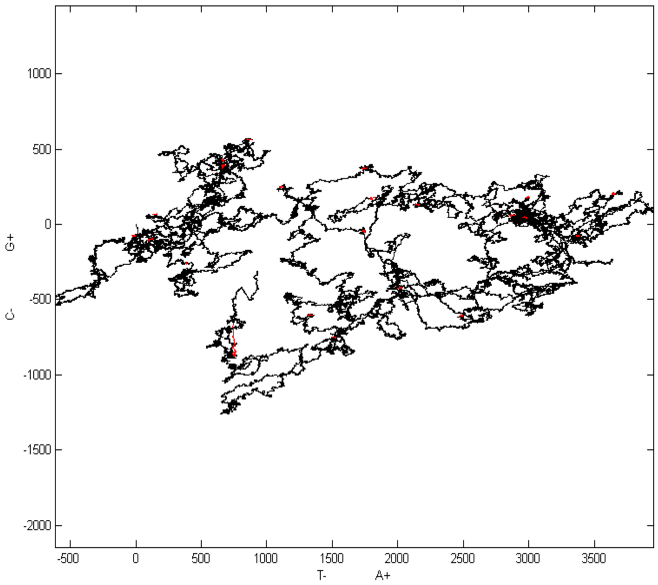

chromosome 8

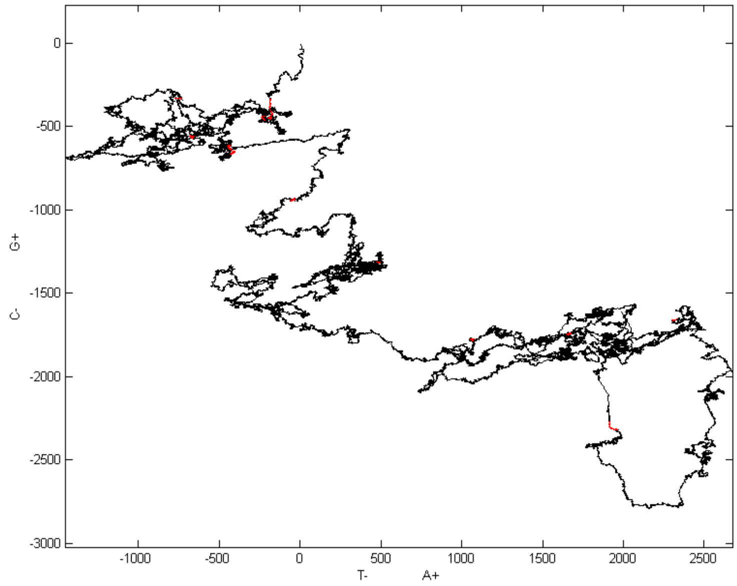

chromosome 9

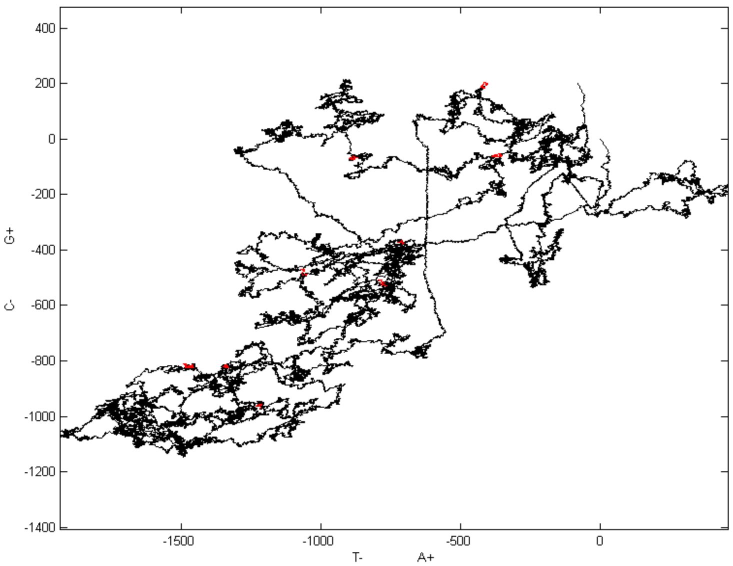

chromosome 10

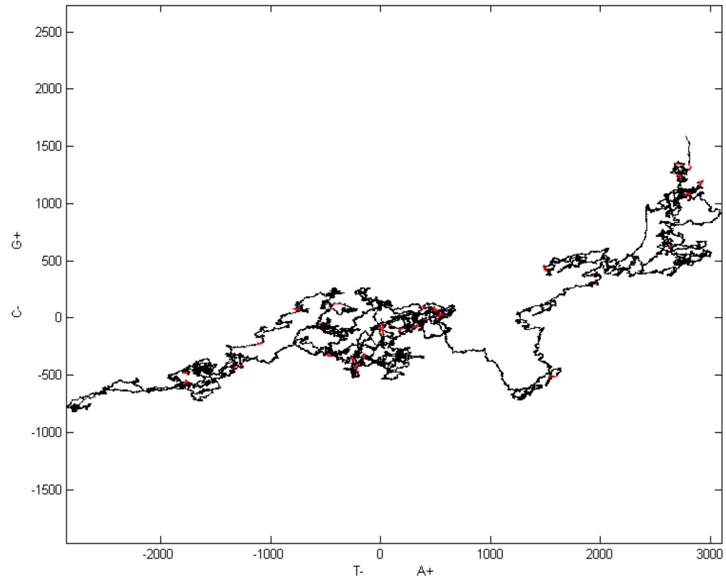

chromosome 11

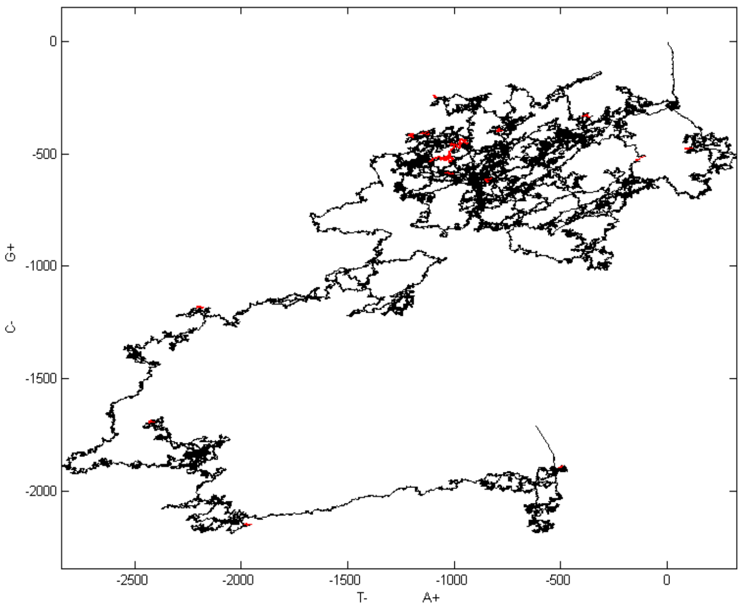

chromosome 12

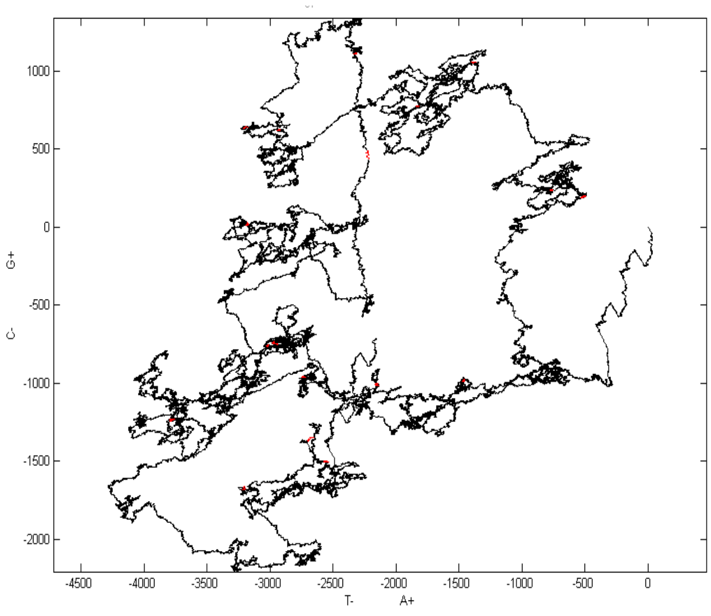

Saccharomyces cereviciae

chromosome 13

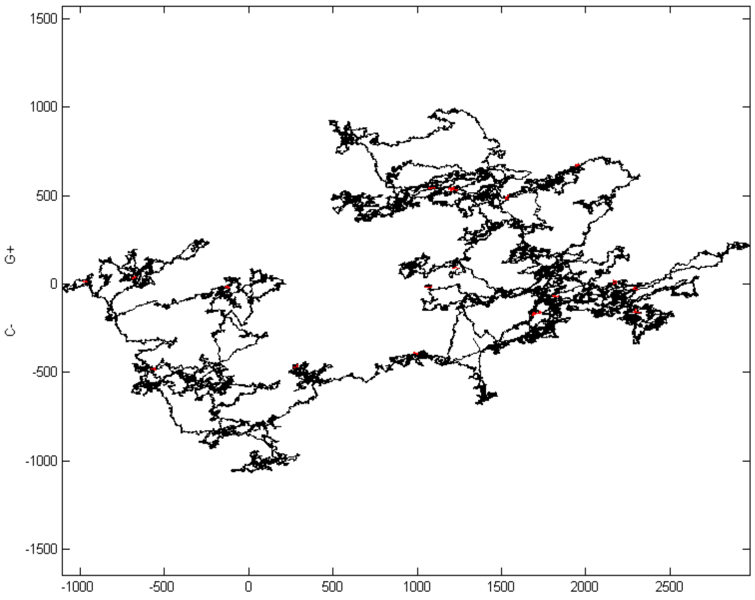

chromosome 14

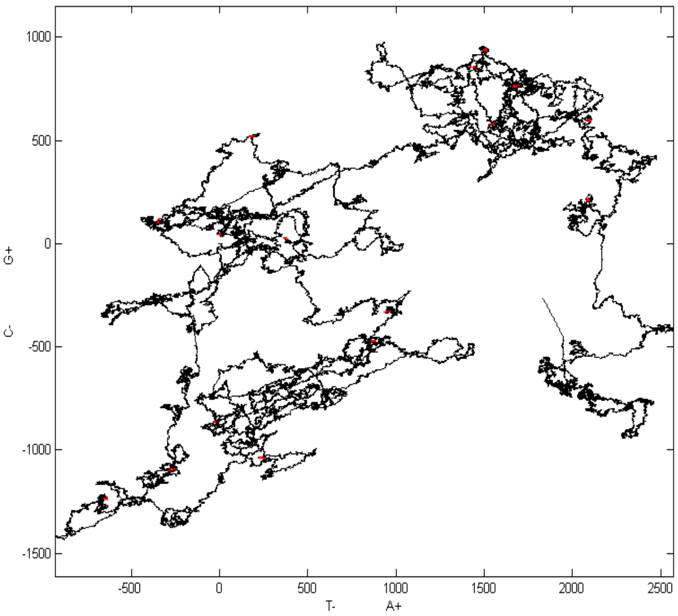

chromosome 15

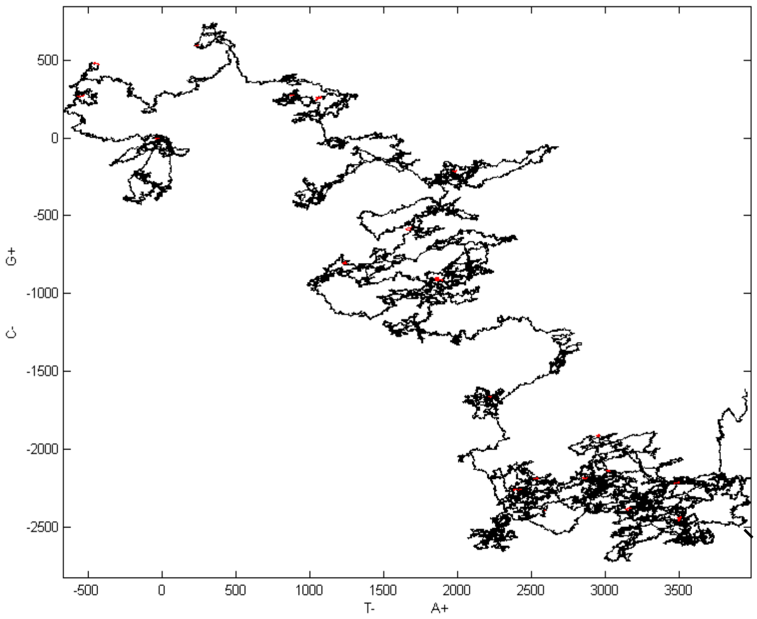

chromosome 16

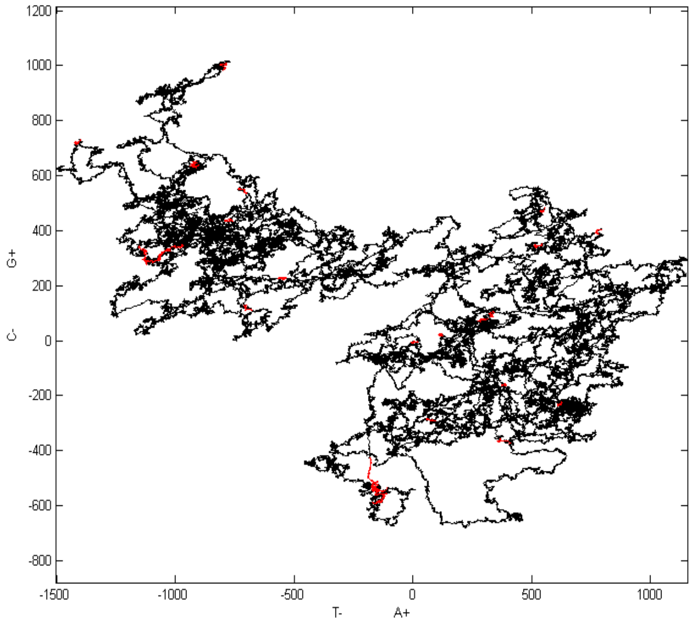

Supplement: Figure S5 — 2D DNA walks with the marked sites of replication origins for archaeon Sulfolobus solfataricus and Saccharomyces cerevisiae. (1.56 MB PDF) [file pone.0006396.s005.pdf]
