# Supplementary material for: Hidden Chromosome Symmetry: In Silico Transformation Reveals Symmetry in 2D DNA Walk Trajectories of 671 Chromosomes
Source: PLoS One. 2009 Jul 28;4(7):e6396. doi: 10.1371/journal.pone.0006396 (PMC2712679; doi:10.1371/journal.pone.0006396)
Supplement: Figure S6 — Detection of symmetrically correlated areas. (a) Plot of the Hausdorf distance dH(X,Y) and K-value (from Minimum Value Enclosed Ellipsoid method) against the sliding window N. (b) Detected correlated areas on the 2D DNA walk trajectory. (0.85 MB PDF) [file pone.0006396.s006.pdf]

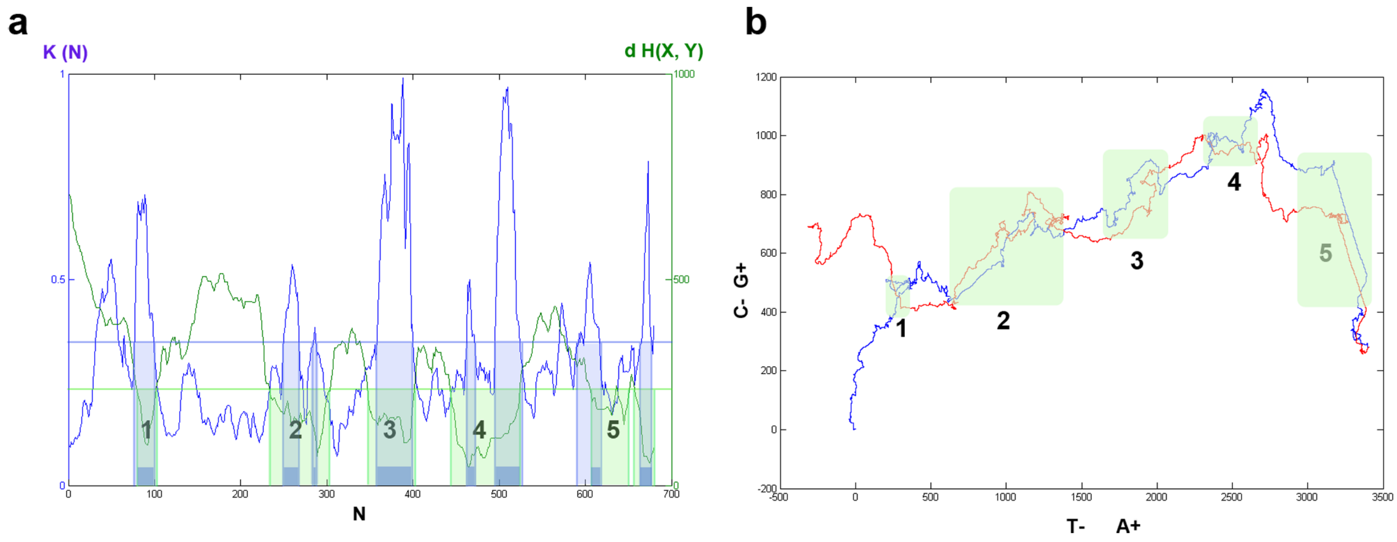

Supplemenatry Figure 6. Detection of symmetrically correlated areas. (a) Plot of the Hausdorf distance dH(X,Y) and K-value (from Minimum Value Enclosed Ellipsoid method) against the sliding window N. (b) Detected correlated areas on the 2D DNA walk trajectory..
