# Supplementary material for: Hidden Chromosome Symmetry: In Silico Transformation Reveals Symmetry in 2D DNA Walk Trajectories of 671 Chromosomes
Source: PLoS One. 2009 Jul 28;4(7):e6396. doi: 10.1371/journal.pone.0006396 (PMC2712679; doi:10.1371/journal.pone.0006396)
Supplement: Table S1 — Total number, total length and cumulative GC and AT skews of genes located on different strands for 671 chromosomes: 524 of bacteria, 36 of archaea, 87 of fungi and 24 of humans. (0.16 MB PDF) [file pone.0006396.s007.pdf]

Supplementary Table 1. Total number, total length and cumulative GC and AT skews of genes located on different strands for 671 chromosomes: 524 of bacteria, 36 of archaea, 87 of fungi and 24 of humans.

Bacteria

Chromosome	Nu of genes on "+"-strand (Nu+)	Nu of genes on "-"-strand (Nu-)	Nu+/Nu-	Total length of genes on "+"-strand (Length+)	Total length of genes on "-"-strand (Length-)	Length+/ Length-	Cumulative skew (G-C) of genes on "+"-strand (G-C)+	Cumulative skew (G-C) of genes on "-"-strand (G-C)-	(G-C)+/ (G-C)-	Cumulative skew (A-T) of genes on "+"-strand (A-T)+	Cumulative skew (A-T) of genes on "-"-strand (A-T)-	(A-T)+/ (A-T)-
Acidobacteria bacterium Ellin345	2348	2428	0.97	2485641	2565678	0.97	6963	2384	2.92	44636	42174	1.06
Acidothermus cellulolyticus 11B	1135	1021	1.11	1167375	1028502	1.14	1901	453	4.2	-18662	-17685	1.06
Acidovorax avenae citrulli AAC00-1	2406	2302	1.05	2418528	2357007	1.03	-19551	-25836	0.76	21425	21997	0.97
Acidovorax JS42	1938	2068	0.94	1877491	2031237	0.92	-5146	-7691	0.67	18494	19862	0.93
Acinetobacter baumannii ATCC 17978	1755	1596	1.1	1473618	1383735	1.06	43277	38002	1.14	1723	1277	1.35
Acinetobacter sp ADP1	1737	1587	1.09	1643622	1520103	1.08	48073	41832	1.15	-1453	-2259	0.64
Actinobacillus pleuropneumoniae L20	990	1021	0.97	942459	1021107	0.92	34492	36038	0.96	20997	23013	0.91
Actinobacillus succinogenes 130Z	1070	1008	1.06	1048518	990627	1.06	37029	34829	1.06	15027	13448	1.12
Aeromonas hydrophila ATCC 7966	2102	2019	1.04	2100217	2053059	1.02	-20159	-21716	0.93	14471	11531	1.25
Aeromonas salmonicida A449	2104	1981	1.06	2028994	1843402	1.1	-1809	-6182	0.29	3899	7684	0.51
Agrobacterium tumefaciens C58 Cereon	1367	1347	1.01	1277109	1258800	1.01	-13600	1380	-9.86	-5635	-12112	0.47
Agrobacterium tumefaciens C58 UWash	1398	1386	1.01	1250658	1244688	1	-14551	850	-17.12	-6581	-12454	0.53
Alcanivorax borkumensis SK2	1441	1313	1.1	1396611	1339368	1.04	33909	31093	1.09	-3954	-3345	1.18
Alkalilimnicola ehrlichei MLHE-1	1467	1397	1.05	1518555	1446420	1.05	12567	13221	0.95	1576	-2153	-0.73
Alkaliphilus metalliredigens QYMF	2360	2264	1.04	2057326	2033157	1.01	136268	129787	1.05	128382	129438	0.99
Anabaena variabilis ATCC 29413	2668	2374	1.12	2796936	2398332	1.17	40430	34981	1.16	51118	40587	1.26
Anaeromyxobacter dehalogenans 2CP-C	2075	2270	0.91	2184345	2379627	0.92	13556	21223	0.64	-16929	-11476	1.48
Anaeromyxobacter Fw109-5	2221	2244	0.99	2306016	2441010	0.94	-10012	-3467	2.89	-13726	-14029	0.98
Anaplasma marginale St Maries	416	532	0.78	469614	556233	0.84	30634	36427	0.84	5154	5370	0.96
Anaplasma phagocytophilum HZ	643	620	1.04	513357	490492	1.05	45605	36714	1.24	-5092	-453	11.24
Aquifex aeolicus	813	715	1.14	771846	680898	1.13	37111	35861	1.03	65955	62089	1.06
Arthrobacter aurescens TC1	1968	2072	0.95	1996035	2060700	0.97	-31353	-29848	1.05	8024	536	14.97
Arthrobacter FB24	2064	2081	0.99	2102403	2107080	1	-42481	-39818	1.07	20934	16738	1.25
Aster yellows witches-broom phytoplasma AYWB	291	379	0.77	216936	302640	0.72	-3182	-4516	0.7	16728	20876	0.8
Azoarcus BH72	2073	1915	1.08	2099130	1899363	1.11	-29354	-22715	1.29	8438	6248	1.35
Azoarcus sp EbN1	2111	2021	1.04	1991570	1930042	1.03	-1737	-2982	0.58	12863	12699	1.01
Bacillus anthracis Ames	2542	2768	0.92	1989316	2231865	0.89	116114	130738	0.89	99886	112362	0.89
Bacillus anthracis Ames 0581	2541	2767	0.92	1988749	2232063	0.89	116087	130720	0.89	99774	112402	0.89
Bacillus anthracis str Sterne	2556	2730	0.94	2090385	2307378	0.91	121643	135704	0.9	105352	116469	0.9
Bacillus cereus ATCC 10987	2772	2830	0.98	2210802	2236666	0.99	129041	131910	0.98	113967	111933	1.02
Bacillus cereus ATCC14579	2610	2623	1	2158917	2211108	0.98	127977	128497	1	112932	113529	0.99
Bacillus cereus cytotoxis NVH 391-98	1828	2004	0.91	1539201	1706616	0.9	87319	98882	0.88	77526	86254	0.9
Bacillus cereus ZK	2469	2664	0.93	2147754	2307510	0.93	123432	135717	0.91	108182	116811	0.93
Bacillus clausii KSM-K16	1992	2103	0.95	1788597	1910151	0.94	67877	73293	0.93	46842	51622	0.91
Bacillus halodurans	2112	1953	1.08	1885934	1694046	1.11	91506	80721	1.13	51291	49577	1.03
Bacillus licheniformis ATCC 14580	1999	2152	0.93	1783866	1849281	0.96	61171	62587	0.98	83955	87716	0.96
Bacillus licheniformis DSM 13	2029	2166	0.94	1810464	1871667	0.97	62358	62998	0.99	84380	89425	0.94
Bacillus subtilis	1941	2163	0.9	1797972	1879532	0.96	68563	73590	0.93	76509	82121	0.93
Bacillus thuringiensis Al Hakam	2320	2415	0.96	2119269	2236860	0.95	125110	131716	0.95	112525	115474	0.97
Bacillus thuringiensis konkukian	2464	2652	0.93	2103120	2291802	0.92	120812	133957	0.9	105316	115676	0.91
Bacteroides fragilis NCTC 9434	2071	2112	0.98	2279148	2310039	0.99	85245	80953	1.05	61227	62148	0.99
Bacteroides fragilis YCH46	2234	2343	0.95	2338026	2417463	0.97	84977	84795	1	60965	67690	0.9
Bacteroides thetaiotaomicron VPI-5482	2364	2413	0.98	2821221	2784939	1.01	100647	91039	1.11	84604	98644	0.86
Bacteroides vulgatus ATCC 8482	2055	2009	1.02	2272125	2295108	0.99	95314	104768	0.91	68607	63518	1.08
Bartonella bacilliformis KC583	764	518	1.47	680422	485553	1.4	27848	13199	2.11	-13885	-5076	2.74
Bartonella henselae Houston-1	749	738	1.01	713748	691674	1.03	24083	21777	1.11	-2327	-9641	0.24
Bartonella quintana Toulouse	578	563	1.03	561141	580461	0.97	17749	18879	0.94	-7750	-10964	0.71
Baumannia cicadellinicola Homalodisca coagulata	300	294	1.02	295917	289398	1.02	7166	7090	1.01	5160	5634	0.92
Bdellovibrio bacteriovorus	1885	1701	1.11	1820340	1697949	1.07	33368	30609	1.09	18216	19948	0.91
Bifidobacterium adolescentis ATCC 15703	866	764	1.13	953580	855501	1.11	-5376	-5469	0.98	20736	18161	1.14
Bifidobacterium longum	859	867	0.99	970518	955446	1.02	-18795	-16829	1.12	13791	11875	1.16
Bordetella bronchiseptica	2615	2378	1.1	2572557	2332927	1.1	-9307	-24459	0.38	7640	9595	0.8
Bordetella parapertussis	2259	1925	1.17	2221879	1910899	1.16	-4856	-25811	0.19	3794	6617	0.57
Bordetella pertussis	1756	1679	1.05	1746117	1630361	1.07	-17214	-24838	0.69	7919	7478	1.06
Borrelia afzelii PKo	421	434	0.97	422646	426177	0.99	20521	22104	0.93	15431	18367	0.84
Borrelia burgdorferi	427	423	1.01	427116	425124	1	20708	21587	0.96	15535	18667	0.83
Borrelia garinii PBi	408	423	0.96	417864	422034	0.99	20215	21420	0.94	16057	18856	0.85
Bradyrhizobium BTAi1	3743	3650	1.03	3599502	3495729	1.03	-18847	-11922	1.58	-17705	-13329	1.33
Bradyrhizobium japonicum	4147	4169	0.99	3933600	3994416	0.98	-37969	-39499	0.96	6921	10867	0.64
Bradyrhizobium ORS278	3319	3397	0.98	3140868	3250626	0.97	-24649	-27221	0.91	-3089	-2705	1.14
Brucella abortus 9-941	991	1038	0.95	862746	859899	1	5114	8772	0.58	-6240	-7043	0.89
Brucella melitensis	1053	1005	1.05	912246	907452	1.01	9421	5951	1.58	-7606	-7181	1.06

Chromosome	Nu of genes on "+"-strand (Nu+)	Nu of genes on "-"-strand (Nu-)	Nu+/Nu-	Total length of genes on "+"-strand (Length+)	Total length of genes on "-"-strand (Length-)	Length+/ Length-	Cumulative skew (G-C) of genes on "+"-strand (G-C)+	Cumulative skew (G-C) of genes on "-"-strand (G-C)-	(G-C)+/ (G-C)-	Cumulative skew (A-T) of genes on "+"-strand (A-T)+	Cumulative skew (A-T) of genes on "-"-strand (A-T)-	(A-T)+/ (A-T)-
Brucella melitensis biovar Abortus	971	1028	0.94	852912	856557	1	5005	8692	0.58	-6495	-6911	0.94
Brucella ovis	494	467	1.06	460554	431022	1.07	1568	3130	0.5	-5926	-4856	1.22
Brucella suis 1330	1025	1097	0.93	884999	894978	0.99	5276	8744	0.6	-7951	-7676	1.04
Buchnera aphidicola	262	241	1.09	260391	239064	1.09	7953	7129	1.12	10834	13701	0.79
Buchnera aphidicola Sg	297	248	1.2	294750	240639	1.22	9310	7678	1.21	16542	13721	1.21
Burkholderia 383	1581	1592	0.99	1573080	1573050	1	14244	10258	1.39	-1712	-180	9.51
Burkholderia cenocepacia AU 1054	1449	1515	0.96	1433007	1447347	0.99	3343	10895	0.31	10262	4634	2.21
Burkholderia cenocepacia HI2424	1641	1517	1.08	1588158	1462809	1.09	10271	4048	2.54	11753	9351	1.26
Burkholderia cepacia AMMD	1713	1499	1.14	1645680	1465956	1.12	9226	7997	1.15	12664	9711	1.3
Burkholderia mallei ATCC 23344	1358	1636	0.83	1273954	1531983	0.83	4304	20115	0.21	5907	5366	1.1
Burkholderia mallei NCTC 10229	1756	1576	1.11	1552554	1440675	1.08	13521	13125	1.03	5199	5416	0.96
Burkholderia mallei NCTC 10247	1737	1738	1	1505217	1526199	0.99	16935	10737	1.58	3617	6827	0.53
Burkholderia mallei SAVP1	1574	1880	0.84	1358763	1807960	0.75	10263	16626	0.62	2888	7713	0.37
Burkholderia pseudomallei 1106a	1902	2116	0.9	1642494	1850757	0.89	11003	18391	0.6	1423	7030	0.2
Burkholderia pseudomallei 1710b	1800	1935	0.93	2068170	2188701	0.94	7058	13227	0.53	256	7912	0.03
Burkholderia pseudomallei 668	1897	2053	0.92	1635387	1788150	0.91	11336	16780	0.68	1621	5580	0.29
Burkholderia pseudomallei K96243	1527	1871	0.82	1532791	1860915	0.82	12176	18452	0.66	2050	8787	0.23
Burkholderia thailandensis E264	1667	1608	1.04	1686819	1627891	1.04	26083	1416	18.42	2358	8204	0.29
Burkholderia vietnamiensis G4	1656	1617	1.02	1589831	1564683	1.02	6715	6223	1.08	15402	13910	1.11
Burkholderia xenovorans LB400	2207	2222	0.99	2152695	2057268	1.05	24089	19663	1.23	8096	10081	0.8
Caldicellulosiruptor saccharolyticus DSM 8903	1669	1009	1.65	1611867	952392	1.69	112098	57177	1.96	101331	61887	1.64
Campylobacter curvus 525 92	917	1013	0.91	826206	877629	0.94	26834	29426	0.91	48032	56783	0.85
Campylobacter fetus 82-40	960	758	1.27	894834	707913	1.26	45161	38877	1.16	59147	43478	1.36
Campylobacter hominis ATCC BAA-381	846	835	1.01	719784	725832	0.99	34404	33360	1.03	47504	48554	0.98
Campylobacter jejuni	807	826	0.98	735216	795711	0.92	38112	42627	0.89	27508	24684	1.11
Campylobacter jejuni 81-176	818	834	0.98	736611	779718	0.94	38007	41338	0.92	27318	23712	1.15
Campylobacter jejuni doylei 269 97	771	959	0.8	698682	827388	0.84	36320	44505	0.82	28536	30285	0.94
Campylobacter jejuni RM1221	883	954	0.93	778401	842868	0.92	40274	44553	0.9	33815	30521	1.11
Candidatus Blochmannia floridanus	335	247	1.36	333123	252927	1.32	16360	9289	1.76	5607	4282	1.31
Candidatus Blochmannia pennsylvanicus BPEN	350	259	1.35	344148	262089	1.31	10359	9283	1.12	10127	2932	3.45
Candidatus Methanoregula boonei 6A8	1208	1241	0.97	1109301	1079790	1.03	3263	630	5.18	20910	16660	1.26
Candidatus Pelagibacter ubique HTCC1062	672	681	0.99	643380	610101	1.05	29685	27982	1.06	43637	39597	1.1
Candidatus Ruthia magnifica Cm Calyptogena magnifica	521	454	1.15	492657	426894	1.15	17713	15485	1.14	7198	5765	1.25
Candidatus Vesicomyosocius okutanii HA	500	436	1.15	463287	407403	1.14	17177	16592	1.04	8548	5081	1.68
Carboxydothermus hydrogenoformans Z-2901	1303	1316	0.99	1099561	1071879	1.03	69072	66582	1.04	28762	25138	1.14
Caulobacter crescentus	1827	1909	0.96	1826427	1811412	1.01	-19794	-15242	1.3	3695	-1584	-2.33
Chlamydia muridarum	453	450	1.01	503031	461328	1.09	8804	8338	1.06	-8098	-7651	1.06
Chlamydia trachomatis	441	453	0.97	565021	459367	1.23	7205	8456	0.85	-6579	-6939	0.95
Chlamydia trachomatis A HAR-13	453	457	0.99	479658	459394	1.04	7155	8214	0.87	-6697	-6934	0.97
Chlamydophila abortus S26 3	477	454	1.05	505663	496413	1.02	3807	4856	0.78	-1055	-1665	0.63
Chlamydophila caviae	515	482	1.07	534282	514824	1.04	5919	6728	0.88	-283	-2146	0.13
Chlamydophila felis Fe C-56	495	509	0.97	531867	531003	1	5227	4546	1.15	-1584	-137	11.56
Chlamydophila pneumoniae AR39	552	559	0.99	533709	561828	0.95	3084	3046	1.01	-3240	-870	3.72
Chlamydophila pneumoniae CWL029	521	530	0.98	557325	531082	1.05	2714	3064	0.89	-657	-3266	0.2
Chlamydophila pneumoniae J138	533	535	1	565137	536166	1.05	2155	3142	0.69	-384	-3179	0.12
Chlamydophila pneumoniae TW 183	548	564	0.97	567657	539385	1.05	2309	3031	0.76	-746	-3384	0.22
Chlorobium chlorochromatii CaD3	1048	953	1.1	1205889	1066206	1.13	33170	25260	1.31	-591	-3942	0.15
Chlorobium phaeobacteroides DSM 266	1150	1499	0.77	1106739	1528839	0.72	18590	34568	0.54	9755	20023	0.49
Chlorobium tepidum TLS	1131	1120	1.01	990960	906294	1.09	-6465	428	-15.11	20205	13299	1.52
Chromobacterium violaceum	2101	2305	0.91	1997559	2209350	0.9	7810	9566	0.82	28669	32140	0.89
Chromohalobacter salexigens DSM 3043	1722	1575	1.09	1718769	1590153	1.08	-131	-12141	0.01	-3920	-3710	1.06
Clavibacter michiganensis NCPPB 382	1465	1518	0.97	1467249	1492332	0.98	-56013	-58173	0.96	-4680	511	-9.16
Clostridium acetobutylicum	1859	1812	1.03	1713045	1668648	1.03	123849	124193	1	133614	133883	1
Clostridium beijerinckii NCIMB 8052	2621	2398	1.09	2469390	2296524	1.08	190837	170728	1.12	212465	177348	1.2
Clostridium botulinum A	1685	1888	0.89	1476794	1679451	0.88	108675	126630	0.86	135086	169904	0.8
Clostridium botulinum A ATCC 19397	1605	1946	0.82	1421184	1715358	0.83	103923	129212	0.8	129109	174882	0.74
Clostridium botulinum A Hall	1584	1822	0.87	1402077	1630011	0.86	102928	122153	0.84	127115	163704	0.78
Clostridium botulinum F Langeland	1648	1986	0.83	1481643	1743393	0.85	107811	132555	0.81	133908	178252	0.75
Clostridium difficile 630	1898	1843	1.03	1781494	1774568	1	136358	130170	1.05	155690	150857	1.03
Clostridium kluyveri DSM 555	1723	2114	0.82	1522410	1824459	0.83	107807	127239	0.85	120899	138742	0.87
Clostridium novyi NT	1216	1108	1.1	1178892	1036476	1.14	83221	76836	1.08	121453	107358	1.13
Clostridium perfringens	1198	1461	0.82	1123512	1406499	0.8	89652	118362	0.76	86036	132011	0.65
Clostridium perfringens ATCC 13124	1326	1549	0.86	1250763	1455928	0.86	100653	120519	0.84	98960	135344	0.73
Clostridium perfringens SM101	1123	1434	0.78	1022766	1335556	0.77	78825	109216	0.72	79113	125187	0.63
Clostridium tetani E88	1087	1285	0.85	1103682	1294473	0.85	83228	95708	0.87	117104	131845	0.89
Clostridium thermocellum ATCC 27405	1586	1604	0.99	1596396	1621308	0.98	107307	110421	0.97	105863	110501	0.96

Chromosome	Nu of genes on "+"-strand (Nu+)	Nu of genes on "-"-strand (Nu-)	Nu+/Nu-	Total length of genes on "+"-strand (Length+)	Total length of genes on "-"-strand (Length-)	Length+/ Length-	Cumulative skew (G-C) of genes on "+"-strand (G-C)+	Cumulative skew (G-C) of genes on "-"-strand (G-C)-	(G-C)+/ (G-C)-	Cumulative skew (A-T) of genes on "+"-strand (A-T)+	Cumulative skew (A-T) of genes on "-"-strand (A-T)-	(A-T)+/ (A-T)-
Colwellia psychrerythraea 34H	2522	2387	1.06	2295816	2255431	1.02	66421	65776	1.01	33451	30600	1.09
Corynebacterium diphtheriae	1156	1115	1.04	1083012	1105999	0.98	2551	1568	1.63	-5815	-5455	1.07
Corynebacterium efficiens YS-314	1483	1466	1.01	1448964	1439919	1.01	-29203	-34000	0.86	-3835	-1223	3.14
Corynebacterium glutamicum ATCC 13032 Bielefeld	1500	1556	0.96	1370307	1503096	0.91	-12671	-8451	1.5	-5352	-4999	1.07
Corynebacterium glutamicum ATCC 13032 Kitasato	1482	1510	0.98	1368018	1491123	0.92	-13567	-8527	1.59	-5031	-4646	1.08
Corynebacterium glutamicum R	1489	1562	0.95	1405209	1472466	0.95	-10880	-12525	0.87	-7508	-7929	0.95
Corynebacterium jeikeium K411	971	1132	0.86	998904	1203462	0.83	-604	6256	-0.1	15018	20566	0.73
Coxiella burnetii	1056	959	1.1	898053	795084	1.13	16823	16170	1.04	7260	4964	1.46
Cyanobacteria bacterium Yellowstone A-Prime	1341	1418	0.95	1212840	1274253	0.95	6496	5075	1.28	-16222	-15606	1.04
Cyanobacteria bacterium Yellowstone B-Prime	1436	1425	1.01	1263603	1336599	0.95	12546	13715	0.91	-17565	-21556	0.81
Cytophaga hutchinsonii ATCC 33406	1899	1885	1.01	2014059	1980033	1.02	48907	49689	0.98	76700	71484	1.07
Dechloromonas aromatica RCB	2152	2018	1.07	2152269	1974936	1.09	-16954	-15598	1.09	-423	5182	-0.08
Dehalococcoides BAV1	675	695	0.97	583722	619704	0.94	10896	14484	0.75	11368	13180	0.86
Dehalococcoides CBDB1	708	749	0.95	613038	642825	0.95	13871	11266	1.23	11623	12147	0.96
Dehalococcoides ethenogenes 195	777	802	0.97	654247	666084	0.98	16797	10405	1.61	11391	15579	0.73
Deinococcus geothermalis DSM 11300	1190	1144	1.04	1144338	1085283	1.05	5355	7570	0.71	1529	2793	0.55
Deinococcus radiodurans	1358	1270	1.07	1229045	1159661	1.06	-10327	-9084	1.14	14711	15481	0.95
Desulfitobacterium hafniense Y51	1811	3248	0.56	1733976	3127152	0.55	41416	128766	0.32	18648	34940	0.53
Desulfotalea psychrophila LSv54	1556	1559	1	1513539	1501944	1.01	18119	35308	0.51	6272	-13378	-0.47
Desulfotomaculum reducens MI-1	1697	1578	1.08	1560339	1446687	1.08	72463	63386	1.14	46392	40749	1.14
Desulfovibrio desulfuricans G20	1965	1809	1.09	1781127	1594527	1.12	27037	19285	1.4	16536	13722	1.21
Desulfovibrio vulgaris DP4	1447	1493	0.97	1488822	1481205	1.01	-33268	-30417	1.09	6636	8254	0.8
Desulfovibrio vulgaris Hildenborough	1648	1730	0.95	1484884	1586490	0.94	-30259	-30850	0.98	7894	7210	1.09
Dichelobacter nodosus VCS1703A	614	665	0.92	610599	644637	0.95	16788	22602	0.74	-129	2424	-0.05
Ehrlichia canis Jake	427	497	0.86	469899	477378	0.98	26528	26119	1.02	18745	19185	0.98
Ehrlichia chaffeensis Arkansas	597	507	1.18	515869	419712	1.23	27572	22051	1.25	19296	14975	1.29
Ehrlichia ruminantium Gardel	453	496	0.91	471969	485580	0.97	23178	24102	0.96	17633	17298	1.02
Ehrlichia ruminantium str. Welgevonden	456	501	0.91	470022	487296	0.96	23245	24390	0.95	17633	17466	1.01
Ehrlichia ruminantium Welgevonden	415	472	0.88	460308	479293	0.96	22930	23895	0.96	17862	17867	1
Enterobacter 638	1968	2146	0.92	1928403	2039268	0.95	49023	51311	0.96	3174	-915	-3.47
Enterococcus faecalis V583	1453	1659	0.88	1279707	1492539	0.86	54964	58767	0.94	55571	64152	0.87
Erwinia carotovora atroseptica SCRI1043	2162	2309	0.94	2081934	2269638	0.92	59220	58494	1.01	2616	-3565	-0.73
Erythrobacter litoralis HTCC2594	1510	1500	1.01	1462428	1713660	0.85	8396	5029	1.67	20884	19251	1.08
Escherichia coli 536	2236	2392	0.93	2058129	2278917	0.9	56282	65613	0.86	579	-578	-1
Escherichia coli APEC O1	2125	2331	0.91	2133960	2307024	0.92	62653	65944	0.95	2282	3846	0.59
Escherichia coli CFT073	2632	2746	0.96	2301069	2441986	0.94	64120	68062	0.94	3107	-649	-4.79
Escherichia coli K12	2083	2183	0.95	2003951	2120358	0.95	55136	59686	0.92	1659	937	1.77
Escherichia coli O157H7	2641	2611	1.01	2400747	2350978	1.02	75982	71801	1.06	15469	13426	1.15
Escherichia coli O157H7 EDL933	2653	2670	0.99	2430931	2412118	1.01	73232	77300	0.95	15744	12249	1.29
Escherichia coli UTI89	2411	2632	0.92	2179011	2430303	0.9	61749	67451	0.92	148	1888	0.08
Escherichia coli W3110	2096	2129	0.98	1984755	2045200	0.97	57182	55759	1.03	295	858	0.34
Fervidobacterium nodosum Rt17-B1	820	929	0.88	810648	949749	0.85	44017	50835	0.87	52913	68382	0.77
Flavobacterium johnsoniae UW101	2418	2598	0.93	2604801	2718351	0.96	71981	97861	0.74	148166	143320	1.03
Flavobacterium psychrophilum JIP02 86	994	1417	0.7	1036857	1382661	0.75	18513	29307	0.63	76882	103582	0.74
Francisella tularensis FSC 198	884	720	1.23	818710	678970	1.21	37050	28319	1.31	26335	19820	1.33
Francisella tularensis holarctica	842	911	0.92	746863	813922	0.92	35704	32024	1.11	20974	25842	0.81
Francisella tularensis holarctica OSU18	771	783	0.98	686612	726587	0.94	33402	28498	1.17	19982	23943	0.83
Francisella tularensis novicida U112	837	881	0.95	827892	880410	0.94	33703	40463	0.83	27173	22815	1.19
Francisella tularensis tularensis	883	719	1.23	818041	675940	1.21	36969	28157	1.31	26439	19746	1.34
Francisella tularensis WY96-3418	776	857	0.91	735384	782696	0.94	32682	33763	0.97	19132	27347	0.7
Frankia alni ACN14a	3335	3375	0.99	3206382	3286617	0.98	-42909	-50422	0.85	-27171	-29029	0.94
Frankia CcI3	2183	2315	0.94	2202678	2414160	0.91	-11616	-12292	0.95	-22458	-23648	0.95
Fusobacterium nucleatum	963	1103	0.87	900105	1137205	0.79	62628	77700	0.81	74515	96185	0.77
Geobacillus kaustophilus HTA426	1668	1829	0.91	1434630	1580538	0.91	64346	72231	0.89	27962	27255	1.03
Geobacillus thermodenitrificans NG80-2	1584	1807	0.88	1392342	1580547	0.88	67220	76131	0.88	24008	22358	1.07
Geobacter metallireducens GS-15	1831	1687	1.09	1875834	1758045	1.07	-11510	-1995	5.77	30234	19720	1.53
Geobacter sulfurreducens	1700	1745	0.97	1657968	1766164	0.94	-6283	-9787	0.64	13059	17700	0.74
Geobacter uraniumreducens Rf4	1944	2412	0.81	2012332	2453411	0.82	16537	29283	0.56	52053	56840	0.92
Gloeobacter violaceus	2187	2242	0.98	2034294	2129670	0.96	-13478	-15057	0.9	-7142	-11497	0.62
Gluconobacter oxydans 621H	1234	1197	1.03	1271748	1163592	1.09	10091	-11563	-0.87	-18073	-9591	1.88
Gramella forsetii KT0803	1699	1884	0.9	1630581	1806324	0.9	80997	90474	0.9	66652	86380	0.77
Granulobacter bethesdensis CGDNIH1	1221	1215	1	1201407	1281639	0.94	28854	30145	0.96	-27417	-29628	0.93
Haemophilus ducreyi 35000HP	865	851	1.02	722292	723306	1	31220	14396	2.17	8652	20670	0.42
Haemophilus influenzae	829	827	1	765507	787718	0.97	24042	26695	0.9	13713	10370	1.32
Haemophilus influenzae 86 028NP	923	867	1.06	839607	821451	1.02	27945	25985	1.08	14804	14416	1.03
Haemophilus influenzae PittEE	812	810	1	737322	708666	1.04	23653	23785	0.99	10283	13593	0.76

Chromosome	Nu of genes on "+"-strand (Nu+)	Nu of genes on "-"-strand (Nu-)	Nu+/Nu-	Total length of genes on "+"-strand (Length+)	Total length of genes on "-"-strand (Length-)	Length+/ Length-	Cumulative skew (G-C) of genes on "+"-strand (G-C)+	Cumulative skew (G-C) of genes on "-"-strand (G-C)-	(G-C)+/ (G-C)-	Cumulative skew (A-T) of genes on "+"-strand (A-T)+	Cumulative skew (A-T) of genes on "-"-strand (A-T)-	(A-T)+/ (A-T)-
Haemophilus influenzae PittGG	786	883	0.89	1210530	744777	1.63	20969	27185	0.77	13032	11216	1.16
Haemophilus somnus 129PT	960	831	1.16	1097764	860415	1.28	38943	38147	1.02	18007	19110	0.94
Hahella chejuensis KCTC 2396	3252	3525	0.92	3059319	3314703	0.92	63669	74392	0.86	35922	39259	0.92
Haloquadratum walsbyi	1274	1335	0.95	1136961	1203585	0.94	30926	38895	0.8	18983	15906	1.19
Halorhodospira halophila SL1	935	1471	0.64	942603	1511805	0.62	-30187	43289	-0.7	14250	-11656	-1.22
Helicobacter acinonychis Sheeba	849	762	1.11	731304	662964	1.1	28075	28239	0.99	26011	19255	1.35
Helicobacter hepaticus	977	897	1.09	860805	816645	1.05	26490	35144	0.75	20877	16121	1.3
Helicobacter pylori 26695	769	806	0.95	723218	785704	0.92	25014	29898	0.84	24077	32840	0.73
Helicobacter pylori HPAG1	762	773	0.99	736164	729633	1.01	26775	26322	1.02	25561	26939	0.95
Helicobacter pylori J99	735	753	0.98	731070	755242	0.97	25674	28284	0.91	26578	29036	0.92
Herminiimonas arsenicoxydans	1664	1660	1	1519557	1497768	1.01	24595	27024	0.91	16290	16252	1
Hyperthermus butylicus	801	800	1	682629	671256	1.02	34803	33341	1.04	27444	22859	1.2
Hyphomonas neptunium ATCC 15444	1825	1679	1.09	1726909	1626375	1.06	-6865	-3370	2.04	-4193	-579	7.24
Idiomarina loihiensis L2TR	1188	1439	0.83	1207608	1409775	0.86	28560	63168	0.45	13276	10697	1.24
Jannaschia CCS1	2216	1995	1.11	2081103	1836687	1.13	48590	20332	2.39	-24885	-20071	1.24
Janthinobacterium Marseille	1754	1942	0.9	1738554	1935466	0.9	23385	26212	0.89	16269	20695	0.79
Kineococcus radiotolerans SRS30216	2274	2205	1.03	2176992	2143395	1.02	-67574	-59206	1.14	-18172	-20431	0.89
Klebsiella pneumoniae MGH 78578	2245	2530	0.89	2170731	2404155	0.9	33525	33197	1.01	-1738	-2650	0.66
Lactobacillus acidophilus NCFM	1018	843	1.21	951561	807381	1.18	42178	32836	1.28	22403	21807	1.03
Lactobacillus brevis ATCC 367	889	1295	0.69	795435	1135974	0.7	16916	42155	0.4	3401	7065	0.48
Lactobacillus casei ATCC 334	1269	1481	0.86	1099311	1286475	0.85	24512	25852	0.95	4249	9835	0.43
Lactobacillus delbrueckii bulgaricus	829	732	1.13	716511	653808	1.1	5997	5435	1.1	24388	25533	0.96
Lactobacillus delbrueckii bulgaricus ATCC BAA-365	896	824	1.09	746739	685950	1.09	6485	6014	1.08	25256	26216	0.96
Lactobacillus gasseri ATCC 33323	1003	751	1.34	954297	721821	1.32	40475	31932	1.27	28600	16387	1.75
Lactobacillus johnsonii NCC 533	1005	815	1.23	990477	788745	1.26	40671	32432	1.25	29868	21875	1.37
Lactobacillus plantarum	1491	1515	0.98	1379127	1390005	0.99	38907	40557	0.96	10300	10784	0.96
Lactobacillus reuteri F275	1023	876	1.17	937815	768195	1.22	32288	25261	1.28	20643	18734	1.1
Lactobacillus sakei 23K	973	905	1.08	827559	810039	1.02	15789	17734	0.89	23006	22035	1.04
Lactobacillus salivarius UCC118	899	817	1.1	782688	754656	1.04	45208	43139	1.05	36688	31015	1.18
Lactococcus lactis	1223	1097	1.11	1060725	954672	1.11	38217	32232	1.19	25854	22688	1.14
Lactococcus lactis cremoris MG1363	1137	1296	0.88	961656	1123817	0.86	34539	37159	0.93	24271	27583	0.88
Lactococcus lactis cremoris SK11	1173	1210	0.97	982920	998562	0.98	33607	32734	1.03	22679	22722	1
Lawsonia intracellularis PHE MN1-00	624	555	1.12	644656	576296	1.12	11601	10173	1.14	11223	8763	1.28
Legionella pneumophila Corby	1516	1689	0.9	1474716	1639479	0.9	24960	56503	0.44	36742	25018	1.47
Legionella pneumophila Lens	1498	1379	1.09	1535778	1379610	1.11	41577	35387	1.17	31560	22107	1.43
Legionella pneumophila Paris	1515	1511	1	1555596	1499007	1.04	39853	39504	1.01	31782	25677	1.24
Legionella pneumophila Philadelphia 1	1453	1488	0.98	1513428	1499931	1.01	39781	37382	1.06	28590	27923	1.02
Leifsonia xyli xyli CTCB0	1044	985	1.06	959730	853179	1.12	-14418	-13528	1.07	-1016	-1631	0.62
Leptospira borgpetersenii serovar Hardjo-bovis JB197	1332	1312	1.02	1313622	1292436	1.02	24767	29467	0.84	35907	37289	0.96
Leptospira borgpetersenii serovar Hardjo-bovis L550	1383	1319	1.05	1357107	1299018	1.04	30015	25407	1.18	39442	34399	1.15
Leptospira interrogans serovar Copenhageni	1637	1756	0.93	1549449	1652988	0.94	37856	42907	0.88	39393	37079	1.06
Leptospira interrogans serovar Lai	2264	2095	1.08	1799721	1588962	1.13	46216	37844	1.22	41701	37612	1.11
Leuconostoc mesenteroides ATCC 8293	1064	905	1.18	976617	826272	1.18	39398	33078	1.19	16981	20100	0.84
Listeria innocua	1426	1541	0.93	1291788	1385697	0.93	49161	56863	0.86	70573	70372	1
Listeria monocytogenes	1379	1466	0.94	1279194	1341507	0.95	48745	56045	0.87	64989	63036	1.03
Listeria monocytogenes 4b F2365	1403	1417	0.99	1271649	1297477	0.98	47681	53768	0.89	64680	58822	1.1
Listeria welshimeri serovar 6b SLCC5334	1377	1396	0.99	1241931	1248066	1	53754	55458	0.97	62263	55478	1.12
Magnetococcus MC-1	1816	1899	0.96	1891623	2190468	0.86	56751	84149	0.67	-16324	-26053	0.63
Magnetospirillum magneticum AMB-1	2467	2091	1.18	2444820	1943427	1.26	-33777	-22578	1.5	725	-6107	-0.12
Mannheimia succiniciproducens MBEL55E	1033	1346	0.77	888633	1190697	0.75	27759	37071	0.75	13008	14158	0.92
Maricaulis maris MCS10	1525	1537	0.99	1512120	1516920	1	-3579	-3018	1.19	-19069	-19990	0.95
Marinobacter aquaeolei VT8	1867	1990	0.94	1896474	2007294	0.94	14894	50852	0.29	20738	1094	18.96
Marinomonas MWYL1	2141	2297	0.93	2116020	2362065	0.9	65216	74818	0.87	-5638	-9229	0.61
Mesoplasma florum L1	327	354	0.92	355491	381246	0.93	15717	16826	0.93	31752	33268	0.95
Mesorhizobium BNC1	2028	2035	1	1967634	1979046	0.99	11075	17296	0.64	-10505	-7208	1.46
Mesorhizobium loti	3374	3368	1	3071463	3045498	1.01	-39124	-5777	6.77	2985	3061	0.98
Metallosphaera sedula DSM 5348	1228	1027	1.2	1064136	862377	1.23	62532	48150	1.3	38254	29439	1.3
Methylibium petroleiphilum PM1	1962	1856	1.06	1917531	1816227	1.06	-4920	-1140	4.32	7637	8271	0.92
Methylobacillus flagellatus KT	1248	1504	0.83	1232961	1447587	0.85	10342	13076	0.79	12413	15867	0.78
Methylococcus capsulatus Bath	1500	1455	1.03	1502319	1452361	1.03	-4180	744	-5.62	7055	4334	1.63
Moorella thermoacetica ATCC 39073	1175	1289	0.91	1088823	1184955	0.92	20994	18338	1.14	11288	8435	1.34
Mycobacterium avium 104	2463	2656	0.93	2319135	2533977	0.92	24	-3882	-0.01	7625	13049	0.58
Mycobacterium avium paratuberculosis	2227	2122	1.05	2261952	2159130	1.05	-3175	631	-5.03	8785	5145	1.71
Mycobacterium bovis	1967	1952	1.01	1932183	2002680	0.96	25756	31274	0.82	-10949	-9912	1.1
Mycobacterium bovis BCG Pasteur 1173P2	1982	1969	1.01	1951533	2020545	0.97	25776	32760	0.79	-10615	-10137	1.05
Mycobacterium gilvum PYR-GCK	2472	2768	0.89	2504979	2694450	0.93	-7640	-7633	1	4837	8101	0.6

Chromosome	Nu of genes on "+"-strand (Nu+)	Nu of genes on "-"-strand (Nu-)	Nu+/Nu-	Total length of genes on "+"-strand (Length+)	Total length of genes on "-"-strand (Length-)	Length+/ Length-	Cumulative skew (G-C) of genes on "+"-strand (G-C)+	Cumulative skew (G-C) of genes on "-"-strand (G-C)-	(G-C)+/ (G-C)-	Cumulative skew (A-T) of genes on "+"-strand (A-T)+	Cumulative skew (A-T) of genes on "-"-strand (A-T)-	(A-T)+/ (A-T)-
Mycobacterium JLS	2895	2843	1.02	2792079	2828919	0.99	-13527	-8400	1.61	9678	8705	1.11
Mycobacterium KMS	2790	2669	1.05	2689011	2652111	1.01	-13072	-11168	1.17	10283	10007	1.03
Mycobacterium leprae	819	785	1.04	851502	769488	1.11	22720	13975	1.63	-11700	-5921	1.98
Mycobacterium MCS	2734	2656	1.03	2658444	2654727	1	-12617	-11109	1.14	9523	10180	0.94
Mycobacterium smegmatis MC2 155	3453	3262	1.06	3290043	3083577	1.07	-20142	-16268	1.24	11859	9459	1.25
Mycobacterium tuberculosis CDC1551	2078	2110	0.98	1958775	2034327	0.96	23963	31589	0.76	-10991	-10005	1.1
Mycobacterium tuberculosis F11	1965	1975	0.99	1966647	2063673	0.95	25893	30795	0.84	-10222	-9672	1.06
Mycobacterium tuberculosis H37Ra	2021	2012	1	1970070	2063652	0.95	25026	30680	0.82	-10776	-10136	1.06
Mycobacterium tuberculosis H37Rv	2008	1980	1.01	1969695	2050692	0.96	25731	30572	0.84	-10064	-9902	1.02
Mycobacterium ulcerans Agy99	2076	2083	1	2018910	2055579	0.98	8269	14919	0.55	931	-6588	-0.14
Mycobacterium vanbaalenii PYR-1	3082	2896	1.06	3012201	2928924	1.03	992	3792	0.26	8369	4494	1.86
Mycoplasma agalactiae PG2	355	386	0.92	357411	409362	0.87	8612	14373	0.6	25411	31501	0.81
Mycoplasma capricolum ATCC 27343	411	400	1.03	452688	439146	1.03	16848	15360	1.1	33836	31748	1.07
Mycoplasma gallisepticum	384	341	1.13	471168	403578	1.17	5923	6794	0.87	28161	23926	1.18
Mycoplasma genitalium	277	199	1.39	293202	234606	1.25	5601	5075	1.1	12557	8627	1.46
Mycoplasma hyopneumoniae 232	337	353	0.95	365442	441228	0.83	4413	4258	1.04	21537	24936	0.86
Mycoplasma hyopneumoniae 7448	323	339	0.95	375513	415809	0.9	4322	4623	0.93	18939	26138	0.72
Mycoplasma hyopneumoniae J	322	342	0.94	372150	414855	0.9	4524	4202	1.08	19780	26227	0.75
Mycoplasma mobile 163K	322	310	1.04	356823	348918	1.02	11653	11922	0.98	23218	24186	0.96
Mycoplasma mycoides	469	546	0.86	467316	524289	0.89	16466	17829	0.92	33638	42216	0.8
Mycoplasma penetrans	504	532	0.95	585267	620100	0.94	19021	18593	1.02	38730	40267	0.96
Mycoplasma pneumoniae	405	283	1.43	418764	301728	1.39	3176	2699	1.18	18442	9619	1.92
Mycoplasma pulmonis	406	375	1.08	467112	404454	1.15	7686	7320	1.05	37334	31818	1.17
Mycoplasma synoviae 53	266	405	0.66	292029	421212	0.69	3834	3655	1.05	22703	33521	0.68
Myxococcus xanthus DK 1622	3592	3738	0.96	4105863	4254607	0.97	59692	70471	0.85	-473	-8157	0.06
Natronomonas pharaonis	1393	1267	1.1	1230963	1120050	1.1	2697	1526	1.77	29394	30888	0.95
Neisseria gonorrhoeae FA 1090	939	1062	0.88	801069	891201	0.9	-4282	-8339	0.51	28083	30388	0.92
Neisseria meningitidis FAM18	961	955	1.01	874279	885500	0.99	-3214	-11122	0.29	30721	29583	1.04
Neisseria meningitidis MC58	1098	964	1.14	941061	855111	1.1	-4049	-10970	0.37	31262	33869	0.92
Neisseria meningitidis Z2491	924	1124	0.82	795535	959805	0.83	-6509	-7591	0.86	29756	31160	0.95
Neorickettsia sennetsu Miyayama	476	455	1.05	375280	377547	0.99	19278	20790	0.93	1505	3591	0.42
Nitratiruptor SB155-2	896	946	0.95	851325	914958	0.93	30324	32432	0.94	51775	52488	0.99
Nitrobacter hamburgensis X14	1882	1921	0.98	1759290	1786497	0.98	5170	4694	1.1	8756	8095	1.08
Nitrobacter winogradskyi Nb-255	1518	1603	0.95	1388073	1474794	0.94	4420	9827	0.45	5689	2077	2.74
Nitrosococcus oceani ATCC 19707	1453	1520	0.96	1411677	1539477	0.92	33887	37857	0.9	-9544	-18680	0.51
Nitrosomonas europaea	1148	1312	0.88	1112361	1293264	0.86	15082	37490	0.4	12693	4862	2.61
Nitrosomonas eutropha C71	1216	1227	0.99	1147611	1130535	1.02	34084	31016	1.1	4753	4865	0.98
Nitrosospira multiformis ATCC 25196	1337	1419	0.94	1287501	1434126	0.9	33546	35223	0.95	14275	12679	1.13
Nocardia farcinica IFM10152	2875	2807	1.02	2736801	2708856	1.01	-34869	-37504	0.93	3530	3090	1.14
Nocardioides JS614	2441	2203	1.11	2414070	2136339	1.13	-31919	-33002	0.97	-2157	1629	-1.32
Nostoc sp	2700	2665	1.01	2621445	2670921	0.98	42591	37928	1.12	43280	48431	0.89
Novosphingobium aromaticivorans DSM 12444	1677	1646	1.02	1627338	1635924	0.99	-10680	-10593	1.01	5890	2281	2.58
Oceanobacillus iheyensis	1743	1756	0.99	1489215	1575180	0.95	81201	84997	0.96	73580	77559	0.95
Ochrobactrum anthropi ATCC 49188	1483	1247	1.19	1361959	1131769	1.2	25458	-1638	-15.54	-14579	-2118	6.88
Oenococcus oeni PSU-1	882	808	1.09	766050	702639	1.09	23561	20941	1.13	5183	512	10.12
Onion yellows phytoplasma	469	284	1.65	391914	234873	1.67	-5132	-5514	0.93	25258	20190	1.25
Orientia tsutsugamushi Boryong	542	639	0.85	492429	545529	0.9	18320	21000	0.87	25311	27539	0.92
Parabacteroides distasonis ATCC 8503	2070	1779	1.16	2305068	2037102	1.13	108102	99570	1.09	34970	18456	1.89
Parachlamydia sp UWE25	1044	986	1.06	971409	1016631	0.96	13317	10786	1.23	13428	16655	0.81
Paracoccus denitrificans PD1222	865	796	1.09	793251	773499	1.03	8549	5618	1.52	-5820	-5555	1.05
Paracoccus denitrificans PD1222	1460	1338	1.09	1339509	1240449	1.08	9584	10910	0.88	-2989	-7315	0.41
Parvibaculum lavamentivorans DS-1	2019	1616	1.25	1997946	1531062	1.3	12492	-11154	-1.12	-5150	15848	-0.32
Pasteurella multocida	1050	964	1.09	1054581	953850	1.11	36930	27022	1.37	12223	10228	1.2
Pediococcus pentosaceus ATCC 25745	829	925	0.9	781974	828756	0.94	32295	32307	1	21907	23951	0.91
Pelobacter carbinolicus	1597	1754	0.91	1515183	1682487	0.9	19279	14511	1.33	-9916	-8310	1.19
Pelobacter propionicus DSM 2379	1786	1789	1	1750755	1780299	0.98	-1514	-2541	0.6	33619	26872	1.25
Pelodictyon luteolum DSM 273	1123	959	1.17	1170336	936546	1.25	-18312	-13649	1.34	24240	13077	1.85
Pelotomaculum thermopropionicum SI	1537	1382	1.11	1399362	1214223	1.15	61412	55272	1.11	39476	31679	1.25
Photobacterium profundum SS9	1797	1618	1.11	1756134	1601799	1.1	54015	48587	1.11	10129	14085	0.72
Photorhabdus luminescens	2137	2545	0.84	2030145	2583510	0.79	64783	91921	0.7	14472	17713	0.82
Picrophilus torridus DSM 9790	768	766	1	698322	719496	0.97	28526	31970	0.89	49752	50542	0.98
Pirellula sp	3627	3697	0.98	3496716	3443526	1.02	16615	45783	0.36	41125	21093	1.95
Polaromonas JS666	2434	2382	1.02	2304888	2268342	1.02	3342	1824	1.83	12702	5262	2.41
Polaromonas naphthalenivorans CJ2	2108	1975	1.07	2035320	1942212	1.05	-8619	-5143	1.68	21011	16951	1.24
Polynucleobacter QLW-P1DMWA-1	1017	1059	0.96	946077	1058436	0.89	23813	28344	0.84	-8248	-10982	0.75
Porphyromonas gingivalis W83	921	987	0.93	971364	963249	1.01	14618	11189	1.31	20182	19058	1.06

Chromosome	Nu of genes on "+"-strand (Nu+)	Nu of genes on "-"-strand (Nu-)	Nu+/Nu-	Total length of genes on "+"-strand (Length+)	Total length of genes on "-"-strand (Length-)	Length+/ Length-	Cumulative skew (G-C) of genes on "+"-strand (G-C)+	Cumulative skew (G-C) of genes on "-"-strand (G-C)-	(G-C)+/ (G-C)-	Cumulative skew (A-T) of genes on "+"-strand (A-T)+	Cumulative skew (A-T) of genes on "-"-strand (A-T)-	(A-T)+/ (A-T)-
Prochlorococcus marinus AS9601	939	981	0.96	746001	771141	0.97	35195	34145	1.03	39930	42546	0.94
Prochlorococcus marinus CCMP1375	952	930	1.02	791190	763905	1.04	31669	31196	1.02	23829	22465	1.06
Prochlorococcus marinus MED4	830	886	0.94	699154	759999	0.92	33221	34994	0.95	35884	39863	0.9
Prochlorococcus marinus MIT 9301	941	965	0.98	730239	763158	0.96	33940	34365	0.99	37667	42635	0.88
Prochlorococcus marinus MIT 9303	1553	1443	1.08	1154943	1121232	1.03	20495	17658	1.16	-12236	-3054	4.01
Prochlorococcus marinus MIT 9312	882	927	0.95	749241	775320	0.97	34877	35273	0.99	39088	41249	0.95
Prochlorococcus marinus MIT 9515	936	969	0.97	725715	784161	0.93	33371	35494	0.94	39294	40133	0.98
Prochlorococcus marinus MIT9313	1210	1058	1.14	1043002	932112	1.12	-397	32610	-0.01	7682	-31074	-0.25
Prochlorococcus marinus NATL1A	1081	1111	0.97	822747	801726	1.03	35809	33911	1.06	30386	31051	0.98
Prochlorococcus marinus NATL2A	940	951	0.99	801318	768867	1.04	34643	33547	1.03	29511	29078	1.01
Propionibacterium acnes KPA171202	1143	1153	0.99	1169265	1125294	1.04	-6193	-5516	1.12	-21102	-19166	1.1
Prosthecochloris vibrioformis DSM 265	908	844	1.08	938376	855204	1.1	3649	7004	0.52	12907	978	13.2
Pseudoalteromonas atlantica T6c	2083	2197	0.95	2182518	2298606	0.95	48668	52861	0.92	36418	40741	0.89
Pseudoalteromonas haloplanktis TAC125	1464	1475	0.99	1386838	1418196	0.98	35863	34449	1.04	36168	39243	0.92
Pseudomonas aeruginosa	2744	2823	0.97	2707389	2893492	0.94	-74444	-43388	1.72	31439	17547	1.79
Pseudomonas aeruginosa PA7	3113	3172	0.98	2950731	2951760	1	-59466	-57159	1.04	31249	26775	1.17
Pseudomonas aeruginosa UCBPP-PA14	2902	2989	0.97	2913093	2932359	0.99	-61228	-62046	0.99	28718	25190	1.14
Pseudomonas entomophila L48	2511	2622	0.96	2537740	2693748	0.94	-29177	-32934	0.89	25394	27202	0.93
Pseudomonas fluorescens Pf-5	3199	2938	1.09	3357286	2924028	1.15	-52086	-41001	1.27	33931	19467	1.74
Pseudomonas fluorescens PfO-1	2931	2804	1.05	3004806	2786928	1.08	-9185	-3226	2.85	31651	22162	1.43
Pseudomonas mendocina ymp	2260	2333	0.97	2233191	2306133	0.97	-32225	-29193	1.1	12616	15162	0.83
Pseudomonas putida F1	2554	2697	0.95	2585157	2703708	0.96	-8986	-2690	3.34	18615	16946	1.1
Pseudomonas putida KT2440	2755	2594	1.06	2738304	2626359	1.04	2925	-16910	-0.17	16479	23717	0.69
Pseudomonas stutzeri A1501	2096	2031	1.03	2072937	2014344	1.03	-13878	-14958	0.93	10159	10268	0.99
Pseudomonas syringae phaseolicola 1448A	2487	2496	1	2542551	2447314	1.04	21231	14646	1.45	2352	11387	0.21
Pseudomonas syringae pv B728a	2536	2552	0.99	2742852	2602968	1.05	8929	12558	0.71	10521	14746	0.71
Pseudomonas syringae tomato DC3000	2848	2620	1.09	2873082	2572626	1.12	13724	16123	0.85	15114	14193	1.06
Psychrobacter arcticum 273-4	1135	984	1.15	1113333	1024353	1.09	31795	29945	1.06	6962	9378	0.74
Psychrobacter cryohalolentis K5	1225	1241	0.99	1258215	1286847	0.98	38286	35801	1.07	4661	8812	0.53
Psychrobacter PRwf-1	1177	1192	0.99	1203867	1270776	0.95	22915	26744	0.86	11410	16702	0.68
Psychromonas ingrahamii 37	1777	1767	1.01	1802280	1738260	1.04	57337	56014	1.02	16161	15640	1.03
Pyrobaculum aerophilum	1361	1243	1.09	1060080	914682	1.16	70494	60443	1.17	30898	23775	1.3
Pyrobaculum arsenaticum DSM 13514	1141	1156	0.99	895512	943932	0.95	52813	55864	0.95	23517	24040	0.98
Pyrobaculum calidifontis JCM 11548	1058	1090	0.97	906867	915885	0.99	56010	57275	0.98	20405	17420	1.17
Pyrobaculum islandicum DSM 4184	1007	970	1.04	813576	778761	1.04	47501	45479	1.04	28317	27558	1.03
Ralstonia eutropha H16	1771	1879	0.94	1716039	1844934	0.93	1716	-1074	-1.6	8591	15056	0.57
Ralstonia eutropha JMP134	1680	1758	0.96	1640421	1744488	0.94	5006	7034	0.71	6819	11854	0.58
Ralstonia metallidurans CH34	1798	1802	1	1754907	1762362	1	11883	11682	1.02	2110	12896	0.16
Ralstonia solanacearum	1693	1746	0.97	1633194	1636263	1	-259	-12652	0.02	11343	17133	0.66
Rhizobium etli CFN 42	2069	1965	1.05	1935261	1839546	1.05	-42105	-12337	3.41	32	-2003	-0.02
Rhizobium leguminosarum bv viciae 3841	2326	2373	0.98	2155470	2836002	0.76	-49308	-13119	3.76	2248	-1603	-1.4
Rhizobium leguminosarum bv viciae 3841	378	401	0.94	384327	401688	0.96	-2701	-5620	0.48	-2194	-1200	1.83
Rhodobacter sphaeroides 2 4 1	1443	1578	0.91	1339287	1500777	0.89	1117	10866	0.1	-11408	-10460	1.09
Rhodobacter sphaeroides ATCC 17025	1628	1482	1.1	1496769	1392282	1.08	19955	7742	2.58	-11600	-6840	1.7
Rhodococcus RHA1	564	581	0.97	468192	454215	1.03	-11568	-11541	1	5614	6164	0.91
Rhodococcus RHA1	3670	3540	1.04	3599343	3519612	1.02	-40157	-51843	0.77	7198	15255	0.47
Rhodoferax ferrireducens T118	2078	2091	0.99	2055714	2177889	0.94	23086	21191	1.09	-8700	-3398	2.56
Rhodopseudomonas palustris BisA53	2391	2486	0.96	2357655	2367048	1	-331	-738	0.45	6106	9388	0.65
Rhodopseudomonas palustris BisB18	2391	2494	0.96	2334594	2382678	0.98	-1713	4338	-0.39	4117	2752	1.5
Rhodopseudomonas palustris BisB5	2199	2197	1	2127360	2122683	1	5103	-4688	-1.09	4423	7201	0.61
Rhodopseudomonas palustris CGA009	2399	2413	0.99	2397647	2358891	1.02	-3434	-10241	0.34	2175	5228	0.42
Rhodopseudomonas palustris HaA2	2367	2315	1.02	2385609	2268375	1.05	-11202	-5814	1.93	9587	7967	1.2
Rhodospirillum rubrum ATCC 11170	1879	1911	0.98	1927200	1919298	1	-27832	-27741	1	-40440	-39981	1.01
Rickettsia bellii RML369-C	653	775	0.84	593040	703219	0.84	21825	29608	0.74	30225	39992	0.76
Rickettsia conorii	654	719	0.91	524469	500110	1.05	20139	18029	1.12	23068	25340	0.91
Rickettsia felis URRWXCal2	724	675	1.07	655375	588741	1.11	26141	22454	1.16	36453	29877	1.22
Rickettsia prowazekii	428	406	1.05	446032	393163	1.13	17926	15252	1.18	15967	18118	0.88
Rickettsia typhi wilmington	390	447	0.87	400013	438714	0.91	14654	18491	0.79	16982	18195	0.93
Roseiflexus RS-1	2286	2230	1.03	2543325	2394080	1.06	42004	43213	0.97	-37132	-36232	1.02
Roseobacter denitrificans OCh 114	1941	2004	0.97	1784523	1923531	0.93	49910	49261	1.01	-10113	-12096	0.84
Rubrobacter xylanophilus DSM 9941	1461	1678	0.87	1382742	1581945	0.87	49292	54763	0.9	-356	-1056	0.34
Saccharophagus degradans 2-40	1962	2045	0.96	2173779	2211288	0.98	41642	42932	0.97	55807	55448	1.01
Saccharopolyspora erythraea NRRL 2338	3533	3663	0.96	3388608	3586293	0.94	14989	23810	0.63	1344	1435	0.94
Salinibacter ruber DSM 13855	1444	1356	1.06	1525134	1489759	1.02	-6602	-4205	1.57	20522	16257	1.26
Salinispora tropica CNB-440	2272	2263	1	2269875	2305092	0.98	2544	-1156	-2.2	-15937	-17712	0.9
Salmonella enterica Choleraesuis	2146	2280	0.94	1941429	2046294	0.95	57223	57725	0.99	-140	-1643	0.09

Chromosome	Nu of genes on "+"-strand (Nu+)	Nu of genes on "-"-strand (Nu-)	Nu+/Nu-	Total length of genes on "+"-strand (Length+)	Total length of genes on "-"-strand (Length-)	Length+/ Length-	Cumulative skew (G-C) of genes on "+"-strand (G-C)+	Cumulative skew (G-C) of genes on "-"-strand (G-C)-	(G-C)+/ (G-C)-	Cumulative skew (A-T) of genes on "+"-strand (A-T)+	Cumulative skew (A-T) of genes on "-"-strand (A-T)-	(A-T)+/ (A-T)-
Salmonella enterica Paratypi ATCC 9150	2009	2083	0.96	1876263	1908145	0.98	52580	59306	0.89	-5205	-238	21.87
Salmonella typhi	2213	2181	1.01	2013450	1985947	1.01	61857	58745	1.05	-1842	-523	3.52
Salmonella typhi Ty2	2242	2075	1.08	2020437	1928590	1.05	62573	56108	1.12	-4088	607	-6.73
Salmonella typhimurium LT2	2114	2310	0.92	2016987	2181471	0.92	60923	62392	0.98	-1124	-153	7.35
Shewanella amazonensis SB2B	1927	1717	1.12	2003403	1789530	1.12	23575	18349	1.28	4284	3197	1.34
Shewanella ANA-3	2114	1996	1.06	2199138	2066256	1.06	29792	27862	1.07	19058	14386	1.32
Shewanella baltica OS155	2180	2126	1.03	2149503	2125989	1.01	36282	38446	0.94	19741	18545	1.06
Shewanella baltica OS185	2222	2100	1.06	2219658	2159166	1.03	37129	38045	0.98	19094	12872	1.48
Shewanella denitrificans OS217	1848	1905	0.97	1895736	1945230	0.97	30144	35124	0.86	28430	22674	1.25
Shewanella frigidimarina NCIMB 400	2079	1949	1.07	2036697	2041419	1	49124	47194	1.04	14361	15613	0.92
Shewanella loihica PV-4	2046	1812	1.13	2069979	1875183	1.1	13801	13634	1.01	30712	30563	1
Shewanella MR-4	2027	1896	1.07	2080710	1955283	1.06	28642	25926	1.1	14562	14347	1.01
Shewanella MR-7	2096	1909	1.1	2108892	1991097	1.06	25364	30022	0.84	20432	13011	1.57
Shewanella oneidensis	2247	2070	1.09	2158233	1985256	1.09	38191	35239	1.08	16853	13606	1.24
Shewanella putrefaciens CN-32	1913	2058	0.93	1911531	2070705	0.92	40073	46201	0.87	6918	10078	0.69
Shewanella W3-18-1	2042	2001	1.02	2061192	1953942	1.05	45717	41253	1.11	11413	10317	1.11
Shigella boydii Sb227	2052	2083	0.99	1841073	1798761	1.02	56240	53075	1.06	5973	7668	0.78
Shigella dysenteriae	2017	2256	0.89	1571823	1802586	0.87	41987	49855	0.84	7760	9533	0.81
Shigella flexneri 2a	2058	2123	0.97	1844040	1850103	1	49980	57295	0.87	6780	3894	1.74
Shigella flexneri 2a 2457T	2027	2040	0.99	1774662	1792087	0.99	50892	52982	0.96	6713	2000	3.36
Shigella flexneri 5 8401	2000	2115	0.95	1811256	1886034	0.96	51849	56312	0.92	7883	3812	2.07
Shigella sonnei Ss046	2074	2148	0.97	1900659	1983813	0.96	57278	57020	1	9131	8519	1.07
Silicibacter pomeroyi DSS-3	1904	1905	1	1841121	1849431	1	36797	29358	1.25	-11462	-12091	0.95
Silicibacter TM1040	1513	1516	1	1467963	1381647	1.06	22654	22594	1	-8821	-8147	1.08
Sinorhizobium medicae WSM419	1758	1770	0.99	1614114	1681361	0.96	-6694	-984	6.8	-1014	2944	-0.34
Sinorhizobium meliloti	1720	1620	1.06	1609608	1533732	1.05	-17392	-8624	2.02	4474	4158	1.08
Sodalis glossinidius morsitans	1267	1164	1.09	1146249	977505	1.17	27640	16651	1.66	3789	-996	-3.8
Solibacter usitatus Ellin6076	3930	3895	1.01	4560393	4454151	1.02	-19878	-4722	4.21	27975	32913	0.85
Sphingomonas wittichii RW1	2471	2378	1.04	2519400	2427924	1.04	-24045	-25020	0.96	-751	3432	-0.22
Sphingopyxis alaskensis RB2256	1470	1694	0.87	1421724	1605915	0.89	3764	6165	0.61	3652	-1008	-3.62
Staphylococcus aureus aureus MRSA252	1286	1369	0.94	1164982	1208196	0.96	51116	51249	1	62189	72171	0.86
Staphylococcus aureus aureus MSSA476	1301	1277	1.02	1181389	1113231	1.06	53285	47523	1.12	64405	60422	1.07
Staphylococcus aureus COL	1328	1286	1.03	1176919	1146046	1.03	53091	47587	1.12	65921	65992	1
Staphylococcus aureus JH1	1427	1319	1.08	1265095	1167948	1.08	57091	48162	1.19	73857	69666	1.06
Staphylococcus aureus JH9	1395	1301	1.07	1256796	1166961	1.08	56840	48185	1.18	73470	69908	1.05
Staphylococcus aureus Mu50	1330	1366	0.97	1204518	1207461	1	53068	49520	1.07	66738	71531	0.93
Staphylococcus aureus MW2	1289	1342	0.96	1165464	1194336	0.98	51470	50136	1.03	62380	72212	0.86
Staphylococcus aureus N315	1249	1338	0.93	1163031	1186827	0.98	50798	49376	1.03	62253	70403	0.88
Staphylococcus aureus NCTC 8325	1355	1536	0.88	1156293	1243329	0.93	49787	53331	0.93	59972	75706	0.79
Staphylococcus aureus Newman	1313	1300	1.01	1213944	1187649	1.02	54232	50231	1.08	68080	71468	0.95
Staphylococcus aureus RF122	1225	1289	0.95	1119297	1096107	1.02	48955	46891	1.04	58216	58074	1
Staphylococcus aureus USA300	1235	1324	0.93	1156437	1201836	0.96	51598	51388	1	63063	72194	0.87
Staphylococcus epidermidis ATCC 12228	1094	1324	0.83	943803	1149093	0.82	36018	41553	0.87	47891	68608	0.7
Staphylococcus epidermidis RP62A	1099	1394	0.79	948763	1212369	0.78	35843	46080	0.78	47995	76319	0.63
Staphylococcus haemolyticus	1424	1251	1.14	1224741	1084719	1.13	44954	40965	1.1	68477	59526	1.15
Staphylococcus saprophyticus	1289	1156	1.12	1093275	1013502	1.08	45404	41411	1.1	56195	51963	1.08
Staphylothermus marinus F1	949	620	1.53	820698	548268	1.5	45549	31238	1.46	57333	36184	1.58
Streptococcus agalactiae 2603	1016	1107	0.92	901185	967563	0.93	31754	36853	0.86	21949	17788	1.23
Streptococcus agalactiae A909	1012	983	1.03	941242	892155	1.06	33399	32474	1.03	23948	15259	1.57
Streptococcus agalactiae NEM316	1011	1082	0.93	941556	997512	0.94	32380	36368	0.89	23182	20924	1.11
Streptococcus mutans	942	1017	0.93	843081	901131	0.94	29642	33980	0.87	-3449	-189	18.25
Streptococcus pneumoniae D39	985	928	1.06	902256	809286	1.11	29426	27179	1.08	12352	10173	1.21
Streptococcus pneumoniae R6	1029	1013	1.02	923559	849387	1.09	30170	27755	1.09	12993	11256	1.15
Streptococcus pneumoniae TIGR4	1080	1024	1.05	937516	866001	1.08	30616	26279	1.17	14329	9090	1.58
Streptococcus pyogenes M1 GAS	948	748	1.27	847410	704013	1.2	23382	19688	1.19	14912	10487	1.42
Streptococcus pyogenes Manfredo	899	845	1.06	779976	760798	1.03	21596	21258	1.02	12386	13793	0.9
Streptococcus pyogenes MGAS10270	1049	936	1.12	886497	799287	1.11	24496	23585	1.04	16105	15018	1.07
Streptococcus pyogenes MGAS10394	968	917	1.06	840144	817176	1.03	21614	25208	0.86	12356	17846	0.69
Streptococcus pyogenes MGAS10750	1112	866	1.28	935775	757767	1.23	28204	20941	1.35	20889	13096	1.6
Streptococcus pyogenes MGAS2096	1002	895	1.12	847038	777837	1.09	23396	23850	0.98	13046	14557	0.9
Streptococcus pyogenes MGAS315	905	959	0.94	808719	820446	0.99	20818	26081	0.8	11779	19275	0.61
Streptococcus pyogenes MGAS5005	939	925	1.02	811470	781587	1.04	20964	23237	0.9	10424	13738	0.76
Streptococcus pyogenes MGAS6180	942	951	0.99	828714	823110	1.01	21740	25418	0.86	11670	18846	0.62
Streptococcus pyogenes MGAS8232	961	883	1.09	844200	770115	1.1	22369	22792	0.98	14387	16139	0.89
Streptococcus pyogenes MGAS9429	1038	838	1.24	877512	734238	1.2	25002	20495	1.22	15934	11795	1.35
Streptococcus pyogenes SSI-1	1031	829	1.24	867444	740106	1.17	26379	20017	1.32	17731	12653	1.4

Chromosome	Nu of genes on "+"-strand (Nu+)	Nu of genes on "-"-strand (Nu-)	Nu+/Nu-	Total length of genes on "+"-strand (Length+)	Total length of genes on "-"-strand (Length-)	Length+/ Length-	Cumulative skew (G-C) of genes on "+"-strand (G-C)+	Cumulative skew (G-C) of genes on "-"-strand (G-C)-	(G-C)+/ (G-C)-	Cumulative skew (A-T) of genes on "+"-strand (A-T)+	Cumulative skew (A-T) of genes on "-"-strand (A-T)-	(A-T)+/ (A-T)-
Streptococcus sanguinis SK36	1126	1143	0.99	1071483	1047747	1.02	36123	36738	0.98	8306	8103	1.03
Streptococcus suis 05ZYH33	992	1193	0.83	834138	1004478	0.83	21456	38851	0.55	14448	12419	1.16
Streptococcus suis 98HAH33	999	1185	0.84	831969	1006059	0.83	21338	38707	0.55	14327	12726	1.13
Streptococcus thermophilus CNRZ1066	976	938	1.04	763245	745320	1.02	23279	22309	1.04	2642	-1915	-1.38
Streptococcus thermophilus LMD-9	852	857	0.99	716883	710928	1.01	20928	22046	0.95	5161	-2798	-1.84
Streptococcus thermophilus LMG 18311	959	929	1.03	764568	745308	1.03	23492	22538	1.04	2890	-1986	-1.46
Streptomyces avermitilis	3696	3880	0.95	3681126	4105308	0.9	-69240	-69995	0.99	30868	20861	1.48
Streptomyces coelicolor	3970	3798	1.05	3980317	3724768	1.07	-87855	-96824	0.91	21504	30147	0.71
Sulfurovum NBC37-1	1237	1200	1.03	1150422	1159485	0.99	31168	30210	1.03	61194	65553	0.93
Symbiobacterium thermophilum IAM14863	1652	1685	0.98	1532766	1577715	0.97	33990	36550	0.93	170	-1535	-0.11
Synechococcus CC9311	1403	1488	0.94	1107229	1164615	0.95	16422	19240	0.85	-18168	-16777	1.08
Synechococcus CC9605	1321	1323	1	1114740	1060341	1.05	9056	6263	1.45	-10196	-9070	1.12
Synechococcus CC9902	1217	1089	1.12	1032444	971325	1.06	14263	16364	0.87	-15449	-13275	1.16
Synechococcus elongatus PCC 6301	1320	1206	1.09	1204560	1536236	0.78	-799	-722	1.11	-19335	-17847	1.08
Synechococcus elongatus PCC 7942	1277	1334	0.96	1216314	1183209	1.03	-591	-1891	0.31	-17837	-19852	0.9
Synechococcus RCC307	1286	1248	1.03	1052898	1051128	1	7108	7785	0.91	-15720	-16721	0.94
Synechococcus sp WH8102	1343	1175	1.14	1170697	1022727	1.14	14011	10914	1.28	-14481	-9247	1.57
Synechococcus WH 7803	1235	1297	0.95	1080579	1123026	0.96	4987	5639	0.88	-20782	-16437	1.26
Synechocystis PCC6803	1661	1510	1.1	1944470	1481478	1.31	20127	18499	1.09	-4771	-1885	2.53
Syntrophobacter fumaroxidans MPOB	2065	1998	1.03	2070891	2032128	1.02	34427	31240	1.1	30792	20272	1.52
Syntrophomonas wolfei Goettingen	1191	1312	0.91	1170735	1260153	0.93	56586	66857	0.85	35335	35596	0.99
Syntrophus aciditrophicus SB	1709	1458	1.17	1528740	1302873	1.17	36898	27908	1.32	25198	23087	1.09
Thermoanaerobacter tengcongensis	1252	1335	0.94	1152054	1191060	0.97	95116	96114	0.99	85368	89624	0.95
Thermobifida fusca YX	1547	1562	0.99	1526346	1574361	0.97	-35851	-41163	0.87	14339	14062	1.02
Thermosipho melanesiensis BI429	907	971	0.93	830145	921405	0.9	61688	64900	0.95	57877	65655	0.88
Thermosynechococcus elongatus	1275	1200	1.06	1171218	1159478	1.01	-3465	-4514	0.77	-18649	-20103	0.93
Thermotoga maritima	1009	848	1.19	960296	798652	1.2	50636	38898	1.3	54899	52291	1.05
Thermotoga petrophila RKU-1	830	954	0.87	798543	928083	0.86	38350	51096	0.75	53055	53539	0.99
Thiobacillus denitrificans ATCC 25259	1373	1453	0.94	1313535	1379034	0.95	-26451	-27488	0.96	14196	13490	1.05
Thiomicrospira crunogena XCL-2	1217	978	1.24	1232322	949371	1.3	44595	33333	1.34	20401	14478	1.41
Thiomicrospira denitrificans ATCC 33889	1099	997	1.1	1062231	982374	1.08	49239	44727	1.1	62500	51591	1.21
Treponema denticola ATCC 35405	1314	1452	0.9	1240131	1365225	0.91	33768	42653	0.79	57519	60926	0.94
Treponema pallidum	556	479	1.16	563057	493838	1.14	26632	23243	1.15	-16445	-15036	1.09
Trichodesmium erythraeum IMS101	2212	2238	0.99	2295897	2347683	0.98	97391	96188	1.01	70094	72659	0.96
Tropheryma whipplei TW08 27	414	368	1.12	397517	385231	1.03	14403	12468	1.16	-9936	-3986	2.49
Tropheryma whipplei Twist	378	429	0.88	383439	415290	0.92	13914	15064	0.92	-6005	-9412	0.64
Ureaplasma urealyticum	330	283	1.17	362580	323832	1.12	10156	7953	1.28	19942	19421	1.03
Verminephrobacter eiseniae EF01-2	2409	2498	0.96	2488749	2466726	1.01	-17557	-14351	1.22	20116	14331	1.4
Vibrio cholerae	1327	1414	0.94	1279697	1322136	0.97	33439	29069	1.15	1472	9257	0.16
Vibrio cholerae O395	1333	1408	0.95	1303152	1655553	0.79	31540	33292	0.95	3644	10070	0.36
Vibrio fischeri ES114	1266	1308	0.97	1254126	1251195	1	40966	40412	1.01	13893	15483	0.9
Vibrio parahaemolyticus	1491	1588	0.94	1402653	1452306	0.97	32879	30816	1.07	22848	31246	0.73
Vibrio vulnificus CMCP6	1521	1405	1.08	1407990	1353972	1.04	34167	32265	1.06	16197	17779	0.91
Vibrio vulnificus YJ016	1565	1693	0.92	1451028	1520730	0.95	37377	36227	1.03	14839	16583	0.89
Wolbachia endosymbiont of Brugia malayi TRS	357	447	0.8	321606	401706	0.8	15731	24698	0.64	21517	17498	1.23
Wolbachia endosymbiont of Drosophila melanogaster	605	589	1.03	510387	505800	1.01	27059	29245	0.93	29162	32825	0.89
Wolinella succinogenes	1052	989	1.06	1030311	953046	1.08	24785	13375	1.85	9970	13867	0.72
Xanthobacter autotrophicus Py2	2351	2394	0.98	2327253	2335995	1	-24553	-59825	0.41	-22326	-13646	1.64
Xanthomonas campestris	2095	2085	1	2143677	2164605	0.99	13472	12877	1.05	7374	2318	3.18
Xanthomonas campestris 8004	2144	2128	1.01	2164098	2202312	0.98	9902	15995	0.62	3442	5815	0.59
Xanthomonas campestris vesicatoria 85-10	2223	2263	0.98	2215077	2310885	0.96	12937	10967	1.18	8038	4882	1.65
Xanthomonas citri	2178	2133	1.02	2212830	2248905	0.98	13112	16513	0.79	4682	3346	1.4
Xanthomonas oryzae KACC10331	1996	2148	0.93	1900533	2064258	0.92	19569	20678	0.95	7396	5910	1.25
Xanthomonas oryzae MAFF 311018	2084	2287	0.91	1957641	2202417	0.89	21420	26384	0.81	10569	6541	1.62
Xylella fastidiosa	1481	1284	1.15	1194126	1042865	1.15	48090	-10882	-4.42	-35178	10602	-3.32
Xylella fastidiosa Temecula1	1000	1033	0.97	971982	996387	0.98	23963	17357	1.38	-14359	-13230	1.09
Yersinia enterocolitica 8081	1950	2028	0.96	1889269	1966800	0.96	57557	61986	0.93	-15089	-13924	1.08
Yersinia pestis Antiqua	2069	2097	0.99	1985529	1966962	1.01	58345	59421	0.98	-5214	-6041	0.86
Yersinia pestis biovar Mediaevails	1965	1929	1.02	1862877	1883463	0.99	59764	53929	1.11	-9231	-8760	1.05
Yersinia pestis CO92	1978	1906	1.04	1862484	1869337	1	62133	52467	1.18	-6205	-9232	0.67
Yersinia pestis KIM	2103	1982	1.06	1956522	1875477	1.04	61934	53916	1.15	-11590	-4915	2.36
Yersinia pestis Nepal516	1907	2073	0.92	1810125	1989588	0.91	51481	65469	0.79	-5238	-8189	0.64
Yersinia pestis Pestoides F	1719	2130	0.81	1654560	2052477	0.81	33094	79771	0.41	2122	-17290	-0.12
Yersinia pseudotuberculosis IP 31758	2005	2118	0.95	1933711	1991223	0.97	56589	65097	0.87	-12287	-7286	1.69
Yersinia pseudotuberculosis IP32953	1907	1993	0.96	1909581	2005651	0.95	64169	55093	1.16	-8938	-7640	1.17
Zymomonas mobilis ZM4	1045	952	1.1	929175	842682	1.1	33211	6466	5.14	-24760	-6386	3.88

Chromosome	Nu of genes on "+"-strand (Nu+)	Nu of genes on "-"-strand (Nu-)	Nu+/Nu-	Total length of genes on "+"-strand (Length+)	Total length of genes on "-"-strand (Length-)	Length+/ Length-	Cumulative skew (G-C) of genes on "+"-strand (G-C)+	Cumulative skew (G-C) of genes on "-"-strand (G-C)-	(G-C)+/ (G-C)-	Cumulative skew (A-T) of genes on "+"-strand (A-T)+	Cumulative skew (A-T) of genes on "-"-strand (A-T)-	(A-T)+/ (A-T)-
**Archaea**												
Aeropyrum pernix	797	902	0.88	707987	773145	0.92	52374	58296	0.9	15393	24849	0.62
Archaeoglobus fulgidus	1187	1232	0.96	1010425	1005112	1.01	82876	77815	1.07	44429	39517	1.12
Haloarcula marismortui ATCC 43049	1522	1608	0.95	1336242	1370058	0.98	6219	7150	0.87	40555	39474	1.03
Halobacterium sp	1006	1068	0.94	865161	921333	0.94	-3426	-1517	2.26	21183	21324	0.99
Methanobacterium thermoautotrophicum	937	935	1	774132	809708	0.96	34634	34923	0.99	43070	46730	0.92
Methanobrevibacter smithii ATCC 35061	823	969	0.85	769812	902232	0.85	35479	53670	0.66	52945	32838	1.61
Methanococcoides burtonii DSM 6242	1168	1104	1.06	1056924	1011261	1.05	50281	42644	1.18	38475	41291	0.93
Methanococcus aeolicus Nankai-3	744	745	1	652929	668076	0.98	42786	42851	1	72807	77141	0.94
Methanococcus jannaschii	871	857	1.02	773219	697734	1.11	72037	66764	1.08	65425	60062	1.09
Methanococcus maripaludis C5	853	959	0.89	729654	811791	0.9	34464	37587	0.92	63292	73818	0.86
Methanococcus maripaludis C7	920	867	1.06	793224	747165	1.06	36698	35783	1.03	70710	63160	1.12
Methanococcus maripaludis S2	893	828	1.08	766383	710133	1.08	35151	33079	1.06	69268	61846	1.12
Methanococcus vannielii SB	865	812	1.07	757932	714849	1.06	39165	37659	1.04	64989	60030	1.08
Methanocorpusculum labreanum Z	866	872	0.99	775647	796638	0.97	5798	8404	0.69	19573	20856	0.94
Methanoculleus marisnigri JR1	1312	1176	1.12	1130154	1035744	1.09	3954	3352	1.18	19962	20349	0.98
Methanopyrus kandleri	842	844	1	744045	763587	0.97	47378	52194	0.91	17380	16290	1.07
Methanosaeta thermophila PT	778	917	0.85	718221	825738	0.87	49056	56242	0.87	25715	28842	0.89
Methanosarcina acetivorans	2272	2267	1	2091492	2170878	0.96	63229	64086	0.99	96165	113046	0.85
Methanosarcina barkeri fusaro	1795	1810	0.99	1635837	1735053	0.94	52642	59582	0.88	85643	78473	1.09
Methanosarcina mazei	1718	1651	1.04	1534788	1539195	1	52299	52044	1	82797	78941	1.05
Methanosphaera stadtmanae	749	784	0.96	743355	742062	1	27908	30838	0.9	85201	77502	1.1
Methanospirillum hungatei JF-1	1573	1565	1.01	1509147	1540488	0.98	34403	40009	0.86	35052	19237	1.82
Pyrococcus abyssi	929	966	0.96	785505	899582	0.87	66384	69396	0.96	50743	52752	0.96
Pyrococcus furiosus	1054	1070	0.99	869340	896716	0.97	65805	65755	1	63582	64129	0.99
Pyrococcus horikoshii	940	1014	0.93	803875	893090	0.9	61362	71203	0.86	49309	52262	0.94
Sulfolobus acidocaldarius DSM 639	1091	1131	0.96	926103	975670	0.95	61099	60635	1.01	51870	55994	0.93
Sulfolobus solfataricus	1490	1486	1	1711755	1249878	1.37	87672	86556	1.01	61332	71734	0.85
Sulfolobus tokodaii	1416	1408	1.01	1174797	1109574	1.06	72970	66308	1.1	53925	61170	0.88
Thermococcus kodakaraensis KOD1	1126	1179	0.96	1122539	954996	1.18	39175	41466	0.94	53961	50262	1.07
Thermofilum pendens Hrk 5	887	936	0.95	804933	805605	1	46935	51443	0.91	22674	24304	0.93
Thermoplasma acidophilum	767	714	1.07	864258	665127	1.3	29461	27276	1.08	36641	32215	1.14
Thermoplasma volcanium	755	743	1.02	694767	659618	1.05	31086	29221	1.06	39006	35572	1.1
Thermus thermophilus HB27	891	1090	0.82	822135	988023	0.83	-6228	-8998	0.69	-6119	-7975	0.77
Thermus thermophilus HB8	890	1082	0.82	812349	959748	0.85	-9741	-3934	2.48	-4530	-9076	0.5
uncultured methanogenic archaeon RC-I	1471	1613	0.91	1286145	1405869	0.91	12583	14014	0.9	57910	65625	0.88
Nanoarchaeum equitans	256	279	0.92	215535	250633	0.86	12223	12400	0.99	24248	24744	0.98
**Fungi**												
Candida_glabrata_CBS138/NC_005967	109	92	1.18	162942	132786	1.23	2671	3463	0.77	8380	5496	1.52
Candida_glabrata_CBS138/NC_005968	100	108	0.93	148816	165802	0.9	3062	4081	0.75	7199	8744	0.82
Candida_glabrata_CBS138/NC_006026	115	115	1	181432	166980	1.09	4202	2355	1.78	9430	8321	1.13
Candida_glabrata_CBS138/NC_006027	147	130	1.13	225922	192633	1.17	5735	4413	1.3	12689	11030	1.15
Candida_glabrata_CBS138/NC_006028	136	147	0.93	204930	231117	0.89	3820	4268	0.9	11368	12696	0.9
Candida_glabrata_CBS138/NC_006029	179	209	0.86	267154	314611	0.85	5725	6958	0.82	15289	18655	0.82
Candida_glabrata_CBS138/NC_006030	205	229	0.9	305013	345769	0.88	6015	6300	0.95	17349	19580	0.89
Candida_glabrata_CBS138/NC_006031	218	236	0.92	320183	366259	0.87	8648	9348	0.93	20075	21236	0.95
Candida_glabrata_CBS138/NC_006032	214	243	0.88	340581	337746	1.01	7522	8276	0.91	18041	17912	1.01
Candida_glabrata_CBS138/NC_006033	248	265	0.94	372468	417661	0.89	10897	11244	0.97	21897	23627	0.93
Candida_glabrata_CBS138/NC_006034	269	281	0.96	392340	428846	0.91	8659	11875	0.73	21735	26596	0.82
Candida_glabrata_CBS138/NC_006035	291	274	1.06	474113	420863	1.13	12908	10927	1.18	26023	23588	1.1
Candida_glabrata_CBS138/NC_006036	301	307	0.98	462282	475575	0.97	11389	12092	0.94	23732	26440	0.9
Cryptococcus_neoformans_var_JEC21/NC_006670	419	391	1.07	821743	751015	1.09	-7973	-6991	1.14	10528	10066	1.05
Cryptococcus_neoformans_var_JEC21/NC_006679	165	159	1.04	318048	326365	0.97	-3463	-3099	1.12	2737	2374	1.15
Cryptococcus_neoformans_var_JEC21/NC_006680	180	156	1.15	376212	309534	1.22	-4487	-4002	1.12	6590	4726	1.39
Cryptococcus_neoformans_var_JEC21/NC_006681	147	166	0.89	284447	312516	0.91	-3455	-3338	1.04	2739	2385	1.15
Cryptococcus_neoformans_var_JEC21/NC_006682	131	122	1.07	256557	234756	1.09	-1939	-2684	0.72	2654	2503	1.06
Cryptococcus_neoformans_var_JEC21/NC_006683	124	106	1.17	251970	205653	1.23	-1230	-2510	0.49	2808	2016	1.39
Cryptococcus_neoformans_var_JEC21/NC_006684	293	273	1.07	592447	519376	1.14	-3634	-5192	0.7	10423	4637	2.25
Cryptococcus_neoformans_var_JEC21/NC_006685	360	354	1.02	675055	679215	0.99	-5727	-4606	1.24	7947	8348	0.95
Cryptococcus_neoformans_var_JEC21/NC_006686	330	298	1.11	663812	589358	1.13	-6219	-5922	1.05	10056	8091	1.24
Cryptococcus_neoformans_var_JEC21/NC_006687	272	257	1.06	536848	516085	1.04	-3568	-4915	0.73	6603	7330	0.9
Cryptococcus_neoformans_var_JEC21/NC_006691	243	230	1.06	471992	492023	0.96	-3853	-6588	0.58	4749	4540	1.05
Cryptococcus_neoformans_var_JEC21/NC_006692	242	224	1.08	488737	424942	1.15	-5755	-2987	1.93	4444	7483	0.59
Cryptococcus_neoformans_var_JEC21/NC_006693	197	187	1.05	374753	398267	0.94	-3607	-3723	0.97	5164	5810	0.89

Chromosome	Nu of genes on "+"-strand (Nu+)	Nu of genes on "-"-strand (Nu-)	Nu+/Nu-	Total length of genes on "+"-strand (Length+)	Total length of genes on "-"-strand (Length-)	Length+/ Length-	Cumulative skew (G-C) of genes on "+"-strand (G-C)+	Cumulative skew (G-C) of genes on "-"-strand (G-C)-	(G-C)+/ (G-C)-	Cumulative skew (A-T) of genes on "+"-strand (A-T)+	Cumulative skew (A-T) of genes on "-"-strand (A-T)-	(A-T)+/ (A-T)-
Cryptococcus_neoformans_var_JEC21/NC_006694	232	203	1.14	454365	377990	1.2	-2388	-2409	0.99	7402	6294	1.18
Encephalitozoon_cuniculi.I	70	88	0.8	75669	83565	0.91	5999	7122	0.84	3916	4271	0.92
Encephalitozoon_cuniculi.II	81	75	1.08	95124	77385	1.23	6672	6817	0.98	4647	4306	1.08
Encephalitozoon_cuniculi.III	88	69	1.28	90627	76350	1.19	6719	5393	1.25	4094	3643	1.12
Encephalitozoon_cuniculi.IV	89	82	1.09	99150	83961	1.18	8251	6357	1.3	4666	4066	1.15
Encephalitozoon_cuniculi.IX	89	116	0.77	97149	123527	0.79	8074	9580	0.84	4937	5455	0.91
Encephalitozoon_cuniculi.V	93	78	1.19	111795	71163	1.57	9270	4583	2.02	4701	3526	1.33
Encephalitozoon_cuniculi.VI	79	92	0.86	94580	96472	0.98	7319	8077	0.91	4353	4940	0.88
Encephalitozoon_cuniculi.VII	108	79	1.37	120718	80581	1.5	10128	6553	1.55	6269	5002	1.25
Encephalitozoon_cuniculi.VIII	107	103	1.04	113118	92262	1.23	9834	7330	1.34	5332	4602	1.16
Encephalitozoon_cuniculi.X	95	94	1.01	114102	122163	0.93	9605	9800	0.98	5168	5457	0.95
Encephalitozoon_cuniculi.XI	96	114	0.84	100874	129546	0.78	7009	10470	0.67	5257	6792	0.77
Eremothecium_gossypi/NC_005782	199	181	1.1	295887	248871	1.19	7768	6268	1.24	9981	7610	1.31
Eremothecium_gossypi/NC_005783	217	245	0.89	339084	355756	0.95	9141	4587	1.99	10646	11118	0.96
Eremothecium_gossypi/NC_005784	227	268	0.85	328935	398832	0.82	8350	8152	1.02	10940	12108	0.9
Eremothecium_gossypi/NC_005785	430	386	1.11	586080	568393	1.03	11342	12028	0.94	18333	17327	1.06
Eremothecium_gossypi/NC_005786	393	404	0.97	587045	618267	0.95	13637	12114	1.13	19442	18444	1.05
Eremothecium_gossypi/NC_005787	493	489	1.01	747013	708203	1.05	16850	16961	0.99	23018	20871	1.1
Eremothecium_gossypi/NC_005788	380	395	0.96	592287	596023	0.99	13840	11484	1.21	17298	18558	0.93
Eremothecium_gossypi/NC_005789	7	0	0	5355	0	0	30	0	0	-419	0	0
Kluyveromyces_lactis_NRRL_Y-1140/NC_006037	257	272	0.94	357297	388889	0.92	7411	7717	0.96	13139	16012	0.82
Kluyveromyces_lactis_NRRL_Y-1140/NC_006038	325	340	0.96	456989	479313	0.95	8901	8343	1.07	17450	19173	0.91
Kluyveromyces_lactis_NRRL_Y-1140/NC_006039	444	432	1.03	676233	583388	1.16	9204	10963	0.84	30859	22452	1.37
Kluyveromyces_lactis_NRRL_Y-1140/NC_006040	433	444	0.98	584116	612229	0.95	10752	11290	0.95	26699	23651	1.13
Kluyveromyces_lactis_NRRL_Y-1140/NC_006041	559	571	0.98	768135	823003	0.93	12931	15440	0.84	28566	34448	0.83
Kluyveromyces_lactis_NRRL_Y-1140/NC_006042	635	613	1.04	931781	899943	1.04	15039	15810	0.95	38585	38897	0.99
Pichia_stipitis/NC_009042	531	514	1.03	797420	791243	1.01	3166	6206	0.51	24861	22659	1.1
Pichia_stipitis/NC_009043	368	321	1.15	537949	502538	1.07	3766	3060	1.23	16292	12239	1.33
Pichia_stipitis/NC_009044	334	358	0.93	511224	551119	0.93	4475	5681	0.79	15050	16813	0.9
Pichia_stipitis/NC_009045	364	314	1.16	521165	480315	1.09	2975	1325	2.25	14141	13541	1.04
Pichia_stipitis/NC_009046	350	334	1.05	543194	498911	1.09	3900	2701	1.44	16449	15454	1.06
Pichia_stipitis/NC_009047	196	186	1.05	309751	294093	1.05	1336	1547	0.86	10451	8476	1.23
Pichia_stipitis/NC_009048	199	164	1.21	297769	245875	1.21	2049	2038	1.01	8707	7820	1.11
Pichia_stipitis/NC_009068	652	623	1.05	1048229	976138	1.07	8399	3290	2.55	26983	28856	0.94
Saccharomyces_cerevisiae.1	48	45	1.07	68660	71790	0.96	304	-386	-0.79	3309	3592	0.92
Saccharomyces_cerevisiae.10	183	173	1.06	280489	280170	1	3972	2522	1.57	14020	11669	1.2
Saccharomyces_cerevisiae.11	159	152	1.05	241812	238600	1.01	2956	4933	0.6	13026	13721	0.95
Saccharomyces_cerevisiae.12	240	267	0.9	373032	411612	0.91	4857	4920	0.99	19085	20387	0.94
Saccharomyces_cerevisiae.13	238	221	1.08	340915	358460	0.95	4256	4612	0.92	20305	18276	1.11
Saccharomyces_cerevisiae.14	205	187	1.1	309432	272283	1.14	3979	3951	1.01	15783	13921	1.13
Saccharomyces_cerevisiae.15	277	258	1.07	419336	365771	1.15	3665	5025	0.73	22765	17438	1.31
Saccharomyces_cerevisiae.16	243	219	1.11	364536	327046	1.11	5260	4804	1.09	18998	19471	0.98
Saccharomyces_cerevisiae.17	18	0	0	69044	0	0	698	0	0	15	0	0
Saccharomyces_cerevisiae.2	186	219	0.85	270669	339036	0.8	1953	5365	0.36	13402	15755	0.85
Saccharomyces_cerevisiae.3	70	89	0.79	105952	112520	0.94	-338	2166	-0.16	5666	3685	1.54
Saccharomyces_cerevisiae.4	374	380	0.98	573001	561185	1.02	8428	7229	1.17	34342	32659	1.05
Saccharomyces_cerevisiae.5	138	137	1.01	190645	204606	0.93	3394	1178	2.88	8947	10899	0.82
Saccharomyces_cerevisiae.6	63	62	1.02	104746	79905	1.31	1537	1906	0.81	5096	4833	1.05
Saccharomyces_cerevisiae.7	275	250	1.1	433046	350762	1.23	5493	5481	1	20031	17965	1.12
Saccharomyces_cerevisiae.8	152	128	1.19	216354	189007	1.14	1670	3012	0.55	10853	8868	1.22
Saccharomyces_cerevisiae.9	95	111	0.86	140612	171653	0.82	1378	1142	1.21	7824	8114	0.96
Schizosaccharomyces_pombe/NC_001326	9	0	0	13977	0	0	36	0	0	-318	0	0
Schizosaccharomyces_pombe/NC_003421	436	454	0.96	666445	654883	1.02	4584	2356	1.95	-4467	-2667	1.67
Schizosaccharomyces_pombe/NC_003423	942	881	1.07	1383401	1317784	1.05	6833	8780	0.78	-6923	-5380	1.29
Schizosaccharomyces_pombe/NC_003424	1098	1179	0.93	1636691	1815199	0.9	7623	9107	0.84	-7978	-7732	1.03
Yarrowia_lipolytica.A	367	363	1.01	501094	503979	0.99	-12090	-10106	1.2	10351	11249	0.92
Yarrowia_lipolytica.B	480	455	1.05	771082	688802	1.12	-15989	-13104	1.22	13007	12255	1.06
Yarrowia_lipolytica.C	484	471	1.03	672506	700550	0.96	-14886	-13975	1.07	11363	10435	1.09
Yarrowia_lipolytica.D	580	552	1.05	891888	803997	1.11	-17063	-15129	1.13	19249	17272	1.11
Yarrowia_lipolytica.E	709	735	0.96	1067169	1070079	1	-27381	-25063	1.09	16373	16377	1
Yarrowia_lipolytica.F	640	678	0.94	938371	1004734	0.93	-18508	-22798	0.81	18374	17602	1.04
**Human (genes)**												
chromosome1	1419	1362	1.04	53896922	50071992	1.08	504116	437696	1.15	-1386046	-1181196	1.17
chromosome10	565	540	1.05	29815217	30167308	0.99	281691	316434	0.89	-864027	-869808	0.99
chromosome11	797	808	0.99	21026857	22100752	0.95	185408	155140	1.2	-583611	-453638	1.29

Chromosome	Nu of genes on "+"-strand (Nu+)	Nu of genes on "-"-strand (Nu-)	Nu+/Nu-	Total length of genes on "+"-strand (Length+)	Total length of genes on "-"-strand (Length-)	Length+/ Length-	Cumulative skew (G-C) of genes on "+"-strand (G-C)+	Cumulative skew (G-C) of genes on "-"-strand (G-C)-	(G-C)+/ (G-C)-	Cumulative skew (A-T) of genes on "+"-strand (A-T)+	Cumulative skew (A-T) of genes on "-"-strand (A-T)-	(A-T)+/ (A-T)-
chromosome12	679	690	0.98	28267757	29096817	0.97	224563	253856	0.88	-726740	-715291	1.02
chromosome13	270	280	0.96	16414689	15196954	1.08	147826	161809	0.91	-427988	-385419	1.11
chromosome14	636	638	1	20327335	15736613	1.29	189474	153299	1.24	-547911	-427482	1.28
chromosome15	467	477	0.98	19578287	19662774	1	201733	211267	0.95	-491788	-536937	0.92
chromosome16	586	522	1.12	20199633	15670352	1.29	182074	141050	1.29	-499157	-430938	1.16
chromosome17	681	787	0.87	19773506	20752783	0.95	172160	194211	0.89	-511092	-489620	1.04
chromosome18	215	216	1	13913712	12766261	1.09	136843	103161	1.33	-373075	-328014	1.14
chromosome19	878	816	1.08	14023830	13645356	1.03	101399	74721	1.36	-313589	-279193	1.12
chromosome2	975	912	1.07	49547779	46783990	1.06	470584	395977	1.19	-1330685	-1180649	1.13
chromosome20	380	356	1.07	15876523	10715410	1.48	137932	105195	1.31	-369285	-279605	1.32
chromosome21	164	187	0.88	5620138	6800151	0.83	69591	41297	1.69	-153059	-164482	0.93
chromosome22	403	338	1.19	10190010	9395713	1.08	87749	82757	1.06	-222335	-255893	0.87
chromosome3	742	726	1.02	43158373	40205773	1.07	369433	359472	1.03	-1082282	-1040390	1.04
chromosome4	591	562	1.05	30216721	32694954	0.92	245614	269687	0.91	-729279	-791613	0.92
chromosome5	663	604	1.1	36902553	32154878	1.15	295498	251139	1.18	-942257	-743943	1.27
chromosome6	733	771	0.95	33712350	32444575	1.04	304375	286170	1.06	-836243	-803741	1.04
chromosome7	760	691	1.1	36647898	34947806	1.05	265961	327472	0.81	-875685	-922876	0.95
chromosome8	458	525	0.87	25607570	27624699	0.93	191687	238654	0.8	-629923	-659571	0.96
chromosome9	571	576	0.99	23137983	24495301	0.94	222472	233746	0.95	-648231	-654471	0.99
chromosomeX	684	651	1.05	23733399	22825242	1.04	166816	114289	1.46	-617595	-580141	1.06
chromosomeY	174	132	1.32	3274475	2288935	1.43	25803	11113	2.32	-102124	-74750	1.37
**Human (CDS)**												
chromosome1	10187	10041	1.01	1787705	1725154	1.04	2719	6909	0.39	63750	64747	0.98
chromosome10	4289	4049	1.06	735130	664815	1.11	3696	3980	0.93	36240	33381	1.09
chromosome11	5905	5196	1.14	1046531	978273	1.07	-6438	-8111	0.79	22479	21832	1.03
chromosome12	5563	5600	0.99	889781	908679	0.98	3385	6483	0.52	33762	37440	0.9
chromosome13	1788	1798	0.99	304903	356110	0.86	3266	4575	0.71	15229	17789	0.86
chromosome14	3178	3203	0.99	551223	587139	0.94	5566	5664	0.98	21011	23525	0.89
chromosome15	3297	4009	0.82	609733	655999	0.93	7824	10675	0.73	30953	31124	0.99
chromosome16	4619	3882	1.19	761982	682508	1.12	-4555	-1767	2.58	22603	22799	0.99
chromosome17	5308	6378	0.83	898350	1064402	0.84	-5528	983	-5.62	29904	41377	0.72
chromosome18	1548	1471	1.05	284012	259664	1.09	1361	2762	0.49	14905	12422	1.2
chromosome19	5667	5622	1.01	1065637	1127305	0.95	-9029	-13577	0.67	39306	39916	0.98
chromosome2	7273	7712	0.94	1215377	1327324	0.92	8818	12589	0.7	54223	60917	0.89
chromosome20	2688	2208	1.22	486947	409239	1.19	2400	-1950	-1.23	15105	15729	0.96
chromosome21	1079	992	1.09	178504	172945	1.03	787	747	1.05	7309	5220	1.4
chromosome22	2272	2203	1.03	392897	385172	1.02	-2760	-1887	1.46	15319	11639	1.32
chromosome3	5737	5950	0.96	966833	998345	0.97	4066	6258	0.65	35095	40927	0.86
chromosome4	3904	3786	1.03	793087	654287	1.21	8517	7924	1.07	31250	32039	0.98
chromosome5	4623	4192	1.1	921243	709651	1.3	9044	3678	2.46	40137	29439	1.36
chromosome6	4793	5113	0.94	855479	910629	0.94	4826	8599	0.56	37823	37198	1.02
chromosome7	4934	4654	1.06	876181	790630	1.11	-3166	598	-5.29	31343	29044	1.08
chromosome8	3365	3370	1	570273	639071	0.89	5509	5142	1.07	23842	25953	0.92
chromosome9	4052	3930	1.03	696681	713996	0.98	1138	649	1.75	29587	28119	1.05
chromosomeX	3579	3470	1.03	695467	681338	1.02	3354	4087	0.82	28819	33757	0.85
chromosomeY	386	224	1.72	67362	41245	1.63	955	-32	-29.84	2971	1499	1.98
