# Supplementary material for: Hidden Chromosome Symmetry: In Silico Transformation Reveals Symmetry in 2D DNA Walk Trajectories of 671 Chromosomes
Source: PLoS One. 2009 Jul 28;4(7):e6396. doi: 10.1371/journal.pone.0006396 (PMC2712679; doi:10.1371/journal.pone.0006396)
Supplement: Table S3 — Total number, total length and cumulative nucleotide skews of genes located on leading/lagging strand in two chromosome halves (approximate replichores) in 524 bacteria. (0.20 MB PDF) [file pone.0006396.s009.pdf]

**Supplementary Table 3. Total number, total length and cumulative nucleotide skews of genes located on leading/lagging strand in two chromosome halves (approximate replichores) in 524 bacteria.**

*In the 1st half of the chromosome "+"-strand is leading and "-"-strand is lagging; in the 2nd half "-" strand is leading and "+"-strand is lagging.*

## Bacteria

| Chromosome | Nu of genes 1st half (Nu1) | Nu of genes 2nd half (Nu2) | Nu1/Nu2 | Total length of genes 1st half (Length1) | Total length of genes 2nd half (Length2) | Length1/ Length2 | Cumulative skew (G-C) of genes 1st half (G-C)1 | Cumulative skew (G-C) of genes 2nd half (G-C)2 | (G-C)1/ (G-C)2 | Cumulative skew (A-T) of genes 1st half (A-T)1 | Cumulative skew (A-T) of genes 2nd half (A-T)2 | (A-T)1/ (A-T)2 |
|---|---|---|---|---|---|---|---|---|---|---|---|---|
| Acidobacteria_bacterium_Ellin345 (leading) | 1259 | 1303 | 0.97 | 1357539 | 1382667 | 0.98 | 11954 | 10349 | 1.16 | 22075 | 19270 | 1.15 |
| Acidobacteria_bacterium_Ellin345 (lagging) | 1125 | 1089 | 1.03 | 1183011 | 1128102 | 1.05 | -7965 | -4991 | 1.6 | 22904 | 22561 | 1.02 |
| Acidothermus_cellulolyticus_11B (leading) | 747 | 669 | 1.12 | 761925 | 683025 | 1.12 | 9317 | 9600 | 0.97 | -15590 | -14781 | 1.05 |
| Acidothermus_cellulolyticus_11B (lagging) | 352 | 388 | 0.91 | 345477 | 405450 | 0.85 | -9147 | -7416 | 1.23 | -2904 | -3072 | 0.95 |
| Acidovorax_avenae_citrulli_AAC00-1 (leading) | 1340 | 1234 | 1.09 | 1331661 | 1304232 | 1.02 | 2418 | -2440 | -0.99 | 8925 | 7978 | 1.12 |
| Acidovorax_avenae_citrulli_AAC00-1 (lagging) | 1068 | 1066 | 1 | 1052775 | 1086867 | 0.97 | -23396 | -21969 | 1.06 | 14019 | 12500 | 1.12 |
| Acidovorax_JS42.3 (leading) | 1053 | 1100 | 0.96 | 1022760 | 1097913 | 0.93 | 10125 | 10552 | 0.96 | 5905 | 8169 | 0.72 |
| Acidovorax_JS42.3 (lagging) | 968 | 885 | 1.09 | 933324 | 854731 | 1.09 | -18243 | -15271 | 1.19 | 11693 | 12589 | 0.93 |
| Acinetobacter_baumannii_ATCC_17978.3 (leading) | 1007 | 964 | 1.04 | 867993 | 854364 | 1.02 | 37385 | 34429 | 1.09 | 56 | -2435 | -0.02 |
| Acinetobacter_baumannii_ATCC_17978.3 (lagging) | 632 | 748 | 0.84 | 529371 | 605625 | 0.87 | 3573 | 5892 | 0.61 | 3712 | 1667 | 2.23 |
| Acinetobacter_sp_ADP1 (leading) | 1023 | 969 | 1.06 | 1011204 | 945186 | 1.07 | 42020 | 38196 | 1.1 | -2432 | -3388 | 0.72 |
| Acinetobacter_sp_ADP1 (lagging) | 618 | 714 | 0.87 | 574917 | 632418 | 0.91 | 3636 | 6053 | 0.6 | 1129 | 979 | 1.15 |
| Actinobacillus_pleuropneumoniae_L20 (leading) | 503 | 556 | 0.9 | 516084 | 543204 | 0.95 | 21803 | 21244 | 1.03 | 13093 | 10486 | 1.25 |
| Actinobacillus_pleuropneumoniae_L20 (lagging) | 465 | 487 | 0.95 | 477903 | 426375 | 1.12 | 14794 | 12689 | 1.17 | 12527 | 7904 | 1.58 |
| Actinobacillus_succinogenes_130Z (leading) | 589 | 555 | 1.06 | 575475 | 554361 | 1.04 | 25184 | 23228 | 1.08 | 5509 | 6199 | 0.89 |
| Actinobacillus_succinogenes_130Z (lagging) | 453 | 481 | 0.94 | 436266 | 473043 | 0.92 | 11601 | 11845 | 0.98 | 7249 | 9518 | 0.76 |
| Aeromonas_hydrophila_ATCC_7966 (leading) | 1170 | 1086 | 1.08 | 1164675 | 1152492 | 1.01 | 10937 | 11048 | 0.99 | 1418 | -166 | -8.54 |
| Aeromonas_hydrophila_ATCC_7966 (lagging) | 933 | 932 | 1 | 900567 | 935542 | 0.96 | -32764 | -31096 | 1.05 | 11697 | 13053 | 0.9 |
| Aeromonas_salmonicida_A449 (leading) | 1175 | 1094 | 1.07 | 1147991 | 1026325 | 1.12 | 17635 | 10782 | 1.64 | -6017 | -2789 | 2.16 |
| Aeromonas_salmonicida_A449 (lagging) | 887 | 929 | 0.95 | 817077 | 881003 | 0.93 | -16964 | -19444 | 0.87 | 10473 | 9916 | 1.06 |
| Agrobacterium_tumefaciens_C58_Cereon (leading) | 783 | 742 | 1.06 | 718038 | 705612 | 1.02 | 1531 | 9905 | 0.15 | -8729 | -11987 | 0.73 |
| Agrobacterium_tumefaciens_C58_Cereon (lagging) | 605 | 584 | 1.04 | 553188 | 559071 | 0.99 | -8525 | -15131 | 0.56 | -125 | 3094 | -0.04 |
| Agrobacterium_tumefaciens_C58_UWash (leading) | 782 | 770 | 1.02 | 699153 | 696624 | 1 | 737 | 9596 | 0.08 | -9134 | -12108 | 0.75 |
| Agrobacterium_tumefaciens_C58_UWash (lagging) | 616 | 616 | 1 | 548064 | 551505 | 0.99 | -8746 | -15288 | 0.57 | -346 | 2553 | -0.14 |
| Alcanivorax_borkumensis_SK2 (leading) | 848 | 789 | 1.07 | 829683 | 797259 | 1.04 | 53824 | 47233 | 1.14 | -21095 | -17474 | 1.21 |
| Alcanivorax_borkumensis_SK2 (lagging) | 524 | 593 | 0.88 | 542109 | 566928 | 0.96 | -16140 | -19915 | 0.81 | 14129 | 17141 | 0.82 |
| Alkalilimnicola_ehrlichei_MLHE-1 (leading) | 844 | 820 | 1.03 | 875616 | 848982 | 1.03 | 24825 | 24140 | 1.03 | -5573 | -7036 | 0.79 |
| Alkalilimnicola_ehrlichei_MLHE-1 (lagging) | 577 | 623 | 0.93 | 597438 | 642939 | 0.93 | -10919 | -12258 | 0.89 | 4883 | 7149 | 0.68 |
| Alkaliphilus_metalliredigens_QYMF (leading) | 1950 | 1907 | 1.02 | 1716104 | 1735161 | 0.99 | 125748 | 123725 | 1.02 | 115561 | 121314 | 0.95 |
| Alkaliphilus_metalliredigens_QYMF (lagging) | 357 | 410 | 0.87 | 297996 | 341222 | 0.87 | 6062 | 10520 | 0.58 | 8124 | 12821 | 0.63 |
| Anabaena_variabilis_ATCC_29413.4 (leading) | 1344 | 1152 | 1.17 | 1388691 | 1199160 | 1.16 | 20064 | 18161 | 1.1 | 26619 | 19959 | 1.33 |
| Anabaena_variabilis_ATCC_29413.4 (lagging) | 1222 | 1324 | 0.92 | 1199172 | 1408245 | 0.85 | 16820 | 20366 | 0.83 | 20628 | 24499 | 0.84 |
| Anaeromyxobacter_dehalogenans_2CP-C (leading) | 1145 | 1203 | 0.95 | 1191927 | 1286235 | 0.93 | 14849 | 17034 | 0.87 | -5452 | -6057 | 0.9 |
| Anaeromyxobacter_dehalogenans_2CP-C (lagging) | 1067 | 930 | 1.15 | 1093392 | 992418 | 1.1 | 4189 | -1293 | -3.24 | -5419 | -11477 | 0.47 |
| Anaeromyxobacter_Fw109-5 (leading) | 1285 | 1222 | 1.05 | 1328754 | 1363242 | 0.97 | 2364 | 8234 | 0.29 | -4510 | -7262 | 0.62 |
| Anaeromyxobacter_Fw109-5 (lagging) | 1022 | 936 | 1.09 | 1077768 | 977262 | 1.1 | -11701 | -12376 | 0.95 | -6767 | -9216 | 0.73 |
| Anaplasma_marginale_St_Maries (leading) | 232 | 312 | 0.74 | 270510 | 319269 | 0.85 | 19699 | 25385 | 0.78 | -1267 | -3486 | 0.36 |
| Anaplasma_marginale_St_Maries (lagging) | 220 | 184 | 1.2 | 236964 | 199104 | 1.19 | 11042 | 10935 | 1.01 | 8856 | 6421 | 1.38 |
| Anaplasma_phagocytophilum_HZ (leading) | 392 | 362 | 1.08 | 309954 | 284880 | 1.09 | 34807 | 28247 | 1.23 | -7007 | -4381 | 1.6 |
| Anaplasma_phagocytophilum_HZ (lagging) | 258 | 251 | 1.03 | 205612 | 203403 | 1.01 | 8467 | 10798 | 0.78 | 3928 | 1915 | 2.05 |
| Aquifex_aeolicus (leading) | 402 | 346 | 1.16 | 372558 | 330465 | 1.13 | 18700 | 17499 | 1.07 | 31240 | 29734 | 1.05 |
| Aquifex_aeolicus (lagging) | 369 | 411 | 0.9 | 350433 | 399288 | 0.88 | 18362 | 18411 | 1 | 32355 | 34715 | 0.93 |
| Arthrobacter_aurescens_TC1 (leading) | 1117 | 1192 | 0.94 | 1145580 | 1186320 | 0.97 | -9625 | -6557 | 1.47 | 1925 | -3157 | -0.61 |
| Arthrobacter_aurescens_TC1 (lagging) | 880 | 851 | 1.03 | 874380 | 850455 | 1.03 | -23291 | -21728 | 1.07 | 3693 | 6099 | 0.61 |
| Arthrobacter_FB24.4 (leading) | 1181 | 1203 | 0.98 | 1234473 | 1250253 | 0.99 | -18749 | -17078 | 1.1 | 12942 | 8907 | 1.45 |
| Arthrobacter_FB24.4 (lagging) | 878 | 883 | 0.99 | 856827 | 867930 | 0.99 | -22740 | -23732 | 0.96 | 7831 | 7992 | 0.98 |
| Aster_yellows_witches-broom_phytoplasma_AYWB (leadin | 212 | 261 | 0.81 | 155640 | 208983 | 0.74 | -1905 | -1463 | 1.3 | 10833 | 9770 | 1.11 |
| Aster_yellows_witches-broom_phytoplasma_AYWB (laggin | 118 | 79 | 1.49 | 93657 | 61296 | 1.53 | -3053 | -1277 | 2.39 | 11106 | 5895 | 1.88 |
| Azoarcus_BH72 (leading) | 1121 | 1050 | 1.07 | 1143867 | 1043451 | 1.1 | 5413 | 5951 | 0.91 | -3028 | -3462 | 0.87 |
| Azoarcus_BH72 (lagging) | 865 | 952 | 0.91 | 855912 | 955263 | 0.9 | -28666 | -34767 | 0.82 | 9710 | 11466 | 0.85 |
| Azoarcus_sp_EbN1 (leading) | 925 | 894 | 1.03 | 863658 | 848074 | 1.02 | -12631 | -9457 | 1.34 | 10597 | 8094 | 1.31 |
| Azoarcus_sp_EbN1 (lagging) | 1127 | 1186 | 0.95 | 1081968 | 1127912 | 0.96 | 6475 | 10894 | 0.59 | 4605 | 2266 | 2.03 |
| Bacillus_anthracis_Ames (leading) | 1862 | 2037 | 0.91 | 1510225 | 1669239 | 0.9 | 113753 | 124919 | 0.91 | 83542 | 90609 | 0.92 |
| Bacillus_anthracis_Ames (lagging) | 731 | 680 | 1.08 | 562626 | 479091 | 1.17 | 5819 | 2361 | 2.46 | 21753 | 16344 | 1.33 |
| Bacillus_anthracis_Ames_0581.3 (leading) | 1861 | 2036 | 0.91 | 1509691 | 1669431 | 0.9 | 113734 | 124901 | 0.91 | 83429 | 90647 | 0.92 |
| Bacillus_anthracis_Ames_0581.3 (lagging) | 731 | 680 | 1.08 | 562632 | 479058 | 1.17 | 5819 | 2353 | 2.47 | 21755 | 16345 | 1.33 |
| Bacillus_anthracis_str_Sterne (leading) | 1884 | 2019 | 0.93 | 1584345 | 1721430 | 0.92 | 119279 | 129563 | 0.92 | 87460 | 93794 | 0.93 |
| Bacillus_anthracis_str_Sterne (lagging) | 711 | 672 | 1.06 | 585948 | 506040 | 1.16 | 6141 | 2364 | 2.6 | 22675 | 17892 | 1.27 |
| Bacillus_cereus_ATCC14579.2 (leading) | 1936 | 1985 | 0.98 | 1651233 | 1691331 | 0.98 | 124980 | 127847 | 0.98 | 94343 | 95822 | 0.98 |
| Bacillus_cereus_ATCC14579.2 (lagging) | 638 | 674 | 0.95 | 519777 | 507684 | 1.02 | 650 | 2997 | 0.22 | 17707 | 18589 | 0.95 |
| Bacillus_cereus_ATCC_10987 (leading) | 2001 | 2101 | 0.95 | 1666131 | 1712299 | 0.97 | 127110 | 129223 | 0.98 | 93677 | 95492 | 0.98 |

| Chromosome | Nu of genes 1st half (Nu1) | Nu of genes 2nd half (Nu2) | Nu1/Nu2 | Total length of genes 1st half (Length1) | Total length of genes 2nd half (Length2) | Length1/ Length2 | Cumulative skew (G-C) of genes 1st half (G-C)1 | Cumulative skew (G-C) of genes 2nd half (G-C)2 | (G-C)1/ (G-C)2 | Cumulative skew (A-T) of genes 1st half (A-T)1 | Cumulative skew (A-T) of genes 2nd half (A-T)2 | (A-T)1/ (A-T)2 |
|---|---|---|---|---|---|---|---|---|---|---|---|---|
| Bacillus_cereus_ATCC_10987 (lagging) | 729 | 771 | 0.95 | 524367 | 544671 | 0.96 | 2687 | 1931 | 1.39 | 16441 | 20290 | 0.81 |
| Bacillus_cereus_cytotoxis_NVH_391-98.2 (leading) | 1338 | 1500 | 0.89 | 1174812 | 1293489 | 0.91 | 85318 | 95567 | 0.89 | 62902 | 71750 | 0.88 |
| Bacillus_cereus_cytotoxis_NVH_391-98.2 (lagging) | 504 | 490 | 1.03 | 413127 | 364389 | 1.13 | 3315 | 2001 | 1.66 | 14504 | 14624 | 0.99 |
| Bacillus_cereus_ZK (leading) | 1804 | 1975 | 0.91 | 1617642 | 1729515 | 0.94 | 121409 | 130718 | 0.93 | 89057 | 94487 | 0.94 |
| Bacillus_cereus_ZK (lagging) | 689 | 665 | 1.04 | 577995 | 530112 | 1.09 | 4999 | 2023 | 2.47 | 22324 | 19125 | 1.17 |
| Bacillus_clausii_KSM-K16 (leading) | 1498 | 1583 | 0.95 | 1373190 | 1449342 | 0.95 | 65380 | 69216 | 0.94 | 35482 | 43108 | 0.82 |
| Bacillus_clausii_KSM-K16 (lagging) | 520 | 494 | 1.05 | 460809 | 415407 | 1.11 | 4077 | 2497 | 1.63 | 8514 | 11360 | 0.75 |
| Bacillus_halodurans (leading) | 1554 | 1504 | 1.03 | 1414923 | 1330416 | 1.06 | 82606 | 76058 | 1.09 | 36977 | 43134 | 0.86 |
| Bacillus_halodurans (lagging) | 449 | 558 | 0.8 | 363630 | 471011 | 0.77 | 4663 | 8900 | 0.52 | 6443 | 14314 | 0.45 |
| Bacillus_licheniformis_ATCC_14580 (leading) | 1471 | 1584 | 0.93 | 1350114 | 1398531 | 0.97 | 57136 | 56918 | 1 | 67870 | 67337 | 1.01 |
| Bacillus_licheniformis_ATCC_14580 (lagging) | 568 | 528 | 1.08 | 450750 | 433752 | 1.04 | 5669 | 4035 | 1.4 | 20379 | 16085 | 1.27 |
| Bacillus_licheniformis_DSM_13 (leading) | 1482 | 1592 | 0.93 | 1366293 | 1411506 | 0.97 | 58121 | 57479 | 1.01 | 68544 | 68215 | 1 |
| Bacillus_licheniformis_DSM_13 (lagging) | 574 | 547 | 1.05 | 460161 | 444171 | 1.04 | 5519 | 4237 | 1.3 | 21210 | 15836 | 1.34 |
| Bacillus_subtilis (leading) | 1391 | 1618 | 0.86 | 1344990 | 1404980 | 0.96 | 63071 | 66859 | 0.94 | 60215 | 63490 | 0.95 |
| Bacillus_subtilis (lagging) | 545 | 550 | 0.99 | 474552 | 452982 | 1.05 | 6731 | 5492 | 1.23 | 18631 | 16294 | 1.14 |
| Bacillus_thuringiensis_Al_Hakam.2 (leading) | 1712 | 1801 | 0.95 | 1602249 | 1694514 | 0.95 | 122792 | 128251 | 0.96 | 91751 | 94411 | 0.97 |
| Bacillus_thuringiensis_Al_Hakam.2 (lagging) | 614 | 608 | 1.01 | 542346 | 517020 | 1.05 | 3465 | 2318 | 1.49 | 21063 | 20774 | 1.01 |
| Bacillus_thuringiensis_konkukian (leading) | 1812 | 1963 | 0.92 | 1588956 | 1719828 | 0.92 | 118899 | 129876 | 0.92 | 86991 | 95491 | 0.91 |
| Bacillus_thuringiensis_konkukian (lagging) | 689 | 652 | 1.06 | 571974 | 514164 | 1.11 | 4081 | 1913 | 2.13 | 20185 | 18325 | 1.1 |
| Bacteroides_fragilis_NCTC_9434 (leading) | 1229 | 1236 | 0.99 | 1339782 | 1327158 | 1.01 | 81800 | 77136 | 1.06 | 5004 | 1620 | 3.09 |
| Bacteroides_fragilis_NCTC_9434 (lagging) | 876 | 842 | 1.04 | 982881 | 939366 | 1.05 | 3817 | 3445 | 1.11 | 60528 | 56223 | 1.08 |
| Bacteroides_fragilis_YCH46.2 (leading) | 1242 | 1323 | 0.94 | 1340268 | 1350324 | 0.99 | 82371 | 80099 | 1.03 | -2061 | 335 | -6.15 |
| Bacteroides_fragilis_YCH46.2 (lagging) | 1020 | 992 | 1.03 | 1067139 | 997758 | 1.07 | 4696 | 2606 | 1.8 | 67355 | 63026 | 1.07 |
| Bacteroides_thetaiotaomicron_VPI-5482 (leading) | 1192 | 1100 | 1.08 | 1353507 | 1357170 | 1 | 35962 | 31204 | 1.15 | 45945 | 63624 | 0.72 |
| Bacteroides_thetaiotaomicron_VPI-5482 (lagging) | 1313 | 1172 | 1.12 | 1427769 | 1467714 | 0.97 | 59835 | 64685 | 0.93 | 35020 | 38659 | 0.91 |
| Bacteroides_vulgatus_ATCC_8482 (leading) | 942 | 1015 | 0.93 | 1102422 | 1090983 | 1.01 | 37918 | 39672 | 0.96 | 38866 | 41743 | 0.93 |
| Bacteroides_vulgatus_ATCC_8482 (lagging) | 994 | 1113 | 0.89 | 1204125 | 1169703 | 1.03 | 65096 | 57396 | 1.13 | 21775 | 29741 | 0.73 |
| Bartonella_bacilliformis_KC583 (leading) | 455 | 295 | 1.54 | 378612 | 276153 | 1.37 | 27673 | 16965 | 1.63 | -12865 | -8480 | 1.52 |
| Bartonella_bacilliformis_KC583 (lagging) | 223 | 309 | 0.72 | 209400 | 301810 | 0.69 | -3766 | 175 | -21.52 | 3404 | -1020 | -3.34 |
| Bartonella_henselae_Houston-1 (leading) | 459 | 404 | 1.14 | 452724 | 380487 | 1.19 | 30616 | 24356 | 1.26 | -10532 | -15217 | 0.69 |
| Bartonella_henselae_Houston-1 (lagging) | 334 | 290 | 1.15 | 311187 | 261024 | 1.19 | -2579 | -6533 | 0.39 | 5576 | 8205 | 0.68 |
| Bartonella_quintana_Toulouse (leading) | 342 | 327 | 1.05 | 343749 | 332478 | 1.03 | 22400 | 21837 | 1.03 | -13189 | -14107 | 0.93 |
| Bartonella_quintana_Toulouse (lagging) | 236 | 236 | 1 | 247983 | 217392 | 1.14 | -2958 | -4651 | 0.64 | 3143 | 5439 | 0.58 |
| Baumannia_cicadellinicola_Homalodisca_coagulata (leadin | 151 | 158 | 0.96 | 152355 | 156630 | 0.97 | 3524 | 3786 | 0.93 | 2938 | 2503 | 1.17 |
| Baumannia_cicadellinicola_Homalodisca_coagulata (laggin | 136 | 149 | 0.91 | 132768 | 143562 | 0.92 | 3304 | 3642 | 0.91 | 3131 | 2222 | 1.41 |
| Bdellovibrio_bacteriovorus (leading) | 1058 | 952 | 1.11 | 1012407 | 957150 | 1.06 | 43175 | 40228 | 1.07 | -694 | 880 | -0.79 |
| Bdellovibrio_bacteriovorus (lagging) | 749 | 827 | 0.91 | 740799 | 807933 | 0.92 | -9619 | -9807 | 0.98 | 19068 | 18910 | 1.01 |
| Bifidobacterium_adolescentis_ATCC_15703 (leading) | 605 | 532 | 1.14 | 684195 | 612714 | 1.12 | -3680 | -3798 | 0.97 | 13591 | 14298 | 0.95 |
| Bifidobacterium_adolescentis_ATCC_15703 (lagging) | 232 | 261 | 0.89 | 242787 | 269385 | 0.9 | -1671 | -1696 | 0.99 | 3863 | 7145 | 0.54 |
| Bifidobacterium_longum (leading) | 424 | 413 | 1.03 | 474525 | 449757 | 1.06 | -9702 | -6476 | 1.5 | 7913 | 3253 | 2.43 |
| Bifidobacterium_longum (lagging) | 454 | 435 | 1.04 | 505689 | 495993 | 1.02 | -10353 | -9093 | 1.14 | 8622 | 5878 | 1.47 |
| Bordetella_bronchiseptica (leading) | 1444 | 1321 | 1.09 | 1428159 | 1310238 | 1.09 | 22303 | 8959 | 2.49 | -4302 | -2817 | 1.53 |
| Bordetella_bronchiseptica (lagging) | 1057 | 1171 | 0.9 | 1022689 | 1144398 | 0.89 | -33418 | -31610 | 1.06 | 12412 | 11942 | 1.04 |
| Bordetella_parapertussis (leading) | 1245 | 1044 | 1.19 | 1231752 | 1076352 | 1.14 | 17926 | 2122 | 8.45 | -2252 | -1050 | 2.14 |
| Bordetella_parapertussis (lagging) | 881 | 1014 | 0.87 | 834547 | 990127 | 0.84 | -27933 | -22782 | 1.23 | 7667 | 6046 | 1.27 |
| Bordetella_pertussis (leading) | 956 | 933 | 1.02 | 971862 | 913657 | 1.06 | -699 | -4640 | 0.15 | 4689 | 825 | 5.68 |
| Bordetella_pertussis (lagging) | 746 | 800 | 0.93 | 716704 | 774255 | 0.93 | -20198 | -16515 | 1.22 | 6653 | 3230 | 2.06 |
| Borrelia_afzelii_PKo.3 (leading) | 143 | 144 | 0.99 | 143790 | 148428 | 0.97 | -792 | 240 | -3.3 | 20456 | 23120 | 0.88 |
| Borrelia_afzelii_PKo.3 (lagging) | 290 | 278 | 1.04 | 277749 | 278856 | 1 | 21864 | 21313 | 1.03 | -4753 | -5025 | 0.95 |
| Borrelia_burgdorferi.11 (leading) | 149 | 136 | 1.1 | 146793 | 143679 | 1.02 | -733 | -60 | 12.22 | 21158 | 23212 | 0.91 |
| Borrelia_burgdorferi.11 (lagging) | 287 | 278 | 1.03 | 281445 | 280323 | 1 | 21647 | 21441 | 1.01 | -4545 | -5623 | 0.81 |
| Borrelia_garinii_PBi.3 (leading) | 138 | 139 | 0.99 | 141597 | 146412 | 0.97 | -248 | 807 | -0.31 | 20435 | 22939 | 0.89 |
| Borrelia_garinii_PBi.3 (lagging) | 284 | 270 | 1.05 | 275622 | 276267 | 1 | 20613 | 20463 | 1.01 | -4083 | -4378 | 0.93 |
| Bradyrhizobium_BTAi1.2 (leading) | 2133 | 2083 | 1.02 | 2081478 | 2017503 | 1.03 | 10237 | 13107 | 0.78 | -23371 | -15440 | 1.51 |
| Bradyrhizobium_BTAi1.2 (lagging) | 1567 | 1610 | 0.97 | 1478226 | 1518024 | 0.97 | -25029 | -29084 | 0.86 | 2111 | 5666 | 0.37 |
| Bradyrhizobium_japonicum (leading) | 2287 | 2340 | 0.98 | 2197407 | 2219526 | 0.99 | -7389 | -9208 | 0.8 | -3942 | -722 | 5.46 |
| Bradyrhizobium_japonicum (lagging) | 1829 | 1860 | 0.98 | 1774890 | 1736193 | 1.02 | -30291 | -30580 | 0.99 | 11589 | 10863 | 1.07 |
| Bradyrhizobium_ORS278 (leading) | 1836 | 1915 | 0.96 | 1783383 | 1846794 | 0.97 | -1362 | -4351 | 0.31 | -6163 | -8221 | 0.75 |
| Bradyrhizobium_ORS278 (lagging) | 1482 | 1483 | 1 | 1403832 | 1357485 | 1.03 | -22870 | -23287 | 0.98 | 5516 | 3074 | 1.79 |
| Brucella_abortus_9-941 (leading) | 566 | 605 | 0.94 | 505722 | 509961 | 0.99 | 9843 | 12825 | 0.77 | -7399 | -7198 | 1.03 |
| Brucella_abortus_9-941 (lagging) | 433 | 425 | 1.02 | 349938 | 357024 | 0.98 | -4053 | -4729 | 0.86 | 155 | 1159 | 0.13 |
| Brucella_melitensis (leading) | 617 | 605 | 1.02 | 552939 | 565521 | 0.98 | 13839 | 10959 | 1.26 | -8056 | -9032 | 0.89 |
| Brucella_melitensis (lagging) | 400 | 436 | 0.92 | 341931 | 359307 | 0.95 | -5008 | -4418 | 1.13 | 1851 | 450 | 4.11 |
| Brucella_melitensis_biovar_Abortus (leading) | 558 | 599 | 0.93 | 498444 | 506628 | 0.98 | 9600 | 12724 | 0.75 | -7596 | -7082 | 1.07 |
| Brucella_melitensis_biovar_Abortus (lagging) | 429 | 413 | 1.04 | 349929 | 354468 | 0.99 | -4032 | -4595 | 0.88 | 171 | 1101 | 0.16 |
| Brucella_ovis (leading) | 272 | 245 | 1.11 | 247962 | 224961 | 1.1 | 4613 | 5613 | 0.82 | -6371 | -4776 | 1.33 |
| Brucella_ovis (lagging) | 222 | 222 | 1 | 206061 | 212592 | 0.97 | -2483 | -3045 | 0.82 | -80 | 445 | -0.18 |

| Chromosome | Nu of genes 1st half (Nu1) | Nu of genes 2nd half (Nu2) | Nu1/Nu2 | Total length of genes 1st half (Length1) | Total length of genes 2nd half (Length2) | Length1/ Length2 | Cumulative skew (G-C) of genes 1st half (G-C)1 | Cumulative skew (G-C) of genes 2nd half (G-C)2 | (G-C)1/ (G-C)2 | Cumulative skew (A-T) of genes 1st half (A-T)1 | Cumulative skew (A-T) of genes 2nd half (A-T)2 | (A-T)1/ (A-T)2 |
|---|---|---|---|---|---|---|---|---|---|---|---|---|
| Brucella_suis_1330 (leading) | 580 | 628 | 0.92 | 522563 | 519897 | 1.01 | 9838 | 12851 | 0.77 | -8755 | -7626 | 1.15 |
| Brucella_suis_1330 (lagging) | 469 | 445 | 1.05 | 375081 | 362436 | 1.03 | -4107 | -4562 | 0.9 | -50 | 804 | -0.06 |
| Buchnera_aphidicola (leading) | 146 | 140 | 1.04 | 147420 | 129138 | 1.14 | 8940 | 7631 | 1.17 | -1426 | 1371 | -1.04 |
| Buchnera_aphidicola (lagging) | 101 | 116 | 0.87 | 109926 | 112971 | 0.97 | -502 | -987 | 0.51 | 12330 | 12260 | 1.01 |
| Buchnera_aphidicola_Sg (leading) | 160 | 142 | 1.13 | 161577 | 128025 | 1.26 | 6203 | 4797 | 1.29 | 3062 | 3290 | 0.93 |
| Buchnera_aphidicola_Sg (lagging) | 106 | 137 | 0.77 | 112614 | 133173 | 0.85 | 2881 | 3107 | 0.93 | 10431 | 13480 | 0.77 |
| Burkholderia_383.3 (leading) | 868 | 884 | 0.98 | 895788 | 898035 | 1 | 23621 | 22639 | 1.04 | -3667 | -2672 | 1.37 |
| Burkholderia_383.3 (lagging) | 708 | 713 | 0.99 | 675015 | 677292 | 1 | -12381 | -9377 | 1.32 | 2492 | 1955 | 1.27 |
| Burkholderia_cenocepacia_AU_1054 (leading) | 828 | 870 | 0.95 | 834006 | 831498 | 1 | 13213 | 16661 | 0.79 | 1501 | 2081 | 0.72 |
| Burkholderia_cenocepacia_AU_1054 (lagging) | 645 | 621 | 1.04 | 615849 | 599001 | 1.03 | -5766 | -9870 | 0.58 | 2553 | 8761 | 0.29 |
| Burkholderia_cenocepacia_HI2424 (leading) | 929 | 870 | 1.07 | 900303 | 846120 | 1.06 | 20054 | 14513 | 1.38 | 3913 | 2823 | 1.39 |
| Burkholderia_cenocepacia_HI2424 (lagging) | 647 | 712 | 0.91 | 616689 | 687855 | 0.9 | -10465 | -9783 | 1.07 | 6528 | 7840 | 0.83 |
| Burkholderia_cepacia_AMMD.2 (leading) | 992 | 893 | 1.11 | 972246 | 890061 | 1.09 | 19597 | 16098 | 1.22 | 4539 | 4863 | 0.93 |
| Burkholderia_cepacia_AMMD.2 (lagging) | 606 | 721 | 0.84 | 575895 | 673434 | 0.86 | -8101 | -10371 | 0.78 | 4848 | 8125 | 0.6 |
| Burkholderia_mallei_ATCC_23344 (leading) | 685 | 877 | 0.78 | 648822 | 801918 | 0.81 | 4972 | 13347 | 0.37 | 1374 | 343 | 4.01 |
| Burkholderia_mallei_ATCC_23344 (lagging) | 759 | 673 | 1.13 | 730065 | 625132 | 1.17 | 6768 | -668 | -10.13 | 5023 | 4533 | 1.11 |
| Burkholderia_mallei_NCTC_10229.2 (leading) | 796 | 715 | 1.11 | 685080 | 628956 | 1.09 | -2289 | -1535 | 1.49 | 2569 | 4769 | 0.54 |
| Burkholderia_mallei_NCTC_10229.2 (lagging) | 861 | 960 | 0.9 | 811719 | 867474 | 0.94 | 14660 | 15810 | 0.93 | 647 | 2630 | 0.25 |
| Burkholderia_mallei_NCTC_10247.2 (leading) | 826 | 850 | 0.97 | 722964 | 739155 | 0.98 | 8409 | 6242 | 1.35 | 648 | 4303 | 0.15 |
| Burkholderia_mallei_NCTC_10247.2 (lagging) | 888 | 911 | 0.97 | 787044 | 782253 | 1.01 | 4495 | 8526 | 0.53 | 2524 | 2969 | 0.85 |
| Burkholderia_mallei_SAVP1.2 (leading) | 840 | 994 | 0.85 | 729459 | 2601067 | 0.28 | 11453 | 16421 | 0.7 | -2346 | 5537 | -0.42 |
| Burkholderia_mallei_SAVP1.2 (lagging) | 886 | 734 | 1.21 | 793107 | 629304 | 1.26 | 205 | -1190 | -0.17 | 2176 | 5234 | 0.42 |
| Burkholderia_pseudomallei_1106a (leading) | 1093 | 1205 | 0.91 | 968235 | 1071495 | 0.9 | 20952 | 28157 | 0.74 | -2951 | 3042 | -0.97 |
| Burkholderia_pseudomallei_1106a (lagging) | 911 | 809 | 1.13 | 779262 | 674259 | 1.16 | -9766 | -9949 | 0.98 | 3988 | 4374 | 0.91 |
| Burkholderia_pseudomallei_1710b (leading) | 977 | 1041 | 0.94 | 1058133 | 1170444 | 0.9 | 19708 | 23906 | 0.82 | -2069 | 2076 | -1 |
| Burkholderia_pseudomallei_1710b (lagging) | 894 | 823 | 1.09 | 1018257 | 1010037 | 1.01 | -10679 | -12650 | 0.84 | 5836 | 2325 | 2.51 |
| Burkholderia_pseudomallei_668 (leading) | 1077 | 1171 | 0.92 | 951711 | 1019859 | 0.93 | 21132 | 26368 | 0.8 | -3221 | 1581 | -2.04 |
| Burkholderia_pseudomallei_668 (lagging) | 882 | 820 | 1.08 | 768291 | 683676 | 1.12 | -9588 | -9796 | 0.98 | 3999 | 4842 | 0.83 |
| Burkholderia_pseudomallei_K96243 (leading) | 891 | 1059 | 0.84 | 895473 | 1068744 | 0.84 | 20906 | 26793 | 0.78 | -2099 | 2305 | -0.91 |
| Burkholderia_pseudomallei_K96243 (lagging) | 812 | 636 | 1.28 | 792171 | 637318 | 1.24 | -8341 | -8730 | 0.96 | 6482 | 4149 | 1.56 |
| Burkholderia_thailandensis_E264.2 (leading) | 928 | 865 | 1.07 | 935250 | 902137 | 1.04 | 20582 | 12452 | 1.65 | -2466 | 1998 | -1.23 |
| Burkholderia_thailandensis_E264.2 (lagging) | 743 | 739 | 1.01 | 725754 | 751569 | 0.97 | -11036 | 5501 | -2.01 | 6206 | 4824 | 1.29 |
| Burkholderia_vietnamiensis_G4.8 (leading) | 955 | 914 | 1.04 | 925608 | 915225 | 1.01 | 15434 | 12416 | 1.24 | 5756 | 6547 | 0.88 |
| Burkholderia_vietnamiensis_G4.8 (lagging) | 703 | 701 | 1 | 649458 | 664223 | 0.98 | -6193 | -8719 | 0.71 | 7363 | 9646 | 0.76 |
| Burkholderia_xenovorans_LB400 (leading) | 1265 | 1288 | 0.98 | 1251459 | 1219473 | 1.03 | 28848 | 25604 | 1.13 | -743 | 5157 | -0.14 |
| Burkholderia_xenovorans_LB400 (lagging) | 934 | 942 | 0.99 | 837795 | 901236 | 0.93 | -5941 | -4759 | 1.25 | 4924 | 8839 | 0.56 |
| Caldicellulosiruptor_saccharolyticus_DSM_8903 (leading) | 974 | 727 | 1.34 | 987102 | 688668 | 1.43 | 72490 | 47277 | 1.53 | 61082 | 47127 | 1.3 |
| Caldicellulosiruptor_saccharolyticus_DSM_8903 (lagging) | 282 | 695 | 0.41 | 263724 | 624765 | 0.42 | 9900 | 39608 | 0.25 | 14760 | 40249 | 0.37 |
| Campylobacter_curvus_525_92 (leading) | 556 | 626 | 0.89 | 530682 | 553065 | 0.96 | 21455 | 23489 | 0.91 | 26609 | 31722 | 0.84 |
| Campylobacter_curvus_525_92 (lagging) | 387 | 361 | 1.07 | 324564 | 295524 | 1.1 | 5937 | 5379 | 1.1 | 25061 | 21423 | 1.17 |
| Campylobacter_fetus_82-40 (leading) | 579 | 494 | 1.17 | 542865 | 450705 | 1.2 | 33120 | 29180 | 1.14 | 25795 | 22747 | 1.13 |
| Campylobacter_fetus_82-40 (lagging) | 264 | 381 | 0.69 | 257208 | 351969 | 0.73 | 9697 | 12041 | 0.81 | 20731 | 33352 | 0.62 |
| Campylobacter_hominis_ATCC_BAA-381.2 (leading) | 482 | 459 | 1.05 | 402228 | 411771 | 0.98 | 22202 | 21422 | 1.04 | 22946 | 23877 | 0.96 |
| Campylobacter_hominis_ATCC_BAA-381.2 (lagging) | 376 | 364 | 1.03 | 314061 | 317556 | 0.99 | 11938 | 12202 | 0.98 | 24677 | 24558 | 1 |
| Campylobacter_jejuni (leading) | 491 | 500 | 0.98 | 461136 | 505674 | 0.91 | 31704 | 34749 | 0.91 | 13910 | 9575 | 1.45 |
| Campylobacter_jejuni (lagging) | 326 | 316 | 1.03 | 290037 | 274080 | 1.06 | 7878 | 6408 | 1.23 | 15109 | 13598 | 1.11 |
| Campylobacter_jejuni_81-176.2 (leading) | 499 | 506 | 0.99 | 468663 | 496386 | 0.94 | 31535 | 33917 | 0.93 | 14280 | 9399 | 1.52 |
| Campylobacter_jejuni_81-176.2 (lagging) | 328 | 319 | 1.03 | 283332 | 267948 | 1.06 | 7421 | 6472 | 1.15 | 14313 | 13038 | 1.1 |
| Campylobacter_jejuni_doylei_269_97 (leading) | 451 | 540 | 0.84 | 425340 | 482052 | 0.88 | 28345 | 33784 | 0.84 | 14301 | 12930 | 1.11 |
| Campylobacter_jejuni_doylei_269_97 (lagging) | 419 | 320 | 1.31 | 345336 | 273342 | 1.26 | 10721 | 7975 | 1.34 | 17355 | 14235 | 1.22 |
| Campylobacter_jejuni_RM1221 (leading) | 533 | 564 | 0.95 | 487803 | 527256 | 0.93 | 33544 | 35367 | 0.95 | 18909 | 13453 | 1.41 |
| Campylobacter_jejuni_RM1221 (lagging) | 390 | 350 | 1.11 | 315612 | 290598 | 1.09 | 9186 | 6730 | 1.36 | 17068 | 14906 | 1.15 |
| Candidatus_Blochmannia_floridanus (leading) | 209 | 159 | 1.31 | 210426 | 161685 | 1.3 | 17241 | 11894 | 1.45 | -1953 | -2041 | 0.96 |
| Candidatus_Blochmannia_floridanus (lagging) | 88 | 126 | 0.7 | 91242 | 122697 | 0.74 | -2605 | -881 | 2.96 | 6323 | 7560 | 0.84 |
| Candidatus_Blochmannia_pennsylvanicus_BPEN (leading) | 219 | 166 | 1.32 | 219957 | 165756 | 1.33 | 10340 | 8285 | 1.25 | 4007 | -239 | -16.77 |
| Candidatus_Blochmannia_pennsylvanicus_BPEN (lagging) | 93 | 131 | 0.71 | 96333 | 124191 | 0.78 | 998 | 19 | 52.53 | 3171 | 6120 | 0.52 |
| Candidatus_Methanoregula_boonei_6A8 (leading) | 624 | 623 | 1 | 564609 | 551634 | 1.02 | 2445 | 2545 | 0.96 | 7726 | 4645 | 1.66 |
| Candidatus_Methanoregula_boonei_6A8 (lagging) | 618 | 584 | 1.06 | 528156 | 544692 | 0.97 | -1915 | 818 | -2.34 | 12015 | 13184 | 0.91 |
| Candidatus_Pelagibacter_ubique_HTCC1062 (leading) | 334 | 352 | 0.95 | 311082 | 296967 | 1.05 | 14484 | 13583 | 1.07 | 24868 | 15794 | 1.57 |
| Candidatus_Pelagibacter_ubique_HTCC1062 (lagging) | 329 | 338 | 0.97 | 313134 | 332298 | 0.94 | 14399 | 15201 | 0.95 | 23803 | 18769 | 1.27 |
| Candidatus_Ruthia_magnifica_Cm_Calyptogena_magnifica | 301 | 274 | 1.1 | 295536 | 256488 | 1.15 | 17114 | 15187 | 1.13 | 66 | -391 | -0.17 |
| Candidatus_Ruthia_magnifica_Cm_Calyptogena_magnifica | 180 | 220 | 0.82 | 170406 | 197121 | 0.86 | 298 | 599 | 0.5 | 6156 | 7132 | 0.86 |
| Candidatus_Vesicomyosocius_okutanii_HA (leading) | 290 | 258 | 1.12 | 270843 | 238599 | 1.14 | 17482 | 16048 | 1.09 | -1397 | -2251 | 0.62 |
| Candidatus_Vesicomyosocius_okutanii_HA (lagging) | 178 | 210 | 0.85 | 168804 | 192444 | 0.88 | 544 | -305 | -1.78 | 7332 | 9945 | 0.74 |
| Carboxydothermus_hydrogenoformans_Z-2901 (leading) | 1122 | 1136 | 0.99 | 977791 | 946890 | 1.03 | 68634 | 67073 | 1.02 | 24972 | 23198 | 1.08 |
| Carboxydothermus_hydrogenoformans_Z-2901 (lagging) | 180 | 181 | 0.99 | 124989 | 121770 | 1.03 | -491 | 438 | -1.12 | 1940 | 3790 | 0.51 |
| Caulobacter_crescentus (leading) | 968 | 1075 | 0.9 | 1011261 | 992814 | 1.02 | -1443 | 1903 | -0.76 | -1218 | -4847 | 0.25 |

| Chromosome | Nu of genes 1st half (Nu1) | Nu of genes 2nd half (Nu2) | Nu1/Nu2 | Total length of genes 1st half (Length1) | Total length of genes 2nd half (Length2) | Length1/ Length2 | Cumulative skew (G-C) of genes 1st half (G-C)1 | Cumulative skew (G-C) of genes 2nd half (G-C)2 | (G-C)1/ (G-C)2 | Cumulative skew (A-T) of genes 1st half (A-T)1 | Cumulative skew (A-T) of genes 2nd half (A-T)2 | (A-T)1/ (A-T)2 |
|---|---|---|---|---|---|---|---|---|---|---|---|---|
| Caulobacter_crescentus (lagging) | 834 | 859 | 0.97 | 818598 | 815166 | 1 | -17145 | -18351 | 0.93 | 3263 | 4913 | 0.66 |
| Chlamydia_muridarum.2 (leading) | 237 | 259 | 0.92 | 272802 | 255234 | 1.07 | 17765 | 17336 | 1.02 | -6472 | -7641 | 0.85 |
| Chlamydia_muridarum.2 (lagging) | 191 | 216 | 0.88 | 206094 | 230229 | 0.9 | -8998 | -8961 | 1 | -10 | -1626 | 0.01 |
| Chlamydia_trachomatis (leading) | 229 | 222 | 1.03 | 797578 | 226618 | 3.52 | 413 | 1640 | 0.25 | -3538 | -3182 | 1.11 |
| Chlamydia_trachomatis (lagging) | 231 | 212 | 1.09 | 232749 | 232557 | 1 | 6816 | 6792 | 1 | -3757 | -3041 | 1.24 |
| Chlamydia_trachomatis_A_HAR-13 (leading) | 240 | 225 | 1.07 | 245913 | 229945 | 1.07 | 371 | 1648 | 0.23 | -3560 | -3303 | 1.08 |
| Chlamydia_trachomatis_A_HAR-13 (lagging) | 232 | 213 | 1.09 | 229449 | 233745 | 0.98 | 6566 | 6784 | 0.97 | -3631 | -3137 | 1.16 |
| Chlamydophila_abortus_S26_3 (leading) | 256 | 236 | 1.08 | 261964 | 255450 | 1.03 | 11152 | 10595 | 1.05 | -3431 | -4923 | 0.7 |
| Chlamydophila_abortus_S26_3 (lagging) | 218 | 221 | 0.99 | 240963 | 243699 | 0.99 | -5739 | -7345 | 0.78 | 3258 | 2376 | 1.37 |
| Chlamydophila_caviae (leading) | 273 | 250 | 1.09 | 271137 | 262620 | 1.03 | 12034 | 12345 | 0.97 | -4031 | -5847 | 0.69 |
| Chlamydophila_caviae (lagging) | 232 | 242 | 0.96 | 252204 | 263145 | 0.96 | -5617 | -6115 | 0.92 | 3701 | 3748 | 0.99 |
| Chlamydophila_felis_Fe_C-56 (leading) | 250 | 263 | 0.95 | 267312 | 267765 | 1 | 12457 | 12054 | 1.03 | -5923 | -3975 | 1.49 |
| Chlamydophila_felis_Fe_C-56 (lagging) | 246 | 245 | 1 | 263238 | 264555 | 1 | -7508 | -7230 | 1.04 | 3838 | 4339 | 0.88 |
| Chlamydophila_pneumoniae_AR39 (leading) | 304 | 294 | 1.03 | 285939 | 297690 | 0.96 | 10585 | 12899 | 0.82 | -5204 | -4113 | 1.27 |
| Chlamydophila_pneumoniae_AR39 (lagging) | 265 | 248 | 1.07 | 264138 | 247770 | 1.07 | -9853 | -7501 | 1.31 | 3243 | 1964 | 1.65 |
| Chlamydophila_pneumoniae_CWL029 (leading) | 274 | 289 | 0.95 | 304746 | 301000 | 1.01 | -1361 | -1326 | 1.03 | 2633 | 1534 | 1.72 |
| Chlamydophila_pneumoniae_CWL029 (lagging) | 241 | 247 | 0.98 | 230082 | 252579 | 0.91 | 4390 | 4075 | 1.08 | -4800 | -3290 | 1.46 |
| Chlamydophila_pneumoniae_J138 (leading) | 285 | 293 | 0.97 | 315981 | 302238 | 1.05 | -1429 | -1289 | 1.11 | 2818 | 1591 | 1.77 |
| Chlamydophila_pneumoniae_J138 (lagging) | 242 | 248 | 0.98 | 233928 | 249156 | 0.94 | 4431 | 3584 | 1.24 | -4770 | -3202 | 1.49 |
| Chlamydophila_pneumoniae_TW_183 (leading) | 291 | 305 | 0.95 | 316152 | 303399 | 1.04 | -1465 | -1405 | 1.04 | 2573 | 1290 | 1.99 |
| Chlamydophila_pneumoniae_TW_183 (lagging) | 259 | 257 | 1.01 | 235986 | 251505 | 0.94 | 4436 | 3774 | 1.18 | -4674 | -3319 | 1.41 |
| Chlorobium_chlorochromatii_CaD3 (leading) | 545 | 566 | 0.96 | 732048 | 659586 | 1.11 | 30357 | 20670 | 1.47 | -7023 | -5724 | 1.23 |
| Chlorobium_chlorochromatii_CaD3 (lagging) | 387 | 503 | 0.77 | 406620 | 473841 | 0.86 | 4590 | 2813 | 1.63 | 1782 | 6432 | 0.28 |
| Chlorobium_phaeobacteroides_DSM_266 (leading) | 706 | 882 | 0.8 | 712101 | 897195 | 0.79 | 27700 | 42389 | 0.65 | -8361 | -3100 | 2.7 |
| Chlorobium_phaeobacteroides_DSM_266 (lagging) | 617 | 444 | 1.39 | 631644 | 394638 | 1.6 | -7821 | -9110 | 0.86 | 23123 | 18116 | 1.28 |
| Chlorobium_tepidum_TLS (leading) | 641 | 628 | 1.02 | 553584 | 506211 | 1.09 | 7622 | 9163 | 0.83 | -266 | -143 | 1.86 |
| Chlorobium_tepidum_TLS (lagging) | 492 | 490 | 1 | 400083 | 437376 | 0.91 | -8735 | -14087 | 0.62 | 13442 | 20471 | 0.66 |
| Chromobacterium_violaceum (leading) | 1180 | 1282 | 0.92 | 1124052 | 1239228 | 0.91 | 25496 | 33384 | 0.76 | 9140 | 7842 | 1.17 |
| Chromobacterium_violaceum (lagging) | 1023 | 921 | 1.11 | 970122 | 873507 | 1.11 | -23818 | -17686 | 1.35 | 24298 | 19529 | 1.24 |
| Chromohalobacter_salexigens_DSM_3043 (leading) | 917 | 878 | 1.04 | 943848 | 879834 | 1.07 | 19511 | 14839 | 1.31 | -6269 | -7345 | 0.85 |
| Chromohalobacter_salexigens_DSM_3043 (lagging) | 697 | 805 | 0.87 | 710319 | 774921 | 0.92 | -26980 | -19642 | 1.37 | 3635 | 2349 | 1.55 |
| Clavibacter_michiganensis_NCPPB_382.3 (leading) | 830 | 887 | 0.94 | 847188 | 871779 | 0.97 | -30366 | -32675 | 0.93 | -2886 | 1988 | -1.45 |
| Clavibacter_michiganensis_NCPPB_382.3 (lagging) | 631 | 635 | 0.99 | 620553 | 620061 | 1 | -25498 | -25647 | 0.99 | -1477 | -1794 | 0.82 |
| Clostridium_acetobutylicum.2 (leading) | 1437 | 1453 | 0.99 | 1338552 | 1337733 | 1 | 117279 | 117593 | 1 | 111611 | 114502 | 0.97 |
| Clostridium_acetobutylicum.2 (lagging) | 359 | 422 | 0.85 | 330915 | 374493 | 0.88 | 6600 | 6570 | 1 | 19381 | 22003 | 0.88 |
| Clostridium_beijerinckii_NCIMB_8052 (leading) | 2147 | 1994 | 1.08 | 2058027 | 1949475 | 1.06 | 179245 | 160892 | 1.11 | 191862 | 160501 | 1.2 |
| Clostridium_beijerinckii_NCIMB_8052 (lagging) | 404 | 474 | 0.85 | 347049 | 411363 | 0.84 | 9836 | 11592 | 0.85 | 16847 | 20603 | 0.82 |
| Clostridium_botulinum_A (leading) | 1417 | 1536 | 0.92 | 1242791 | 1387698 | 0.9 | 100679 | 115310 | 0.87 | 120833 | 151617 | 0.8 |
| Clostridium_botulinum_A (lagging) | 352 | 268 | 1.31 | 291753 | 234003 | 1.25 | 11320 | 7996 | 1.42 | 18287 | 14253 | 1.28 |
| Clostridium_botulinum_A_ATCC_19397 (leading) | 1329 | 1551 | 0.86 | 1190580 | 1384566 | 0.86 | 96276 | 115336 | 0.83 | 114496 | 152784 | 0.75 |
| Clostridium_botulinum_A_ATCC_19397 (lagging) | 395 | 276 | 1.43 | 330792 | 230604 | 1.43 | 13876 | 7647 | 1.81 | 22098 | 14613 | 1.51 |
| Clostridium_botulinum_A_Hall (leading) | 1313 | 1471 | 0.89 | 1177452 | 1339869 | 0.88 | 95329 | 110953 | 0.86 | 113531 | 145584 | 0.78 |
| Clostridium_botulinum_A_Hall (lagging) | 351 | 271 | 1.3 | 290142 | 224625 | 1.29 | 11200 | 7599 | 1.47 | 18120 | 13584 | 1.33 |
| Clostridium_botulinum_F_Langeland (leading) | 1372 | 1610 | 0.85 | 1247781 | 1423587 | 0.88 | 99668 | 118891 | 0.84 | 119101 | 157000 | 0.76 |
| Clostridium_botulinum_F_Langeland (lagging) | 376 | 276 | 1.36 | 319806 | 233862 | 1.37 | 13664 | 8143 | 1.68 | 21252 | 14807 | 1.44 |
| Clostridium_dicile_630.ffn.2 (leading) | 1538 | 1484 | 1.04 | 1455903 | 1453227 | 1 | 121250 | 119102 | 1.02 | 134717 | 131918 | 1.02 |
| Clostridium_dicile_630.ffn.2 (lagging) | 359 | 360 | 1 | 321341 | 325591 | 0.99 | 11068 | 15108 | 0.73 | 18939 | 20973 | 0.9 |
| Clostridium_kluyveri_DSM_555.2 (leading) | 1264 | 1533 | 0.82 | 1177488 | 1318191 | 0.89 | 98914 | 102812 | 0.96 | 96776 | 101145 | 0.96 |
| Clostridium_kluyveri_DSM_555.2 (lagging) | 581 | 459 | 1.27 | 506268 | 344922 | 1.47 | 24427 | 8893 | 2.75 | 37597 | 24123 | 1.56 |
| Clostridium_novyi_NT (leading) | 989 | 920 | 1.08 | 967509 | 869664 | 1.11 | 78125 | 73091 | 1.07 | 106962 | 97287 | 1.1 |
| Clostridium_novyi_NT (lagging) | 188 | 227 | 0.83 | 166812 | 211383 | 0.79 | 3745 | 5096 | 0.73 | 10071 | 14491 | 0.69 |
| Clostridium_perfringens.2 (leading) | 974 | 1150 | 0.85 | 934524 | 1109916 | 0.84 | 83752 | 102759 | 0.82 | 77638 | 112457 | 0.69 |
| Clostridium_perfringens.2 (lagging) | 311 | 224 | 1.39 | 296583 | 188988 | 1.57 | 15603 | 5900 | 2.64 | 19554 | 8398 | 2.33 |
| Clostridium_perfringens_ATCC_13124 (leading) | 1084 | 1220 | 0.89 | 1041348 | 1179316 | 0.88 | 94400 | 108488 | 0.87 | 88884 | 118967 | 0.75 |
| Clostridium_perfringens_ATCC_13124 (lagging) | 329 | 242 | 1.36 | 276612 | 209415 | 1.32 | 12031 | 6253 | 1.92 | 16377 | 10076 | 1.63 |
| Clostridium_perfringens_SM101 (leading) | 915 | 1097 | 0.83 | 844890 | 1047070 | 0.81 | 73218 | 95676 | 0.77 | 70838 | 106961 | 0.66 |
| Clostridium_perfringens_SM101 (lagging) | 337 | 208 | 1.62 | 288486 | 177876 | 1.62 | 13540 | 5607 | 2.41 | 18226 | 8275 | 2.2 |
| Clostridium_tetani_E88 (leading) | 885 | 1018 | 0.87 | 931719 | 1034172 | 0.9 | 78726 | 85776 | 0.92 | 110601 | 116368 | 0.95 |
| Clostridium_tetani_E88 (lagging) | 267 | 202 | 1.32 | 260301 | 171963 | 1.51 | 9932 | 4502 | 2.21 | 15477 | 6503 | 2.38 |
| Clostridium_thermocellum_ATCC_27405 (leading) | 764 | 795 | 0.96 | 804063 | 791922 | 1.02 | 50949 | 51613 | 0.99 | 50336 | 56423 | 0.89 |
| Clostridium_thermocellum_ATCC_27405 (lagging) | 809 | 822 | 0.98 | 829386 | 792333 | 1.05 | 58808 | 56358 | 1.04 | 54078 | 55527 | 0.97 |
| Colwellia_psychrerythraea_34H (leading) | 1479 | 1352 | 1.09 | 1359237 | 1364410 | 1 | 52930 | 52608 | 1.01 | 12541 | 11373 | 1.1 |
| Colwellia_psychrerythraea_34H (lagging) | 1035 | 1043 | 0.99 | 891021 | 936579 | 0.95 | 13168 | 13491 | 0.98 | 19227 | 20910 | 0.92 |
| Corynebacterium_diphtheriae (leading) | 747 | 675 | 1.11 | 692475 | 698661 | 0.99 | 11162 | 16536 | 0.68 | -4629 | -5591 | 0.83 |
| Corynebacterium_diphtheriae (lagging) | 440 | 409 | 1.08 | 407338 | 390537 | 1.04 | -14968 | -8611 | 1.74 | 136 | -1186 | -0.11 |
| Corynebacterium_iciens_YS-314.ffn (leading) | 877 | 838 | 1.05 | 853515 | 841659 | 1.01 | -10456 | -13072 | 0.8 | -1855 | 903 | -2.05 |
| Corynebacterium_iciens_YS-314.ffn (lagging) | 628 | 606 | 1.04 | 598260 | 595449 | 1 | -20928 | -18747 | 1.12 | -2126 | -1980 | 1.07 |

| Chromosome | Nu of genes 1st half (Nu1) | Nu of genes 2nd half (Nu2) | Nu1/Nu2 | Total length of genes 1st half (Length1) | Total length of genes 2nd half (Length2) | Length1/ Length2 | Cumulative skew (G-C) of genes 1st half (G-C)1 | Cumulative skew (G-C) of genes 2nd half (G-C)2 | (G-C)1/ (G-C)2 | Cumulative skew (A-T) of genes 1st half (A-T)1 | Cumulative skew (A-T) of genes 2nd half (A-T)2 | (A-T)1/ (A-T)2 |
|---|---|---|---|---|---|---|---|---|---|---|---|---|
| Corynebacterium_glutamicum_ATCC_13032_Bielefeld (lea | 889 | 904 | 0.98 | 814524 | 871668 | 0.93 | 6199 | 8424 | 0.74 | -5805 | -2122 | 2.74 |
| Corynebacterium_glutamicum_ATCC_13032_Bielefeld (lag | 652 | 611 | 1.07 | 631428 | 555783 | 1.14 | -16875 | -18870 | 0.89 | -2877 | 453 | -6.35 |
| Corynebacterium_glutamicum_ATCC_13032_Kitasato (lea | 863 | 869 | 0.99 | 808092 | 855126 | 0.94 | 6137 | 8548 | 0.72 | -5741 | -2262 | 2.54 |
| Corynebacterium_glutamicum_ATCC_13032_Kitasato (lag | 641 | 619 | 1.04 | 635997 | 559926 | 1.14 | -17075 | -19704 | 0.87 | -2384 | 710 | -3.36 |
| Corynebacterium_glutamicum_R (leading) | 893 | 930 | 0.96 | 839043 | 889785 | 0.94 | 6023 | 5883 | 1.02 | -6056 | -4736 | 1.28 |
| Corynebacterium_glutamicum_R (lagging) | 632 | 596 | 1.06 | 582681 | 566166 | 1.03 | -18408 | -16903 | 1.09 | -3193 | -1452 | 2.2 |
| Corynebacterium_jeikeium_K411.2 (leading) | 589 | 678 | 0.87 | 604449 | 713025 | 0.85 | 7727 | 15812 | 0.49 | 6926 | 8815 | 0.79 |
| Corynebacterium_jeikeium_K411.2 (lagging) | 454 | 382 | 1.19 | 490437 | 394455 | 1.24 | -9556 | -8331 | 1.15 | 11751 | 8092 | 1.45 |
| Coxiella_burnetii (leading) | 608 | 564 | 1.08 | 525384 | 486105 | 1.08 | 18957 | 16813 | 1.13 | 2835 | 2176 | 1.3 |
| Coxiella_burnetii (lagging) | 395 | 448 | 0.88 | 308979 | 372669 | 0.83 | -643 | -2134 | 0.3 | 2788 | 4425 | 0.63 |
| Cyanobacteria_bacterium_Yellowstone_A-Prime (leading) | 653 | 687 | 0.95 | 586257 | 626973 | 0.94 | 3261 | 3154 | 1.03 | -6966 | -8169 | 0.85 |
| Cyanobacteria_bacterium_Yellowstone_A-Prime (lagging) | 731 | 688 | 1.06 | 647280 | 626583 | 1.03 | 1921 | 3235 | 0.59 | -7437 | -9256 | 0.8 |
| Cyanobacteria_bacterium_Yellowstone_B-Prime (leading) | 691 | 702 | 0.98 | 626415 | 661728 | 0.95 | 4994 | 7941 | 0.63 | -9807 | -9147 | 1.07 |
| Cyanobacteria_bacterium_Yellowstone_B-Prime (lagging) | 723 | 745 | 0.97 | 674871 | 637188 | 1.06 | 5774 | 7552 | 0.76 | -12409 | -7758 | 1.6 |
| Cytophaga_hutchinsonii_ATCC_33406 (leading) | 1002 | 1018 | 0.98 | 1092186 | 1085868 | 1.01 | 35680 | 38060 | 0.94 | 36860 | 37134 | 0.99 |
| Cytophaga_hutchinsonii_ATCC_33406 (lagging) | 867 | 897 | 0.97 | 894165 | 921873 | 0.97 | 11629 | 13227 | 0.88 | 34350 | 39840 | 0.86 |
| Dechloromonas_aromatica_RCB (leading) | 1172 | 1112 | 1.05 | 1185855 | 1106610 | 1.07 | 23938 | 17307 | 1.38 | -17195 | -11533 | 1.49 |
| Dechloromonas_aromatica_RCB (lagging) | 906 | 980 | 0.92 | 868326 | 966414 | 0.9 | -32905 | -40892 | 0.8 | 16715 | 16772 | 1 |
| Dehalococcoides_BAV1 (leading) | 311 | 333 | 0.93 | 270915 | 287643 | 0.94 | 9500 | 10838 | 0.88 | 2257 | 1119 | 2.02 |
| Dehalococcoides_BAV1 (lagging) | 362 | 364 | 0.99 | 332061 | 312807 | 1.06 | 3646 | 1396 | 2.61 | 12061 | 9111 | 1.32 |
| Dehalococcoides_CBDB1 (leading) | 362 | 396 | 0.91 | 324252 | 325341 | 1 | 13717 | 12185 | 1.13 | 419 | -778 | -0.54 |
| Dehalococcoides_CBDB1 (lagging) | 353 | 346 | 1.02 | 317484 | 288786 | 1.1 | -919 | 154 | -5.97 | 12925 | 11204 | 1.15 |
| Dehalococcoides_ethenogenes_195 (leading) | 390 | 419 | 0.93 | 329611 | 330825 | 1 | 14234 | 11209 | 1.27 | -442 | 1628 | -0.27 |
| Dehalococcoides_ethenogenes_195 (lagging) | 383 | 387 | 0.99 | 335259 | 324636 | 1.03 | -804 | 2563 | -0.31 | 13951 | 11833 | 1.18 |
| Deinococcus_geothermalis_DSM_11300.2 (leading) | 604 | 594 | 1.02 | 574581 | 541443 | 1.06 | 4990 | 5827 | 0.86 | -4283 | -2556 | 1.68 |
| Deinococcus_geothermalis_DSM_11300.2 (lagging) | 550 | 586 | 0.94 | 543840 | 569757 | 0.95 | 1743 | 365 | 4.78 | 5349 | 5812 | 0.92 |
| Deinococcus_radiodurans.3 (leading) | 712 | 667 | 1.07 | 633272 | 594009 | 1.07 | -5957 | -4532 | 1.31 | 5225 | 5377 | 0.97 |
| Deinococcus_radiodurans.3 (lagging) | 603 | 646 | 0.93 | 565652 | 595773 | 0.95 | -4552 | -4370 | 1.04 | 10104 | 9486 | 1.07 |
| Desulfitobacterium_hafniense_Y51 (leading) | 1339 | 2090 | 0.64 | 1308147 | 2012967 | 0.65 | 47658 | 96260 | 0.5 | 15405 | 24759 | 0.62 |
| Desulfitobacterium_hafniense_Y51 (lagging) | 1158 | 472 | 2.45 | 1114185 | 425829 | 2.62 | 32506 | -6242 | -5.21 | 10181 | 3243 | 3.14 |
| Desulfotalea_psychrophila_LSv54 (leading) | 824 | 806 | 1.02 | 796623 | 805311 | 0.99 | 19379 | 29292 | 0.66 | -10024 | -11111 | 0.9 |
| Desulfotalea_psychrophila_LSv54 (lagging) | 753 | 732 | 1.03 | 696633 | 716916 | 0.97 | 6016 | -1260 | -4.77 | -2267 | 16296 | -0.14 |
| Desulfotomaculum_reducens_MI-1 (leading) | 1273 | 1226 | 1.04 | 1192635 | 1149126 | 1.04 | 62782 | 59659 | 1.05 | 38393 | 33307 | 1.15 |
| Desulfotomaculum_reducens_MI-1 (lagging) | 352 | 424 | 0.83 | 297561 | 367704 | 0.81 | 3727 | 9681 | 0.38 | 7442 | 7999 | 0.93 |
| Desulfovibrio_desulfuricans_G20 (leading) | 944 | 944 | 1 | 882348 | 787488 | 1.12 | 39055 | 31847 | 1.23 | -9125 | -6875 | 1.33 |
| Desulfovibrio_desulfuricans_G20 (lagging) | 865 | 1021 | 0.85 | 807039 | 898779 | 0.9 | -12562 | -12018 | 1.05 | 20597 | 25661 | 0.8 |
| Desulfovibrio_vulgaris_DP4.2 (leading) | 670 | 738 | 0.91 | 690357 | 696816 | 0.99 | 6280 | 8296 | 0.76 | -8283 | -8396 | 0.99 |
| Desulfovibrio_vulgaris_DP4.2 (lagging) | 755 | 777 | 0.97 | 784389 | 798465 | 0.98 | -38713 | -39548 | 0.98 | 16650 | 14919 | 1.12 |
| Desulfovibrio_vulgaris_Hildenborough (leading) | 814 | 842 | 0.97 | 717471 | 753849 | 0.95 | 8777 | 8227 | 1.07 | -9168 | -8360 | 1.1 |
| Desulfovibrio_vulgaris_Hildenborough (lagging) | 888 | 834 | 1.06 | 832641 | 767413 | 1.08 | -39077 | -39036 | 1 | 15570 | 17062 | 0.91 |
| Dichelobacter_nodosus_VCS1703A (leading) | 338 | 385 | 0.88 | 346239 | 360261 | 0.96 | 14707 | 18865 | 0.78 | -1560 | 471 | -3.31 |
| Dichelobacter_nodosus_VCS1703A (lagging) | 280 | 276 | 1.01 | 284376 | 264360 | 1.08 | 3737 | 2081 | 1.8 | 1953 | 1431 | 1.36 |
| Ehrlichia_canis_Jake (leading) | 235 | 296 | 0.79 | 292002 | 287448 | 1.02 | 26112 | 26582 | 0.98 | 1338 | -2090 | -0.64 |
| Ehrlichia_canis_Jake (lagging) | 201 | 192 | 1.05 | 189930 | 177897 | 1.07 | -463 | 416 | -1.11 | 21275 | 17407 | 1.22 |
| Ehrlichia_cheensis_Arkansas.ffn (leading) | 341 | 295 | 1.16 | 306663 | 256821 | 1.19 | 27843 | 22870 | 1.22 | -624 | -1025 | 0.61 |
| Ehrlichia_cheensis_Arkansas.ffn (lagging) | 212 | 256 | 0.83 | 162891 | 209206 | 0.78 | -819 | -271 | 3.02 | 16000 | 19920 | 0.8 |
| Ehrlichia_ruminantium_Gardel (leading) | 233 | 268 | 0.87 | 279177 | 270861 | 1.03 | 21944 | 24548 | 0.89 | 165 | -2801 | -0.06 |
| Ehrlichia_ruminantium_Gardel (lagging) | 228 | 220 | 1.04 | 214719 | 192792 | 1.11 | -446 | 1234 | -0.36 | 20099 | 17468 | 1.15 |
| Ehrlichia_ruminantium_str._Welgevonden (leading) | 239 | 267 | 0.9 | 280728 | 270753 | 1.04 | 21949 | 24691 | 0.89 | 639 | -2638 | -0.24 |
| Ehrlichia_ruminantium_str._Welgevonden (lagging) | 234 | 217 | 1.08 | 216543 | 189294 | 1.14 | -301 | 1296 | -0.23 | 20104 | 16994 | 1.18 |
| Ehrlichia_ruminantium_Welgevonden (leading) | 219 | 253 | 0.87 | 279192 | 266760 | 1.05 | 21668 | 24385 | 0.89 | 1626 | -2237 | -0.73 |
| Ehrlichia_ruminantium_Welgevonden (lagging) | 219 | 196 | 1.12 | 212533 | 181116 | 1.17 | -490 | 1262 | -0.39 | 20104 | 16236 | 1.24 |
| Enterobacter_638.2 (leading) | 1164 | 1243 | 0.94 | 1138197 | 1191810 | 0.96 | 38374 | 40280 | 0.95 | 2117 | -2220 | -0.95 |
| Enterobacter_638.2 (lagging) | 903 | 804 | 1.12 | 847458 | 790206 | 1.07 | 11031 | 10649 | 1.04 | 1305 | 1057 | 1.23 |
| Enterococcus_faecalis_V583 (leading) | 1177 | 1288 | 0.91 | 1071870 | 1188411 | 0.9 | 54007 | 56137 | 0.96 | 50397 | 56862 | 0.89 |
| Enterococcus_faecalis_V583 (lagging) | 371 | 276 | 1.34 | 304128 | 207837 | 1.46 | 2630 | 957 | 2.75 | 7290 | 5174 | 1.41 |
| Erwinia_carotovora_atroseptica_SCRI1043 (leading) | 1214 | 1333 | 0.91 | 1190820 | 1286643 | 0.93 | 58767 | 64494 | 0.91 | -8897 | -15449 | 0.58 |
| Erwinia_carotovora_atroseptica_SCRI1043 (lagging) | 976 | 948 | 1.03 | 982995 | 891114 | 1.1 | -6000 | 453 | -13.25 | 11884 | 11513 | 1.03 |
| Erythrobacter_litoralis_HTCC2594 (leading) | 739 | 772 | 0.96 | 717345 | 2392599 | 0.3 | 7767 | 5040 | 1.54 | 9340 | 9999 | 0.93 |
| Erythrobacter_litoralis_HTCC2594 (lagging) | 728 | 771 | 0.94 | 678939 | 745083 | 0.91 | -11 | 629 | -0.02 | 9252 | 11544 | 0.8 |
| Escherichia_coli_536 (leading) | 1170 | 1249 | 0.94 | 1086657 | 1184508 | 0.92 | 34140 | 38304 | 0.89 | -643 | 1032 | -0.62 |
| Escherichia_coli_536 (lagging) | 1143 | 1066 | 1.07 | 1094409 | 971472 | 1.13 | 27309 | 22142 | 1.23 | -1610 | 1222 | -1.32 |
| Escherichia_coli_APEC_O1 (leading) | 1125 | 1245 | 0.9 | 1142913 | 1226436 | 0.93 | 38765 | 39233 | 0.99 | 1126 | 2845 | 0.4 |
| Escherichia_coli_APEC_O1 (lagging) | 1086 | 1000 | 1.09 | 1080588 | 991047 | 1.09 | 26711 | 23888 | 1.12 | 1001 | 1156 | 0.87 |
| Escherichia_coli_CFT073 (leading) | 1388 | 1436 | 0.97 | 1224141 | 1314877 | 0.93 | 41413 | 42649 | 0.97 | 2077 | -332 | -6.26 |
| Escherichia_coli_CFT073 (lagging) | 1310 | 1244 | 1.05 | 1127109 | 1076928 | 1.05 | 25413 | 22707 | 1.12 | -317 | 1030 | -0.31 |
| Escherichia_coli_K12 (leading) | 1102 | 1111 | 0.99 | 1069749 | 1104019 | 0.97 | 34662 | 35352 | 0.98 | -199 | -696 | 0.29 |

| Chromosome | Nu of genes 1st half (Nu1) | Nu of genes 2nd half (Nu2) | Nu1/Nu2 | Total length of genes 1st half (Length1) | Total length of genes 2nd half (Length2) | Length1/ Length2 | Cumulative skew (G-C) of genes 1st half (G-C)1 | Cumulative skew (G-C) of genes 2nd half (G-C)2 | (G-C)1/ (G-C)2 | Cumulative skew (A-T) of genes 1st half (A-T)1 | Cumulative skew (A-T) of genes 2nd half (A-T)2 | (A-T)1/ (A-T)2 |
|---|---|---|---|---|---|---|---|---|---|---|---|---|
| Escherichia_coli_K12 (lagging) | 1072 | 981 | 1.09 | 1016339 | 934202 | 1.09 | 24334 | 20474 | 1.19 | 1633 | 1858 | 0.88 |
| Escherichia_coli_O157H7.3 (leading) | 1484 | 1373 | 1.08 | 1323393 | 1304617 | 1.01 | 51907 | 45282 | 1.15 | 11050 | 2602 | 4.25 |
| Escherichia_coli_O157H7.3 (lagging) | 1238 | 1157 | 1.07 | 1046361 | 1077354 | 0.97 | 26519 | 24075 | 1.1 | 10824 | 4419 | 2.45 |
| Escherichia_coli_O157H7_EDL933 (leading) | 1506 | 1410 | 1.07 | 1337851 | 1338127 | 1 | 50108 | 47174 | 1.06 | 10947 | 1971 | 5.55 |
| Escherichia_coli_O157H7_EDL933 (lagging) | 1260 | 1147 | 1.1 | 1073991 | 1093080 | 0.98 | 30126 | 23124 | 1.3 | 10278 | 4797 | 2.14 |
| Escherichia_coli_UTI89.2 (leading) | 1271 | 1385 | 0.92 | 1166109 | 1282041 | 0.91 | 38945 | 41498 | 0.94 | 92 | 1823 | 0.05 |
| Escherichia_coli_UTI89.2 (lagging) | 1247 | 1140 | 1.09 | 1148262 | 1012902 | 1.13 | 25953 | 22804 | 1.14 | 65 | 56 | 1.16 |
| Escherichia_coli_W3110 (leading) | 1090 | 1066 | 1.02 | 1041249 | 1064539 | 0.98 | 33858 | 31589 | 1.07 | -849 | -699 | 1.21 |
| Escherichia_coli_W3110 (lagging) | 1063 | 1006 | 1.06 | 980661 | 943506 | 1.04 | 24170 | 23324 | 1.04 | 1557 | 1144 | 1.36 |
| Fervidobacterium_nodosum_Rt17-B1 (leading) | 403 | 454 | 0.89 | 400101 | 464184 | 0.86 | 21296 | 23059 | 0.92 | 26239 | 37213 | 0.71 |
| Fervidobacterium_nodosum_Rt17-B1 (lagging) | 475 | 417 | 1.14 | 485565 | 410547 | 1.18 | 27776 | 22721 | 1.22 | 31169 | 26674 | 1.17 |
| Flavobacterium_johnsoniae_UW101 (leading) | 1241 | 1288 | 0.96 | 1387554 | 1423242 | 0.97 | 48592 | 57401 | 0.85 | 78526 | 79731 | 0.98 |
| Flavobacterium_johnsoniae_UW101 (lagging) | 1310 | 1177 | 1.11 | 1295109 | 1217247 | 1.06 | 40460 | 23389 | 1.73 | 63589 | 69640 | 0.91 |
| Flavobacterium_psychrophilum_JIP02_86 (leading) | 544 | 768 | 0.71 | 581382 | 749013 | 0.78 | 13359 | 20817 | 0.64 | 39645 | 50628 | 0.78 |
| Flavobacterium_psychrophilum_JIP02_86 (lagging) | 649 | 450 | 1.44 | 633648 | 455475 | 1.39 | 8490 | 5154 | 1.65 | 52954 | 37237 | 1.42 |
| Francisella_tularensis_FSC_198 (leading) | 555 | 430 | 1.29 | 516042 | 423411 | 1.22 | 32146 | 24331 | 1.32 | 10904 | 10360 | 1.05 |
| Francisella_tularensis_FSC_198 (lagging) | 290 | 329 | 0.88 | 255559 | 302668 | 0.84 | 3988 | 4904 | 0.81 | 9460 | 15431 | 0.61 |
| Francisella_tularensis_holarctica (leading) | 502 | 539 | 0.93 | 450432 | 489339 | 0.92 | 26787 | 25989 | 1.03 | 12232 | 11655 | 1.05 |
| Francisella_tularensis_holarctica (lagging) | 372 | 340 | 1.09 | 324583 | 296431 | 1.09 | 6035 | 8917 | 0.68 | 14187 | 8742 | 1.62 |
| Francisella_tularensis_holarctica_OSU18 (leading) | 467 | 463 | 1.01 | 421093 | 437202 | 0.96 | 25180 | 23435 | 1.07 | 11362 | 11099 | 1.02 |
| Francisella_tularensis_holarctica_OSU18 (lagging) | 320 | 304 | 1.05 | 289385 | 265519 | 1.09 | 5063 | 8222 | 0.62 | 12844 | 8620 | 1.49 |
| Francisella_tularensis_novicida_U112 (leading) | 530 | 536 | 0.99 | 528462 | 554535 | 0.95 | 30299 | 34988 | 0.87 | 12751 | 12431 | 1.03 |
| Francisella_tularensis_novicida_U112 (lagging) | 345 | 307 | 1.12 | 325875 | 299430 | 1.09 | 5475 | 3404 | 1.61 | 10384 | 14422 | 0.72 |
| Francisella_tularensis_tularensis (leading) | 554 | 429 | 1.29 | 515484 | 420381 | 1.23 | 32096 | 24170 | 1.33 | 10994 | 10289 | 1.07 |
| Francisella_tularensis_tularensis (lagging) | 290 | 329 | 0.88 | 255559 | 302557 | 0.84 | 3987 | 4873 | 0.82 | 9457 | 15445 | 0.61 |
| Francisella_tularensis_WY96-3418 (leading) | 461 | 507 | 0.91 | 447324 | 466788 | 0.96 | 26504 | 27183 | 0.98 | 9266 | 12528 | 0.74 |
| Francisella_tularensis_WY96-3418 (lagging) | 350 | 315 | 1.11 | 315908 | 288060 | 1.1 | 6580 | 6178 | 1.07 | 14819 | 9866 | 1.5 |
| Frankia_alni_ACN14a (leading) | 1819 | 1827 | 1 | 1787856 | 1812453 | 0.99 | -5990 | -11805 | 0.51 | -24176 | -25460 | 0.95 |
| Frankia_alni_ACN14a (lagging) | 1548 | 1516 | 1.02 | 1474164 | 1418526 | 1.04 | -38617 | -36919 | 1.05 | -3569 | -2995 | 1.19 |
| Frankia_CcI3 (leading) | 1273 | 1273 | 1 | 1257147 | 1371927 | 0.92 | 9090 | 12732 | 0.71 | -21717 | -23193 | 0.94 |
| Frankia_CcI3 (lagging) | 1042 | 910 | 1.15 | 1042233 | 945531 | 1.1 | -25024 | -20706 | 1.21 | -455 | -741 | 0.61 |
| Fusobacterium_nucleatum (leading) | 457 | 540 | 0.85 | 421341 | 1664476 | 0.25 | 30826 | 37020 | 0.83 | 36071 | 42494 | 0.85 |
| Fusobacterium_nucleatum (lagging) | 563 | 506 | 1.11 | 527271 | 478764 | 1.1 | 40680 | 31802 | 1.28 | 53691 | 38444 | 1.4 |
| Geobacillus_kaustophilus_HTA426.2 (leading) | 1334 | 1418 | 0.94 | 1184070 | 1276173 | 0.93 | 64476 | 72194 | 0.89 | 21266 | 19935 | 1.07 |
| Geobacillus_kaustophilus_HTA426.2 (lagging) | 411 | 334 | 1.23 | 304365 | 250560 | 1.21 | 37 | -130 | -0.28 | 7320 | 6696 | 1.09 |
| Geobacillus_thermodenitrificans_NG80-2 (leading) | 1258 | 1404 | 0.9 | 1144212 | 1263072 | 0.91 | 66865 | 74180 | 0.9 | 17219 | 18086 | 0.95 |
| Geobacillus_thermodenitrificans_NG80-2 (lagging) | 403 | 326 | 1.24 | 317475 | 248130 | 1.28 | 1951 | 355 | 5.5 | 4272 | 6789 | 0.63 |
| Geobacter_metallireducens_GS-15.2 (leading) | 1226 | 1150 | 1.07 | 1266696 | 1202412 | 1.05 | 2103 | 11678 | 0.18 | 10769 | 7634 | 1.41 |
| Geobacter_metallireducens_GS-15.2 (lagging) | 537 | 605 | 0.89 | 555633 | 609138 | 0.91 | -13673 | -13613 | 1 | 12086 | 19465 | 0.62 |
| Geobacter_sulfurreducens (leading) | 1132 | 1146 | 0.99 | 1116906 | 1179388 | 0.95 | 6514 | 4062 | 1.6 | -782 | 3831 | -0.2 |
| Geobacter_sulfurreducens (lagging) | 599 | 568 | 1.05 | 586776 | 541062 | 1.08 | -13849 | -12797 | 1.08 | 13869 | 13841 | 1 |
| Geobacter_uraniumreducens_Rf4 (leading) | 1321 | 1559 | 0.85 | 1411714 | 1638206 | 0.86 | 20544 | 37186 | 0.55 | 28120 | 28768 | 0.98 |
| Geobacter_uraniumreducens_Rf4 (lagging) | 853 | 623 | 1.37 | 815205 | 600618 | 1.36 | -7903 | -4007 | 1.97 | 28072 | 23933 | 1.17 |
| Gloeobacter_violaceus (leading) | 1080 | 1143 | 0.94 | 1017144 | 1061691 | 0.96 | -8489 | -8044 | 1.06 | -3913 | -5391 | 0.73 |
| Gloeobacter_violaceus (lagging) | 1099 | 1107 | 0.99 | 1067979 | 1017150 | 1.05 | -7013 | -4989 | 1.41 | -6106 | -3229 | 1.89 |
| Gluconobacter_oxydans_621H.6 (leading) | 564 | 560 | 1.01 | 602862 | 541587 | 1.11 | -8642 | -19754 | 0.44 | -5628 | 409 | -13.76 |
| Gluconobacter_oxydans_621H.6 (lagging) | 637 | 670 | 0.95 | 622005 | 668886 | 0.93 | 8191 | 18733 | 0.44 | -10000 | -12445 | 0.8 |
| Gramella_forsetii_KT0803 (leading) | 942 | 1036 | 0.91 | 895722 | 991359 | 0.9 | 50976 | 59022 | 0.86 | 34988 | 44667 | 0.78 |
| Gramella_forsetii_KT0803 (lagging) | 848 | 757 | 1.12 | 814965 | 734859 | 1.11 | 31452 | 30021 | 1.05 | 41713 | 31664 | 1.32 |
| Granulobacter_bethesdensis_CGDNIH1 (leading) | 691 | 687 | 1.01 | 684030 | 718500 | 0.95 | 33568 | 35502 | 0.95 | -23110 | -24522 | 0.94 |
| Granulobacter_bethesdensis_CGDNIH1 (lagging) | 528 | 530 | 1 | 563139 | 517377 | 1.09 | -5357 | -4714 | 1.14 | -5106 | -4307 | 1.19 |
| Haemophilus_ducreyi_35000HP (leading) | 514 | 452 | 1.14 | 398913 | 400314 | 1 | 20720 | 14090 | 1.47 | 2011 | 9196 | 0.22 |
| Haemophilus_ducreyi_35000HP (lagging) | 399 | 351 | 1.14 | 322992 | 323379 | 1 | 306 | 10500 | 0.03 | 11474 | 6641 | 1.73 |
| Haemophilus_influenzae (leading) | 406 | 401 | 1.01 | 370301 | 387296 | 0.96 | 11019 | 11879 | 0.93 | 6695 | 5643 | 1.19 |
| Haemophilus_influenzae (lagging) | 426 | 423 | 1.01 | 400422 | 395206 | 1.01 | 14816 | 13023 | 1.14 | 4727 | 7018 | 0.67 |
| Haemophilus_influenzae_86_028NP (leading) | 460 | 442 | 1.04 | 430446 | 436095 | 0.99 | 11388 | 10884 | 1.05 | 8982 | 10677 | 0.84 |
| Haemophilus_influenzae_86_028NP (lagging) | 425 | 463 | 0.92 | 385356 | 409161 | 0.94 | 15101 | 16557 | 0.91 | 3739 | 5822 | 0.64 |
| Haemophilus_influenzae_PittEE (leading) | 430 | 473 | 0.91 | 388989 | 380493 | 1.02 | 16806 | 16709 | 1.01 | 1531 | 4124 | 0.37 |
| Haemophilus_influenzae_PittEE (lagging) | 337 | 382 | 0.88 | 328173 | 348333 | 0.94 | 7076 | 6847 | 1.03 | 9469 | 8752 | 1.08 |
| Haemophilus_influenzae_PittGG (leading) | 363 | 404 | 0.9 | 1564425 | 362049 | 4.32 | 7957 | 10276 | 0.77 | 8285 | 6757 | 1.23 |
| Haemophilus_influenzae_PittGG (lagging) | 479 | 423 | 1.13 | 382728 | 353895 | 1.08 | 16909 | 13012 | 1.3 | 4459 | 4747 | 0.94 |
| Haemophilus_somnus_129PT.2 (leading) | 565 | 480 | 1.18 | 1481416 | 486588 | 3.04 | 27687 | 26600 | 1.04 | 4791 | 8030 | 0.6 |
| Haemophilus_somnus_129PT.2 (lagging) | 351 | 395 | 0.89 | 373827 | 383652 | 0.97 | 11547 | 11256 | 1.03 | 11080 | 13216 | 0.84 |
| Hahella_chejuensis_KCTC_2396 (leading) | 1801 | 2029 | 0.89 | 1720734 | 1861032 | 0.92 | 80907 | 88656 | 0.91 | 1227 | 5986 | 0.2 |
| Hahella_chejuensis_KCTC_2396 (lagging) | 1496 | 1451 | 1.03 | 1453671 | 1338585 | 1.09 | -14264 | -17238 | 0.83 | 33273 | 34695 | 0.96 |
| Haloquadratum_walsbyi (leading) | 650 | 714 | 0.91 | 585168 | 620880 | 0.94 | 13151 | 19099 | 0.69 | 6519 | 7361 | 0.89 |
| Haloquadratum_walsbyi (lagging) | 621 | 624 | 1 | 582705 | 551793 | 1.06 | 19796 | 17775 | 1.11 | 8545 | 12464 | 0.69 |

| Chromosome | Nu of genes 1st half (Nu1) | Nu of genes 2nd half (Nu2) | Nu1/Nu2 | Total length of genes 1st half (Length1) | Total length of genes 2nd half (Length2) | Length1/ Length2 | Cumulative skew (G-C) of genes 1st half (G-C)1 | Cumulative skew (G-C) of genes 2nd half (G-C)2 | (G-C)1/ (G-C)2 | Cumulative skew (A-T) of genes 1st half (A-T)1 | Cumulative skew (A-T) of genes 2nd half (A-T)2 | (A-T)1/ (A-T)2 |
|---|---|---|---|---|---|---|---|---|---|---|---|---|
| Halorhodospira_halophila_SL1 (leading) | 483 | 742 | 0.65 | 481785 | 786921 | 0.61 | -13974 | 27119 | -0.52 | 6151 | -10466 | -0.59 |
| Halorhodospira_halophila_SL1 (lagging) | 729 | 452 | 1.61 | 724884 | 460818 | 1.57 | 16170 | -16213 | -1 | -1190 | 8099 | -0.15 |
| Helicobacter_acinonychis_Sheeba (leading) | 477 | 440 | 1.08 | 424518 | 390303 | 1.09 | 22078 | 20679 | 1.07 | 11518 | 12204 | 0.94 |
| Helicobacter_acinonychis_Sheeba (lagging) | 322 | 372 | 0.87 | 272661 | 306786 | 0.89 | 7560 | 5997 | 1.26 | 7051 | 14493 | 0.49 |
| Helicobacter_hepaticus (leading) | 427 | 397 | 1.08 | 377376 | 352599 | 1.07 | 3676 | 8161 | 0.45 | 13834 | 7176 | 1.93 |
| Helicobacter_hepaticus (lagging) | 500 | 550 | 0.91 | 464046 | 483429 | 0.96 | 26983 | 22814 | 1.18 | 8945 | 7043 | 1.27 |
| Helicobacter_pylori_26695 (leading) | 447 | 484 | 0.92 | 423406 | 451759 | 0.94 | 19494 | 21135 | 0.92 | 14581 | 16931 | 0.86 |
| Helicobacter_pylori_26695 (lagging) | 322 | 322 | 1 | 333945 | 299812 | 1.11 | 8763 | 5520 | 1.59 | 15909 | 9496 | 1.68 |
| Helicobacter_pylori_HPAG1 (leading) | 425 | 436 | 0.97 | 409167 | 396633 | 1.03 | 19167 | 17772 | 1.08 | 13452 | 12653 | 1.06 |
| Helicobacter_pylori_HPAG1 (lagging) | 337 | 337 | 1 | 333000 | 326997 | 1.02 | 8550 | 7608 | 1.12 | 14286 | 12109 | 1.18 |
| Helicobacter_pylori_J99 (leading) | 404 | 420 | 0.96 | 405249 | 410290 | 0.99 | 18637 | 18285 | 1.02 | 13674 | 15669 | 0.87 |
| Helicobacter_pylori_J99 (lagging) | 333 | 331 | 1.01 | 344952 | 325821 | 1.06 | 9999 | 7037 | 1.42 | 13367 | 12904 | 1.04 |
| Herminiimonas_arsenicoxydans (leading) | 928 | 973 | 0.95 | 891975 | 889500 | 1 | 31120 | 29353 | 1.06 | 1433 | 3693 | 0.39 |
| Herminiimonas_arsenicoxydans (lagging) | 687 | 736 | 0.93 | 608268 | 627582 | 0.97 | -2329 | -6525 | 0.36 | 12559 | 14857 | 0.85 |
| Hyperthermus_butylicus (leading) | 405 | 398 | 1.02 | 332547 | 329082 | 1.01 | 17044 | 16376 | 1.04 | 11149 | 12610 | 0.88 |
| Hyperthermus_butylicus (lagging) | 402 | 396 | 1.02 | 342174 | 350082 | 0.98 | 16965 | 17759 | 0.96 | 10249 | 16295 | 0.63 |
| Hyphomonas_neptunium_ATCC_15444 (leading) | 1016 | 920 | 1.1 | 961398 | 906498 | 1.06 | 11451 | 9452 | 1.21 | -8045 | -4378 | 1.84 |
| Hyphomonas_neptunium_ATCC_15444 (lagging) | 759 | 809 | 0.94 | 719877 | 765511 | 0.94 | -12822 | -18316 | 0.7 | 3799 | 3852 | 0.99 |
| Idiomarina_loihiensis_L2TR (leading) | 669 | 794 | 0.84 | 696051 | 789111 | 0.88 | 24418 | 39216 | 0.62 | 5771 | 4613 | 1.25 |
| Idiomarina_loihiensis_L2TR (lagging) | 645 | 519 | 1.24 | 620664 | 511557 | 1.21 | 23952 | 4142 | 5.78 | 6084 | 7505 | 0.81 |
| Jannaschia_CCS1.2 (leading) | 1221 | 1067 | 1.14 | 1119306 | 997626 | 1.12 | 39826 | 24102 | 1.65 | -18122 | -14980 | 1.21 |
| Jannaschia_CCS1.2 (lagging) | 928 | 995 | 0.93 | 839061 | 961797 | 0.87 | -3770 | 8764 | -0.43 | -5091 | -6763 | 0.75 |
| Janthinobacterium_Marseille (leading) | 1041 | 1161 | 0.9 | 1084788 | 1195644 | 0.91 | 31211 | 36298 | 0.86 | 1721 | 2140 | 0.8 |
| Janthinobacterium_Marseille (lagging) | 781 | 713 | 1.1 | 739822 | 653766 | 1.13 | -10086 | -7826 | 1.29 | 18555 | 14548 | 1.28 |
| Kineococcus_radiotolerans_SRS30216.2 (leading) | 1332 | 1262 | 1.06 | 1303923 | 1258746 | 1.04 | -40011 | -32270 | 1.24 | -10456 | -13952 | 0.75 |
| Kineococcus_radiotolerans_SRS30216.2 (lagging) | 943 | 942 | 1 | 884649 | 873069 | 1.01 | -26936 | -27563 | 0.98 | -6479 | -7716 | 0.84 |
| Klebsiella_pneumoniae_MGH_78578 (leading) | 1181 | 1316 | 0.9 | 1140747 | 1269978 | 0.9 | 25161 | 26617 | 0.95 | -4442 | -1469 | 3.02 |
| Klebsiella_pneumoniae_MGH_78578 (lagging) | 1214 | 1064 | 1.14 | 1134177 | 1029984 | 1.1 | 6580 | 8364 | 0.79 | -1181 | 2704 | -0.44 |
| Lactobacillus_acidophilus_NCFM (leading) | 742 | 629 | 1.18 | 697653 | 624471 | 1.12 | 38656 | 31565 | 1.22 | 14485 | 18008 | 0.8 |
| Lactobacillus_acidophilus_NCFM (lagging) | 214 | 276 | 0.78 | 182910 | 253908 | 0.72 | 1271 | 3522 | 0.36 | 3799 | 7918 | 0.48 |
| Lactobacillus_brevis_ATCC_367 (leading) | 613 | 806 | 0.76 | 571338 | 743151 | 0.77 | 24220 | 37701 | 0.64 | 2184 | 2338 | 0.93 |
| Lactobacillus_brevis_ATCC_367 (lagging) | 489 | 276 | 1.77 | 392823 | 224097 | 1.75 | 4454 | -7304 | -0.61 | 4727 | 1217 | 3.88 |
| Lactobacillus_casei_ATCC_334.2 (leading) | 913 | 1028 | 0.89 | 807798 | 906804 | 0.89 | 29555 | 32219 | 0.92 | 2489 | 6041 | 0.41 |
| Lactobacillus_casei_ATCC_334.2 (lagging) | 453 | 356 | 1.27 | 379671 | 291513 | 1.3 | -6367 | -5043 | 1.26 | 3794 | 1760 | 2.16 |
| Lactobacillus_delbrueckii_bulgaricus (leading) | 632 | 573 | 1.1 | 566883 | 513090 | 1.1 | 9208 | 10870 | 0.85 | 20319 | 20710 | 0.98 |
| Lactobacillus_delbrueckii_bulgaricus (lagging) | 159 | 197 | 0.81 | 140718 | 149628 | 0.94 | -5435 | -3211 | 1.69 | 4823 | 4069 | 1.19 |
| Lactobacillus_delbrueckii_bulgaricus_ATCC_BAA-365 (lead | 672 | 634 | 1.06 | 580545 | 536442 | 1.08 | 9727 | 11269 | 0.86 | 20050 | 21717 | 0.92 |
| Lactobacillus_delbrueckii_bulgaricus_ATCC_BAA-365 (lagg | 190 | 224 | 0.85 | 149508 | 166194 | 0.9 | -5255 | -3242 | 1.62 | 4499 | 5206 | 0.86 |
| Lactobacillus_gasseri_ATCC_33323 (leading) | 742 | 579 | 1.28 | 716085 | 576909 | 1.24 | 38495 | 31566 | 1.22 | 23128 | 13953 | 1.66 |
| Lactobacillus_gasseri_ATCC_33323 (lagging) | 172 | 261 | 0.66 | 144912 | 238212 | 0.61 | 366 | 1980 | 0.18 | 2434 | 5472 | 0.44 |
| Lactobacillus_johnsonii_NCC_533 (leading) | 741 | 622 | 1.19 | 722259 | 609999 | 1.18 | 36617 | 31985 | 1.14 | 22332 | 19740 | 1.13 |
| Lactobacillus_johnsonii_NCC_533 (lagging) | 193 | 264 | 0.73 | 178746 | 268218 | 0.67 | 447 | 4054 | 0.11 | 2135 | 7536 | 0.28 |
| Lactobacillus_plantarum (leading) | 1111 | 1108 | 1 | 1053420 | 1041291 | 1.01 | 49947 | 52718 | 0.95 | 4791 | 4493 | 1.07 |
| Lactobacillus_plantarum (lagging) | 407 | 380 | 1.07 | 348714 | 325707 | 1.07 | -12161 | -11040 | 1.1 | 6291 | 5509 | 1.14 |
| Lactobacillus_reuteri_F275 (leading) | 752 | 702 | 1.07 | 714858 | 623919 | 1.15 | 32520 | 25866 | 1.26 | 18906 | 15037 | 1.26 |
| Lactobacillus_reuteri_F275 (lagging) | 174 | 271 | 0.64 | 144276 | 222957 | 0.65 | -605 | -232 | 2.61 | 3697 | 1737 | 2.13 |
| Lactobacillus_sakei_23K (leading) | 746 | 703 | 1.06 | 656601 | 644619 | 1.02 | 22837 | 24096 | 0.95 | 19266 | 18051 | 1.07 |
| Lactobacillus_sakei_23K (lagging) | 202 | 227 | 0.89 | 165420 | 170958 | 0.97 | -6362 | -7048 | 0.9 | 3984 | 3740 | 1.07 |
| Lactobacillus_salivarius_UCC118.3 (leading) | 721 | 662 | 1.09 | 641322 | 630150 | 1.02 | 44294 | 41840 | 1.06 | 36288 | 29586 | 1.23 |
| Lactobacillus_salivarius_UCC118.3 (lagging) | 155 | 178 | 0.87 | 124506 | 141366 | 0.88 | 1299 | 914 | 1.42 | 1429 | 400 | 3.57 |
| Lactococcus_lactis (leading) | 966 | 862 | 1.12 | 844029 | 779604 | 1.08 | 35802 | 31214 | 1.15 | 21791 | 21646 | 1.01 |
| Lactococcus_lactis (lagging) | 235 | 257 | 0.91 | 175068 | 216696 | 0.81 | 1018 | 2415 | 0.42 | 1042 | 4063 | 0.26 |
| Lactococcus_lactis_cremoris_MG1363 (leading) | 932 | 993 | 0.94 | 814278 | 893153 | 0.91 | 34073 | 36605 | 0.93 | 21667 | 24021 | 0.9 |
| Lactococcus_lactis_cremoris_MG1363 (lagging) | 303 | 205 | 1.48 | 230664 | 147378 | 1.57 | 554 | 466 | 1.19 | 3562 | 2604 | 1.37 |
| Lactococcus_lactis_cremoris_SK11.6 (leading) | 938 | 951 | 0.99 | 804435 | 800199 | 1.01 | 33538 | 32628 | 1.03 | 19951 | 21765 | 0.92 |
| Lactococcus_lactis_cremoris_SK11.6 (lagging) | 259 | 235 | 1.1 | 198363 | 178485 | 1.11 | 106 | 69 | 1.54 | 957 | 2728 | 0.35 |
| Lawsonia_intracellularis_PHE_MN1-00 (leading) | 307 | 273 | 1.12 | 317119 | 285173 | 1.11 | 1547 | 3111 | 0.5 | 7472 | 6072 | 1.23 |
| Lawsonia_intracellularis_PHE_MN1-00 (lagging) | 282 | 317 | 0.89 | 291123 | 327537 | 0.89 | 7062 | 10054 | 0.7 | 2691 | 3751 | 0.72 |
| Legionella_pneumophila_Corby (leading) | 794 | 928 | 0.86 | 813783 | 894624 | 0.91 | 23175 | 39294 | 0.59 | 15452 | 5530 | 2.79 |
| Legionella_pneumophila_Corby (lagging) | 761 | 722 | 1.05 | 744855 | 660933 | 1.13 | 17209 | 1785 | 9.64 | 19488 | 21290 | 0.92 |
| Legionella_pneumophila_Lens.2 (leading) | 868 | 777 | 1.12 | 878388 | 796281 | 1.1 | 40769 | 37118 | 1.1 | 4574 | 5563 | 0.82 |
| Legionella_pneumophila_Lens.2 (lagging) | 602 | 630 | 0.96 | 583329 | 657390 | 0.89 | -1731 | 808 | -2.14 | 16544 | 26986 | 0.61 |
| Legionella_pneumophila_Paris.2 (leading) | 876 | 847 | 1.03 | 897897 | 860655 | 1.04 | 39993 | 39379 | 1.02 | 4401 | 6078 | 0.72 |
| Legionella_pneumophila_Paris.2 (lagging) | 664 | 639 | 1.04 | 638352 | 657699 | 0.97 | 125 | -140 | -0.89 | 19599 | 27381 | 0.72 |
| Legionella_pneumophila_Philadelphia_1 (leading) | 842 | 823 | 1.02 | 870468 | 858138 | 1.01 | 39801 | 38737 | 1.03 | 2880 | 8211 | 0.35 |
| Legionella_pneumophila_Philadelphia_1 (lagging) | 665 | 611 | 1.09 | 641793 | 642960 | 1 | -1355 | -20 | 67.75 | 19712 | 25710 | 0.77 |
| Leifsonia_xyli_xyli_CTCB0 (leading) | 615 | 624 | 0.99 | 607197 | 549537 | 1.1 | -5185 | -3989 | 1.3 | -3110 | -3538 | 0.88 |

| Chromosome | Nu of genes 1st half (Nu1) | Nu of genes 2nd half (Nu2) | Nu1/Nu2 | Total length of genes 1st half (Length1) | Total length of genes 2nd half (Length2) | Length1/ Length2 | Cumulative skew (G-C) of genes 1st half (G-C)1 | Cumulative skew (G-C) of genes 2nd half (G-C)2 | (G-C)1/ (G-C)2 | Cumulative skew (A-T) of genes 1st half (A-T)1 | Cumulative skew (A-T) of genes 2nd half (A-T)2 | (A-T)1/ (A-T)2 |
|---|---|---|---|---|---|---|---|---|---|---|---|---|
| Leifsonia_xyli_xyli_CTCB0 (lagging) | 361 | 429 | 0.84 | 303642 | 352533 | 0.86 | -9539 | -9233 | 1.03 | 1907 | 2094 | 0.91 |
| Leptospira_borgpetersenii_serovar_Hardjo-bovis_JB197 (l | 753 | 732 | 1.03 | 749712 | 739500 | 1.01 | 25836 | 26850 | 0.96 | 14094 | 14598 | 0.97 |
| Leptospira_borgpetersenii_serovar_Hardjo-bovis_JB197 (l | 580 | 579 | 1 | 552936 | 563910 | 0.98 | 2617 | -1069 | -2.45 | 22691 | 21813 | 1.04 |
| Leptospira_borgpetersenii_serovar_Hardjo-bovis_L550 (le | 791 | 742 | 1.07 | 774162 | 740103 | 1.05 | 29171 | 24405 | 1.2 | 16137 | 13250 | 1.22 |
| Leptospira_borgpetersenii_serovar_Hardjo-bovis_L550 (la | 577 | 592 | 0.97 | 558915 | 582945 | 0.96 | 1002 | 844 | 1.19 | 21149 | 23305 | 0.91 |
| Leptospira_interrogans_serovar_Copenhageni (leading) | 948 | 1002 | 0.95 | 914358 | 966420 | 0.95 | 37996 | 43218 | 0.88 | 15690 | 8964 | 1.75 |
| Leptospira_interrogans_serovar_Copenhageni (lagging) | 754 | 689 | 1.09 | 686568 | 635091 | 1.08 | -311 | -140 | 2.22 | 28115 | 23703 | 1.19 |
| Leptospira_interrogans_serovar_Lai (leading) | 1283 | 1191 | 1.08 | 1053891 | 931992 | 1.13 | 45354 | 38698 | 1.17 | 12125 | 12890 | 0.94 |
| Leptospira_interrogans_serovar_Lai (lagging) | 904 | 981 | 0.92 | 656970 | 745830 | 0.88 | -854 | 862 | -0.99 | 24722 | 29576 | 0.84 |
| Leuconostoc_mesenteroides_ATCC_8293.2 (leading) | 794 | 754 | 1.05 | 766926 | 692679 | 1.11 | 36859 | 34289 | 1.07 | 10491 | 18254 | 0.57 |
| Leuconostoc_mesenteroides_ATCC_8293.2 (lagging) | 151 | 270 | 0.56 | 133593 | 209691 | 0.64 | -1211 | 2539 | -0.48 | 1846 | 6490 | 0.28 |
| Listeria_innocua (leading) | 1147 | 1196 | 0.96 | 1050993 | 1091841 | 0.96 | 46138 | 52707 | 0.88 | 64717 | 60142 | 1.08 |
| Listeria_innocua (lagging) | 345 | 279 | 1.24 | 293856 | 240795 | 1.22 | 4156 | 3023 | 1.37 | 10230 | 5856 | 1.75 |
| Listeria_monocytogenes (leading) | 1095 | 1131 | 0.97 | 1024713 | 1054782 | 0.97 | 45925 | 52669 | 0.87 | 59050 | 53789 | 1.1 |
| Listeria_monocytogenes (lagging) | 335 | 284 | 1.18 | 286725 | 254481 | 1.13 | 3376 | 2820 | 1.2 | 9247 | 5939 | 1.56 |
| Listeria_monocytogenes_4b_F2365 (leading) | 1119 | 1094 | 1.02 | 1017228 | 1026598 | 0.99 | 45106 | 50915 | 0.89 | 59158 | 51086 | 1.16 |
| Listeria_monocytogenes_4b_F2365 (lagging) | 323 | 284 | 1.14 | 270879 | 254421 | 1.06 | 2853 | 2575 | 1.11 | 7736 | 5522 | 1.4 |
| Listeria_welshimeri_serovar_6b_SLCC5334 (leading) | 1101 | 1089 | 1.01 | 1000911 | 1008165 | 0.99 | 49918 | 51163 | 0.98 | 57667 | 48718 | 1.18 |
| Listeria_welshimeri_serovar_6b_SLCC5334 (lagging) | 307 | 276 | 1.11 | 239901 | 241020 | 1 | 4295 | 3836 | 1.12 | 6760 | 4596 | 1.47 |
| Magnetococcus_MC-1 (leading) | 1104 | 1127 | 0.98 | 1165653 | 1314831 | 0.89 | 66663 | 81685 | 0.82 | -18330 | -22330 | 0.82 |
| Magnetococcus_MC-1 (lagging) | 772 | 712 | 1.08 | 875637 | 725970 | 1.21 | 2464 | -9912 | -0.25 | -3723 | 2006 | -1.86 |
| Magnetospirillum_magneticum_AMB-1 (leading) | 1303 | 1112 | 1.17 | 1267179 | 1042899 | 1.22 | -1238 | 349 | -3.55 | -5782 | -7544 | 0.77 |
| Magnetospirillum_magneticum_AMB-1 (lagging) | 979 | 1164 | 0.84 | 900528 | 1177641 | 0.76 | -22927 | -32539 | 0.7 | 1437 | 6507 | 0.22 |
| Mannheimia_succiniciproducens_MBEL55E (leading) | 565 | 744 | 0.76 | 509139 | 654681 | 0.78 | 23476 | 28993 | 0.81 | 3237 | 3886 | 0.83 |
| Mannheimia_succiniciproducens_MBEL55E (lagging) | 602 | 468 | 1.29 | 536016 | 379494 | 1.41 | 8078 | 4283 | 1.89 | 10272 | 9771 | 1.05 |
| Maricaulis_maris_MCS10 (leading) | 882 | 891 | 0.99 | 885732 | 891666 | 0.99 | 7952 | 11247 | 0.71 | -16956 | -16477 | 1.03 |
| Maricaulis_maris_MCS10 (lagging) | 646 | 643 | 1 | 625254 | 626388 | 1 | -14265 | -11531 | 1.24 | -3513 | -2113 | 1.66 |
| Marinobacter_aquaeolei_VT8.3 (leading) | 1031 | 1110 | 0.93 | 1064742 | 1129527 | 0.94 | 27641 | 43118 | 0.64 | 3191 | -7039 | -0.45 |
| Marinobacter_aquaeolei_VT8.3 (lagging) | 880 | 836 | 1.05 | 877767 | 831732 | 1.06 | 7734 | -12747 | -0.61 | 8133 | 17547 | 0.46 |
| Marinomonas_MWYL1 (leading) | 1312 | 1361 | 0.96 | 1297395 | 1398321 | 0.93 | 74332 | 82616 | 0.9 | -33551 | -35527 | 0.94 |
| Marinomonas_MWYL1 (lagging) | 936 | 829 | 1.13 | 963744 | 818625 | 1.18 | -7798 | -9116 | 0.86 | 26298 | 27913 | 0.94 |
| Mesoplasma_florum_L1 (leading) | 294 | 308 | 0.95 | 327135 | 337212 | 0.97 | 14445 | 14935 | 0.97 | 30152 | 29909 | 1.01 |
| Mesoplasma_florum_L1 (lagging) | 46 | 33 | 1.39 | 44034 | 28356 | 1.55 | 1891 | 1272 | 1.49 | 3359 | 1600 | 2.1 |
| Mesorhizobium_BNC1.4 (leading) | 1006 | 998 | 1.01 | 993162 | 992262 | 1 | 10595 | 12473 | 0.85 | -4291 | -8199 | 0.52 |
| Mesorhizobium_BNC1.4 (lagging) | 1037 | 1022 | 1.01 | 986784 | 974472 | 1.01 | 4823 | 480 | 10.05 | 991 | -6214 | -0.16 |
| Mesorhizobium_loti (leading) | 1615 | 1571 | 1.03 | 1435974 | 1403175 | 1.02 | -29589 | -11123 | 2.66 | 7561 | 2046 | 3.7 |
| Mesorhizobium_loti (lagging) | 1797 | 1759 | 1.02 | 1642323 | 1635489 | 1 | 5346 | -9535 | -0.56 | 1015 | -4576 | -0.22 |
| Metallosphaera_sedula_DSM_5348 (leading) | 632 | 525 | 1.2 | 549657 | 440448 | 1.25 | 33068 | 23792 | 1.39 | 16389 | 15416 | 1.06 |
| Metallosphaera_sedula_DSM_5348 (lagging) | 502 | 596 | 0.84 | 421929 | 514479 | 0.82 | 24358 | 29464 | 0.83 | 14023 | 21865 | 0.64 |
| Methylibium_petroleiphilum_PM1 (leading) | 1080 | 1052 | 1.03 | 1070595 | 1020378 | 1.05 | 5970 | 8214 | 0.73 | 541 | 3836 | 0.14 |
| Methylibium_petroleiphilum_PM1 (lagging) | 804 | 882 | 0.91 | 795849 | 846936 | 0.94 | -9354 | -10890 | 0.86 | 4435 | 7096 | 0.63 |
| Methylobacillus_flagellatus_KT (leading) | 725 | 845 | 0.86 | 704910 | 821307 | 0.86 | 25142 | 28745 | 0.87 | -1920 | -1782 | 1.08 |
| Methylobacillus_flagellatus_KT (lagging) | 659 | 523 | 1.26 | 626280 | 528051 | 1.19 | -15669 | -14800 | 1.06 | 17649 | 14333 | 1.23 |
| Methylococcus_capsulatus_Bath (leading) | 815 | 808 | 1.01 | 787197 | 776730 | 1.01 | 16743 | 16568 | 1.01 | -6432 | -3550 | 1.81 |
| Methylococcus_capsulatus_Bath (lagging) | 647 | 685 | 0.94 | 675631 | 715122 | 0.94 | -15824 | -20923 | 0.76 | 7884 | 13487 | 0.58 |
| Moorella_thermoacetica_ATCC_39073 (leading) | 975 | 1019 | 0.96 | 904710 | 942873 | 0.96 | 22889 | 20467 | 1.12 | 9974 | 5748 | 1.74 |
| Moorella_thermoacetica_ATCC_39073 (lagging) | 270 | 200 | 1.35 | 242082 | 184113 | 1.31 | -2129 | -1895 | 1.12 | 2687 | 1314 | 2.04 |
| Mycobacterium_avium_104 (leading) | 1430 | 1494 | 0.96 | 1371447 | 1502880 | 0.91 | 4321 | 3449 | 1.25 | 6980 | 5119 | 1.36 |
| Mycobacterium_avium_104 (lagging) | 1162 | 1033 | 1.12 | 1031097 | 947688 | 1.09 | -7331 | -4297 | 1.71 | 7930 | 645 | 12.29 |
| Mycobacterium_avium_paratuberculosis (leading) | 1157 | 1105 | 1.05 | 1188261 | 1141272 | 1.04 | 1700 | 3613 | 0.47 | 4609 | 3275 | 1.41 |
| Mycobacterium_avium_paratuberculosis (lagging) | 1017 | 1070 | 0.95 | 1017858 | 1073691 | 0.95 | -2982 | -4875 | 0.61 | 1870 | 4176 | 0.45 |
| Mycobacterium_bovis (leading) | 1153 | 1162 | 0.99 | 1140786 | 1166334 | 0.98 | 28984 | 32940 | 0.88 | -12656 | -12446 | 1.02 |
| Mycobacterium_bovis (lagging) | 790 | 814 | 0.97 | 836346 | 791397 | 1.06 | -1666 | -3228 | 0.52 | 2534 | 1707 | 1.48 |
| Mycobacterium_bovis_BCG_Pasteur_1173P2 (leading) | 1159 | 1172 | 0.99 | 1151949 | 1179522 | 0.98 | 28747 | 34266 | 0.84 | -12496 | -12512 | 1 |
| Mycobacterium_bovis_BCG_Pasteur_1173P2 (lagging) | 797 | 823 | 0.97 | 841023 | 799584 | 1.05 | -1506 | -2971 | 0.51 | 2375 | 1881 | 1.26 |
| Mycobacterium_gilvum_PYR-GCK (leading) | 1337 | 1441 | 0.93 | 1361622 | 1449366 | 0.94 | 1454 | 2692 | 0.54 | 3818 | 4652 | 0.82 |
| Mycobacterium_gilvum_PYR-GCK (lagging) | 1327 | 1135 | 1.17 | 1245084 | 1143357 | 1.09 | -10325 | -9094 | 1.14 | 3449 | 1019 | 3.38 |
| Mycobacterium_JLS (leading) | 1652 | 1634 | 1.01 | 1606116 | 1624896 | 0.99 | -439 | 1506 | -0.29 | 5613 | 5410 | 1.04 |
| Mycobacterium_JLS (lagging) | 1209 | 1243 | 0.97 | 1204023 | 1185963 | 1.02 | -9906 | -13088 | 0.76 | 3295 | 4065 | 0.81 |
| Mycobacterium_KMS.3 (leading) | 1605 | 1556 | 1.03 | 1564884 | 1550631 | 1.01 | -516 | 114 | -4.53 | 5954 | 5903 | 1.01 |
| Mycobacterium_KMS.3 (lagging) | 1113 | 1185 | 0.94 | 1101480 | 1124127 | 0.98 | -11282 | -12556 | 0.9 | 4104 | 4329 | 0.95 |
| Mycobacterium_leprae (leading) | 551 | 484 | 1.14 | 579333 | 482925 | 1.2 | 25315 | 18413 | 1.37 | -15624 | -10390 | 1.5 |
| Mycobacterium_leprae (lagging) | 301 | 268 | 1.12 | 286563 | 272169 | 1.05 | -4438 | -2595 | 1.71 | 4469 | 3924 | 1.14 |
| Mycobacterium_MCS (leading) | 1586 | 1556 | 1.02 | 1554381 | 1558155 | 1 | -366 | 569 | -0.64 | 5941 | 5874 | 1.01 |
| Mycobacterium_MCS (lagging) | 1100 | 1148 | 0.96 | 1096572 | 1104063 | 0.99 | -11678 | -12251 | 0.95 | 4306 | 3582 | 1.2 |
| Mycobacterium_smegmatis_MC2_155 (leading) | 1930 | 1890 | 1.02 | 1880043 | 1792284 | 1.05 | 2481 | 907 | 2.74 | 7274 | 5979 | 1.22 |
| Mycobacterium_smegmatis_MC2_155 (lagging) | 1372 | 1523 | 0.9 | 1291293 | 1410000 | 0.92 | -17175 | -22623 | 0.76 | 3480 | 4585 | 0.76 |

| Chromosome | Nu of genes 1st half (Nu1) | Nu of genes 2nd half (Nu2) | Nu1/Nu2 | Total length of genes 1st half (Length1) | Total length of genes 2nd half (Length2) | Length1/ Length2 | Cumulative skew (G-C) of genes 1st half (G-C)1 | Cumulative skew (G-C) of genes 2nd half (G-C)2 | (G-C)1/ (G-C)2 | Cumulative skew (A-T) of genes 1st half (A-T)1 | Cumulative skew (A-T) of genes 2nd half (A-T)2 | (A-T)1/ (A-T)2 |
|---|---|---|---|---|---|---|---|---|---|---|---|---|
| Mycobacterium_tuberculosis_CDC1551 (leading) | 1204 | 1253 | 0.96 | 1150365 | 1189020 | 0.97 | 28296 | 34917 | 0.81 | -12821 | -12217 | 1.05 |
| Mycobacterium_tuberculosis_CDC1551 (lagging) | 857 | 874 | 0.98 | 845307 | 808410 | 1.05 | -3328 | -4333 | 0.77 | 2212 | 1830 | 1.21 |
| Mycobacterium_tuberculosis_F11 (leading) | 1166 | 1162 | 1 | 1164036 | 1201548 | 0.97 | 29107 | 33054 | 0.88 | -12529 | -12348 | 1.01 |
| Mycobacterium_tuberculosis_F11 (lagging) | 813 | 799 | 1.02 | 862125 | 802611 | 1.07 | -2259 | -3214 | 0.7 | 2676 | 2307 | 1.16 |
| Mycobacterium_tuberculosis_H37Ra (leading) | 1178 | 1189 | 0.99 | 1163019 | 1204227 | 0.97 | 29139 | 33307 | 0.87 | -12666 | -12886 | 0.98 |
| Mycobacterium_tuberculosis_H37Ra (lagging) | 823 | 843 | 0.98 | 859425 | 807051 | 1.06 | -2627 | -4113 | 0.64 | 2750 | 1890 | 1.46 |
| Mycobacterium_tuberculosis_H37Rv (leading) | 1177 | 1163 | 1.01 | 1159728 | 1194306 | 0.97 | 29075 | 32911 | 0.88 | -12417 | -12609 | 0.98 |
| Mycobacterium_tuberculosis_H37Rv (lagging) | 817 | 831 | 0.98 | 856386 | 809967 | 1.06 | -2339 | -3344 | 0.7 | 2707 | 2353 | 1.15 |
| Mycobacterium_ulcerans_Agy99 (leading) | 1165 | 1165 | 1 | 1146075 | 1136559 | 1.01 | 16948 | 18325 | 0.92 | -3595 | -5750 | 0.63 |
| Mycobacterium_ulcerans_Agy99 (lagging) | 918 | 911 | 1.01 | 919020 | 872835 | 1.05 | -3406 | -8679 | 0.39 | -838 | 4526 | -0.19 |
| Mycobacterium_vanbaalenii_PYR-1 (leading) | 1798 | 1666 | 1.08 | 1769022 | 1715211 | 1.03 | 14465 | 14043 | 1.03 | 3009 | 1488 | 2.02 |
| Mycobacterium_vanbaalenii_PYR-1 (lagging) | 1230 | 1284 | 0.96 | 1213713 | 1243179 | 0.98 | -10251 | -13473 | 0.76 | 3006 | 5360 | 0.56 |
| Mycoplasma_agalactiae_PG2 (leading) | 210 | 228 | 0.92 | 221388 | 239649 | 0.92 | 5868 | 9735 | 0.6 | 16624 | 18800 | 0.88 |
| Mycoplasma_agalactiae_PG2 (lagging) | 158 | 145 | 1.09 | 169713 | 136023 | 1.25 | 4638 | 2744 | 1.69 | 12701 | 8787 | 1.45 |
| Mycoplasma_capricolum_ATCC_27343 (leading) | 287 | 295 | 0.97 | 315282 | 308673 | 1.02 | 12009 | 11798 | 1.02 | 22445 | 21305 | 1.05 |
| Mycoplasma_capricolum_ATCC_27343 (lagging) | 105 | 124 | 0.85 | 130473 | 137406 | 0.95 | 3562 | 4839 | 0.74 | 10443 | 11391 | 0.92 |
| Mycoplasma_gallisepticum (leading) | 292 | 279 | 1.05 | 370272 | 340722 | 1.09 | 4621 | 5821 | 0.79 | 22543 | 20517 | 1.1 |
| Mycoplasma_gallisepticum (lagging) | 62 | 92 | 0.67 | 62856 | 100896 | 0.62 | 973 | 1302 | 0.75 | 3409 | 5618 | 0.61 |
| Mycoplasma_genitalium (leading) | 214 | 168 | 1.27 | 235173 | 206949 | 1.14 | 4498 | 4534 | 0.99 | 9323 | 7457 | 1.25 |
| Mycoplasma_genitalium (lagging) | 31 | 63 | 0.49 | 27657 | 58029 | 0.48 | 541 | 1103 | 0.49 | 1170 | 3234 | 0.36 |
| Mycoplasma_hyopneumoniae_232 (leading) | 196 | 179 | 1.09 | 201528 | 243210 | 0.83 | 2871 | 1419 | 2.02 | 12651 | 14765 | 0.86 |
| Mycoplasma_hyopneumoniae_232 (lagging) | 174 | 141 | 1.23 | 198018 | 163914 | 1.21 | 2839 | 1542 | 1.84 | 10171 | 8886 | 1.14 |
| Mycoplasma_hyopneumoniae_7448 (leading) | 201 | 180 | 1.12 | 231834 | 249009 | 0.93 | 3117 | 2113 | 1.48 | 11587 | 15386 | 0.75 |
| Mycoplasma_hyopneumoniae_7448 (lagging) | 159 | 122 | 1.3 | 166800 | 143679 | 1.16 | 2510 | 1205 | 2.08 | 10752 | 7352 | 1.46 |
| Mycoplasma_hyopneumoniae_J (leading) | 197 | 180 | 1.09 | 223632 | 244965 | 0.91 | 2918 | 1684 | 1.73 | 11672 | 15517 | 0.75 |
| Mycoplasma_hyopneumoniae_J (lagging) | 162 | 125 | 1.3 | 169890 | 148518 | 1.14 | 2518 | 1606 | 1.57 | 10710 | 8108 | 1.32 |
| Mycoplasma_mobile_163K (leading) | 194 | 188 | 1.03 | 231003 | 215595 | 1.07 | 6462 | 6350 | 1.02 | 13843 | 14419 | 0.96 |
| Mycoplasma_mobile_163K (lagging) | 122 | 128 | 0.95 | 133323 | 125820 | 1.06 | 5572 | 5191 | 1.07 | 9767 | 9375 | 1.04 |
| Mycoplasma_mycoides (leading) | 301 | 342 | 0.88 | 307065 | 331368 | 0.93 | 11298 | 11578 | 0.98 | 22189 | 26104 | 0.85 |
| Mycoplasma_mycoides (lagging) | 204 | 168 | 1.21 | 192921 | 160251 | 1.2 | 6251 | 5168 | 1.21 | 16112 | 11449 | 1.41 |
| Mycoplasma_penetrans (leading) | 424 | 421 | 1.01 | 511173 | 522735 | 0.98 | 17200 | 15912 | 1.08 | 32495 | 31041 | 1.05 |
| Mycoplasma_penetrans (lagging) | 111 | 80 | 1.39 | 97365 | 74094 | 1.31 | 2681 | 1821 | 1.47 | 9226 | 6235 | 1.48 |
| Mycoplasma_pneumoniae (leading) | 298 | 240 | 1.24 | 322050 | 266328 | 1.21 | 2836 | 2370 | 1.2 | 12792 | 8764 | 1.46 |
| Mycoplasma_pneumoniae (lagging) | 43 | 107 | 0.4 | 35400 | 96714 | 0.37 | 329 | 340 | 0.97 | 855 | 5650 | 0.15 |
| Mycoplasma_pulmonis (leading) | 253 | 223 | 1.13 | 278418 | 246054 | 1.13 | 5251 | 4851 | 1.08 | 21759 | 19481 | 1.12 |
| Mycoplasma_pulmonis (lagging) | 152 | 153 | 0.99 | 158400 | 188694 | 0.84 | 2469 | 2435 | 1.01 | 12337 | 15575 | 0.79 |
| Mycoplasma_synoviae_53 (leading) | 137 | 203 | 0.67 | 147963 | 212478 | 0.7 | 1649 | 2977 | 0.55 | 10974 | 15845 | 0.69 |
| Mycoplasma_synoviae_53 (lagging) | 202 | 129 | 1.57 | 208734 | 144066 | 1.45 | 678 | 2185 | 0.31 | 17676 | 11729 | 1.51 |
| Myxococcus_xanthus_DK_1622 (leading) | 1921 | 1959 | 0.98 | 2242380 | 2309101 | 0.97 | 78599 | 88094 | 0.89 | -13981 | -23182 | 0.6 |
| Myxococcus_xanthus_DK_1622 (lagging) | 1779 | 1671 | 1.06 | 1945506 | 1863483 | 1.04 | -17623 | -18907 | 0.93 | 15025 | 13508 | 1.11 |
| Natronomonas_pharaonis (leading) | 704 | 646 | 1.09 | 624027 | 562905 | 1.11 | -2114 | -1238 | 1.71 | 12957 | 14511 | 0.89 |
| Natronomonas_pharaonis (lagging) | 621 | 689 | 0.9 | 557145 | 606936 | 0.92 | 2764 | 4811 | 0.57 | 16377 | 16437 | 1 |
| Neisseria_gonorrhoeae_FA_1090 (leading) | 505 | 544 | 0.93 | 420573 | 452691 | 0.93 | 2196 | 2259 | 0.97 | 14731 | 11116 | 1.33 |
| Neisseria_gonorrhoeae_FA_1090 (lagging) | 518 | 434 | 1.19 | 438510 | 380496 | 1.15 | -10598 | -6478 | 1.64 | 19272 | 13352 | 1.44 |
| Neisseria_meningitidis_FAM18 (leading) | 541 | 488 | 1.11 | 471496 | 460399 | 1.02 | 10754 | 7822 | 1.37 | 10535 | 8656 | 1.22 |
| Neisseria_meningitidis_FAM18 (lagging) | 467 | 420 | 1.11 | 425101 | 402783 | 1.06 | -18944 | -13968 | 1.36 | 20927 | 20186 | 1.04 |
| Neisseria_meningitidis_MC58 (leading) | 621 | 508 | 1.22 | 507975 | 456348 | 1.11 | 10372 | 4943 | 2.1 | 10679 | 13447 | 0.79 |
| Neisseria_meningitidis_MC58 (lagging) | 456 | 477 | 0.96 | 398763 | 433086 | 0.92 | -15913 | -14421 | 1.1 | 20422 | 20583 | 0.99 |
| Neisseria_meningitidis_Z2491 (leading) | 465 | 548 | 0.85 | 396180 | 475347 | 0.83 | 2693 | 4710 | 0.57 | 10633 | 12397 | 0.86 |
| Neisseria_meningitidis_Z2491 (lagging) | 576 | 459 | 1.25 | 484458 | 399355 | 1.21 | -12301 | -9202 | 1.34 | 18763 | 19123 | 0.98 |
| Neorickettsia_sennetsu_Miyayama (leading) | 291 | 257 | 1.13 | 212100 | 214164 | 0.99 | 16389 | 16850 | 0.97 | -6785 | -5316 | 1.28 |
| Neorickettsia_sennetsu_Miyayama (lagging) | 198 | 185 | 1.07 | 163383 | 163180 | 1 | 3940 | 2889 | 1.36 | 8907 | 8290 | 1.07 |
| Nitratiruptor_SB155-2 (leading) | 584 | 610 | 0.96 | 552615 | 589965 | 0.94 | 29076 | 31648 | 0.92 | 32747 | 31575 | 1.04 |
| Nitratiruptor_SB155-2 (lagging) | 336 | 312 | 1.08 | 324993 | 298710 | 1.09 | 784 | 1248 | 0.63 | 20913 | 19028 | 1.1 |
| Nitrobacter_hamburgensis_X14.4 (leading) | 1044 | 1082 | 0.96 | 1007811 | 998730 | 1.01 | 11040 | 14003 | 0.79 | 385 | -2295 | -0.17 |
| Nitrobacter_hamburgensis_X14.4 (lagging) | 839 | 838 | 1 | 787767 | 751479 | 1.05 | -9309 | -5870 | 1.59 | 10390 | 8371 | 1.24 |
| Nitrobacter_winogradskyi_Nb-255 (leading) | 840 | 896 | 0.94 | 786690 | 822318 | 0.96 | 11344 | 15042 | 0.75 | -2054 | -4272 | 0.48 |
| Nitrobacter_winogradskyi_Nb-255 (lagging) | 707 | 678 | 1.04 | 652476 | 601383 | 1.08 | -5215 | -6924 | 0.75 | 6349 | 7743 | 0.82 |
| Nitrosococcus_oceani_ATCC_19707.2 (leading) | 901 | 932 | 0.97 | 883386 | 965073 | 0.92 | 41004 | 47464 | 0.86 | -15554 | -22645 | 0.69 |
| Nitrosococcus_oceani_ATCC_19707.2 (lagging) | 588 | 552 | 1.07 | 574404 | 528291 | 1.09 | -9607 | -7117 | 1.35 | 3965 | 6010 | 0.66 |
| Nitrosomonas_europaea (leading) | 640 | 718 | 0.89 | 617931 | 706131 | 0.88 | 19974 | 34026 | 0.59 | 663 | -4793 | -0.14 |
| Nitrosomonas_europaea (lagging) | 594 | 508 | 1.17 | 587133 | 494430 | 1.19 | 3464 | -4892 | -0.71 | 9655 | 12030 | 0.8 |
| Nitrosomonas_eutropha_C71.3 (leading) | 653 | 653 | 1 | 601722 | 586428 | 1.03 | 31336 | 28017 | 1.12 | -3998 | -2269 | 1.76 |
| Nitrosomonas_eutropha_C71.3 (lagging) | 574 | 563 | 1.02 | 544107 | 545889 | 1 | 2999 | 2748 | 1.09 | 7134 | 8751 | 0.82 |
| Nitrosospira_multiformis_ATCC_25196 (leading) | 795 | 823 | 0.97 | 780351 | 854211 | 0.91 | 26349 | 28604 | 0.92 | 6426 | 5861 | 1.1 |
| Nitrosospira_multiformis_ATCC_25196 (lagging) | 596 | 542 | 1.1 | 579915 | 507150 | 1.14 | 6619 | 7197 | 0.92 | 6818 | 7849 | 0.87 |
| Nocardia_farcinica_IFM10152 (leading) | 1632 | 1613 | 1.01 | 1612863 | 1604316 | 1.01 | -11904 | -14005 | 0.85 | -129 | 1317 | -0.1 |

| Chromosome | Nu of genes 1st half (Nu1) | Nu of genes 2nd half (Nu2) | Nu1/Nu2 | Total length of genes 1st half (Length1) | Total length of genes 2nd half (Length2) | Length1/ Length2 | Cumulative skew (G-C) of genes 1st half (G-C)1 | Cumulative skew (G-C) of genes 2nd half (G-C)2 | (G-C)1/ (G-C)2 | Cumulative skew (A-T) of genes 1st half (A-T)1 | Cumulative skew (A-T) of genes 2nd half (A-T)2 | (A-T)1/ (A-T)2 |
|---|---|---|---|---|---|---|---|---|---|---|---|---|
| Nocardia_farcinica_IFM10152 (lagging) | 1194 | 1243 | 0.96 | 1104540 | 1123938 | 0.98 | -23499 | -22965 | 1.02 | 1773 | 3659 | 0.48 |
| Nocardioides_JS614.2 (leading) | 1390 | 1302 | 1.07 | 1381866 | 1272903 | 1.09 | -14868 | -11640 | 1.28 | -1574 | -761 | 2.07 |
| Nocardioides_JS614.2 (lagging) | 901 | 1051 | 0.86 | 863436 | 1032204 | 0.84 | -21362 | -17051 | 1.25 | 2390 | -583 | -4.1 |
| Nostoc_sp.5 (leading) | 1298 | 1316 | 0.99 | 1275627 | 1293753 | 0.99 | 21790 | 19578 | 1.11 | 22481 | 22649 | 0.99 |
| Nostoc_sp.5 (lagging) | 1349 | 1402 | 0.96 | 1377168 | 1345818 | 1.02 | 18350 | 20801 | 0.88 | 25782 | 20799 | 1.24 |
| Novosphingobium_aromaticivorans_DSM_12444 (leading) | 934 | 895 | 1.04 | 905349 | 907803 | 1 | 995 | 3563 | 0.28 | -1682 | -2318 | 0.73 |
| Novosphingobium_aromaticivorans_DSM_12444 (lagging) | 751 | 743 | 1.01 | 728121 | 721989 | 1.01 | -14156 | -11675 | 1.21 | 4599 | 7572 | 0.61 |
| Oceanobacillus_iheyensis (leading) | 1316 | 1293 | 1.02 | 1143108 | 1210809 | 0.94 | 72516 | 75787 | 0.96 | 59376 | 62148 | 0.96 |
| Oceanobacillus_iheyensis (lagging) | 463 | 427 | 1.08 | 364371 | 346107 | 1.05 | 9210 | 8685 | 1.06 | 15411 | 14204 | 1.08 |
| Ochrobactrum_anthropi_ATCC_49188 (leading) | 765 | 660 | 1.16 | 724632 | 615416 | 1.18 | 13977 | 1573 | 8.89 | -7817 | -2115 | 3.7 |
| Ochrobactrum_anthropi_ATCC_49188 (lagging) | 587 | 718 | 0.82 | 516353 | 637327 | 0.81 | -3211 | 11481 | -0.28 | -3 | -6762 | 0 |
| Oenococcus_oeni_PSU-1 (leading) | 620 | 572 | 1.08 | 539004 | 532446 | 1.01 | 19967 | 19256 | 1.04 | -2567 | -4318 | 0.59 |
| Oenococcus_oeni_PSU-1 (lagging) | 236 | 262 | 0.9 | 170193 | 227046 | 0.75 | 1685 | 3594 | 0.47 | 4830 | 7750 | 0.62 |
| Onion_yellows_phytoplasma (leading) | 309 | 208 | 1.49 | 253776 | 170244 | 1.49 | -1788 | -3045 | 0.59 | 12746 | 13436 | 0.95 |
| Onion_yellows_phytoplasma (lagging) | 76 | 160 | 0.48 | 64629 | 138138 | 0.47 | -2469 | -3344 | 0.74 | 6754 | 12512 | 0.54 |
| Orientia_tsutsugamushi_Boryong (leading) | 236 | 271 | 0.87 | 219069 | 225897 | 0.97 | 6647 | 7007 | 0.95 | 13738 | 12880 | 1.07 |
| Orientia_tsutsugamushi_Boryong (lagging) | 368 | 306 | 1.2 | 319632 | 273360 | 1.17 | 13993 | 11673 | 1.2 | 14659 | 11573 | 1.27 |
| Parabacteroides_distasonis_ATCC_8503 (leading) | 1083 | 885 | 1.22 | 1174752 | 1031919 | 1.14 | 55225 | 50184 | 1.1 | 16023 | 14281 | 1.12 |
| Parabacteroides_distasonis_ATCC_8503 (lagging) | 894 | 987 | 0.91 | 1005183 | 1130316 | 0.89 | 49386 | 52877 | 0.93 | 4175 | 18947 | 0.22 |
| Parachlamydia_sp_UWE25 (leading) | 515 | 490 | 1.05 | 481605 | 490074 | 0.98 | 15543 | 12596 | 1.23 | 3390 | 7196 | 0.47 |
| Parachlamydia_sp_UWE25 (lagging) | 496 | 529 | 0.94 | 526557 | 489804 | 1.08 | -1810 | -2226 | 0.81 | 9459 | 10038 | 0.94 |
| Paracoccus_denitrificans_PD1222.2 (leading) | 490 | 419 | 1.17 | 428157 | 431925 | 0.99 | 6391 | 3356 | 1.9 | -5454 | -3943 | 1.38 |
| Paracoccus_denitrificans_PD1222.2 (lagging) | 377 | 375 | 1.01 | 341574 | 365094 | 0.94 | 2262 | 2158 | 1.05 | -1612 | -366 | 4.4 |
| Paracoccus_denitrificans_PD1222 (leading) | 837 | 731 | 1.15 | 751878 | 701715 | 1.07 | 11489 | 10835 | 1.06 | -4717 | -6310 | 0.75 |
| Paracoccus_denitrificans_PD1222 (lagging) | 607 | 623 | 0.97 | 538734 | 587631 | 0.92 | 75 | -1905 | -0.04 | -1005 | 1728 | -0.58 |
| Parvibaculum_lavamentivorans_DS-1 (leading) | 981 | 801 | 1.22 | 987879 | 754035 | 1.31 | -2867 | -12624 | 0.23 | -3330 | 7477 | -0.45 |
| Parvibaculum_lavamentivorans_DS-1 (lagging) | 815 | 1038 | 0.79 | 777027 | 1010067 | 0.77 | 1470 | 15359 | 0.1 | 8371 | -1820 | -4.6 |
| Pasteurella_multocida (leading) | 490 | 499 | 0.98 | 533475 | 485568 | 1.1 | 19953 | 14991 | 1.33 | 5896 | 5469 | 1.08 |
| Pasteurella_multocida (lagging) | 465 | 560 | 0.83 | 468282 | 521106 | 0.9 | 12031 | 16977 | 0.71 | 4759 | 6327 | 0.75 |
| Pediococcus_pentosaceus_ATCC_25745 (leading) | 659 | 721 | 0.91 | 636519 | 668469 | 0.95 | 31939 | 34376 | 0.93 | 21450 | 22535 | 0.95 |
| Pediococcus_pentosaceus_ATCC_25745 (lagging) | 204 | 170 | 1.2 | 160287 | 145455 | 1.1 | -2069 | 356 | -5.81 | 1416 | 457 | 3.1 |
| Pelobacter_carbinolicus (leading) | 1008 | 1079 | 0.93 | 963363 | 1043112 | 0.92 | 35750 | 34672 | 1.03 | -20617 | -24482 | 0.84 |
| Pelobacter_carbinolicus (lagging) | 675 | 589 | 1.15 | 639375 | 551820 | 1.16 | -20161 | -16471 | 1.22 | 16172 | 10701 | 1.51 |
| Pelobacter_propionicus_DSM_2379.3 (leading) | 1043 | 1002 | 1.04 | 998793 | 1016514 | 0.98 | 10379 | 9563 | 1.09 | 8716 | 3225 | 2.7 |
| Pelobacter_propionicus_DSM_2379.3 (lagging) | 787 | 743 | 1.06 | 763785 | 751962 | 1.02 | -12104 | -11893 | 1.02 | 23647 | 24903 | 0.95 |
| Pelodictyon_luteolum_DSM_273 (leading) | 615 | 571 | 1.08 | 665301 | 556242 | 1.2 | 3464 | 2794 | 1.24 | -373 | -5694 | 0.07 |
| Pelodictyon_luteolum_DSM_273 (lagging) | 388 | 508 | 0.76 | 380304 | 505035 | 0.75 | -16443 | -21776 | 0.76 | 18771 | 24613 | 0.76 |
| Pelotomaculum_thermopropionicum_SI (leading) | 1163 | 1095 | 1.06 | 1085628 | 983184 | 1.1 | 59023 | 51081 | 1.16 | 27623 | 23703 | 1.17 |
| Pelotomaculum_thermopropionicum_SI (lagging) | 287 | 374 | 0.77 | 231039 | 313734 | 0.74 | 4191 | 2389 | 1.75 | 7976 | 11853 | 0.67 |
| Photobacterium_profundum_SS9.2 (leading) | 1020 | 915 | 1.11 | 1006023 | 907425 | 1.11 | 50140 | 46165 | 1.09 | -4549 | -4991 | 0.91 |
| Photobacterium_profundum_SS9.2 (lagging) | 703 | 777 | 0.9 | 694374 | 750111 | 0.93 | 2422 | 3875 | 0.63 | 19076 | 14678 | 1.3 |
| Photorhabdus_luminescens (leading) | 1215 | 1475 | 0.82 | 1205541 | 1508139 | 0.8 | 65499 | 88436 | 0.74 | -6846 | -13407 | 0.51 |
| Photorhabdus_luminescens (lagging) | 1070 | 922 | 1.16 | 1075371 | 824604 | 1.3 | 3485 | -716 | -4.87 | 31120 | 21318 | 1.46 |
| Picrophilus_torridus_DSM_9790 (leading) | 358 | 367 | 0.98 | 311964 | 333138 | 0.94 | 13388 | 15353 | 0.87 | 22594 | 20507 | 1.1 |
| Picrophilus_torridus_DSM_9790 (lagging) | 399 | 410 | 0.97 | 386358 | 386358 | 1 | 16617 | 15138 | 1.1 | 30035 | 27158 | 1.11 |
| Pirellula_sp (leading) | 1844 | 1774 | 1.04 | 1719549 | 1692759 | 1.02 | 11117 | 23176 | 0.48 | 19408 | 11207 | 1.73 |
| Pirellula_sp (lagging) | 1923 | 1783 | 1.08 | 1750767 | 1777167 | 0.99 | 22607 | 5498 | 4.11 | 9886 | 21717 | 0.46 |
| Polaromonas_JS666 (leading) | 1361 | 1310 | 1.04 | 1289967 | 1278237 | 1.01 | 16292 | 14191 | 1.15 | -1755 | -3922 | 0.45 |
| Polaromonas_JS666 (lagging) | 1072 | 1073 | 1 | 990105 | 1014921 | 0.98 | -12367 | -12950 | 0.95 | 9184 | 14457 | 0.64 |
| Polaromonas_naphthalenivorans_CJ2.9 (leading) | 1125 | 1042 | 1.08 | 1071384 | 1030299 | 1.04 | 4156 | 4545 | 0.91 | 3270 | 4758 | 0.69 |
| Polaromonas_naphthalenivorans_CJ2.9 (lagging) | 933 | 983 | 0.95 | 911913 | 963936 | 0.95 | -9688 | -12775 | 0.76 | 12193 | 17741 | 0.69 |
| Polynucleobacter_QLW-P1DMWA-1 (leading) | 659 | 648 | 1.02 | 621903 | 679452 | 0.92 | 23854 | 26165 | 0.91 | -10625 | -10211 | 1.04 |
| Polynucleobacter_QLW-P1DMWA-1 (lagging) | 411 | 358 | 1.15 | 378984 | 324174 | 1.17 | 2179 | -41 | -53.15 | -771 | 2377 | -0.32 |
| Porphyromonas_gingivalis_W83 (leading) | 496 | 528 | 0.94 | 537870 | 509484 | 1.06 | 10948 | 10058 | 1.09 | 9138 | 5780 | 1.58 |
| Porphyromonas_gingivalis_W83 (lagging) | 459 | 425 | 1.08 | 453765 | 433494 | 1.05 | 1131 | 3670 | 0.31 | 13278 | 11044 | 1.2 |
| Prochlorococcus_marinus_AS9601 (leading) | 474 | 490 | 0.97 | 372570 | 378873 | 0.98 | 20689 | 19629 | 1.05 | 12985 | 14010 | 0.93 |
| Prochlorococcus_marinus_AS9601 (lagging) | 491 | 465 | 1.06 | 392268 | 373431 | 1.05 | 14516 | 14506 | 1 | 28536 | 26945 | 1.06 |
| Prochlorococcus_marinus_CCMP1375 (leading) | 480 | 454 | 1.06 | 378609 | 360885 | 1.05 | 21453 | 20865 | 1.03 | -2678 | -3064 | 0.87 |
| Prochlorococcus_marinus_CCMP1375 (lagging) | 476 | 472 | 1.01 | 403020 | 412581 | 0.98 | 10331 | 10216 | 1.01 | 25529 | 26507 | 0.96 |
| Prochlorococcus_marinus_MED4 (leading) | 429 | 445 | 0.96 | 353086 | 385680 | 0.92 | 19668 | 19791 | 0.99 | 12027 | 13487 | 0.89 |
| Prochlorococcus_marinus_MED4 (lagging) | 441 | 401 | 1.1 | 374319 | 346068 | 1.08 | 15203 | 13553 | 1.12 | 26376 | 23857 | 1.11 |
| Prochlorococcus_marinus_MIT9313 (leading) | 570 | 516 | 1.1 | 479559 | 457398 | 1.05 | 13381 | 27473 | 0.49 | -9982 | -30299 | 0.33 |
| Prochlorococcus_marinus_MIT9313 (lagging) | 542 | 640 | 0.85 | 474714 | 563443 | 0.84 | 5137 | -13778 | -0.37 | -775 | 17664 | -0.04 |
| Prochlorococcus_marinus_MIT_9301 (leading) | 469 | 482 | 0.97 | 364689 | 377256 | 0.97 | 20121 | 20119 | 1 | 12138 | 14451 | 0.84 |
| Prochlorococcus_marinus_MIT_9301 (lagging) | 483 | 472 | 1.02 | 385902 | 365550 | 1.06 | 14246 | 13819 | 1.03 | 28184 | 25529 | 1.1 |
| Prochlorococcus_marinus_MIT_9303 (leading) | 765 | 683 | 1.12 | 539808 | 537138 | 1 | 25642 | 24562 | 1.04 | -25428 | -23350 | 1.09 |
| Prochlorococcus_marinus_MIT_9303 (lagging) | 760 | 788 | 0.96 | 584094 | 615135 | 0.95 | -6904 | -5147 | 1.34 | 20296 | 13192 | 1.54 |

| Chromosome | Nu of genes 1st half (Nu1) | Nu of genes 2nd half (Nu2) | Nu1/Nu2 | Total length of genes 1st half (Length1) | Total length of genes 2nd half (Length2) | Length1/ Length2 | Cumulative skew (G-C) of genes 1st half (G-C)1 | Cumulative skew (G-C) of genes 2nd half (G-C)2 | (G-C)1/ (G-C)2 | Cumulative skew (A-T) of genes 1st half (A-T)1 | Cumulative skew (A-T) of genes 2nd half (A-T)2 | (A-T)1/ (A-T)2 |
|---|---|---|---|---|---|---|---|---|---|---|---|---|
| Prochlorococcus_marinus_MIT_9312 (leading) | 458 | 464 | 0.99 | 383358 | 379560 | 1.01 | 21506 | 20914 | 1.03 | 13132 | 13828 | 0.95 |
| Prochlorococcus_marinus_MIT_9312 (lagging) | 463 | 424 | 1.09 | 395760 | 365883 | 1.08 | 14359 | 13371 | 1.07 | 27421 | 25956 | 1.06 |
| Prochlorococcus_marinus_MIT_9515 (leading) | 483 | 494 | 0.98 | 372366 | 392952 | 0.95 | 20265 | 20259 | 1 | 14741 | 13949 | 1.06 |
| Prochlorococcus_marinus_MIT_9515 (lagging) | 475 | 453 | 1.05 | 391209 | 353349 | 1.11 | 15235 | 13106 | 1.16 | 26184 | 24553 | 1.07 |
| Prochlorococcus_marinus_NATL1A (leading) | 501 | 612 | 0.82 | 418104 | 393486 | 1.06 | 23482 | 20987 | 1.12 | 1936 | 2611 | 0.74 |
| Prochlorococcus_marinus_NATL1A (lagging) | 499 | 580 | 0.86 | 408240 | 404643 | 1.01 | 12924 | 12327 | 1.05 | 28440 | 28450 | 1 |
| Prochlorococcus_marinus_NATL2A (leading) | 500 | 477 | 1.05 | 399369 | 422025 | 0.95 | 15637 | 17121 | 0.91 | 15998 | 16368 | 0.98 |
| Prochlorococcus_marinus_NATL2A (lagging) | 474 | 440 | 1.08 | 346842 | 401949 | 0.86 | 16426 | 19006 | 0.86 | 12710 | 13513 | 0.94 |
| Propionibacterium_acnes_KPA171202 (leading) | 703 | 698 | 1.01 | 700956 | 669603 | 1.05 | 14393 | 12548 | 1.15 | -22309 | -18853 | 1.18 |
| Propionibacterium_acnes_KPA171202 (lagging) | 455 | 440 | 1.03 | 455691 | 468309 | 0.97 | -18064 | -20586 | 0.88 | -313 | 1207 | -0.26 |
| Prosthecochloris_vibrioformis_DSM_265 (leading) | 505 | 487 | 1.04 | 538425 | 504075 | 1.07 | 22663 | 19995 | 1.13 | -11560 | -17154 | 0.67 |
| Prosthecochloris_vibrioformis_DSM_265 (lagging) | 357 | 403 | 0.89 | 351129 | 399951 | 0.88 | -12991 | -19014 | 0.68 | 18132 | 24467 | 0.74 |
| Pseudoalteromonas_atlantica_T6c (leading) | 1199 | 1269 | 0.94 | 1259868 | 1323372 | 0.95 | 44272 | 46614 | 0.95 | 12738 | 15930 | 0.8 |
| Pseudoalteromonas_atlantica_T6c (lagging) | 928 | 884 | 1.05 | 975234 | 922650 | 1.06 | 6247 | 4396 | 1.42 | 24811 | 23680 | 1.05 |
| Pseudoalteromonas_haloplanktis_TAC125 (leading) | 913 | 879 | 1.04 | 853543 | 875544 | 0.97 | 32289 | 31967 | 1.01 | 21225 | 21495 | 0.99 |
| Pseudoalteromonas_haloplanktis_TAC125 (lagging) | 596 | 551 | 1.08 | 542652 | 533295 | 1.02 | 2482 | 3574 | 0.69 | 17748 | 14943 | 1.19 |
| Pseudomonas_aeruginosa (leading) | 1533 | 1583 | 0.97 | 1507230 | 1604845 | 0.94 | -18225 | -3712 | 4.91 | 9153 | 3116 | 2.94 |
| Pseudomonas_aeruginosa (lagging) | 1240 | 1211 | 1.02 | 1288647 | 1200159 | 1.07 | -39676 | -56219 | 0.71 | 14431 | 22286 | 0.65 |
| Pseudomonas_aeruginosa_PA7 (leading) | 1669 | 1694 | 0.99 | 1616376 | 1618008 | 1 | -2697 | -1924 | 1.4 | 5099 | 5664 | 0.9 |
| Pseudomonas_aeruginosa_PA7 (lagging) | 1478 | 1444 | 1.02 | 1333752 | 1334355 | 1 | -55235 | -56769 | 0.97 | 21111 | 26150 | 0.81 |
| Pseudomonas_aeruginosa_UCBPP-PA14 (leading) | 1568 | 1624 | 0.97 | 1610589 | 1624848 | 0.99 | -3075 | -4694 | 0.66 | 3660 | 4298 | 0.85 |
| Pseudomonas_aeruginosa_UCBPP-PA14 (lagging) | 1365 | 1334 | 1.02 | 1307511 | 1302504 | 1 | -57352 | -58153 | 0.99 | 20892 | 25058 | 0.83 |
| Pseudomonas_entomophila_L48 (leading) | 1417 | 1475 | 0.96 | 1447129 | 1544526 | 0.94 | 9616 | 10004 | 0.96 | 4224 | 5288 | 0.8 |
| Pseudomonas_entomophila_L48 (lagging) | 1147 | 1094 | 1.05 | 1149222 | 1090611 | 1.05 | -42938 | -38793 | 1.11 | 21914 | 21170 | 1.04 |
| Pseudomonas_fluorescens_Pf-5 (leading) | 1669 | 1584 | 1.05 | 1825732 | 1597836 | 1.14 | 8435 | 12198 | 0.69 | 1660 | -7280 | -0.23 |
| Pseudomonas_fluorescens_Pf-5 (lagging) | 1354 | 1530 | 0.88 | 1326192 | 1531554 | 0.87 | -53199 | -60521 | 0.88 | 26747 | 32271 | 0.83 |
| Pseudomonas_fluorescens_PfO-1 (leading) | 1501 | 1513 | 0.99 | 1603797 | 1482927 | 1.08 | 18440 | 21253 | 0.87 | 5911 | 1462 | 4.04 |
| Pseudomonas_fluorescens_PfO-1 (lagging) | 1291 | 1430 | 0.9 | 1304001 | 1401009 | 0.93 | -24479 | -27625 | 0.89 | 20700 | 25740 | 0.8 |
| Pseudomonas_mendocina_ymp (leading) | 1215 | 1267 | 0.96 | 1224429 | 1254693 | 0.98 | 6714 | 7019 | 0.96 | -4751 | -3628 | 1.31 |
| Pseudomonas_mendocina_ymp (lagging) | 1066 | 1045 | 1.02 | 1051440 | 1008762 | 1.04 | -36212 | -38939 | 0.93 | 18790 | 17367 | 1.08 |
| Pseudomonas_putida_F1 (leading) | 1356 | 1482 | 0.91 | 1398246 | 1463049 | 0.96 | 18551 | 23765 | 0.78 | -1611 | -2762 | 0.58 |
| Pseudomonas_putida_F1 (lagging) | 1215 | 1198 | 1.01 | 1240659 | 1186911 | 1.05 | -26455 | -27537 | 0.96 | 19708 | 20226 | 0.97 |
| Pseudomonas_putida_KT2440 (leading) | 1476 | 1397 | 1.06 | 1471242 | 1400133 | 1.05 | 21090 | 12584 | 1.68 | 432 | 447 | 0.97 |
| Pseudomonas_putida_KT2440 (lagging) | 1197 | 1279 | 0.94 | 1226226 | 1267062 | 0.97 | -29494 | -18165 | 1.62 | 23270 | 16047 | 1.45 |
| Pseudomonas_stutzeri_A1501 (leading) | 1128 | 1095 | 1.03 | 1100451 | 1079121 | 1.02 | 12711 | 12704 | 1 | -2290 | -4009 | 0.57 |
| Pseudomonas_stutzeri_A1501 (lagging) | 936 | 968 | 0.97 | 935223 | 972486 | 0.96 | -27662 | -26589 | 1.04 | 14277 | 12449 | 1.15 |
| Pseudomonas_syringae_phaseolicola_1448A (leading) | 1311 | 1354 | 0.97 | 1340616 | 1289185 | 1.04 | 28535 | 24199 | 1.18 | -10581 | -10071 | 1.05 |
| Pseudomonas_syringae_phaseolicola_1448A (lagging) | 1142 | 1176 | 0.97 | 1158129 | 1201935 | 0.96 | -9553 | -7304 | 1.31 | 21458 | 12933 | 1.66 |
| Pseudomonas_syringae_pv_B728a (leading) | 1337 | 1328 | 1.01 | 1452546 | 1381506 | 1.05 | 22420 | 25585 | 0.88 | -8956 | -5987 | 1.5 |
| Pseudomonas_syringae_pv_B728a (lagging) | 1224 | 1199 | 1.02 | 1221462 | 1290306 | 0.95 | -13027 | -13491 | 0.97 | 20733 | 19477 | 1.06 |
| Pseudomonas_syringae_tomato_DC3000 (leading) | 1540 | 1373 | 1.12 | 1532739 | 1368102 | 1.12 | 25423 | 25264 | 1.01 | -5106 | -4576 | 1.12 |
| Pseudomonas_syringae_tomato_DC3000 (lagging) | 1247 | 1308 | 0.95 | 1204524 | 1340343 | 0.9 | -9141 | -11699 | 0.78 | 18769 | 20220 | 0.93 |
| Psychrobacter_arcticum_273-4 (leading) | 658 | 554 | 1.19 | 642765 | 604200 | 1.06 | 24850 | 23389 | 1.06 | 4241 | 3159 | 1.34 |
| Psychrobacter_arcticum_273-4 (lagging) | 430 | 477 | 0.9 | 420153 | 470568 | 0.89 | 6556 | 6945 | 0.94 | 6219 | 2721 | 2.29 |
| Psychrobacter_cryohalolentis_K5.2 (leading) | 703 | 689 | 1.02 | 716796 | 723681 | 0.99 | 28840 | 27359 | 1.05 | 3184 | 3150 | 1.01 |
| Psychrobacter_cryohalolentis_K5.2 (lagging) | 552 | 522 | 1.06 | 563166 | 541419 | 1.04 | 8442 | 9446 | 0.89 | 5662 | 1477 | 3.83 |
| Psychrobacter_PRwf-1.3 (leading) | 673 | 663 | 1.02 | 699927 | 718038 | 0.97 | 22113 | 25173 | 0.88 | 6400 | 11381 | 0.56 |
| Psychrobacter_PRwf-1.3 (lagging) | 529 | 504 | 1.05 | 552738 | 503940 | 1.1 | 1571 | 802 | 1.96 | 5321 | 5010 | 1.06 |
| Psychromonas_ingrahamii_37 (leading) | 1084 | 1072 | 1.01 | 1105695 | 1077606 | 1.03 | 47386 | 45245 | 1.05 | 1605 | 3309 | 0.49 |
| Psychromonas_ingrahamii_37 (lagging) | 695 | 693 | 1 | 660654 | 696585 | 0.95 | 10769 | 9951 | 1.08 | 12331 | 14556 | 0.85 |
| Pyrobaculum_aerophilum (leading) | 701 | 646 | 1.09 | 542835 | 483882 | 1.12 | 35257 | 31640 | 1.11 | 14846 | 13858 | 1.07 |
| Pyrobaculum_aerophilum (lagging) | 597 | 660 | 0.9 | 430800 | 517245 | 0.83 | 28803 | 35237 | 0.82 | 9917 | 16052 | 0.62 |
| Pyrobaculum_arsenaticum_DSM_13514 (leading) | 579 | 611 | 0.95 | 452235 | 502302 | 0.9 | 27034 | 31284 | 0.86 | 10585 | 14934 | 0.71 |
| Pyrobaculum_arsenaticum_DSM_13514 (lagging) | 545 | 562 | 0.97 | 441630 | 443277 | 1 | 24580 | 25779 | 0.95 | 9106 | 12932 | 0.7 |
| Pyrobaculum_calidifontis_JCM_11548 (leading) | 531 | 570 | 0.93 | 470397 | 475365 | 0.99 | 27587 | 28526 | 0.97 | 10398 | 7617 | 1.37 |
| Pyrobaculum_calidifontis_JCM_11548 (lagging) | 520 | 527 | 0.99 | 440520 | 436470 | 1.01 | 28749 | 28423 | 1.01 | 9803 | 10007 | 0.98 |
| Pyrobaculum_islandicum_DSM_4184 (leading) | 481 | 449 | 1.07 | 382944 | 352110 | 1.09 | 21547 | 20954 | 1.03 | 15295 | 11126 | 1.37 |
| Pyrobaculum_islandicum_DSM_4184 (lagging) | 521 | 526 | 0.99 | 426651 | 430632 | 0.99 | 24525 | 25954 | 0.94 | 16432 | 13022 | 1.26 |
| Ralstonia_eutropha_H16 (leading) | 1034 | 1097 | 0.94 | 1026123 | 1092639 | 0.94 | 10464 | 10818 | 0.97 | 4929 | 8221 | 0.6 |
| Ralstonia_eutropha_H16 (lagging) | 782 | 737 | 1.06 | 752295 | 689916 | 1.09 | -11892 | -8748 | 1.36 | 6835 | 3662 | 1.87 |
| Ralstonia_eutropha_JMP134.3 (leading) | 987 | 1013 | 0.97 | 985503 | 1032567 | 0.95 | 11771 | 15415 | 0.76 | 1714 | 6198 | 0.28 |
| Ralstonia_eutropha_JMP134.3 (lagging) | 745 | 693 | 1.08 | 711921 | 654918 | 1.09 | -8381 | -6765 | 1.24 | 5656 | 5105 | 1.11 |
| Ralstonia_metallidurans_CH34.3 (leading) | 1023 | 1021 | 1 | 1032243 | 1016859 | 1.02 | 19330 | 20957 | 0.92 | -1759 | 3502 | -0.5 |
| Ralstonia_metallidurans_CH34.3 (lagging) | 781 | 775 | 1.01 | 745503 | 722664 | 1.03 | -9275 | -7447 | 1.25 | 9394 | 3869 | 2.43 |
| Ralstonia_solanacearum (leading) | 1020 | 1031 | 0.99 | 993567 | 993900 | 1 | 10051 | 6143 | 1.64 | 3880 | 6115 | 0.63 |
| Ralstonia_solanacearum (lagging) | 715 | 673 | 1.06 | 642363 | 639627 | 1 | -18795 | -10310 | 1.82 | 11018 | 7463 | 1.48 |
| Rhizobium_etli_CFN_42.2 (leading) | 1124 | 1046 | 1.07 | 1035468 | 988491 | 1.05 | -13386 | 874 | -15.32 | -1962 | -6351 | 0.31 |

| Chromosome | Nu of genes 1st half (Nu1) | Nu of genes 2nd half (Nu2) | Nu1/Nu2 | Total length of genes 1st half (Length1) | Total length of genes 2nd half (Length2) | Length1/ Length2 | Cumulative skew (G-C) of genes 1st half (G-C)1 | Cumulative skew (G-C) of genes 2nd half (G-C)2 | (G-C)1/ (G-C)2 | Cumulative skew (A-T) of genes 1st half (A-T)1 | Cumulative skew (A-T) of genes 2nd half (A-T)2 | (A-T)1/ (A-T)2 |
|---|---|---|---|---|---|---|---|---|---|---|---|---|
| Rhizobium_etli_CFN_42.2 (lagging) | 919 | 945 | 0.97 | 851055 | 899793 | 0.95 | -13211 | -28719 | 0.46 | 4348 | 1994 | 2.18 |
| Rhizobium_leguminosarum_bv_viciae_3841.3 (leading) | 1247 | 1235 | 1.01 | 1134015 | 3882570 | 0.29 | -15274 | -161 | 94.87 | -1373 | -6859 | 0.2 |
| Rhizobium_leguminosarum_bv_viciae_3841.3 (lagging) | 1138 | 1079 | 1.05 | 1046568 | 1021455 | 1.02 | -12958 | -34034 | 0.38 | 5256 | 3621 | 1.45 |
| Rhizobium_leguminosarum_bv_viciae_3841 (leading) | 189 | 200 | 0.95 | 180339 | 187080 | 0.96 | -2198 | -3713 | 0.59 | -1307 | -675 | 1.94 |
| Rhizobium_leguminosarum_bv_viciae_3841 (lagging) | 201 | 189 | 1.06 | 214608 | 203988 | 1.05 | -1907 | -503 | 3.79 | -525 | -887 | 0.59 |
| Rhodobacter_sphaeroides_2_4_1.4 (leading) | 768 | 839 | 0.92 | 709626 | 802077 | 0.88 | 5948 | 12661 | 0.47 | -5540 | -8189 | 0.68 |
| Rhodobacter_sphaeroides_2_4_1.4 (lagging) | 739 | 675 | 1.09 | 698700 | 629661 | 1.11 | -1795 | -4831 | 0.37 | -2271 | -5868 | 0.39 |
| Rhodobacter_sphaeroides_ATCC_17025 (leading) | 923 | 856 | 1.08 | 847260 | 808314 | 1.05 | 16633 | 9219 | 1.8 | -6999 | -3595 | 1.95 |
| Rhodobacter_sphaeroides_ATCC_17025 (lagging) | 626 | 705 | 0.89 | 583968 | 649509 | 0.9 | -1477 | 3322 | -0.44 | -3245 | -4601 | 0.71 |
| Rhodococcus_RHA1.2 (leading) | 297 | 293 | 1.01 | 234066 | 226488 | 1.03 | -10094 | -9046 | 1.12 | 3482 | 3924 | 0.89 |
| Rhodococcus_RHA1.2 (lagging) | 288 | 267 | 1.08 | 227727 | 234126 | 0.97 | -2495 | -1474 | 1.69 | 2240 | 2132 | 1.05 |
| Rhodococcus_RHA1 (leading) | 1638 | 1519 | 1.08 | 1503348 | 1495407 | 1.01 | -43495 | -43773 | 0.99 | 9959 | 8152 | 1.22 |
| Rhodococcus_RHA1 (lagging) | 2021 | 2032 | 0.99 | 2024205 | 2095995 | 0.97 | -8070 | 3338 | -2.42 | 7103 | -2761 | -2.57 |
| Rhodoferax_ferrireducens_T118.2 (leading) | 1147 | 1114 | 1.03 | 1135149 | 1186035 | 0.96 | 23979 | 22629 | 1.06 | -14188 | -5782 | 2.45 |
| Rhodoferax_ferrireducens_T118.2 (lagging) | 977 | 931 | 1.05 | 991854 | 920565 | 1.08 | -1438 | -893 | 1.61 | 2384 | 5488 | 0.43 |
| Rhodopseudomonas_palustris_BisA53 (leading) | 1352 | 1363 | 0.99 | 1303947 | 1324554 | 0.98 | 7101 | 8032 | 0.88 | 2586 | 4248 | 0.61 |
| Rhodopseudomonas_palustris_BisA53 (lagging) | 1123 | 1039 | 1.08 | 1042494 | 1053708 | 0.99 | -8770 | -7432 | 1.18 | 5140 | 3520 | 1.46 |
| Rhodopseudomonas_palustris_BisB18 (leading) | 1447 | 1428 | 1.01 | 1393716 | 1423197 | 0.98 | 7478 | 10664 | 0.7 | -74 | -3335 | 0.02 |
| Rhodopseudomonas_palustris_BisB18 (lagging) | 1066 | 944 | 1.13 | 959481 | 940878 | 1.02 | -6326 | -9191 | 0.69 | 6087 | 4191 | 1.45 |
| Rhodopseudomonas_palustris_BisB5 (leading) | 1181 | 1194 | 0.99 | 1144320 | 1135443 | 1.01 | 9012 | 4237 | 2.13 | -2652 | -1662 | 1.6 |
| Rhodopseudomonas_palustris_BisB5 (lagging) | 1003 | 1018 | 0.99 | 987240 | 983040 | 1 | -8925 | -3909 | 2.28 | 8863 | 7075 | 1.25 |
| Rhodopseudomonas_palustris_CGA009 (leading) | 1286 | 1310 | 0.98 | 1313873 | 1265544 | 1.04 | 9143 | 5710 | 1.6 | -5212 | -2534 | 2.06 |
| Rhodopseudomonas_palustris_CGA009 (lagging) | 1103 | 1113 | 0.99 | 1093347 | 1083774 | 1.01 | -15951 | -12577 | 1.27 | 7762 | 7387 | 1.05 |
| Rhodopseudomonas_palustris_HaA2 (leading) | 1290 | 1297 | 0.99 | 1354185 | 1283715 | 1.05 | 1091 | 4125 | 0.26 | 1584 | 692 | 2.29 |
| Rhodopseudomonas_palustris_HaA2 (lagging) | 1018 | 1077 | 0.95 | 984660 | 1031424 | 0.95 | -9939 | -12293 | 0.81 | 7275 | 8003 | 0.91 |
| Rhodospirillum_rubrum_ATCC_11170.2 (leading) | 958 | 996 | 0.96 | 995880 | 1003641 | 0.99 | 2065 | 500 | 4.13 | -24603 | -28293 | 0.87 |
| Rhodospirillum_rubrum_ATCC_11170.2 (lagging) | 915 | 921 | 0.99 | 915657 | 931320 | 0.98 | -28241 | -29897 | 0.94 | -11688 | -15837 | 0.74 |
| Rickettsia_bellii_RML369-C (leading) | 353 | 445 | 0.79 | 342000 | 399750 | 0.86 | 15240 | 20004 | 0.76 | 17072 | 21156 | 0.81 |
| Rickettsia_bellii_RML369-C (lagging) | 330 | 300 | 1.1 | 303469 | 251040 | 1.21 | 9604 | 6585 | 1.46 | 18836 | 13153 | 1.43 |
| Rickettsia_conorii (leading) | 379 | 446 | 0.85 | 321000 | 301281 | 1.07 | 16227 | 14360 | 1.13 | 12801 | 14063 | 0.91 |
| Rickettsia_conorii (lagging) | 273 | 275 | 0.99 | 198829 | 203469 | 0.98 | 3669 | 3912 | 0.94 | 11277 | 10267 | 1.1 |
| Rickettsia_felis_URRWXCal2 (leading) | 404 | 387 | 1.04 | 360591 | 328548 | 1.1 | 17247 | 16388 | 1.05 | 18370 | 16422 | 1.12 |
| Rickettsia_felis_URRWXCal2 (lagging) | 288 | 320 | 0.9 | 260193 | 294784 | 0.88 | 6066 | 8894 | 0.68 | 13455 | 18083 | 0.74 |
| Rickettsia_prowazekii (leading) | 270 | 241 | 1.12 | 273270 | 237441 | 1.15 | 14116 | 11978 | 1.18 | 7750 | 9001 | 0.86 |
| Rickettsia_prowazekii (lagging) | 165 | 158 | 1.04 | 155722 | 172762 | 0.9 | 3274 | 3810 | 0.86 | 9117 | 8217 | 1.11 |
| Rickettsia_typhi_wilmington (leading) | 225 | 248 | 0.91 | 226141 | 238413 | 0.95 | 10932 | 12463 | 0.88 | 8508 | 8528 | 1 |
| Rickettsia_typhi_wilmington (lagging) | 199 | 165 | 1.21 | 200301 | 173872 | 1.15 | 6028 | 3722 | 1.62 | 9667 | 8474 | 1.14 |
| Roseiflexus_RS-1 (leading) | 1133 | 1084 | 1.05 | 1248833 | 1187219 | 1.05 | 24995 | 27022 | 0.92 | -21355 | -24290 | 0.88 |
| Roseiflexus_RS-1 (lagging) | 1146 | 1153 | 0.99 | 1206861 | 1294492 | 0.93 | 16191 | 17009 | 0.95 | -11942 | -15777 | 0.76 |
| Roseobacter_denitrificans_OCh_114 (leading) | 1061 | 1046 | 1.01 | 952542 | 1018224 | 0.94 | 36288 | 36526 | 0.99 | -9546 | -10530 | 0.91 |
| Roseobacter_denitrificans_OCh_114 (lagging) | 958 | 880 | 1.09 | 905307 | 831981 | 1.09 | 12735 | 13622 | 0.93 | -1566 | -567 | 2.76 |
| Rubrobacter_xylanophilus_DSM_9941 (leading) | 907 | 1018 | 0.89 | 869493 | 946488 | 0.92 | 36016 | 38132 | 0.94 | -3703 | -2644 | 1.4 |
| Rubrobacter_xylanophilus_DSM_9941 (lagging) | 660 | 554 | 1.19 | 635457 | 513249 | 1.24 | 16631 | 13276 | 1.25 | 1588 | 3347 | 0.47 |
| Saccharophagus_degradans_2-40 (leading) | 1229 | 1272 | 0.97 | 1387368 | 1406595 | 0.99 | 59348 | 60775 | 0.98 | 13020 | 11512 | 1.13 |
| Saccharophagus_degradans_2-40 (lagging) | 773 | 733 | 1.05 | 804693 | 786411 | 1.02 | -17843 | -17706 | 1.01 | 43936 | 42787 | 1.03 |
| Saccharopolyspora_erythraea_NRRL_2338 (leading) | 2060 | 2055 | 1 | 2005383 | 2110140 | 0.95 | 27765 | 38725 | 0.72 | -3657 | -6023 | 0.61 |
| Saccharopolyspora_erythraea_NRRL_2338 (lagging) | 1608 | 1473 | 1.09 | 1476153 | 1383225 | 1.07 | -14915 | -12776 | 1.17 | 7458 | 5001 | 1.49 |
| Salinibacter_ruber_DSM_13855 (leading) | 806 | 776 | 1.04 | 826158 | 856465 | 0.96 | 14617 | 14628 | 1 | 12241 | 10702 | 1.14 |
| Salinibacter_ruber_DSM_13855 (lagging) | 580 | 638 | 0.91 | 633294 | 698976 | 0.91 | -18833 | -21219 | 0.89 | 5555 | 8281 | 0.67 |
| Salinispora_tropica_CNB-440 (leading) | 1340 | 1344 | 1 | 1369299 | 1414206 | 0.97 | 24416 | 23654 | 1.03 | -20921 | -25164 | 0.83 |
| Salinispora_tropica_CNB-440 (lagging) | 919 | 932 | 0.99 | 890886 | 900576 | 0.99 | -24810 | -21872 | 1.13 | 7452 | 4984 | 1.5 |
| Salmonella_enterica_Choleraesuis.3 (leading) | 1219 | 1233 | 0.99 | 1081749 | 1129677 | 0.96 | 38967 | 38635 | 1.01 | -2136 | -1410 | 1.51 |
| Salmonella_enterica_Choleraesuis.3 (lagging) | 1047 | 927 | 1.13 | 916617 | 859680 | 1.07 | 19090 | 18256 | 1.05 | -233 | 1996 | -0.12 |
| Salmonella_enterica_Paratypi_ATCC_9150 (leading) | 1109 | 1141 | 0.97 | 1038414 | 1044010 | 0.99 | 33541 | 37329 | 0.9 | -6321 | 2994 | -2.11 |
| Salmonella_enterica_Paratypi_ATCC_9150 (lagging) | 942 | 900 | 1.05 | 864135 | 837849 | 1.03 | 21977 | 19039 | 1.15 | -3232 | 1116 | -2.9 |
| Salmonella_typhi (leading) | 1159 | 1071 | 1.08 | 1057659 | 1024876 | 1.03 | 35965 | 33803 | 1.06 | -2641 | 970 | -2.72 |
| Salmonella_typhi (lagging) | 1110 | 1054 | 1.05 | 961071 | 955791 | 1.01 | 24942 | 25892 | 0.96 | -1493 | 799 | -1.87 |
| Salmonella_typhi_Ty2 (leading) | 1175 | 1069 | 1.1 | 1061172 | 1008022 | 1.05 | 36416 | 34078 | 1.07 | -5046 | 2155 | -2.34 |
| Salmonella_typhi_Ty2 (lagging) | 1006 | 1067 | 0.94 | 920568 | 959265 | 0.96 | 22030 | 26157 | 0.84 | -1548 | 958 | -1.62 |
| Salmonella_typhimurium_LT2 (leading) | 1185 | 1254 | 0.94 | 1126026 | 1199385 | 0.94 | 40503 | 40910 | 0.99 | -2931 | 2139 | -1.37 |
| Salmonella_typhimurium_LT2 (lagging) | 1056 | 929 | 1.14 | 982086 | 890961 | 1.1 | 21482 | 20420 | 1.05 | -2292 | 1807 | -1.27 |
| Shewanella_amazonensis_SB2B (leading) | 1042 | 1007 | 1.03 | 1115697 | 1016148 | 1.1 | 32703 | 27494 | 1.19 | -9816 | -9940 | 0.99 |
| Shewanella_amazonensis_SB2B (lagging) | 710 | 885 | 0.8 | 773382 | 887706 | 0.87 | -9145 | -9128 | 1 | 13137 | 14100 | 0.93 |
| Shewanella_ANA-3.2 (leading) | 1204 | 1139 | 1.06 | 1258461 | 1179285 | 1.07 | 41465 | 40327 | 1.03 | -5382 | -7166 | 0.75 |
| Shewanella_ANA-3.2 (lagging) | 857 | 910 | 0.94 | 886971 | 940677 | 0.94 | -12465 | -11673 | 1.07 | 21552 | 24440 | 0.88 |
| Shewanella_baltica_OS155.5 (leading) | 1261 | 1212 | 1.04 | 1219644 | 1203954 | 1.01 | 44176 | 45880 | 0.96 | -8644 | -5814 | 1.49 |
| Shewanella_baltica_OS155.5 (lagging) | 914 | 919 | 0.99 | 922035 | 929859 | 0.99 | -7434 | -7894 | 0.94 | 24359 | 28385 | 0.86 |

| Chromosome | Nu of genes 1st half (Nu1) | Nu of genes 2nd half (Nu2) | Nu1/Nu2 | Total length of genes 1st half (Length1) | Total length of genes 2nd half (Length2) | Length1/ Length2 | Cumulative skew (G-C) of genes 1st half (G-C)1 | Cumulative skew (G-C) of genes 2nd half (G-C)2 | (G-C)1/ (G-C)2 | Cumulative skew (A-T) of genes 1st half (A-T)1 | Cumulative skew (A-T) of genes 2nd half (A-T)2 | (A-T)1/ (A-T)2 |
|---|---|---|---|---|---|---|---|---|---|---|---|---|
| Shewanella_baltica_OS185.2 (leading) | 1280 | 1220 | 1.05 | 1268827 | 1253214 | 1.01 | 46951 | 47749 | 0.98 | -6950 | -11512 | 0.6 |
| Shewanella_baltica_OS185.2 (lagging) | 880 | 942 | 0.93 | 905952 | 950831 | 0.95 | -9704 | -9822 | 0.99 | 24384 | 26044 | 0.94 |
| Shewanella_denitrificans_OS217 (leading) | 1150 | 1146 | 1 | 1146915 | 1155426 | 0.99 | 36996 | 39275 | 0.94 | 8073 | 4185 | 1.93 |
| Shewanella_denitrificans_OS217 (lagging) | 759 | 698 | 1.09 | 789804 | 748821 | 1.05 | -4151 | -6852 | 0.61 | 18489 | 20357 | 0.91 |
| Shewanella_frigidimarina_NCIMB_400 (leading) | 1211 | 1135 | 1.07 | 1168179 | 1178262 | 0.99 | 45317 | 45553 | 0.99 | -4462 | -4409 | 1.01 |
| Shewanella_frigidimarina_NCIMB_400 (lagging) | 814 | 868 | 0.94 | 863157 | 868518 | 0.99 | 1641 | 3807 | 0.43 | 20022 | 18823 | 1.06 |
| Shewanella_loihica_PV-4 (leading) | 1181 | 1012 | 1.17 | 1161669 | 1046505 | 1.11 | 35980 | 33239 | 1.08 | -67 | 926 | -0.07 |
| Shewanella_loihica_PV-4 (lagging) | 800 | 865 | 0.92 | 828678 | 908310 | 0.91 | -19605 | -22179 | 0.88 | 29637 | 30779 | 0.96 |
| Shewanella_MR-4 (leading) | 1149 | 1077 | 1.07 | 1180089 | 1110423 | 1.06 | 40475 | 38820 | 1.04 | -6898 | -8237 | 0.84 |
| Shewanella_MR-4 (lagging) | 819 | 878 | 0.93 | 844860 | 900621 | 0.94 | -12894 | -11833 | 1.09 | 22584 | 21460 | 1.05 |
| Shewanella_MR-7.2 (leading) | 1198 | 1093 | 1.1 | 1192545 | 1140195 | 1.05 | 39783 | 39993 | 0.99 | -3320 | -8054 | 0.41 |
| Shewanella_MR-7.2 (lagging) | 816 | 898 | 0.91 | 850902 | 916347 | 0.93 | -9971 | -14419 | 0.69 | 21065 | 23752 | 0.89 |
| Shewanella_oneidensis (leading) | 1240 | 1156 | 1.07 | 1196718 | 1109391 | 1.08 | 47409 | 41982 | 1.13 | -11687 | -7154 | 1.63 |
| Shewanella_oneidensis (lagging) | 914 | 1007 | 0.91 | 875865 | 961515 | 0.91 | -6743 | -9218 | 0.73 | 20760 | 28540 | 0.73 |
| Shewanella_putrefaciens_CN-32 (leading) | 1102 | 1131 | 0.97 | 1056627 | 1143249 | 0.92 | 46294 | 49026 | 0.94 | -16563 | -11693 | 1.42 |
| Shewanella_putrefaciens_CN-32 (lagging) | 927 | 811 | 1.14 | 927456 | 854904 | 1.08 | -2825 | -6221 | 0.45 | 21771 | 23481 | 0.93 |
| Shewanella_W3-18-1 (leading) | 1174 | 1157 | 1.01 | 1184109 | 1119588 | 1.06 | 50162 | 46430 | 1.08 | -10941 | -10850 | 1.01 |
| Shewanella_W3-18-1 (lagging) | 844 | 868 | 0.97 | 834354 | 877083 | 0.95 | -5177 | -4445 | 1.16 | 21167 | 22354 | 0.95 |
| Shigella_boydii_Sb227.2 (leading) | 1072 | 1055 | 1.02 | 946500 | 940422 | 1.01 | 31999 | 29527 | 1.08 | 2909 | 2099 | 1.39 |
| Shigella_boydii_Sb227.2 (lagging) | 1028 | 980 | 1.05 | 858339 | 894573 | 0.96 | 23548 | 24241 | 0.97 | 5569 | 3064 | 1.82 |
| Shigella_dysenteriae (leading) | 1094 | 1131 | 0.97 | 825390 | 936486 | 0.88 | 25392 | 29607 | 0.86 | 2820 | 2337 | 1.21 |
| Shigella_dysenteriae (lagging) | 1125 | 923 | 1.22 | 866100 | 746433 | 1.16 | 20248 | 16595 | 1.22 | 7196 | 4940 | 1.46 |
| Shigella_flexneri_2a (leading) | 1071 | 1072 | 1 | 953340 | 970842 | 0.98 | 30995 | 33263 | 0.93 | 3627 | -619 | -5.86 |
| Shigella_flexneri_2a (lagging) | 1051 | 987 | 1.06 | 879261 | 890700 | 0.99 | 24032 | 18985 | 1.27 | 4513 | 3153 | 1.43 |
| Shigella_flexneri_2a_2457T (leading) | 1056 | 1025 | 1.03 | 937680 | 962143 | 0.97 | 30961 | 30567 | 1.01 | 2793 | -1721 | -1.62 |
| Shigella_flexneri_2a_2457T (lagging) | 1015 | 971 | 1.05 | 829944 | 836982 | 0.99 | 22415 | 19931 | 1.12 | 3721 | 3920 | 0.95 |
| Shigella_flexneri_5_8401 (leading) | 1055 | 1091 | 0.97 | 951243 | 1011966 | 0.94 | 30125 | 32478 | 0.93 | 3812 | -1358 | -2.81 |
| Shigella_flexneri_5_8401 (lagging) | 1024 | 945 | 1.08 | 874068 | 860013 | 1.02 | 23834 | 21724 | 1.1 | 5170 | 4071 | 1.27 |
| Shigella_sonnei_Ss046 (leading) | 1108 | 1118 | 0.99 | 1013625 | 1050477 | 0.96 | 34383 | 33849 | 1.02 | 6240 | 4078 | 1.53 |
| Shigella_sonnei_Ss046 (lagging) | 1030 | 966 | 1.07 | 933336 | 887034 | 1.05 | 23171 | 22895 | 1.01 | 4441 | 2891 | 1.54 |
| Silicibacter_pomeroyi_DSS-3 (leading) | 1019 | 1035 | 0.98 | 993435 | 1000395 | 0.99 | 33829 | 28821 | 1.17 | -9520 | -9078 | 1.05 |
| Silicibacter_pomeroyi_DSS-3 (lagging) | 870 | 885 | 0.98 | 849036 | 847686 | 1 | 537 | 2968 | 0.18 | -3013 | -1942 | 1.55 |
| Silicibacter_TM1040.3 (leading) | 798 | 819 | 0.97 | 761067 | 721281 | 1.06 | 23004 | 22610 | 1.02 | -6911 | -8143 | 0.85 |
| Silicibacter_TM1040.3 (lagging) | 697 | 715 | 0.97 | 660366 | 706896 | 0.93 | -16 | -350 | 0.05 | -4 | -1910 | 0 |
| Sinorhizobium_medicae_WSM419.4 (leading) | 976 | 963 | 1.01 | 876744 | 914457 | 0.96 | 2252 | 2669 | 0.84 | -1752 | 92 | -19.04 |
| Sinorhizobium_medicae_WSM419.4 (lagging) | 807 | 782 | 1.03 | 766904 | 737370 | 1.04 | -3653 | -8946 | 0.41 | 2852 | 738 | 3.86 |
| Sinorhizobium_meliloti.2 (leading) | 963 | 898 | 1.07 | 898260 | 858648 | 1.05 | -1496 | 3461 | -0.43 | 820 | 229 | 3.58 |
| Sinorhizobium_meliloti.2 (lagging) | 722 | 757 | 0.95 | 675084 | 711348 | 0.95 | -12085 | -15896 | 0.76 | 3929 | 3654 | 1.08 |
| Sodalis_glossinidius_morsitans (leading) | 747 | 674 | 1.11 | 699909 | 572823 | 1.22 | 23654 | 14921 | 1.59 | 1785 | -3860 | -0.46 |
| Sodalis_glossinidius_morsitans (lagging) | 490 | 520 | 0.94 | 404682 | 446340 | 0.91 | 1730 | 3986 | 0.43 | 2864 | 2004 | 1.43 |
| Solibacter_usitatus_Ellin6076 (leading) | 2055 | 2061 | 1 | 2436042 | 2356926 | 1.03 | -4074 | 4775 | -0.85 | 13102 | 15315 | 0.86 |
| Solibacter_usitatus_Ellin6076 (lagging) | 1834 | 1875 | 0.98 | 2097225 | 2124351 | 0.99 | -9497 | -15804 | 0.6 | 17598 | 14873 | 1.18 |
| Sphingomonas_wittichii_RW1.3 (leading) | 1130 | 1103 | 1.02 | 1159908 | 1093734 | 1.06 | -18890 | -20507 | 0.92 | 3208 | 1395 | 2.3 |
| Sphingomonas_wittichii_RW1.3 (lagging) | 1275 | 1341 | 0.95 | 1334190 | 1359492 | 0.98 | -4513 | -5155 | 0.88 | 2037 | -3959 | -0.51 |
| Sphingopyxis_alaskensis_RB2256.2 (leading) | 750 | 862 | 0.87 | 724314 | 816648 | 0.89 | 9127 | 7709 | 1.18 | 339 | -2427 | -0.14 |
| Sphingopyxis_alaskensis_RB2256.2 (lagging) | 832 | 720 | 1.16 | 789267 | 697410 | 1.13 | -1544 | -5363 | 0.29 | 1419 | 3313 | 0.43 |
| Staphylococcus_aureus_aureus_MRSA252 (leading) | 967 | 1017 | 0.95 | 907156 | 924588 | 0.98 | 48257 | 47504 | 1.02 | 51204 | 60952 | 0.84 |
| Staphylococcus_aureus_aureus_MRSA252 (lagging) | 352 | 319 | 1.1 | 283608 | 257826 | 1.1 | 3745 | 2859 | 1.31 | 11219 | 10985 | 1.02 |
| Staphylococcus_aureus_aureus_MSSA476 (leading) | 984 | 967 | 1.02 | 923623 | 867900 | 1.06 | 50126 | 45567 | 1.1 | 54388 | 51661 | 1.05 |
| Staphylococcus_aureus_aureus_MSSA476 (lagging) | 310 | 317 | 0.98 | 245331 | 257766 | 0.95 | 1956 | 3159 | 0.62 | 8761 | 10017 | 0.87 |
| Staphylococcus_aureus_COL (leading) | 1018 | 969 | 1.05 | 919732 | 899977 | 1.02 | 49711 | 45474 | 1.09 | 55798 | 56918 | 0.98 |
| Staphylococcus_aureus_COL (lagging) | 317 | 310 | 1.02 | 246069 | 257187 | 0.96 | 2113 | 3380 | 0.63 | 9074 | 10123 | 0.9 |
| Staphylococcus_aureus_JH1.2 (leading) | 1071 | 990 | 1.08 | 969517 | 910458 | 1.06 | 52103 | 45903 | 1.14 | 61539 | 58945 | 1.04 |
| Staphylococcus_aureus_JH1.2 (lagging) | 329 | 356 | 0.92 | 257490 | 295578 | 0.87 | 2259 | 4988 | 0.45 | 10721 | 12318 | 0.87 |
| Staphylococcus_aureus_JH9.2 (leading) | 1051 | 981 | 1.07 | 963888 | 911568 | 1.06 | 51826 | 45847 | 1.13 | 61008 | 59343 | 1.03 |
| Staphylococcus_aureus_JH9.2 (lagging) | 320 | 344 | 0.93 | 255393 | 292908 | 0.87 | 2338 | 5014 | 0.47 | 10565 | 12462 | 0.85 |
| Staphylococcus_aureus_Mu50 (leading) | 988 | 1007 | 0.98 | 926916 | 924561 | 1 | 49552 | 46535 | 1.06 | 55350 | 60650 | 0.91 |
| Staphylococcus_aureus_Mu50 (lagging) | 359 | 342 | 1.05 | 282900 | 277602 | 1.02 | 2985 | 3516 | 0.85 | 10881 | 11388 | 0.96 |
| Staphylococcus_aureus_MW2 (leading) | 974 | 1027 | 0.95 | 923208 | 942831 | 0.98 | 49241 | 47906 | 1.03 | 52899 | 63351 | 0.84 |
| Staphylococcus_aureus_MW2 (lagging) | 315 | 315 | 1 | 251505 | 242256 | 1.04 | 2230 | 2229 | 1 | 8861 | 9481 | 0.93 |
| Staphylococcus_aureus_N315 (leading) | 935 | 999 | 0.94 | 913845 | 920739 | 0.99 | 48317 | 46760 | 1.03 | 51916 | 60449 | 0.86 |
| Staphylococcus_aureus_N315 (lagging) | 339 | 314 | 1.08 | 266088 | 249186 | 1.07 | 2616 | 2481 | 1.05 | 9954 | 10337 | 0.96 |
| Staphylococcus_aureus_NCTC_8325 (leading) | 994 | 1165 | 0.85 | 902349 | 944280 | 0.96 | 47649 | 50293 | 0.95 | 50272 | 62005 | 0.81 |
| Staphylococcus_aureus_NCTC_8325 (lagging) | 371 | 361 | 1.03 | 299049 | 253944 | 1.18 | 3038 | 2138 | 1.42 | 13701 | 9700 | 1.41 |
| Staphylococcus_aureus_Newman (leading) | 1005 | 1000 | 1.01 | 964059 | 944454 | 1.02 | 51464 | 48004 | 1.07 | 58075 | 63448 | 0.92 |
| Staphylococcus_aureus_Newman (lagging) | 300 | 308 | 0.97 | 243195 | 249885 | 0.97 | 2227 | 2768 | 0.8 | 8020 | 10005 | 0.8 |
| Staphylococcus_aureus_RF122 (leading) | 912 | 988 | 0.92 | 873501 | 862059 | 1.01 | 46650 | 44825 | 1.04 | 48219 | 50958 | 0.95 |

| Chromosome | Nu of genes 1st half (Nu1) | Nu of genes 2nd half (Nu2) | Nu1/Nu2 | Total length of genes 1st half (Length1) | Total length of genes 2nd half (Length2) | Length1/ Length2 | Cumulative skew (G-C) of genes 1st half (G-C)1 | Cumulative skew (G-C) of genes 2nd half (G-C)2 | (G-C)1/ (G-C)2 | Cumulative skew (A-T) of genes 1st half (A-T)1 | Cumulative skew (A-T) of genes 2nd half (A-T)2 | (A-T)1/ (A-T)2 |
|---|---|---|---|---|---|---|---|---|---|---|---|---|
| Staphylococcus_aureus_RF122 (lagging) | 301 | 313 | 0.96 | 234048 | 245796 | 0.95 | 2066 | 2305 | 0.9 | 7116 | 9997 | 0.71 |
| Staphylococcus_aureus_USA300.4 (leading) | 957 | 1001 | 0.96 | 923163 | 938448 | 0.98 | 49094 | 48521 | 1.01 | 53299 | 62379 | 0.85 |
| Staphylococcus_aureus_USA300.4 (lagging) | 323 | 278 | 1.16 | 263388 | 233274 | 1.13 | 2867 | 2504 | 1.14 | 9815 | 9764 | 1.01 |
| Staphylococcus_epidermidis_ATCC_12228 (leading) | 787 | 899 | 0.88 | 706398 | 800937 | 0.88 | 33526 | 37271 | 0.9 | 38338 | 47812 | 0.8 |
| Staphylococcus_epidermidis_ATCC_12228 (lagging) | 425 | 307 | 1.38 | 348156 | 237405 | 1.47 | 4282 | 2492 | 1.72 | 20796 | 9553 | 2.18 |
| Staphylococcus_epidermidis_RP62A (leading) | 733 | 889 | 0.82 | 649051 | 773526 | 0.84 | 31640 | 35861 | 0.88 | 33820 | 48469 | 0.7 |
| Staphylococcus_epidermidis_RP62A (lagging) | 505 | 366 | 1.38 | 438843 | 299712 | 1.46 | 10219 | 4203 | 2.43 | 27850 | 14175 | 1.96 |
| Staphylococcus_haemolyticus (leading) | 935 | 882 | 1.06 | 838686 | 776172 | 1.08 | 37086 | 36158 | 1.03 | 47448 | 47634 | 1 |
| Staphylococcus_haemolyticus (lagging) | 369 | 489 | 0.75 | 308547 | 386055 | 0.8 | 4807 | 7868 | 0.61 | 11892 | 21029 | 0.57 |
| Staphylococcus_saprophyticus (leading) | 884 | 825 | 1.07 | 784752 | 739983 | 1.06 | 41367 | 39639 | 1.04 | 43431 | 42352 | 1.03 |
| Staphylococcus_saprophyticus (lagging) | 331 | 405 | 0.82 | 273519 | 308523 | 0.89 | 1772 | 4037 | 0.44 | 9611 | 12764 | 0.75 |
| Staphylothermus_marinus_F1 (leading) | 425 | 273 | 1.56 | 365751 | 233304 | 1.57 | 21113 | 14163 | 1.49 | 23572 | 16235 | 1.45 |
| Staphylothermus_marinus_F1 (lagging) | 347 | 524 | 0.66 | 314964 | 454947 | 0.69 | 17075 | 24436 | 0.7 | 19949 | 33761 | 0.59 |
| Streptococcus_agalactiae_2603 (leading) | 783 | 847 | 0.92 | 702852 | 754935 | 0.93 | 32780 | 35117 | 0.93 | 16148 | 11218 | 1.44 |
| Streptococcus_agalactiae_2603 (lagging) | 260 | 233 | 1.12 | 212628 | 198333 | 1.07 | 1736 | -1026 | -1.69 | 6570 | 5801 | 1.13 |
| Streptococcus_agalactiae_A909 (leading) | 797 | 794 | 1 | 749662 | 738561 | 1.02 | 34494 | 32518 | 1.06 | 17869 | 10025 | 1.78 |
| Streptococcus_agalactiae_A909 (lagging) | 189 | 215 | 0.88 | 153594 | 191580 | 0.8 | -44 | -1095 | 0.04 | 5234 | 6079 | 0.86 |
| Streptococcus_agalactiae_NEM316 (leading) | 788 | 831 | 0.95 | 739788 | 789441 | 0.94 | 33797 | 34952 | 0.97 | 17369 | 13243 | 1.31 |
| Streptococcus_agalactiae_NEM316 (lagging) | 251 | 223 | 1.13 | 208071 | 201768 | 1.03 | 1416 | -1417 | -1 | 7681 | 5813 | 1.32 |
| Streptococcus_mutans (leading) | 761 | 801 | 0.95 | 700704 | 727566 | 0.96 | 30816 | 32663 | 0.94 | -3648 | -1047 | 3.48 |
| Streptococcus_mutans (lagging) | 216 | 181 | 1.19 | 173565 | 142377 | 1.22 | 1317 | -1174 | -1.12 | 858 | 199 | 4.31 |
| Streptococcus_pneumoniae_D39 (leading) | 784 | 764 | 1.03 | 724944 | 683001 | 1.06 | 31606 | 29612 | 1.07 | 8382 | 6879 | 1.22 |
| Streptococcus_pneumoniae_D39 (lagging) | 164 | 201 | 0.82 | 126285 | 177312 | 0.71 | -2433 | -2180 | 1.12 | 3294 | 3970 | 0.83 |
| Streptococcus_pneumoniae_R6 (leading) | 816 | 794 | 1.03 | 740943 | 701067 | 1.06 | 32659 | 30893 | 1.06 | 8850 | 7448 | 1.19 |
| Streptococcus_pneumoniae_R6 (lagging) | 219 | 213 | 1.03 | 148320 | 182616 | 0.81 | -3138 | -2489 | 1.26 | 3808 | 4143 | 0.92 |
| Streptococcus_pneumoniae_TIGR4 (leading) | 865 | 819 | 1.06 | 755275 | 723339 | 1.04 | 33177 | 29589 | 1.12 | 10099 | 5490 | 1.84 |
| Streptococcus_pneumoniae_TIGR4 (lagging) | 205 | 215 | 0.95 | 142662 | 182241 | 0.78 | -3310 | -2561 | 1.29 | 3600 | 4230 | 0.85 |
| Streptococcus_pyogenes_M1_GAS (leading) | 717 | 605 | 1.19 | 641331 | 582999 | 1.1 | 24141 | 21593 | 1.12 | 11308 | 8270 | 1.37 |
| Streptococcus_pyogenes_M1_GAS (lagging) | 143 | 231 | 0.62 | 121014 | 206079 | 0.59 | -1905 | -759 | 2.51 | 2217 | 3604 | 0.62 |
| Streptococcus_pyogenes_Manfredo (leading) | 702 | 679 | 1.03 | 620523 | 615136 | 1.01 | 23450 | 23119 | 1.01 | 9235 | 11184 | 0.83 |
| Streptococcus_pyogenes_Manfredo (lagging) | 166 | 197 | 0.84 | 145662 | 159453 | 0.91 | -1861 | -1854 | 1 | 2609 | 3151 | 0.83 |
| Streptococcus_pyogenes_MGAS10270 (leading) | 814 | 737 | 1.1 | 702960 | 661323 | 1.06 | 26536 | 25726 | 1.03 | 12032 | 12997 | 0.93 |
| Streptococcus_pyogenes_MGAS10270 (lagging) | 199 | 235 | 0.85 | 137964 | 183537 | 0.75 | -2141 | -2040 | 1.05 | 2021 | 4073 | 0.5 |
| Streptococcus_pyogenes_MGAS10394 (leading) | 741 | 714 | 1.04 | 659082 | 652998 | 1.01 | 23619 | 26020 | 0.91 | 8411 | 16176 | 0.52 |
| Streptococcus_pyogenes_MGAS10394 (lagging) | 203 | 227 | 0.89 | 164178 | 181062 | 0.91 | -812 | -2005 | 0.4 | 1670 | 3945 | 0.42 |
| Streptococcus_pyogenes_MGAS10750 (leading) | 833 | 679 | 1.23 | 702471 | 621156 | 1.13 | 26927 | 23100 | 1.17 | 12410 | 10664 | 1.16 |
| Streptococcus_pyogenes_MGAS10750 (lagging) | 187 | 279 | 0.67 | 136611 | 233304 | 0.59 | -2159 | 1277 | -1.69 | 2432 | 8479 | 0.29 |
| Streptococcus_pyogenes_MGAS2096 (leading) | 788 | 714 | 1.1 | 677154 | 648678 | 1.04 | 25483 | 26019 | 0.98 | 10487 | 12787 | 0.82 |
| Streptococcus_pyogenes_MGAS2096 (lagging) | 181 | 214 | 0.85 | 129159 | 169884 | 0.76 | -2169 | -2087 | 1.04 | 1770 | 2559 | 0.69 |
| Streptococcus_pyogenes_MGAS315 (leading) | 706 | 774 | 0.91 | 642534 | 656727 | 0.98 | 22470 | 26541 | 0.85 | 8204 | 17710 | 0.46 |
| Streptococcus_pyogenes_MGAS315 (lagging) | 185 | 199 | 0.93 | 163719 | 166185 | 0.99 | -460 | -1652 | 0.28 | 1565 | 3575 | 0.44 |
| Streptococcus_pyogenes_MGAS5005 (leading) | 736 | 733 | 1 | 643902 | 636555 | 1.01 | 22972 | 24887 | 0.92 | 7062 | 12340 | 0.57 |
| Streptococcus_pyogenes_MGAS5005 (lagging) | 192 | 203 | 0.95 | 145032 | 167568 | 0.87 | -1650 | -2008 | 0.82 | 1398 | 3362 | 0.42 |
| Streptococcus_pyogenes_MGAS6180 (leading) | 723 | 748 | 0.97 | 657618 | 659100 | 1 | 23703 | 26430 | 0.9 | 7665 | 17784 | 0.43 |
| Streptococcus_pyogenes_MGAS6180 (lagging) | 203 | 219 | 0.93 | 164010 | 171096 | 0.96 | -1012 | -1963 | 0.52 | 1062 | 4005 | 0.27 |
| Streptococcus_pyogenes_MGAS8232 (leading) | 755 | 721 | 1.05 | 666372 | 635235 | 1.05 | 24523 | 24962 | 0.98 | 10823 | 14033 | 0.77 |
| Streptococcus_pyogenes_MGAS8232 (lagging) | 162 | 206 | 0.79 | 134880 | 177828 | 0.76 | -2170 | -2154 | 1.01 | 2106 | 3564 | 0.59 |
| Streptococcus_pyogenes_MGAS9429 (leading) | 787 | 669 | 1.18 | 671316 | 608871 | 1.1 | 25819 | 22658 | 1.14 | 12935 | 9623 | 1.34 |
| Streptococcus_pyogenes_MGAS9429 (lagging) | 169 | 251 | 0.67 | 125367 | 206196 | 0.61 | -2163 | -817 | 2.65 | 2172 | 2999 | 0.72 |
| Streptococcus_pyogenes_SSI-1 (leading) | 803 | 670 | 1.2 | 668910 | 614022 | 1.09 | 26775 | 21770 | 1.23 | 15875 | 9528 | 1.67 |
| Streptococcus_pyogenes_SSI-1 (lagging) | 159 | 228 | 0.7 | 126084 | 198534 | 0.64 | -1753 | -396 | 4.43 | 3125 | 1856 | 1.68 |
| Streptococcus_sanguinis_SK36 (leading) | 924 | 965 | 0.96 | 903747 | 888402 | 1.02 | 38851 | 40525 | 0.96 | 4854 | 5163 | 0.94 |
| Streptococcus_sanguinis_SK36 (lagging) | 178 | 202 | 0.88 | 159345 | 167736 | 0.95 | -3787 | -2728 | 1.39 | 2940 | 3452 | 0.85 |
| Streptococcus_suis_05ZYH33 (leading) | 763 | 884 | 0.86 | 649335 | 751629 | 0.86 | 26637 | 35240 | 0.76 | 9846 | 6553 | 1.5 |
| Streptococcus_suis_05ZYH33 (lagging) | 309 | 229 | 1.35 | 252849 | 184803 | 1.37 | 3611 | -5181 | -0.7 | 5866 | 4602 | 1.27 |
| Streptococcus_suis_98HAH33 (leading) | 766 | 866 | 0.88 | 647865 | 749535 | 0.86 | 26637 | 35112 | 0.76 | 9840 | 7019 | 1.4 |
| Streptococcus_suis_98HAH33 (lagging) | 319 | 233 | 1.37 | 256524 | 184104 | 1.39 | 3595 | -5299 | -0.68 | 5707 | 4487 | 1.27 |
| Streptococcus_thermophilus_CNRZ1066 (leading) | 763 | 746 | 1.02 | 609888 | 611100 | 1 | 24553 | 24224 | 1.01 | -147 | -4256 | 0.03 |
| Streptococcus_thermophilus_CNRZ1066 (lagging) | 192 | 213 | 0.9 | 134220 | 153357 | 0.88 | -1915 | -1274 | 1.5 | 2341 | 2789 | 0.84 |
| Streptococcus_thermophilus_LMD-9.3 (leading) | 678 | 715 | 0.95 | 581082 | 596874 | 0.97 | 22631 | 23704 | 0.95 | 1873 | -4490 | -0.42 |
| Streptococcus_thermophilus_LMD-9.3 (lagging) | 142 | 174 | 0.82 | 114054 | 135801 | 0.84 | -1658 | -1703 | 0.97 | 1692 | 3288 | 0.51 |
| Streptococcus_thermophilus_LMG_18311 (leading) | 749 | 749 | 1 | 611763 | 610692 | 1 | 24892 | 24539 | 1.01 | -177 | -4419 | 0.04 |
| Streptococcus_thermophilus_LMG_18311 (lagging) | 180 | 210 | 0.86 | 134616 | 152805 | 0.88 | -2001 | -1400 | 1.43 | 2433 | 3067 | 0.79 |
| Streptomyces_avermitilis (leading) | 1593 | 1826 | 0.87 | 1605549 | 1840992 | 0.87 | -32172 | -21757 | 1.48 | 10743 | 7615 | 1.41 |
| Streptomyces_avermitilis (lagging) | 2054 | 2103 | 0.98 | 2264316 | 2075577 | 1.09 | -48238 | -37068 | 1.3 | 13246 | 20125 | 0.66 |
| Streptomyces_coelicolor (leading) | 1776 | 1680 | 1.06 | 1789264 | 1665159 | 1.07 | -14258 | -27979 | 0.51 | 1686 | 10012 | 0.17 |
| Streptomyces_coelicolor (lagging) | 2118 | 2194 | 0.97 | 2059609 | 2191053 | 0.94 | -68845 | -73597 | 0.94 | 20135 | 19818 | 1.02 |

| Chromosome | Nu of genes 1st half (Nu1) | Nu of genes 2nd half (Nu2) | Nu1/Nu2 | Total length of genes 1st half (Length1) | Total length of genes 2nd half (Length2) | Length1/ Length2 | Cumulative skew (G-C) of genes 1st half (G-C)1 | Cumulative skew (G-C) of genes 2nd half (G-C)2 | (G-C)1/ (G-C)2 | Cumulative skew (A-T) of genes 1st half (A-T)1 | Cumulative skew (A-T) of genes 2nd half (A-T)2 | (A-T)1/ (A-T)2 |
|---|---|---|---|---|---|---|---|---|---|---|---|---|
| Sulfurovum_NBC37-1 (leading) | 803 | 759 | 1.06 | 754287 | 753645 | 1 | 30725 | 31509 | 0.98 | 35498 | 39456 | 0.9 |
| Sulfurovum_NBC37-1 (lagging) | 441 | 434 | 1.02 | 405840 | 396135 | 1.02 | -1299 | 443 | -2.93 | 26097 | 25696 | 1.02 |
| Symbiobacterium_thermophilum_IAM14863 (leading) | 1170 | 1218 | 0.96 | 1114122 | 1134972 | 0.98 | 34322 | 38363 | 0.89 | 658 | -971 | -0.68 |
| Symbiobacterium_thermophilum_IAM14863 (lagging) | 467 | 482 | 0.97 | 442743 | 418644 | 1.06 | -1813 | -332 | 5.46 | -564 | -488 | 1.16 |
| Synechococcus_CC9311 (leading) | 667 | 762 | 0.88 | 519972 | 556428 | 0.93 | 20624 | 22067 | 0.93 | -26650 | -28793 | 0.93 |
| Synechococcus_CC9311 (lagging) | 726 | 736 | 0.99 | 608187 | 587257 | 1.04 | -2827 | -4202 | 0.67 | 12016 | 8482 | 1.42 |
| Synechococcus_CC9605 (leading) | 638 | 648 | 0.98 | 551718 | 510444 | 1.08 | 12608 | 10380 | 1.21 | -20820 | -17884 | 1.16 |
| Synechococcus_CC9605 (lagging) | 675 | 683 | 0.99 | 549897 | 563022 | 0.98 | -4117 | -3552 | 1.16 | 8814 | 10624 | 0.83 |
| Synechococcus_CC9902 (leading) | 586 | 531 | 1.1 | 509022 | 477009 | 1.07 | 19866 | 20697 | 0.96 | -25680 | -24220 | 1.06 |
| Synechococcus_CC9902 (lagging) | 558 | 631 | 0.88 | 494316 | 523422 | 0.94 | -4333 | -5603 | 0.77 | 10945 | 10231 | 1.07 |
| Synechococcus_elongatus_PCC_6301 (leading) | 612 | 582 | 1.05 | 555864 | 2151755 | 0.26 | -3115 | -2629 | 1.18 | -7391 | -7627 | 0.97 |
| Synechococcus_elongatus_PCC_6301 (lagging) | 624 | 708 | 0.88 | 615519 | 648696 | 0.95 | 1907 | 2316 | 0.82 | -10220 | -11944 | 0.86 |
| Synechococcus_elongatus_PCC_7942.2 (leading) | 655 | 674 | 0.97 | 624522 | 609633 | 1.02 | 2778 | 1343 | 2.07 | -10702 | -12206 | 0.88 |
| Synechococcus_elongatus_PCC_7942.2 (lagging) | 660 | 622 | 1.06 | 573576 | 591792 | 0.97 | -3234 | -3369 | 0.96 | -7646 | -7135 | 1.07 |
| Synechococcus_RCC307 (leading) | 615 | 608 | 1.01 | 513030 | 512295 | 1 | 12150 | 10394 | 1.17 | -21050 | -20883 | 1.01 |
| Synechococcus_RCC307 (lagging) | 640 | 671 | 0.95 | 538833 | 539868 | 1 | -2609 | -5042 | 0.52 | 4162 | 5330 | 0.78 |
| Synechococcus_sp_WH8102 (leading) | 641 | 595 | 1.08 | 598758 | 508509 | 1.18 | 15877 | 15007 | 1.06 | -19973 | -15570 | 1.28 |
| Synechococcus_sp_WH8102 (lagging) | 580 | 702 | 0.83 | 514218 | 571939 | 0.9 | -4093 | -1866 | 2.19 | 6323 | 5492 | 1.15 |
| Synechococcus_WH_7803 (leading) | 602 | 611 | 0.99 | 524118 | 546006 | 0.96 | 11053 | 14131 | 0.78 | -23307 | -20999 | 1.11 |
| Synechococcus_WH_7803 (lagging) | 686 | 633 | 1.08 | 577020 | 556461 | 1.04 | -8492 | -6066 | 1.4 | 4562 | 2525 | 1.81 |
| Synechocystis_PCC6803 (leading) | 849 | 728 | 1.17 | 2751332 | 738603 | 3.73 | 10234 | 9667 | 1.06 | -2332 | -1460 | 1.6 |
| Synechocystis_PCC6803 (lagging) | 782 | 812 | 0.96 | 742875 | 806862 | 0.92 | 8832 | 9893 | 0.89 | -425 | -2439 | 0.17 |
| Syntrophobacter_fumaroxidans_MPOB (leading) | 1046 | 1031 | 1.01 | 1047084 | 1038738 | 1.01 | 15648 | 15750 | 0.99 | 16554 | 9860 | 1.68 |
| Syntrophobacter_fumaroxidans_MPOB (lagging) | 967 | 1019 | 0.95 | 993390 | 1023807 | 0.97 | 15490 | 18779 | 0.82 | 10412 | 14238 | 0.73 |
| Syntrophomonas_wolfei_Goettingen (leading) | 901 | 994 | 0.91 | 902373 | 942105 | 0.96 | 51082 | 57872 | 0.88 | 29109 | 28053 | 1.04 |
| Syntrophomonas_wolfei_Goettingen (lagging) | 318 | 290 | 1.1 | 318048 | 268362 | 1.19 | 8985 | 5504 | 1.63 | 7543 | 6226 | 1.21 |
| Syntrophus_aciditrophicus_SB (leading) | 927 | 845 | 1.1 | 840027 | 743064 | 1.13 | 33381 | 24915 | 1.34 | 13614 | 9979 | 1.36 |
| Syntrophus_aciditrophicus_SB (lagging) | 613 | 782 | 0.78 | 559809 | 688713 | 0.81 | 2993 | 3517 | 0.85 | 13108 | 11584 | 1.13 |
| Thermoanaerobacter_tengcongensis (leading) | 1085 | 1155 | 0.94 | 1007223 | 1026108 | 0.98 | 90509 | 91129 | 0.99 | 72576 | 73923 | 0.98 |
| Thermoanaerobacter_tengcongensis (lagging) | 180 | 167 | 1.08 | 164952 | 144831 | 1.14 | 4985 | 4607 | 1.08 | 15701 | 12792 | 1.23 |
| Thermobifida_fusca_YX (leading) | 948 | 937 | 1.01 | 933135 | 957954 | 0.97 | -20570 | -27726 | 0.74 | 14659 | 15140 | 0.97 |
| Thermobifida_fusca_YX (lagging) | 625 | 599 | 1.04 | 616407 | 593211 | 1.04 | -13437 | -15281 | 0.88 | -1078 | -320 | 3.37 |
| Thermosipho_melanesiensis_BI429 (leading) | 380 | 453 | 0.84 | 353400 | 407118 | 0.87 | 16917 | 21319 | 0.79 | 31793 | 35577 | 0.89 |
| Thermosipho_melanesiensis_BI429 (lagging) | 518 | 527 | 0.98 | 514287 | 476745 | 1.08 | 43581 | 44771 | 0.97 | 30078 | 26084 | 1.15 |
| Thermosynechococcus_elongatus (leading) | 652 | 601 | 1.08 | 588726 | 585618 | 1.01 | -552 | -2662 | 0.21 | -7078 | -10554 | 0.67 |
| Thermosynechococcus_elongatus (lagging) | 599 | 623 | 0.96 | 573860 | 582492 | 0.99 | -1852 | -2913 | 0.64 | -9549 | -11571 | 0.83 |
| Thermotoga_maritima (leading) | 533 | 478 | 1.12 | 513015 | 429679 | 1.19 | 29615 | 23082 | 1.28 | 28950 | 28034 | 1.03 |
| Thermotoga_maritima (lagging) | 370 | 476 | 0.78 | 368973 | 447281 | 0.82 | 15816 | 21021 | 0.75 | 24257 | 25949 | 0.93 |
| Thermotoga_petrophila_RKU-1 (leading) | 355 | 430 | 0.83 | 344796 | 404676 | 0.85 | 14937 | 21052 | 0.71 | 23205 | 24620 | 0.94 |
| Thermotoga_petrophila_RKU-1 (lagging) | 524 | 475 | 1.1 | 523407 | 453747 | 1.15 | 30044 | 23413 | 1.28 | 28919 | 29850 | 0.97 |
| Thiobacillus_denitrificans_ATCC_25259 (leading) | 793 | 872 | 0.91 | 785592 | 815235 | 0.96 | -7323 | -6899 | 1.06 | 6789 | 5938 | 1.14 |
| Thiobacillus_denitrificans_ATCC_25259 (lagging) | 581 | 580 | 1 | 563799 | 527943 | 1.07 | -20589 | -19128 | 1.08 | 7552 | 7407 | 1.02 |
| Thiomicrospira_crunogena_XCL-2 (leading) | 751 | 607 | 1.24 | 752940 | 605067 | 1.24 | 46159 | 36530 | 1.26 | 2543 | -131 | -19.41 |
| Thiomicrospira_crunogena_XCL-2 (lagging) | 371 | 466 | 0.8 | 344304 | 479382 | 0.72 | -3197 | -1564 | 2.04 | 14609 | 17858 | 0.82 |
| Thiomicrospira_denitrificans_ATCC_33889 (leading) | 653 | 613 | 1.07 | 636753 | 601473 | 1.06 | 34897 | 33736 | 1.03 | 37486 | 27735 | 1.35 |
| Thiomicrospira_denitrificans_ATCC_33889 (lagging) | 384 | 446 | 0.86 | 380901 | 425478 | 0.9 | 10991 | 14342 | 0.77 | 23856 | 25014 | 0.95 |
| Treponema_denticola_ATCC_35405 (leading) | 714 | 796 | 0.9 | 689664 | 755628 | 0.91 | 22486 | 26193 | 0.86 | 23402 | 26637 | 0.88 |
| Treponema_denticola_ATCC_35405 (lagging) | 656 | 600 | 1.09 | 609597 | 550467 | 1.11 | 16460 | 11282 | 1.46 | 34289 | 34117 | 1.01 |
| Treponema_pallidum (leading) | 361 | 316 | 1.14 | 354374 | 325472 | 1.09 | 29579 | 24853 | 1.19 | -16804 | -14904 | 1.13 |
| Treponema_pallidum (lagging) | 163 | 195 | 0.84 | 168366 | 208683 | 0.81 | -1610 | -2947 | 0.55 | -132 | 359 | -0.37 |
| Trichodesmium_erythraeum_IMS101 (leading) | 1083 | 1136 | 0.95 | 1137267 | 1171989 | 0.97 | 47583 | 45651 | 1.04 | 35512 | 37642 | 0.94 |
| Trichodesmium_erythraeum_IMS101 (lagging) | 1102 | 1129 | 0.98 | 1175694 | 1158630 | 1.01 | 50537 | 49808 | 1.01 | 35017 | 34582 | 1.01 |
| Tropheryma_whipplei_TW08_27 (leading) | 305 | 273 | 1.12 | 294109 | 282790 | 1.04 | 10679 | 8750 | 1.22 | -11641 | -8147 | 1.43 |
| Tropheryma_whipplei_TW08_27 (lagging) | 95 | 109 | 0.87 | 102441 | 103408 | 0.99 | 3718 | 3724 | 1 | 4161 | 1705 | 2.44 |
| Tropheryma_whipplei_Twist (leading) | 255 | 308 | 0.83 | 260664 | 293514 | 0.89 | 9222 | 10550 | 0.87 | -9902 | -9912 | 1 |
| Tropheryma_whipplei_Twist (lagging) | 121 | 123 | 0.98 | 121776 | 122775 | 0.99 | 4514 | 4692 | 0.96 | 500 | 3897 | 0.13 |
| Ureaplasma_urealyticum (leading) | 224 | 175 | 1.28 | 233433 | 220275 | 1.06 | 7109 | 5164 | 1.38 | 13176 | 13261 | 0.99 |
| Ureaplasma_urealyticum (lagging) | 108 | 106 | 1.02 | 103557 | 129147 | 0.8 | 2789 | 3047 | 0.92 | 6160 | 6766 | 0.91 |
| Verminephrobacter_eiseniae_EF01-2.2 (leading) | 1247 | 1344 | 0.93 | 1329111 | 1315902 | 1.01 | -5856 | -5529 | 1.06 | 8967 | 8759 | 1.02 |
| Verminephrobacter_eiseniae_EF01-2.2 (lagging) | 1154 | 1162 | 0.99 | 1150824 | 1159638 | 0.99 | -8822 | -11701 | 0.75 | 5572 | 11149 | 0.5 |
| Vibrio_cholerae (leading) | 790 | 830 | 0.95 | 769718 | 790403 | 0.97 | 32414 | 30782 | 1.05 | -3580 | 1897 | -1.89 |
| Vibrio_cholerae (lagging) | 584 | 537 | 1.09 | 531733 | 509979 | 1.04 | -1713 | 1025 | -1.67 | 7360 | 5052 | 1.46 |
| Vibrio_cholerae_O395.2 (leading) | 731 | 758 | 0.96 | 724659 | 2283336 | 0.32 | 23786 | 25667 | 0.93 | -585 | 1196 | -0.49 |
| Vibrio_cholerae_O395.2 (lagging) | 650 | 602 | 1.08 | 627783 | 578493 | 1.09 | 7625 | 7754 | 0.98 | 8874 | 4229 | 2.1 |
| Vibrio_fischeri_ES114 (leading) | 767 | 777 | 0.99 | 763014 | 745257 | 1.02 | 37806 | 38899 | 0.97 | 2712 | 4868 | 0.56 |
| Vibrio_fischeri_ES114 (lagging) | 531 | 499 | 1.06 | 505938 | 491112 | 1.03 | 1513 | 3160 | 0.48 | 10615 | 11181 | 0.95 |
| Vibrio_parahaemolyticus (leading) | 896 | 953 | 0.94 | 857604 | 862269 | 0.99 | 33948 | 32830 | 1.03 | 7368 | 12061 | 0.61 |

| Chromosome | Nu of genes 1st half (Nu1) | Nu of genes 2nd half (Nu2) | Nu1/Nu2 | Total length of genes 1st half (Length1) | Total length of genes 2nd half (Length2) | Length1/ Length2 | Cumulative skew (G-C) of genes 1st half (G-C)1 | Cumulative skew (G-C) of genes 2nd half (G-C)2 | (G-C)1/ (G-C)2 | Cumulative skew (A-T) of genes 1st half (A-T)1 | Cumulative skew (A-T) of genes 2nd half (A-T)2 | (A-T)1/ (A-T)2 |
|---|---|---|---|---|---|---|---|---|---|---|---|---|
| Vibrio_parahaemolyticus (lagging) | 635 | 595 | 1.07 | 590037 | 545049 | 1.08 | -2014 | -1069 | 1.88 | 19185 | 15480 | 1.24 |
| Vibrio_vulnificus_CMCP6 (leading) | 695 | 608 | 1.14 | 651003 | 613314 | 1.06 | 13575 | 10376 | 1.31 | 10746 | 9708 | 1.11 |
| Vibrio_vulnificus_CMCP6 (lagging) | 797 | 826 | 0.96 | 740658 | 756987 | 0.98 | 21889 | 20592 | 1.06 | 8071 | 5451 | 1.48 |
| Vibrio_vulnificus_YJ016.2 (leading) | 962 | 1025 | 0.94 | 900000 | 912180 | 0.99 | 37861 | 39384 | 0.96 | 3081 | 4032 | 0.76 |
| Vibrio_vulnificus_YJ016.2 (lagging) | 668 | 603 | 1.11 | 608550 | 551028 | 1.1 | -3157 | -484 | 6.52 | 12551 | 11758 | 1.07 |
| Wolbachia_endosymbiont_of_Brugia_malayi_TRS (leading | 193 | 232 | 0.83 | 177234 | 217488 | 0.81 | 9809 | 14295 | 0.69 | 10577 | 8597 | 1.23 |
| Wolbachia_endosymbiont_of_Brugia_malayi_TRS (lagging | 215 | 164 | 1.31 | 184218 | 144372 | 1.28 | 10403 | 5922 | 1.76 | 8901 | 10940 | 0.81 |
| Wolbachia_endosymbiont_of_Drosophila_melanogaster (le | 275 | 290 | 0.95 | 235317 | 240954 | 0.98 | 13992 | 13458 | 1.04 | 13781 | 13760 | 1 |
| Wolbachia_endosymbiont_of_Drosophila_melanogaster (la | 299 | 330 | 0.91 | 264846 | 275070 | 0.96 | 15787 | 13067 | 1.21 | 19065 | 15381 | 1.24 |
| Wolinella_succinogenes (leading) | 617 | 588 | 1.05 | 593301 | 554919 | 1.07 | 32351 | 25596 | 1.26 | -1474 | 4985 | -0.3 |
| Wolinella_succinogenes (lagging) | 401 | 435 | 0.92 | 398127 | 437010 | 0.91 | -12221 | -7566 | 1.62 | 8882 | 11444 | 0.78 |
| Xanthobacter_autotrophicus_Py2.2 (leading) | 1136 | 1169 | 0.97 | 1117017 | 1115091 | 1 | -11671 | -26842 | 0.43 | -10988 | -7745 | 1.42 |
| Xanthobacter_autotrophicus_Py2.2 (lagging) | 1225 | 1215 | 1.01 | 1220904 | 1210236 | 1.01 | -32983 | -12882 | 2.56 | -5901 | -11338 | 0.52 |
| Xanthomonas_campestris (leading) | 1165 | 1138 | 1.02 | 1210986 | 1218222 | 0.99 | 22209 | 20546 | 1.08 | 530 | -5682 | -0.09 |
| Xanthomonas_campestris (lagging) | 947 | 930 | 1.02 | 946383 | 932691 | 1.01 | -7669 | -8737 | 0.88 | 8000 | 6844 | 1.17 |
| Xanthomonas_campestris_8004 (leading) | 1181 | 1201 | 0.98 | 1226541 | 1254093 | 0.98 | 19704 | 24761 | 0.8 | -4603 | -1106 | 4.16 |
| Xanthomonas_campestris_8004 (lagging) | 927 | 963 | 0.96 | 948219 | 937557 | 1.01 | -8766 | -9802 | 0.89 | 6921 | 8045 | 0.86 |
| Xanthomonas_campestris_vesicatoria_85-10.5 (leading) | 1276 | 1283 | 0.99 | 1282947 | 1336164 | 0.96 | 22950 | 19720 | 1.16 | 241 | -3838 | -0.06 |
| Xanthomonas_campestris_vesicatoria_85-10.5 (lagging) | 980 | 947 | 1.03 | 974721 | 932130 | 1.05 | -8753 | -10013 | 0.87 | 8720 | 7797 | 1.12 |
| Xanthomonas_citri (leading) | 1208 | 1178 | 1.03 | 1249317 | 1266393 | 0.99 | 22852 | 21558 | 1.06 | -2949 | -4201 | 0.7 |
| Xanthomonas_citri (lagging) | 955 | 970 | 0.98 | 982512 | 963513 | 1.02 | -5045 | -9740 | 0.52 | 7547 | 7631 | 0.99 |
| Xanthomonas_oryzae_KACC10331 (leading) | 1108 | 1154 | 0.96 | 1056153 | 1142148 | 0.92 | 22535 | 21666 | 1.04 | -1490 | -1772 | 0.84 |
| Xanthomonas_oryzae_KACC10331 (lagging) | 994 | 888 | 1.12 | 922110 | 844380 | 1.09 | -988 | -2966 | 0.33 | 7682 | 8886 | 0.86 |
| Xanthomonas_oryzae_MAFF_311018 (leading) | 1170 | 1225 | 0.96 | 1085889 | 1223097 | 0.89 | 23680 | 25600 | 0.93 | 987 | -765 | -1.29 |
| Xanthomonas_oryzae_MAFF_311018 (lagging) | 1062 | 914 | 1.16 | 979320 | 871752 | 1.12 | 784 | -2260 | -0.35 | 7306 | 9582 | 0.76 |
| Xylella_fastidiosa (leading) | 847 | 751 | 1.13 | 700092 | 638849 | 1.1 | 44788 | 10962 | 4.09 | -38938 | -9814 | 3.97 |
| Xylella_fastidiosa (lagging) | 533 | 634 | 0.84 | 404016 | 494034 | 0.82 | -21844 | 3302 | -6.62 | 20416 | 3760 | 5.43 |
| Xylella_fastidiosa_Temecula1.2 (leading) | 580 | 606 | 0.96 | 579792 | 610194 | 0.95 | 42275 | 31450 | 1.34 | -33157 | -27468 | 1.21 |
| Xylella_fastidiosa_Temecula1.2 (lagging) | 427 | 420 | 1.02 | 386193 | 392190 | 0.98 | -14093 | -18312 | 0.77 | 14238 | 18798 | 0.76 |
| Yersinia_enterocolitica_8081.2 (leading) | 1108 | 1171 | 0.95 | 1084469 | 1140272 | 0.95 | 48257 | 51628 | 0.93 | -15093 | -14593 | 1.03 |
| Yersinia_enterocolitica_8081.2 (lagging) | 857 | 842 | 1.02 | 826528 | 804800 | 1.03 | 10358 | 9300 | 1.11 | 669 | 4 | 167.25 |
| Yersinia_pestis_Antiqua.4 (leading) | 1126 | 1130 | 1 | 1066734 | 1069275 | 1 | 39396 | 41614 | 0.95 | -7174 | -6075 | 1.18 |
| Yersinia_pestis_Antiqua.4 (lagging) | 967 | 943 | 1.03 | 897687 | 918795 | 0.98 | 17807 | 18949 | 0.94 | 34 | 1960 | 0.02 |
| Yersinia_pestis_biovar_Mediaevails (leading) | 1056 | 988 | 1.07 | 947997 | 970911 | 0.98 | 36637 | 36250 | 1.01 | -8950 | -7435 | 1.2 |
| Yersinia_pestis_biovar_Mediaevails (lagging) | 941 | 909 | 1.04 | 912552 | 914880 | 1 | 17679 | 23127 | 0.76 | -1325 | -281 | 4.72 |
| Yersinia_pestis_CO92.4 (leading) | 1094 | 1067 | 1.03 | 1038750 | 1031589 | 1.01 | 43815 | 41579 | 1.05 | -7993 | -11344 | 0.7 |
| Yersinia_pestis_CO92.4 (lagging) | 839 | 884 | 0.95 | 837748 | 823734 | 1.02 | 10888 | 18318 | 0.59 | 2112 | 1788 | 1.18 |
| Yersinia_pestis_KIM (leading) | 1192 | 1121 | 1.06 | 1106703 | 1080807 | 1.02 | 49026 | 44347 | 1.11 | -12985 | -7720 | 1.68 |
| Yersinia_pestis_KIM (lagging) | 861 | 911 | 0.95 | 794670 | 849819 | 0.94 | 9569 | 12908 | 0.74 | 2805 | 1395 | 2.01 |
| Yersinia_pestis_Nepal516.3 (leading) | 1145 | 1216 | 0.94 | 1095612 | 1192785 | 0.92 | 45225 | 53511 | 0.85 | -10181 | -12400 | 0.82 |
| Yersinia_pestis_Nepal516.3 (lagging) | 857 | 762 | 1.12 | 796803 | 714513 | 1.12 | 11958 | 6256 | 1.91 | 4211 | 4943 | 0.85 |
| Yersinia_pestis_Pestoides_F.3 (leading) | 923 | 1098 | 0.84 | 882426 | 1083099 | 0.81 | 22464 | 48481 | 0.46 | -952 | -15548 | 0.06 |
| Yersinia_pestis_Pestoides_F.3 (lagging) | 1032 | 796 | 1.3 | 969378 | 772134 | 1.26 | 31290 | 10630 | 2.94 | -1742 | 3074 | -0.57 |
| Yersinia_pseudotuberculosis_IP32953.3 (leading) | 1129 | 1140 | 0.99 | 1135386 | 1178248 | 0.96 | 52443 | 49517 | 1.06 | -14527 | -11939 | 1.22 |
| Yersinia_pseudotuberculosis_IP32953.3 (lagging) | 853 | 778 | 1.1 | 827403 | 774195 | 1.07 | 5576 | 11726 | 0.48 | 4299 | 5589 | 0.77 |
| Yersinia_pseudotuberculosis_IP_31758.3 (leading) | 1169 | 1270 | 0.92 | 1173106 | 1206036 | 0.97 | 50898 | 54694 | 0.93 | -14301 | -14594 | 0.98 |
| Yersinia_pseudotuberculosis_IP_31758.3 (lagging) | 848 | 836 | 1.01 | 785187 | 760605 | 1.03 | 10403 | 5691 | 1.83 | 7308 | 2014 | 3.63 |
| Zymomonas_mobilis_ZM4 (leading) | 575 | 518 | 1.11 | 527310 | 468843 | 1.12 | 24019 | 8352 | 2.88 | -18357 | -9285 | 1.98 |
| Zymomonas_mobilis_ZM4 (lagging) | 434 | 470 | 0.92 | 373839 | 401865 | 0.93 | -1886 | 9192 | -0.21 | 2899 | -6403 | -0.45 |
